# Chemoenzymatic synthesis of heparan sulfate and heparin oligosaccharides and NMR analysis: paving the way to a diverse library for glycobiologists†
†Electronic supplementary information (ESI) available: Materials, detailed experimental protocols, supporting schemes and figures, synthetic methods, analytical data including HPLC profiles, mass and NMR spectra of synthesized oligosaccharides. See DOI: 10.1039/c7sc03541a


**DOI:** 10.1039/c7sc03541a

**Published:** 2017-09-21

**Authors:** Xing Zhang, Vijayakanth Pagadala, Hannah M. Jester, Andrew M. Lim, Truong Quang Pham, Anna Marie P. Goulas, Jian Liu, Robert J. Linhardt

**Affiliations:** a Department of Chemistry and Chemical Biology , Rensselaer Polytechnic Institute , Troy , New York 12180 , USA . Email: linhar@rpi.edu; b Glycan Therapeutics , LLC , Chapel Hill , North Carolina 27599 , USA; c Division of Chemical Biology and Medicinal Chemistry , Eshelman School of Pharmacy , University of North Carolina , Chapel Hill , North Carolina 27599 , USA . Email: liuj@email.unc.edu

## Abstract

Heparan sulfate (HS) is a member of the glycosaminoglycans (GAG) family that plays essential roles in biological processes from animal sources. Heparin, a highly sulfated form of HS, is widely used as anticoagulant drug worldwide. The high diversity and complexity of HS and heparin represent a roadblock for structural characterization and biological activity studies. Access to structurally defined oligosaccharides is critical for the successful development of HS and heparin structure–activity relationships. In this study, a library of **66** HS and heparin oligosaccharides covering different sulfation patterns and sizes was prepared through an efficient method of chemoenzymatic synthesis. A systematic nuclear magnetic resonance spectroscopy study was firstly undertaken for every oligosaccharide in the library. In addition to the availability of different oligosaccharides, this work also provides spectroscopic data helpful for characterizing more complicated polysaccharide structures providing a safeguard to ensure the quality of the drug heparin. This HS/heparin library will be useful for activity screening and facilitate future structure–activity relationship studies.

## Abstract 2

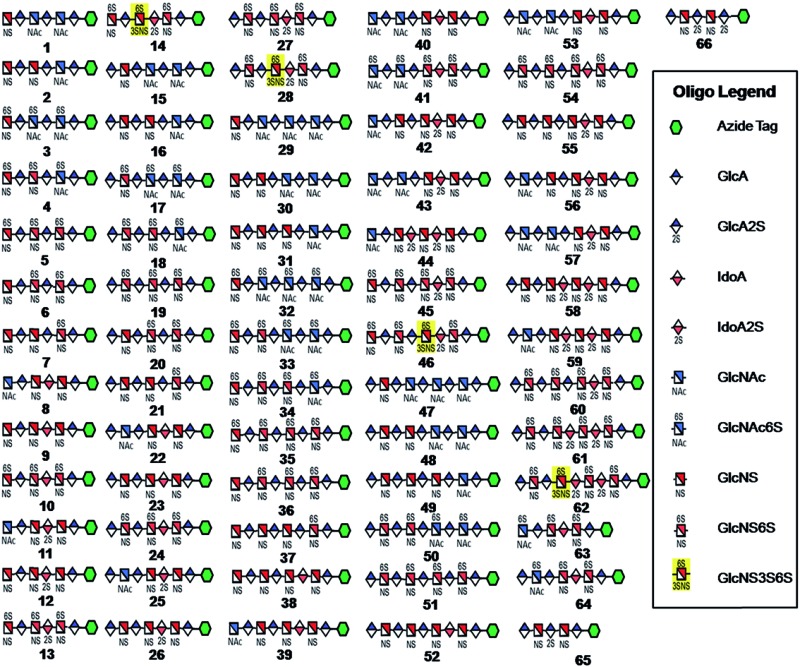
A library of diverse heparan sulfate (HS) oligosaccharides was chemoenzymatically synthesized and systematically studied using NMR.

## Introduction

Heparan sulfate (HS) glycosaminoglycan (GAG) is a linear sulfated polysaccharide consists of repeating 1→4 glycosidically linked disaccharide units comprised of alternating uronic acid residues, β-d-glucuronic acid (GlcA) or α-l-iduronic acid (IdoA) and α-d-glucosamine (GlcN) residues.^1^ The HS disaccharide units can be substituted with *N*-acetyl (Ac) or *N*-sulfo (S) groups and *O*-sulfo (S) groups (at the 3-*O*- and 6-*O*-positions of the GlcN residue and 2-*O*-position of the uronic acid residues).^2^ HS is widely expressed on mammalian cell surfaces and in the extracellular matrix and plays essential roles in a number of biological processes, controlling angiogenesis, embryonic development, inflammatory responses, lipid metabolism, infectious disease, tumor metastasis and blood coagulation.^3^ Heparin is a special form of HS, found within specialized granulated cells, having a higher level of sulfation, a higher IdoA content, and displaying prominent anticoagulant activity.^4^ There are an average of 2.6 sulfo groups and 80–90% IdoA per disaccharide unit in heparin, compared with only 0.6 sulfo groups and 20% IdoA per disaccharide unit in a typical HS.^5^ A pentasaccharide sequence motif present within HS and heparin polysaccharides including, →4)GlcNS6S(1→4)GlcA(1→4)GlcNS3S6S(1→4)IdoA2S(1→4)GlcNS6S(1→, is responsible for its specific binding to the serine protease inhibitor (serpin) antithrombin III (AT).^6^ A repeating trisulfated disaccharide sequence →4)IdoA2S(1→4)GlcNS6S(1→ corresponds to heparin's thrombin (or factor IIa, FIIa) binding site and facilitates the assembly of the ternary heparin–AT–FIIa complex required for heparin's global anticoagulant activity.^7^

Unfractionated heparins (UFHs) and low molecular weight heparins (LMWHs) are polypharmacological agents, complex mixtures of molecules prepared from animal tissues.^8^ Due to the poorly regulated supply chain of pharmaceutical heparin, a worldwide distribution of oversulfated chondroitin sulfate (OCSC) contaminated heparin in 2007 was discovered and associated with over 200 deaths in the U.S., adversely affecting the purity and safety of animal-sourced heparins.^9^ Another potential threat to the supply chain of heparin is that other bioactive entities, such as prions and viruses, might remain associated with the HS and heparin chains in animal extracts.^10^ Thus, the cost-effective preparation of a more structurally defined heparin from non-animal sources is highly desirable.^11^ Meanwhile, developing more reliable structural characterization techniques is critically important to safeguard the quality of heparin drugs.^12^

Heparin impurities and contaminants are often other sulfated polysaccharides that are challenging to distinguish as impurities or contaminants when present in the heterogeneous and polydisperse drug heparin. Often there are only relatively subtle structural features, such as sugar epimers, sulfo group position, or differences in linkage position and anomeric configuration, unique to these impurities or contaminants.^13^ Nuclear magnetic resonance (NMR) spectroscopy is widely utilized for such sophisticated structure analysis and is considered one of the best methods for determining subtle structural differences in heterogeneous and polydisperse polysaccharides, such as identifying and quantifying the dermatan sulfate impurity and the oversulfated chondroitin sulfate heparin contaminant, and, thus, playing an important role in heparin quality control.^14^ Pure HS and heparin oligosaccharide library standards have not been widely available and in the past have been isolated through the partial depolymerization of HS or heparin.^15^ This makes NMR signal assignment of HS and heparin polysaccharides, often requiring multi-dimensional techniques, very challenging and time-consuming.^16^ The synthesis of a library of structurally defined HS and heparin oligosaccharides in sufficient amounts to serve as NMR standards for the analysis of current heparin drugs, is especially beneficial for the analysis of structurally important or “rare” residues, such as GlcNS3S6S, GlcA2S and IdoA residues.^17^ HS and heparin oligosaccharides of moderate size, with 5 to 10 saccharide units (pentasaccharides to decasaccharides), are generally required for high-affinity interactions with heparin-binding proteins and are responsible for many of heparin's biological activities.^18^ Moreover, a library comprised of moderate sized oligosaccharides would also support structure–activity relationship (SAR) studies.^19^

There are generally two approaches for the target driven synthesis of a library of structurally defined HS and heparin oligosaccharides. The first approach relies on a purely chemical synthesis method, based on repetitive steps of protection, activation, coupling and de-protection, and is incredibly challenging for the preparation of oligosaccharides larger than pentasaccharides.^20^ For example, Arixtra®, an FDA-approved pentasaccharide anticoagulant drug, requires as many as 60 chemical steps, and is produced in a reported overall yield is only 0.1%.^21^ In addition, during the preparation, there are many separation steps needed to remove undesirable isomers (especially the incorrect anomers) as well as the unavoidable side products that add cost and decrease overall yields. The second approach, chemoenzymatic synthesis, relies on combined chemical and enzymatic methods, mimicking the biosynthetic pathway of heparin, and represents a promising strategy to address many of these synthetic challenges.^22^ The enzymatic component of synthesis is usually performed under mild conditions (20–37 °C) using aqueous “green” solvents catalyzing glycosylation reactions with exquisite stereoselectivity (α- or β-glycosidic linkages) and sulfonation reactions with excellent regioselectivity, without the need for repetitive protection and deprotection steps.^23^ In recent years, a number of reports on the chemoenzymatic synthesis of structurally defined oligosaccharides have demonstrated the feasibility of preparing those oligosaccharides that have been difficult to synthesize by traditional organic approaches.^24^ Chen and coworkers24*a* have developed an efficient one-pot multi-enzyme (OPME) system utilizing with a series of unnatural sugar nucleotides to synthesize heparin oligosaccharides, where oligosaccharides were prepared without the required isolation or purification of intermediates. Wang and coworkers24*b* have reported a practical chemoenzymatic strategy, core synthesis/enzymatic extension (CSEE), for the rapid production of diverse *N*-glycans. In our previous efforts,24c–f we demonstrated that chemoenzymatic synthesis, starting from an inexpensive commercially available acceptor, *p*-nitrophenyl glucuronide (GlcA-*p*NP), which is ultraviolet detectable, is capable of generating a series of heparin oligosaccharides in good overall yield and possessed excellent anticoagulant activity. Herein, we have utilized another ultraviolet detectable, addressable acceptor, *N*-(6-azidohexanamidyl) *p*-aminophenyl glucuronide (GlcA-*p*NA-N_3_), for chemoenzymatic synthesis and a systematic NMR study, and produced the largest library of **66** HS and heparin oligosaccharides to date.

## Results

### Key intermediate synthesis

**Scheme 1:**
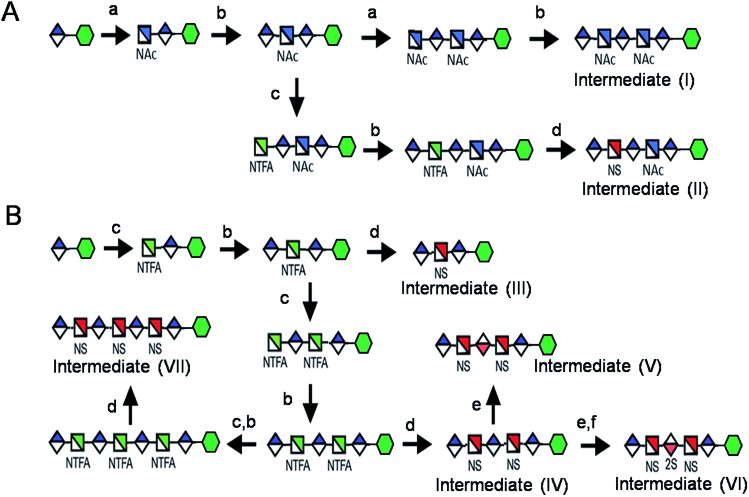
Route for the chemoenzymatic synthesis of seven key intermediates. Reagents and conditions: (a) pmHS2, GlcNAc; (b) pmHS2, GlcA; (c) pmHS2, GlcNTFA; (d) de-*N*-trifluoroacetylation, NST, PAPS; (e) C_5_-epimerase; (f) 2-OST, PAPS. PmHS2, *Pasteurella multocida* heparosan synthase-2; NST, *N*-sulfotransferase; 2-OST, 2-*O*-sulfotransferase; PAPS, 3′-phosphoadenosine-5′-phosphosulfate. Each intermediate was purified to minimum 97% purity by HPLC with a TSKgel DNA-NPR column (ESI).†

**Fig. 1:**
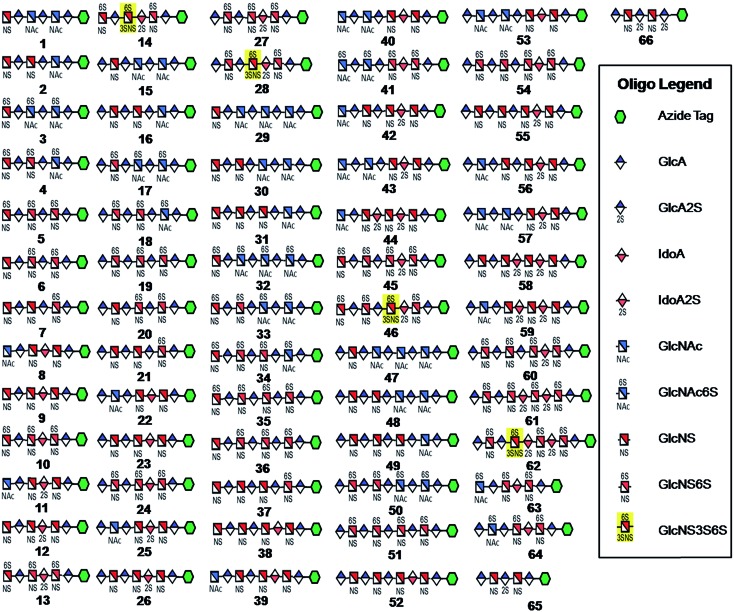
HS and heparin oligosaccharides **1–66** were synthesized using a chemoenzymatic approach. The azide tag is *N*-(6-azidohexanamidyl) *p*-aminophenyl (*p*NA-N_3_) and the chemical structure is presented in Fig. 2A.

Seven key intermediates **I–VII** (Scheme 1 and ESI†), of sizes ranging from trisaccharide to heptasaccharide, were designed to prepare the target library of **66** oligosaccharides. The GlcN residues of pentasaccharide intermediate **I** were both GlcNAc and pentasaccharide intermediate **II** contained one GlcNAc and one GlcNS residue. These were primarily used to prepare oligosaccharides having low sulfation levels such as those typically encountered in HS (Scheme 1A). The GlcN residues of intermediates **III–VII** were all GlcNS and represent versatile precursors to access highly sulfated heparin oligosaccharides (Scheme 1B). Oligosaccharide synthesis was initiated on a β-GlcA(1→*p*NA-N_3_) monosaccharide acceptor and extended with UDP-sugar donors using a chemoenzymatic approach. Heparosan synthase-2 (pmHS2) from *Pasteurella multocida*22*a* was used to elongate this monosaccharide acceptor, to appropriate sized backbones **I–VII**. GlcNTFA containing backbones were then subjected to chemical de-*N*-trifluoracetylation and modification by *N*-sulfotransferase (NST) resulting in key intermediates **II–IV** and **VII**. Key intermediates **VI** and **VII** were subjected to additional enzymatic modification relying on C_5_-epimerase, 2-*O*-sulfotransferase (2-OST), 6-*O*-sulfotransferases (6-OSTs) and 3-*O*-sulfotransferases (3-OSTs). For example, to synthesize intermediates **IV** through **VI**, GlcA-*p*NA-N_3_ was first repetitively elongated using pmHS2 with UDP-*N*-trifluoroacetyl glucosamine (UDP-GlcNTFA) donor and UDP-glucuronic acid (UDP-GlcA), to form pentasaccharide, GlcA(1→4)GlcNTFA(1→4)GlcA(1→4) GlcNTFA(1→4)GlcA-*p*NA-N_3_ (Scheme 1B). The resulting GlcNTFA residues were then converted to GlcNS by removing the trifluoroacetyl group under mild alkaline conditions followed by the introduction of *N*-sulfo groups using NST and 3′-phosphoadenosine-5′-phosphosulfate (PAPS) to obtain intermediate **IV**. Treatment of intermediate (**IV**) with C_5_-epimerase and 2-OST afforded IdoA2S-containing intermediate (**VI**) for further modification. C_5_-epimerase is known to be a bi-directional enzyme, catalyzing both the forward and reverse reaction and affording an equilibrium mixture of GlcA and IdoA residues.^25^ While the GlcA residue is thermodynamically favored, the IdoA residue can be selectively generated in the presence of 2-OST and PAPS to afford IdoA2S, which cannot be converted by C_5_-epimerase to GlcA2S.17b,c However, interestingly, we obtained IdoA containing intermediate **V** by treating with C_5_-epimerase alone. This resulting intermediate **V** could be applied into the preparation of more structurally complex IdoA-containing heparin oligosaccharides (Fig. 1). The sequence of enzymatic reactions needs to be carefully controlled for the synthesis of each desired oligosaccharide target. For example, C_5_-epimerase can only be used after the introduction of *N*-sulfo groups, as it will only act on a GlcA residue that is between two GlcNS residues. The remaining key intermediates were similarly synthesized (ESI†).

### Library oligosaccharides synthesis

**Fig. 2:**
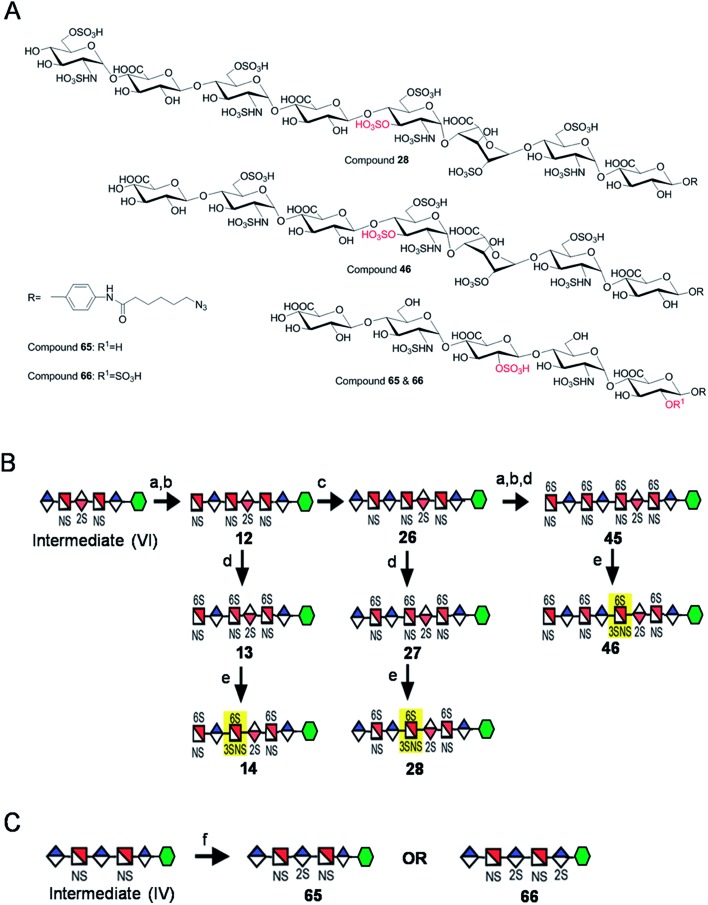
Structures and synthetic schemes for different HS and heparin oligosaccharides. Panel A shows the structure of the compounds **28**, **46**, **65** and **66**. Panel B shows the scheme for the synthesis of GlcNS3S6S containing compounds and panel C for the GlcA2S containing compounds. Reagents and conditions: (a) pmHS2, GlcNTFA; (b) deacetylation, NST, PAPS; (c) pmHS2, GlcA; (d) 6-OST, PAPS; (e) 3-OST-1, PAPS; (f) 2-OST, PAPS.

**Fig. 3:**
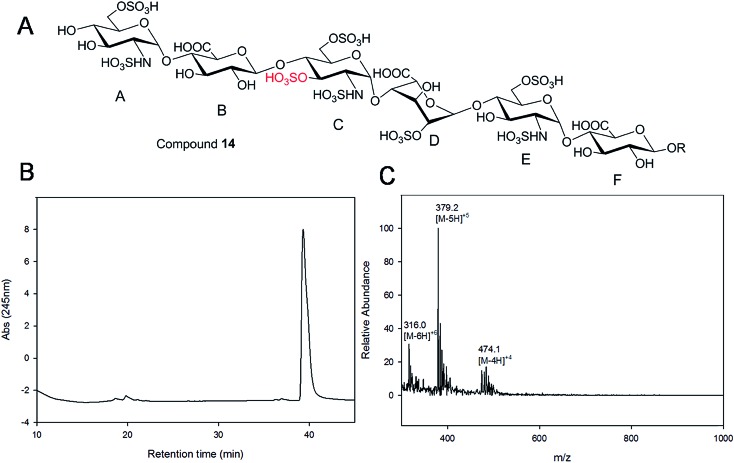
Purity and structure analysis of hexasaccharide **14**. Panel A shows the chemical structure of hexasaccharide **14**. Panel B shows the chromatogram of TSKgel DNA-NPR HPLC analysis. Panel C shows the ESI-MS spectrum of hexasaccharide **14**. The molecular ions carrying 4, 5 and 6 negative charges are indicated.

Starting from pentasaccharide intermediate **VI**, GlcA(1→4)GlcNS(1→4)IdoA2S(1→4) GlcNS(1→4)GlcA-*p*NA-N_3_ (where the residues are labeled alphabetically with the **A** residue GlcA at the non-reducing end and the **E** residue GlcA at the reducing end) three different GlcNS3S6S-containing oligosaccharides **14**, **28**, **46**, were prepared (Fig. 2A and B). Intermediate **VI** was first elongated with pmHS2 and UDP-GlcNTFA to afford the hexasaccharide. After NTFA group deprotection, and subsequent enzymatic *N*-sulfonation and *O*-sulfonation, catalyzed by *N*-sulfotransferase, 2-*O*-sulfotransferase (2-OST), 6-*O*-sulfotransferase (6-OST-1 and 6-OST-3) and 3-*O*-sulfotransferase-1 (3-OST-1), GlcNS3S6S-containing hexasaccharide **14** was obtained (Fig. 2B and 3A). Following the same approach, pmHS2 and UDP-GlcA were employed to construct heptasaccharide **28** and octasaccharide **46**. Chemoenzymatic synthesis requires careful reaction scheme design, especially the selection of an appropriate modification sequence. For example, as residue **C** is flanked by a GlcA residue at its non-reducing end, 3-OST-1 selectively adds a 3-*O*-sulfo group to residue **C** rather than residue **E** to generate compound **14** (Fig. 3A). Oligosaccharides **14**, **28** and **46**, contain an AT-binding pentasaccharide sequence, but also provide another option to access Arixtra®-like compounds with anticoagulant activity, or even ones in which GlcA-*p*NA-N_3_ has been removed using Smith or alkaline degradation methods.^26^

The 2-*O*-sulfo glucuronic acid (GlcA2S) residue represents <1–5% of total uronic acid residues in HS and heparin isolated from natural sources and only limited information on the biological role of these rare saccharide residues has been reported.17*b* The availability of pure structurally defined GlcA2S-containing oligosaccharides should promote investigations into the SAR of this rare functional group.17*b* Pentasaccharide intermediate **IV**, GlcA(1→4)GlcNS(1→4)GlcA(1→4)GlcNS (1→4)GlcA-*p*NA-N_3_, was treated with 2-OST17*d* and PAPS to introduce a GlcA2S residue (Fig. 2C). Surprisingly, two oligosaccharides containing one GlcA2S residue, **65**, or two GlcA2S residues, **66**, respectively, were obtained with good regioselectivity and their structures were confirmed by ESI-MS and NMR analyses (ESI).

Using a similar approach, all the other compounds presented in Fig. 1 were obtained. Their purity was >97% as assessed by HPLC and their structures were confirmed by ESI-MS and NMR analyses (ESI).

### A HPLC based approach for rapid access to pure HS and heparin oligosaccharides

Gel filtration chromatography relying in Bio-gel P2 columns represents an excellent way of removing salts but is inefficient for oligosaccharide purification, since reaction byproducts, often introduced through incomplete glycosylation, have molecular weights similar to the target compounds. An HPLC based approach for rapid access to pure HS and heparin oligosaccharides was developed. The elongated products of the HS backbone prior to sulfation reactions were purified using a C-18 column, while all the sulfated products of the oligosaccharides were purified using Q-Sepharose fast flow column. Purity and structural analysis of the entire oligosaccharide library (Fig. 1) was conducted (ESI†). Representative data for the analysis of a GlcNS3S6S-containing hexasaccharide, **14** GlcNS6S (1→4)GlcA(1→4)GlcNS3S6S(1→4)IdoA2S(1→4)GlcNS6S(1→4)GlcA-*p*NA-N_3_, is presented in Fig. 3. Hexasaccharide **14** eluted as a single peak from high-resolution anion exchange HPLC column, indicating that this compound was pure (97% purity, Fig. 3B). The molecular mass of hexasaccharide **14** was determined to be 1901.1 ± 0.8 by electrospray ionization mass spectrometry (ESI-MS), identical to the calculated molecular mass of 1900.6. During chemoenzymatic synthesis the strong UV absorbance of the *p*NA-N_3_ aglycone facilitates the detection and purification of the synthetic intermediates.

### NMR characterization and study

**Fig. 4:**
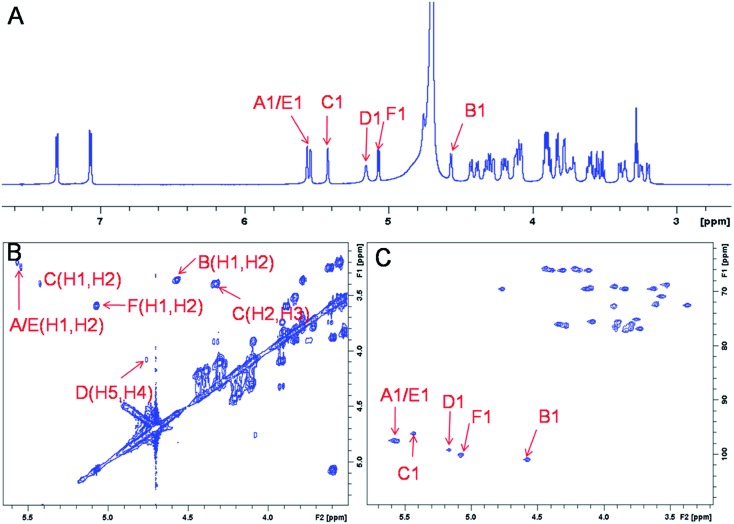
NMR characterization of hexasaccharide **14** (structure see Fig. 3A). Panel A, B, C show the 1D ^1^H NMR, 2D ^1^H–^1^H COSY and ^1^H–^13^C HSQC spectra, respectively. Peaks corresponding to the anomeric protons of these compounds can be clearly identified.

The representative characterization of oligosaccharide **14** by NMR spectroscopy is presented in Fig. 4. In the ^1^H NMR spectrum, six anomeric proton signals are assigned confirming that this compound is a hexasaccharide (Fig. 4A and 3A). The signals corresponding to the anomeric protons of the three GlcNS residues (residues **A**, **C** and **E**) resonate from 5.4 to 5.6 ppm and the signals at 4.56 and 5.07 ppm correspond to GlcA residues **B** and **F**. The signal at 5.07 ppm of residue F-1 is due to the de-shielding effect of the nearby aromatic ring. The ^3^*J*_HH_ coupling constants of A-1, C-1 and E-1 were 4 Hz while B-1 and F-1 were 8 Hz, suggesting α-linkages and β-linkages, respectively. Interestingly, when conducting NMR analysis on our oligosaccharide library, we discovered that the anomeric proton of IdoA2S residues usually appeared as broad peaks in the ^1^H NMR spectra, and correlation signals in 2D spectra were often of very low intensity. For example, the anomeric proton of the IdoA2S residue (D-1) of hexasaccharide **14** showed a broad peak at 5.18 ppm and the (D-1, D-2) cross-peak was so weak that it was not observed in the ^1^H–^1^H COSY spectrum. However, a relatively weak correlation in the IdoA2S residue between D-5 and D-4 was detected (4.76, 4.08). The broad anomeric signal for the IdoA2S observed in the ^1^H NMR spectrum is attributed to the inter-conversion of the multiple conformational forms of the IdoA2S pyranose ring **D**, ^1^C_4_-chair, ^2^S_0_-skew boat and ^4^C_1_-chair. The addition of ethylenediaminetetraacetic acid (EDTA) significantly sharpened the IdoA2S signals and often resulted in significant chemical shift variations, possibly due to the chelation of trace amounts of divalent cations.17*c*

The proton signals from the saccharide residues in **14** are highly overlapped, requiring that we conduct 2D NMR experiments, including, ^1^H–^1^H TOCSY, NOESY and ^1^H–^13^C HSQC, to fully assign chemical shifts (Fig. 4C and ESI†). By combining TOCSY and COSY experiments, we were able to identify the proton signals of A-3 and A-4 were 3.56 and 3.51 ppm, respectively, which suggests that residue **A** is at the non-reducing end of the carbohydrate chain. The NOESY signals between F-1, F-5 protons and aromatic aglycone demonstrated that residue **F** was located at the reducing end of the chain (ESI†). The cross peak (3.39, 4.33) in COSY spectrum is corresponding to the correlation between H-2 and H-3 from residue **C**, supporting the presence of its important 3-*O*-sulfo group.

Since anomeric signals in an oligosaccharide are unique and are critical for making NMR assignments, a summary of the anomeric proton and carbon chemical shifts for various glucosamine and uronic acid residues, based on the NMR analysis of our library is presented in Table 1. It was not surprising that the anomeric chemical shift was relatively sensitive to the flanking residue on its reducing end, presumably due to the steric interactions. In contrast, the neighboring residue that is on the non-reducing end has a minimal impact on chemical shift values. These observations inspired us to summarize the anomeric chemical shift variation according to the neighboring residue on the reducing end.

**Table 1:** Anomeric ^1^H and ^13^C chemical shifts (*δ*) of glucosamine and uronic acid residues in D_2_O at 298 K

	^1^H, *δ* (ppm)		^13^C, *δ* (ppm)	
Glucosamine	GlcA^*a*^	IdoA(±2S)^*a*^	GlcA^*a*^	IdoA(±2S)^*a*^
GlcNS	5.52(±0.04)	5.24(±0.02)	97.0(±0.2)	97.3(±0.2)
GlcNS6S	5.52(±0.04)	5.33(±0.02)	97.1(±0.2)	96.2(±0.2)
GlcNS3S6S	—^*b*^	5.43(±0.04)	—^*b*^	96.0(±0.2)
GlcNAc	5.32(±0.03)	—^*b*^	96.8(±0.3)	—^*b*^
GlcNAc6S	5.33(±0.02)	—^*b*^	96.7(±0.3)	—^*b*^

Uronic acid	GlcNX^*c*^	GlcNX6S^*c*^	GlcNX^*c*^	GlcNX6S^*c*^
GlcA	4.43(±0.03)	4.54(±0.03)	102.0(±0.2)	101.5(±0.04)
GlcA2S	4.65	—^*b*^	100.1	—^*b*^
IdoA	4.95(±0.01)	4.95(±0.02)	101.5(±0.1)	101.6(±0.2)
IdoA2S	5.18(±0.02)	5.18(±0.02)	99.0(±0.1)	99.0(±0.1)

^*a*^Uronic residues are adjacent to the reducing end of glucosamine residues.

^*b*^Not present.

^*c*^X = SO_3_ or Ac; glucosamine residues are adjacent to the reducing end of uronic acid residues.

The shift of the anomeric ^1^H/^13^C is 5.52/97.0 ppm for GlcNS and 5.52/97.1 ppm for GlcNS6S, in the presence of a GlcA residue attached to its reducing end. The anomeric shift values are relatively unaffected by the presence of a 6-*O*-sulfo group. In contrast, when an IdoA (±2S) residue is on the reducing end of a hexosamine residue, both proton and carbon anomeric signals for GlcNS and GlcNS6S are dramatically shifted to 5.24/97.3 and 5.33/96.2 ppm, respectively. Similarly, the anomeric signal of GlcNAc and GlcNAc6S have a comparable chemical shift of 5.32/96.8 ppm if a GlcA residue is present on the reducing end side of this residue. The shift of the H-1 signal is very similar for GlcNAc(6S) and GlcNS6S, only a COSY spectrum could easily distinguish these due to their large differences in the shift values of their H-2 signals. The 3-*O*-sulfo group also exhibits an effect on GlcNS3S6S with an apparent downfield shift signal to ∼5.43 ppm, compared to the GlcNS and GlcNS6S residues. This observation is helpful in identifying the →4)GlcNS3S6S(1→4)IdoA2S(1→ disaccharide unit, which is an important component of the AT-binding site.

Uronic acid residues show characteristic anomeric ^1^H/^13^C shifts for GlcA of 4.43/102.0 ppm and GlcA2S of 4.65/100.1 ppm, respectively, in the presence of a neighboring GlcNX (X = Ac or SO_3_H) on its reducing end. The proton chemical shift for GlcA notably shifts to 4.54 ppm when the reducing end GlcNX residue carries a 6-*O*-sulfo group. It is noteworthy that the 6-*O*-sulfo group has a greater impact than *N*-sulfo group of the neighboring glucosamine residue on the reducing end. The anomeric position of the IdoA2S residue exhibits consistent chemical shifts of 5.18/99.0 ppm independent of whether it has a GlcNX or GlcNX6S at its reducing end. The anomeric position of the IdoA residue has very different chemical shifts 4.95/101.5 ppm.

## Discussion

Heparin is a widely used clinical anticoagulant for the prevention and treatment of arterial and venous thrombosis.^7^ Due to the vulnerability of animal-sourced heparin towards the presence of impurities and contamination, developing a method to prepare heparin from non-animal sources represents a safer approach to the development of anticoagulant and antithrombotic therapeutics.^24^ New synthetic versions of heparin might also make it possible to develop non-anticoagulant heparins that could be exploited as antiviral and anticancer agents.^27^ As our previous studies have already demonstrated, chemoenzymatically synthesized oligosaccharides exhibit excellent anticoagulant activity and improved pharmacological properties.24c,e The control the placement of uronic acid epimers and sulfo groups are essential for obtaining heparin and HS chains having specific biological functions. The diverse HS and heparin oligosaccharide library prepared in the current study serve as a valuable set of tools to perform SAR studies and also might contain potential drug candidates.

Introducing chemically active functional groups is expected to expand the use of the library. In the current library, each oligosaccharide possesses an azido group at the reducing end. The azide functionality is well known to be inert within biological systems but can be readily tagged covalently with imaging probes or modified using an azide-specific reaction, such as Staudinger ligation with phosphines and the [3 + 2] cycloaddition with alkynes.^28^ The library of oligosaccharides synthesized in the current study are azide-functionalized, which permits their detection or selective capture in cells or in living organisms.^29^ Moreover, azide can be reduced to a free amino group, which would be subsequently biotinylated using carbodiimide chemistry or through reductive amination.^30^ The oligosaccharide library can be coupled to the surface of a microarray for high-throughput screening of heparin-binding proteins.^31^

Although sulfation at the 3-OH position of glucosamine occurs infrequently in HS, this sulfation pattern is closely related to its biological functions, facilitating the entry of herpes simplex virus into host cells to establish infection,^32^ regulating axon guidance and growth of neurons,^33^ controlling follicular properties in ovulation,^34^ and enabling HS and heparin to bind to AT promoting blood anticoagulation.^35^ It is still not entirely clear how the 3-*O*-sulfoglucosamine (GlcNS3S±6S) effects these important roles in contributing to HS biological activity. The ready availability of multi-milligram quantities of oligosaccharide structural variants containing a GlcNS3S6S residue should facilitate further biological investigation.

Due to the low abundance of 2-*O*-sulfoglucuronic acid (GlcA2S) in heparin and HS very little research has been focused on the SAR of this residue. Moreover, while 2-OST has been shown to act, albeit poorly, on GlcA in a polysaccharide substrate17*d* it had not been shown to act on oligosaccharide substrates, nor had the regioselectivity of this enzyme been established. IdoA is another less frequently occurring residue that has been difficult to prepare due to the reversible C5-epimerization. In this study, we successfully synthesized and fully characterized a series of GlcA2S- or IdoA-containing oligosaccharides. Ever since the 2007 heparin contamination heparin crisis, NMR spectroscopy has played an increasingly important role in heparin drug analysis. A library of oligosaccharides, including many possible and often rare structures is in demand but the availability of various oligosaccharides is still a roadblock for such studies. This is the first report of a library covering diverse heparin and HS oligosaccharides and a systematic NMR study that should pave the way for improving our understanding of the SAR of HS and heparin.

## Conclusions

We have successfully developed a chemoenzymatic approach for the efficient preparation of **66** structurally defined HS and heparin oligosaccharides, including “rare” and critical moieties, such as GlcA2S, IdoA, GlcNS3S6S and AT-binding pentasaccharide sequence. The ultraviolet detectable tag and an HPLC-based purification method facilitate the rapid access to a diverse library containing tens of milligrams of high purity HS and heparin oligosaccharides. These oligosaccharides are not only potentially important for bioactivity evaluation but also good substrates to construct glycoconjugates or microarrays through azide chemistry. This work also describes a systematic NMR study on these HS and heparin oligosaccharides, paving the way to more complicated HS and heparin polysaccharide characterization and helping to safeguard the quality of the critically important drug, heparin.

## Author contribution

R. J. L. and J. L. designed experiments, analyzed data and wrote the manuscript. X. Z. and V. P., H. M. J., A. M. L. and A. M. P. G. performed all the synthesis and purification of oligosaccharides described within this manuscript. T. Q. P. prepared the enzymes and cofactors for the synthesis.

## Conflicts of interest

J. L. is a founder of Glycan Therapeutics. V. P., H. M. J., A. M. L. and A. M. P. G. are employees of Glycan Therapeutics. X. Z., T. Q. P. and R. J. L. declare no competing financial interests.

## Supplementary Material

Supplementary information

## References

[cit1] Poulain F. E., Yost H. J. (2015). Development.

[cit2] Fu L., Suflita M., Linhardt R. J. (2016). Adv. Drug Delivery Rev..

[cit3] Oduah E. I., Linhardt R. J., Sharfstein S. T. (2016). Pharmaceuticals.

[cit4] Linhardt R. J. (2003). J. Med. Chem..

[cit5] Griffin C. C., Linhardt R. J., Van Gorp C. L., Toida T., Hileman R. E., Schubert II R. L., Brown S. E. (1995). Carbohydr. Res..

[cit6] Chen Y., Lin L., Agyekum I., Zhang X., St Ange K., Yu Y., Zhang F., Liu J., Amster I. J., Linhardt R. J. (2017). J. Pharm. Sci..

[cit7] Onishi A., St Ange K., Dordick J. S., Linhardt R. J. (2016). Front. Biosci..

[cit8] Bhaskar U., Sterner E., Hickey A. M., Onishi A., Zhang F., Dordick J. S., Linhardt R. J. (2012). Appl. Microbiol. Biotechnol..

[cit9] Guerrini M., Beccati D., Shriver Z., Naggi A., Viswanathan K., Bisio A., Capila I., Lansing J. C., Guglieri S., Fraser B., Al-Hakim A., Gunay N. S., Zhang Z., Robinson L., Buhse L., Nasr M., Woodcock J., Langer R., Venkataraman G., Linhardt R. J., Casu B., Torri G., Sasisekharan R. (2008). Nat. Biotechnol..

[cit10] Keire D., Mulloy B., Chase C., Al-Hakim A., Cairatti D., Gray E., Hogwood J., Morris T., Mourão P., Soares M. D. C., Szajek A. (2015). BioPharm Int..

[cit11] Liu J., Linhardt R. J. (2014). Nat. Prod. Rep..

[cit12] Szajek A. Y., Chess E., Johansen K., Gratzl G., Gray E., Keire D., Linhardt R. J., Liu J., Morris T., Mulloy B., Nasr M., Shriver Z., Torralba P., Viskov C., Williams R., Woodcock J., Workman W., Al-Hakim A. (2016). Nat. Biotechnol..

[cit13] Zhang Z., Li B., Suwan J., Zhang F., Wang Z., Liu H., Mulloy B., Linhardt R. J. (2009). J. Pharm. Sci..

[cit14] Rudd T. R., Gaudesi D., Lima M. A., Skidmore M. A., Mulloy B., Torri G., Nader H. B., Guerrini M., Yates E. A. (2011). Analyst.

[cit15] Pervin A., Gallo C., Jandik K. A., Han X. J., Linhardt R. J. (1995). Glycobiology.

[cit16] Casu B., Guerrini M., Naggi A., Torri G., De-Ambrosi L., Boveri G., Gonella S., Cedro A., Ferró L., Lanzarotti E., Paterno M., Attolini M., Valle M. G. (1996). Arzneimittelforschung.

[cit17] Wang Z., Hsieh P., Xu Y., Thieker D., Chai E. J. E., Xie S., Cooley B., Woods R. J., Chi L., Liu J. (2017). J. Am. Chem. Soc..

[cit18] Yin J., Seeberger P. H. (2010). Methods Enzymol..

[cit19] Yates E. A., Guimond S. E., Turnbull J. E. (2004). J. Med. Chem..

[cit20] Lucas R., Angulo J., Nieto P. M., Martín-Lomas M. (2003). Org. Biomol. Chem..

[cit21] (b) Petitou M., Jacquinet J. C., Choay J., Lormeau J. C. and Nassr M., U.S. Pat., 4,818,816, 1989.

[cit22] DeAngelis P. L., Liu J., Linhardt R. J. (2013). Glycobiology.

[cit23] Farrán A., Cai C., Sandoval M., Xu Y., Liu J., Hernáiz M. J., Linhardt R. J. (2015). Chem. Rev..

[cit24] Chen Y., Li Y., Yu H., Sugiarto G., Thon V., Hwang J., Ding L., Hie L., Chen X. (2013). Angew. Chem., Int. Ed..

[cit25] Sheng J., Xu Y., Dulaney S. B., Huang X., Liu J. (2012). J. Biol. Chem..

[cit26] Zhang X., Xu Y., Hsieh P. H., Liu J., Lin L., Schmidt E. P., Linhardt R. J. (2017). Org. Biomol. Chem..

[cit27] Hwang H. H., Lee D. Y. (2016). Macromol. Res..

[cit28] Borrmann A., van Hest J. C. M. (2014). Chem. Sci..

[cit29] Li S., Wang L., Yu F., Zhu Z., Shobaki D., Chen H., Wang W., Wang J., Qin G., Erasquin U. J., Ren L., Wang Y., Cai C. (2017). Chem. Sci..

[cit30] Zhao J., Liu X., Malhotra A., Li Q., Zhang F., Linhardt R. J. (2017). Anal. Biochem..

[cit31] Yang J., Hsieh P. H., Liu X., Zhou W., Zhang X., Zhao J., Xu Y., Zhang F., Linhardt R. J., Liu J. (2017). Chem. Commun..

[cit32] Shukla D., Liu J., Blaiklock P., Shworak N. W., Bai X., Esko J. D., Cohen G. H., Eisenberg R. J., Rosenberg R. D., Spear P. G. (1999). Cell.

[cit33] Thacker B. E., Seamen E., Lawrence R., Parker M. W., Xu Y., Liu J., Vander Kooi C. W., Esko J. D. (2016). ACS Chem. Biol..

[cit34] de Agostini A. I., Dong J. C., de Vantéry Arrighi C., Ramus M. A., Dentand-Quadri I., Thalmann S., Ventura P., Ibecheole V., Monge F., Fischer A. M., HajMohammadi S., Shworak N. W., Zhang L., Zhang Z., Linhardt R. J. (2008). J. Biol. Chem..

[cit35] Lindahl U., Backstrom G., Thunberg L., Leder I. G. (1980). Proc. Natl. Acad. Sci. U. S. A..

