# Supplementary material for: Chemoenzymatic synthesis of heparan sulfate and heparin oligosaccharides and NMR analysis: paving the way to a diverse library for glycobiologists
Source: Chem Sci. 2017 Sep 21;8(12):7932–40. doi: 10.1039/c7sc03541a (PMC5849142; doi:10.1039/c7sc03541a)
Supplement: Supplementary file 1 [file SC-008-C7SC03541A-s001.pdf]

Electronic Supplementary Material (ESI) for Chemical Science.
This journal is © The Royal Society of Chemistry 2017

Chemoenzymatic synthesis of heparan sulfate and heparin oligosaccharides and NMR analysis: Paving the way to a diverse library for glycobiologists

Xing Zhang,‡[a] Vijayakanth Pagadala,‡[b] Hannah M. Jester[b], Andrew M. Lim[b], Truong Quang Pham[c], Anna Marie Panagiotis Goulas[b], Jian Liu,*[c] Robert J. Linhardt*[a]

[a] Department of Chemistry and Chemical Biology, Rensselaer Polytechnic Institute, Troy, New York 12180, USA

[b] Glycan Therapeutics, LLC, Chapel Hill, North Carolina 27599.

[C] Division of Chemical Biology and Medicinal Chemistry, Eshelman School of Pharmacy, University of North Carolina, Chapel Hill, North Carolina 27599

‡ Contributed equally to this work.

Prepared for *Chemical Science*, June 2017

To whom all correspondence should be addressed at linhar@rpi.edu and liuj@email.unc.edu

Supporting Information

Table of Contents

I. Expression of HS biosynthetic enzymes S3

II. General methods for chemoenzymatic synthesis S4

III. Synthetic schemes for oligosaccharides S6

IV. General methods for HPLC analysis and purification S8

V. General methods for mass spectrum analysis S9

VI. General methods for NMR analysis S9

VII. HPLC profiles, MS and NMR data S11

I. Expression of HS biosynthetic enzymes

Seven enzymes were used for the synthesis of the heparan sulfate oligosaccharides. The enzymes NST, 6-OST-1, 6-OST-3, 3-OST-1 and *Pasteurella multocida* heparosan synthase 2 (pmHS2) were expressed in *E. coli* (Renpeng Liu, Yongmei Xu, Miao Chen, Michel Weïwer, Xianxuan Zhou, Arlene S. Bridges, Paul L. DeAngelis, Qisheng Zhang, Robert J. Linhardt and Jian Liu. *J. Biol. Chem.* 2010, **285**, 34240-34249.). C_5-epi and 2-OST, were expressed in sf-9 insect cells and purified by chromatography as described previously (K. Balagurunathan, M. Z. Lech, D. L. Beeler, Z. L. Wu, R. D. Rosenberg, *Nat. Biotechnol.,* 2003*,* **21**, 1343-1346.).

All the bacterial recombinant enzymes were purified using Ni-Sepharose resin (GE Healthcare). Briefly the recombinant *E. coli* cells were pelleted at 9000 × g and resuspended in lysis buffer containing 50 mM Tris pH 7.0, 500 mM NaCl, 30 mM imidazole (Buffer A). The cell suspension is lysed by sonication and the protein containing supernatant is collected after centrifugation at 16000 × g for 30 min. The supernatant is loaded on Ni-Sepharose column and washed with 5 column volumes of buffer A then eluted with Buffer A containing 300 mM imidazole. The enzyme rich fractions were polled and 10% glycerol was added and stored in -80°C until further use.

The 2-OST and C_5-epi enzymes were purified from the insect cell culture supernatants using Toyopearl AF-heparin HC 650 M resin (Tosoh Biosciences). Enzymes were eluted using a linear gradient of NaCl from 100 mM to 1000 mM in 50 mM MOPS pH 7.0 buffer, 3% glycerol. The 2-OST activity rich fractions eluted at 750 mM NaCl, whereas C_5-epi eluted at 500 mM NaCl. Final concentration of 10% glycerol was added to the purified enzymes and the preparation was frozen at –80 °C until further use.

II. General methods for chemoenzymatic synthesis

The synthesis scheme involved two broad steps, elongation and modification. The sequence and combination of the elongation and modification steps determines the structure of the final oligosaccharide. Each elongation step results in an increase in the size of oligomer by one sugar residue. Modification step involves site-specific sulfation on a particular sugar residue or installation of IdoA residues.

Nine different enzymatic reaction steps are involved in the overall synthesis including, Step 1 (elongation to add GlcNTFA), Step 2 (elongation to add GlcA), Step 3 (elongation to add GlcNAc), Step 4 (detrifluoroacetylation/*N*-sulfation), step 5 (epimerization), Step 6 (2-*O*-sulfation), Step 7 (one step epimerization/2-O-sulfation), step 8 (6-*O*-sulfation), step 9 (3-*O*-sulfation by 3-OST-1).

Step 1. Elongation of oligosaccharide backbone by addition of GlcNTFA residue. For instance, GlcA-*p*NA-N_3 (3.2 mM) monomer was dissolved in buffer containing Tris (25 mM, pH 7.2), $MnCl_2$ (5 mM), pmHS2 (60 µg/ml) and UDP-GlcNTFA (4.5 mM), then incubated at 30°C overnight. Addition of GlcNTFA residue results in reduction in binding affinity to the PAMN column and hence quicker elution time.

Step 2. Elongation of oligosaccharide backbone by addition of a GlcA residue. For instance, a disaccharide (3.2 mM) was dissolved in a buffer containing Tris (25 mM, pH 7.2), $MnCl_2$ (5 mM), pmHS2 (60 µg/ml) and UDP-GlcA (4.5 mM), then incubated at 30°C overnight. Addition of GlcA residue results in an increase in binding affinity to PAMN column and hence delayed elution time.

Step 3. Elongation of oligosaccharide backbone by addition of GlcNAc residue. Briefly, GlcA-*p*NA-N_3 (J. Yang, P. H. Hsieh, X. Liu, W. Zhou, X. Zhang, J. Zhao, Y. Xu, F. Zhang, R. J. Linhardt and J. Liu, *Chem. Commun.*, 2017, **53**, 1743-1746.) 3.2 mM was dissolved in buffer containing Tris (25 mM, pH 7.2),

$MnCl_2$ (5 mM), pmHS2 (60 μg/ml) and UDP-GlcNAc (4.5 mM), then incubated at 30°C overnight. For the steps 1 and 3 the starting material is either monomer GlcA-*p*NA-N_3 or any of the backbones containing odd numbered sugars- 3 mer, 5 mer, or 7 mer. For the step 2 the starting material is even numbered sugar backbones 2 mer, 4 mer, 6 mer and 8 mer. All the unsulfated elongation intermediates were purified on C-18 Column (Biotage) equilibrated to pH 2.0 using 0.2% TFA in water and eluted with 70% methanol, 0.2% TFA in water.

Step 4. Conversion of a GlcNTFA residue to a GlcNS residue involves detrifluoroacetylation and *N*-sulfation. The detrifluoroacetylation of oligosaccharide was acheived by addition of 0.1 M LiOH and maintaining a pH of >12.0 at room temperature for 10 minutes. The product formation was monitored by electrospray ionizationmass spectrometry (ESI-MS). The pH of the product was immediately adjusted to 7.0 using hydrochloric acid (1 M). The detrifluoroacetylated compound was *N*-sulfated in a solution containing 2-(*N*-morpholino) ethanesulfonic acid (MES, 50 mM) pH 7.0 and *N*-sulfotransferase (32 μg/ml) and 3'-Phosphoadenosine-5'-phosphosulfate (PAPS, 1.5 mole equiv. of free amino groups) at 37°C reaction overnight.

Step 5. Epimerization of GlcA residue to an IdoA residue was completed using C_5-epi. Oligosaccharide (1.2 mM) was incubated in a solution containing Tris (25 mM) buffer (pH 7.5) and C_5-epi (3 μg/ml), at 37°C ovenight. As the epimerization is a simple reversible reaction, only 30%-40% of the GlcA is converted to IdoA before it reached equilibrium. The IdoA containing compound migrates faster on the silica based polyamine (PAMN)-HPLC analysis and was separated.

Step 6. 2-*O*-sulfation. Conversion of GlcA to GlcA2S was completed using 2-OST enzyme. Oligosaccharide (1.2 mM) containing the GlcA was treated with 2-OST enzyme and 1.5X molar excess of PAPS in MOPS buffer overnight at 37 °C, 2 mM $MnCl_2$.

Step 7. Combined epimerization and 2-*O*-sulfation reaction to generate

IdoA2S residues. Oligosaccharide containing the GlcA was treated simultaneously with epimerase and 2-OST enzymes in presence of 1.5X molar excess of PAPS in MOPS buffer overnight at 37 ^{o}C, 2 mM $MnCl_2$. This results in nearly complete conversion (>90%) of GlcA to IdoA2S with a significant delay in the elution time of the compound corresponding to the addition of a sulfate group.

Step 8. 6-*O*-sulfation. 6-*O*-sulfotrasnferase isoforms 1 and 3 (6-OST-1 and 6-OST-3) were used. In this step, the oligosaccharide intermediate (0.5 mM) was incubated in a buffer containing MES (50 mM, pH 7.0), 6-OST-1 (50 µg/ml), 6-OST-3 (50 µg/ml) and PAPS (1.5 mole equiv. of 6-hydroxyl groups) at 37 °C overnight. 6-*O*-sulfation results in significant delay in the elution time on HPLC.

Step 9. 3-*O*-sulfation by 3-OST-1. The oligosaccharides (0.5 mM) were incubated in a solution containing MES (50 mM) buffer (pH 7.0), 3-OST-1 (20 µg/ml) and PAPS (0.675 mM) at 37 °C 2 h. 3-*O*-sulfation results in a delay in the elution time of the product.

III. Synthetic schemes for oligosaccharides

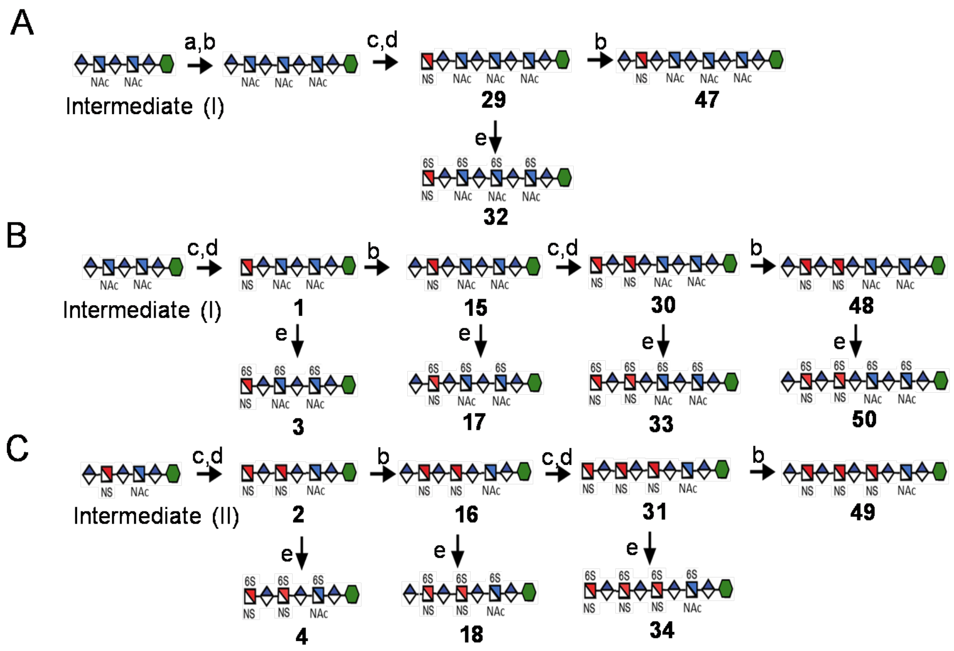

Supplementary Scheme S1. Reagents and conditions: (a) pmHS2, GlcNAc; (b) pmHS2, GlcA; (c) pmHS2, GlcNTFA; (d) Deacetylation, NST, PAPS; (e) 6-OST, PAPS.

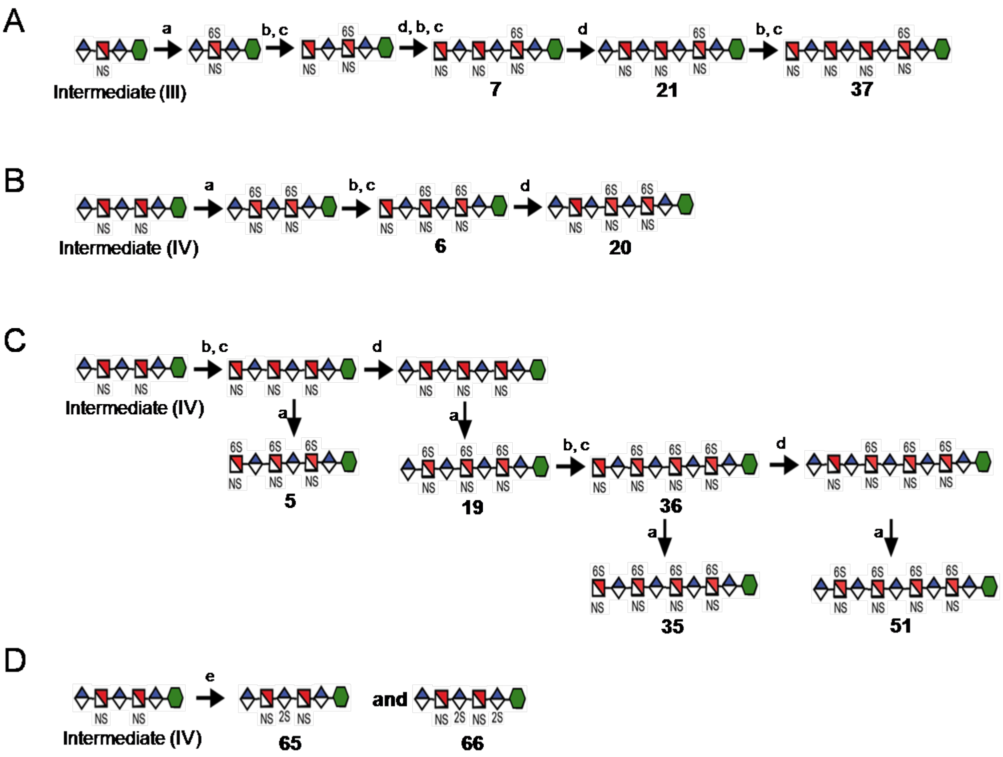

Supplementary Scheme S2. Reagents and conditions: (a) 6-OST, PAPS; (b) pmHS2, GlcNTFA; (c) Deacetylation, NST, PAPS; (d) pmHS2, GlcA; (e) 2-OST, PAPS.

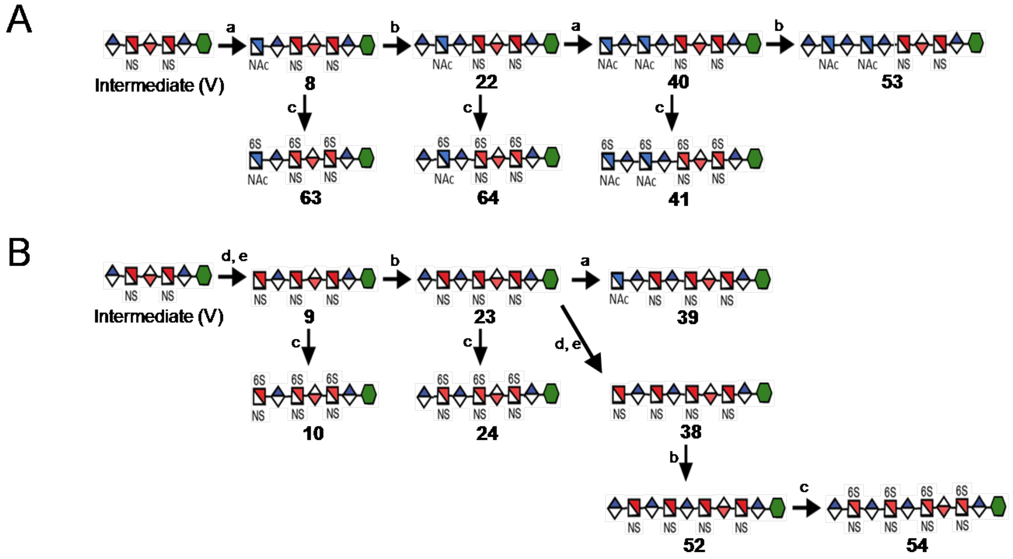

Supplementary Scheme S3. Reagents and conditions: (a) pmHS2, GlcNAc; (b) pmHS2, GlcA; (c) 6-OST, PAPS; (d) pmHS2, GlcNTFA; (e) Deacetylation, NST, PAPS.

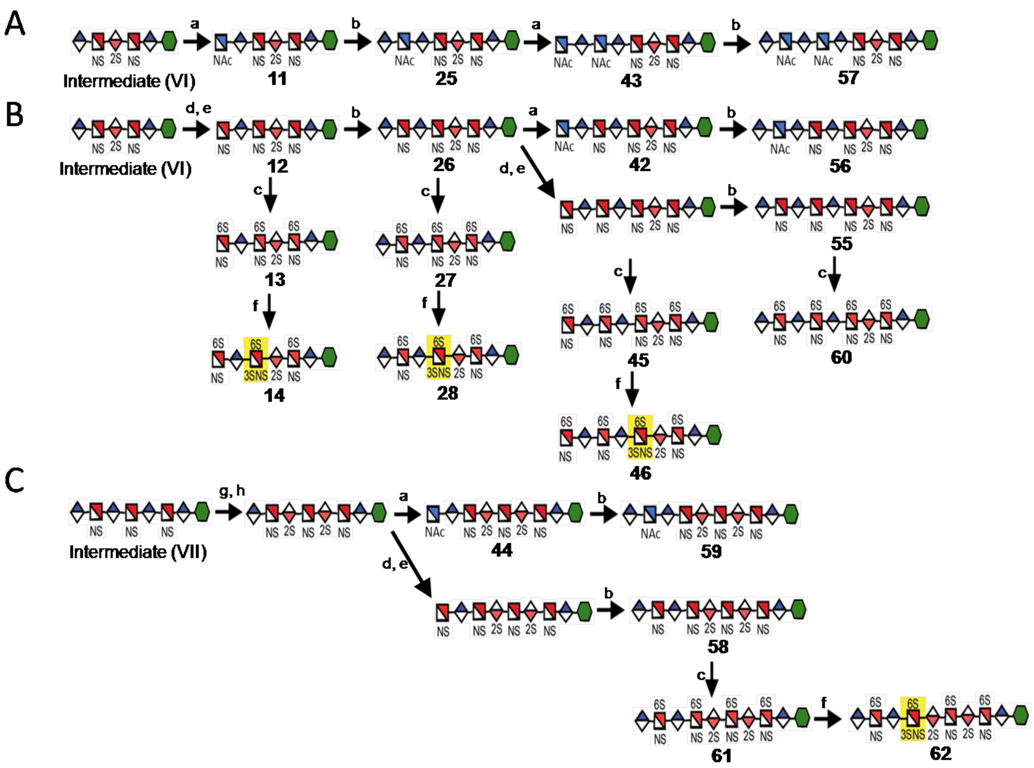

Supplementary Scheme S4. Reagents and conditions: (a) pmHS2, GlcNAc; (b) pmHS2, GlcA; (c) 6-OST, PAPS; (d) pmHS2, GlcNTFA; (e) Deacetylation, NST, PAPS; (f) 3-OST-1, PAPS; (g) C_5-epimerase; (h) 2-OST, PAPS.

IV. General methods for HPLC analysis and purification

Purification of intermediates and compounds:

The elongated products of the HS backbone prior to sulfation reactions were purified using a C-18 column. Reaction mixture was adjusted to pH 2.0 using TFA and loaded on to the C18 column equilibrated with 0.2% TFA in water (Buffer A). After washing the column with 5-column volume of buffer A, the compound was eluted using a step elution of 60% methanol in 0.2% TFA. The methanol and TFA were freeze dried in a centrivap to collect the sample for further analysis.

All the sulfated products of the oligosaccharides were purified using Q-Sepharose fast flow column (GE Healthcare), eluted with a linear gradient

0-2 M NaCl in 20 mM NaOAc-HOAc, pH 5.0.

HPLC analysis:

Both DEAE-HPLC and polyamine-based anion exchange (PAMN)-HPLC were used to analyze the purity of the products. Briefly, for DEAE-HPLC method, the column TSK gel DNA-NPR (4.6 × 75 mm, from Tosoh Bioscience) was eluted with a linear gradient of NaCl in 20 mM sodium acetate buffer (pH 5.0) from 0 to 1 M in 60 min at a flow rate of 0.4 ml min^{-1}. As for PAMN-HPLC, the column (Polyamine II-HPLC, 4.6×250 mm, from YMC) was eluted with a linear gradient of KH_2PO_4 from 0 to 1 M in 40 min then at 1 M for 30 min at a flow rate of 0.5 ml min^{-1}.

V. General methods for mass spectrum analysis

The low-resolution analyses were performed at a Thermo LCQ-Deca. Oligosaccharides were either desalted on Biogel-P2 resin or dialyzed extensively against water. The desalted or dialyzed oligosaccharides were diluted in water to achieve clear signals. A syringe pump (Harvard Apparatus) was used to introduce the sample by direct infusion (50 μl min^{-1}). Experiments were carried out in negative ionization mode with the electrospray source set to 3 KV and 150 °C. 10 mM ammonium bicarbonate was added to enhance signal when necessary. The automatic gain control was set to 1×10^7 for full scan MS. The MS data were acquired and processed using Xcalibur 1.3.

VI. General methods for NMR analysis

The NMR spectra of oligosaccharides were obtained on Bruker 600 MHz or 800 MHz standard-bore NMR spectrometer equipped with a 1H/2H/13C/15N cryoprobe with z-axis gradients. Oligosaccharide (3 mg) was dissolved in 0.4 mL of 99.9% D_2O centrifuged at 5000 x g for 1 min and lyophilized. The

process was repeated twice, and the final sample was dissolved in 0.45 mL of 99.9% D_2O. ^{1}H spectroscopy, ^{1}H-^{1}H correlated spectroscopy (COSY), ^{1}H-^{13}C heteronuclear single quantum coherence spectroscopy (HSQC), ^{1}H-^{1}H total correlation spectroscopy (TOCSY) and ^{1}H-^{1}H nuclear overhauser effect spectroscopy (NOESY) experiments were all carried out at 298 K.

VII. HPLC profiles, MS and NMR data

^{1}H NMR chemical shift assignments (in ppm) of compound **1**

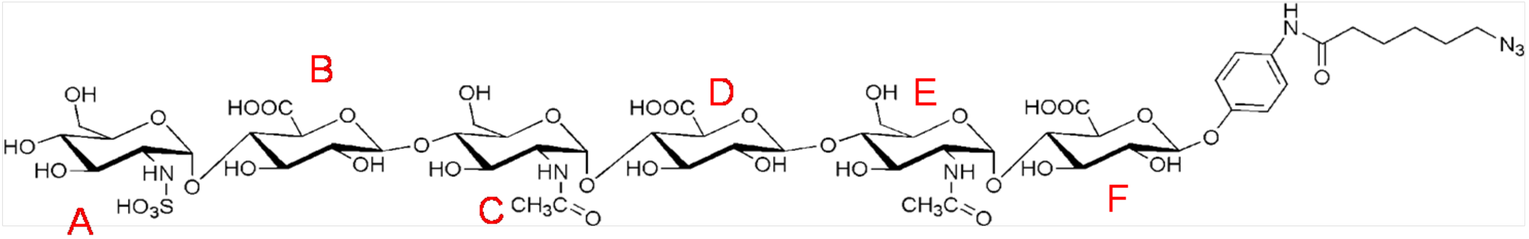

	1	2	3	4	5	6a	6b
A	5.55	3.14	3.51	3.40	3.60	3.62	3.70
B	4.44	3.31	3.77	3.73	3.82	----------	----------
C	5.29	3.79	3.72	3.77	3.80	3.61	3.83
D	4.41	3.25	3.60	3.69	3.78	----------	----------
E	5.32	3.80	3.75	3.79	3.81	3.61	3.83
F	4.99	3.49	3.70	3.75	3.85	----------	----------

Supplementary Fig. S1. ^{1}H NMR chemical shift assignments (in ppm) of compound **1**. The structure of compound **1** is shown. The protons of *p*NA-N_3 are not listed in the table.

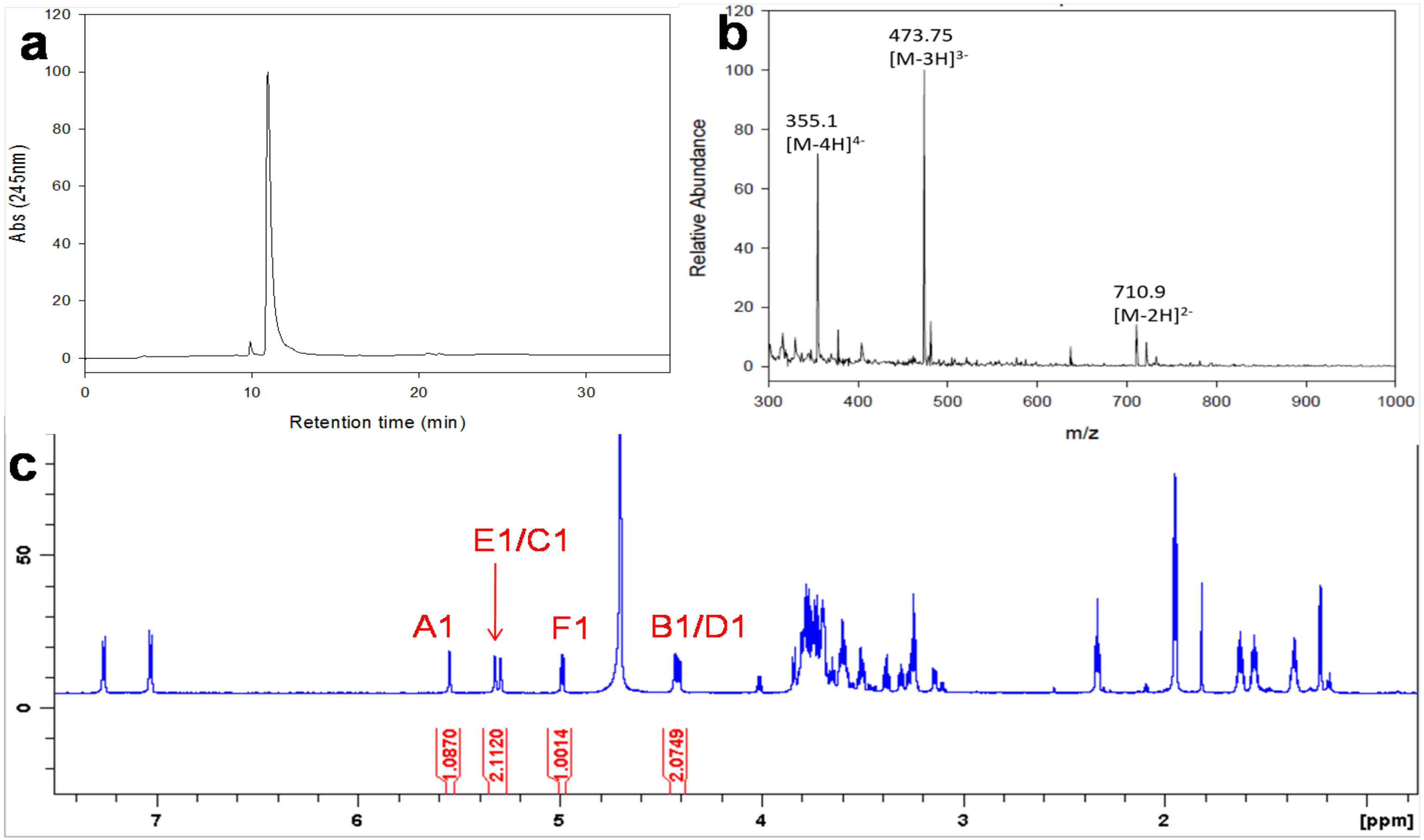

Supplementary Fig. S2. HPLC analysis (a), MS spectrum (b) and ^{1}H NMR spectrum (c) of compound **1**.

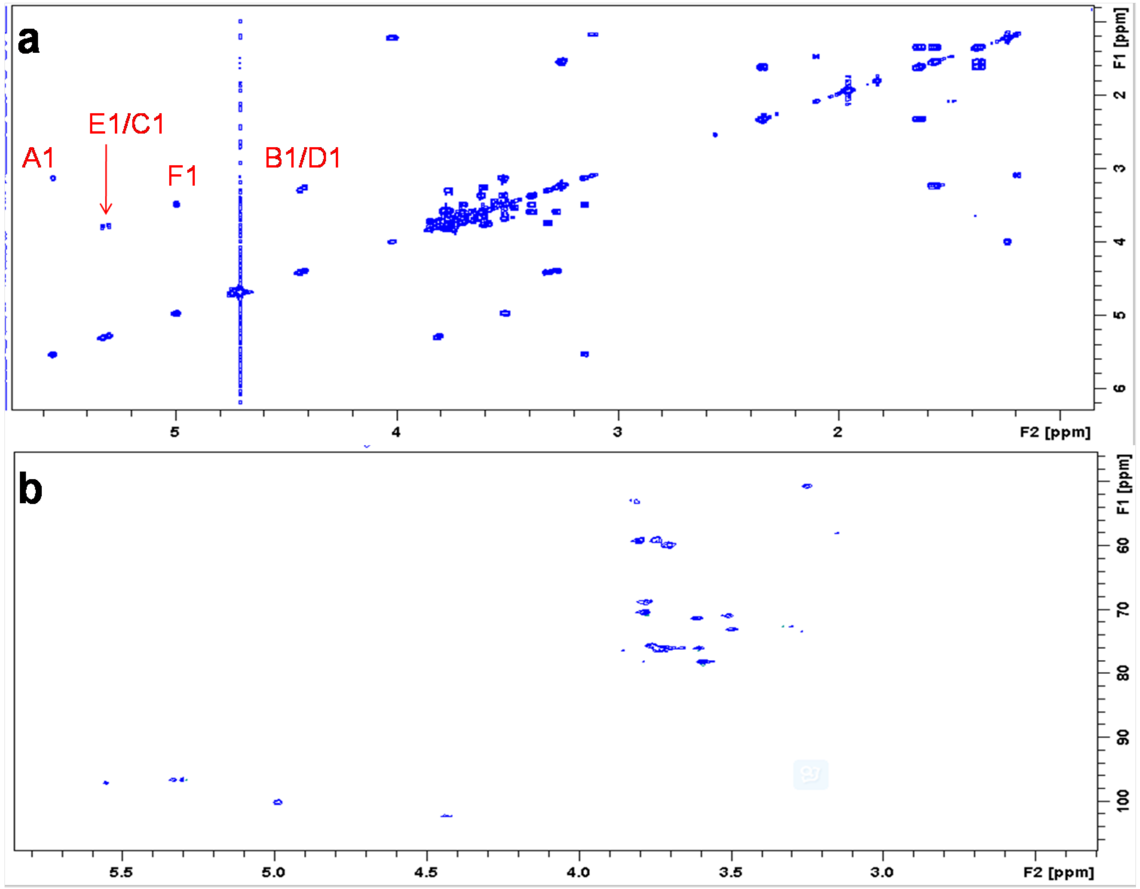

Supplementary Fig. S3. 2D NMR COSY (a) and HSQC (b) spectra of compound **1**.

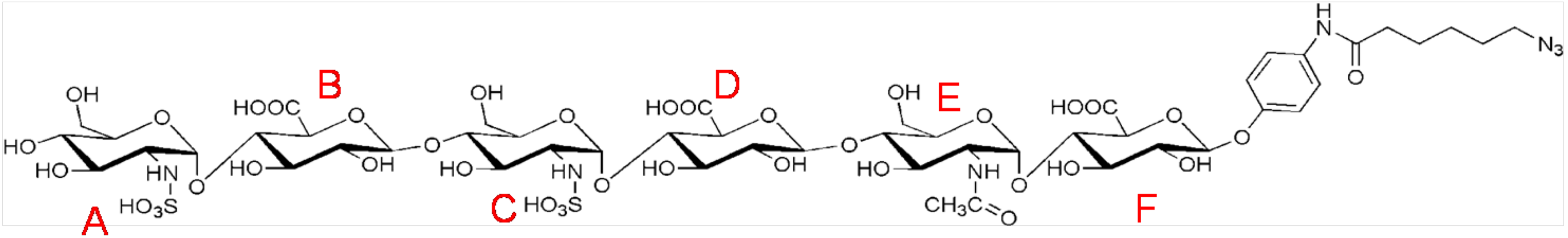

	1	2	3	4	5	6a	6b
A	5.56	3.15	3.53	3.40	3.62	3.66	3.71
B	4.46	3.33	3.76	3.73	3.75	----------	----------
C	5.53	3.20	3.61	3.67	3.75	3.75	3.80
D	4.46	3.33	3.76	3.73	3.75	----------	----------
E	5.34	3.82	3.75	3.79	3.78	3.62	3.84
F	5.01	3.51	3.73	3.77	3.86	----------	----------

Supplementary Fig. S4. ^{1}H NMR chemical shift assignments (in ppm) of compound **2**. The structure of compound **2** is shown. The protons of *p*NA-N_3 are not listed in the table.

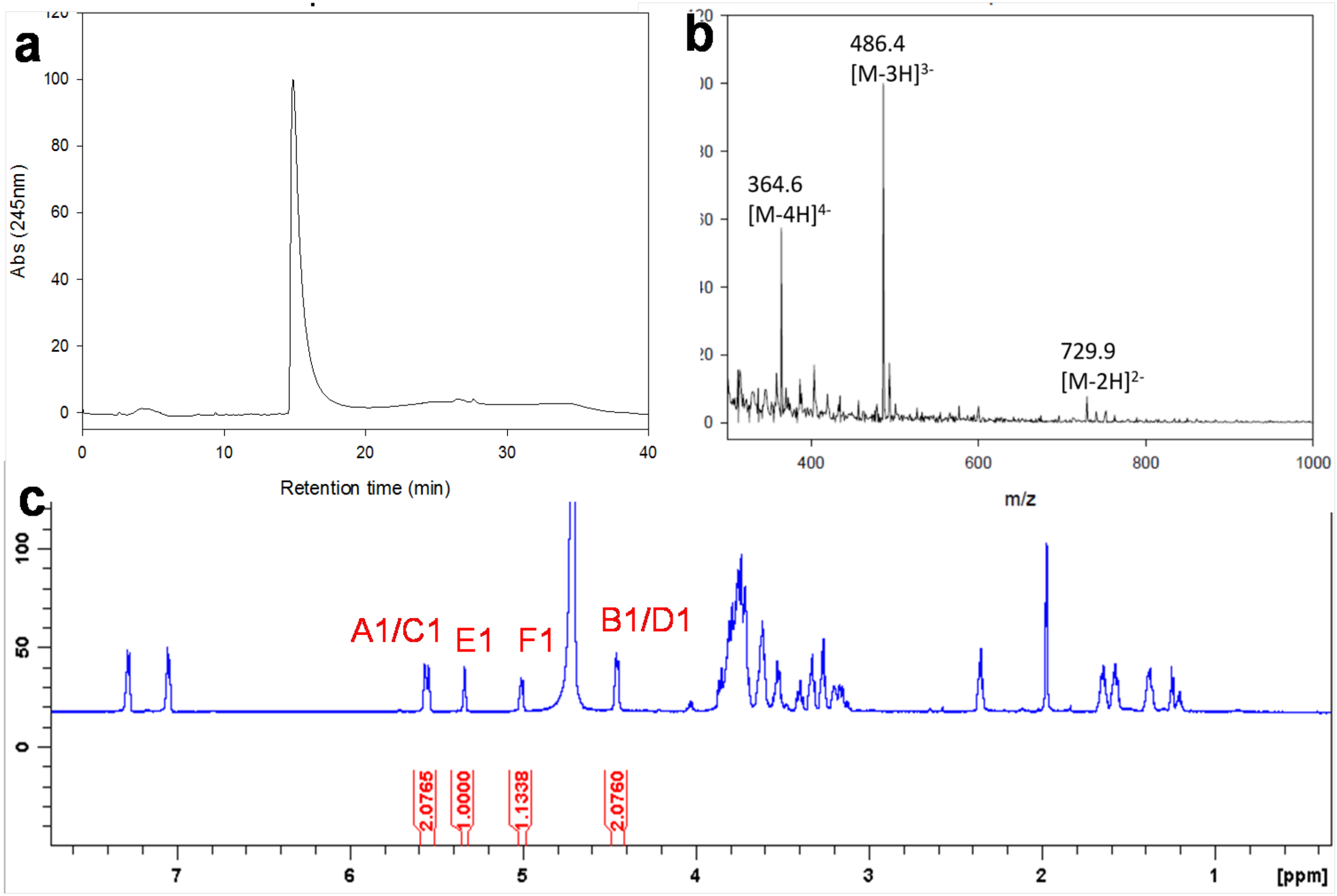

Supplementary Fig. S5. HPLC analysis (a), MS spectrum (b) and ^{1}H NMR spectrum (c) of compound **2**.

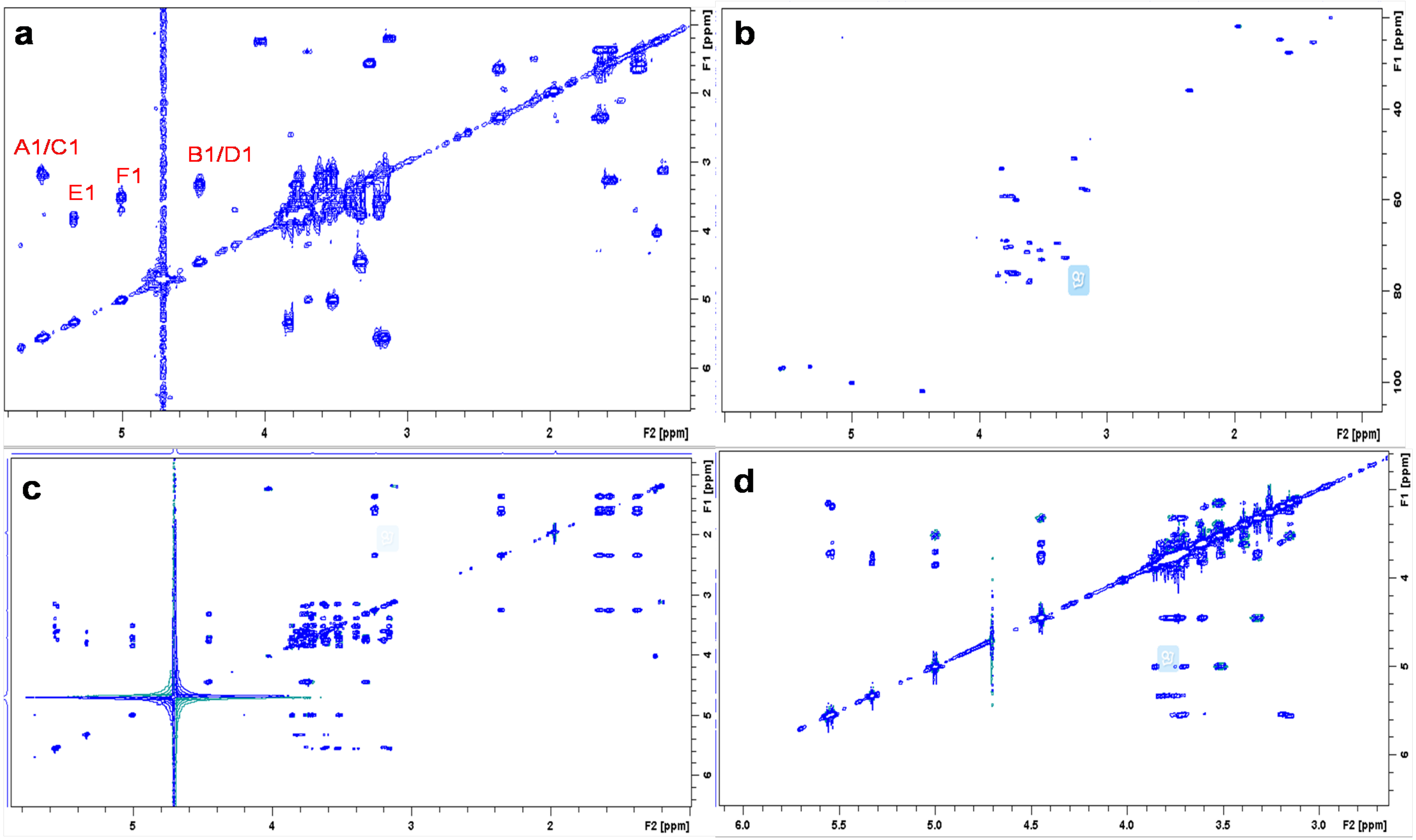

Supplementary Fig. S6. 2D NMR COSY (a), HSQC (b), TOCSY (c) and NOE (d) spectra of compound **2**.

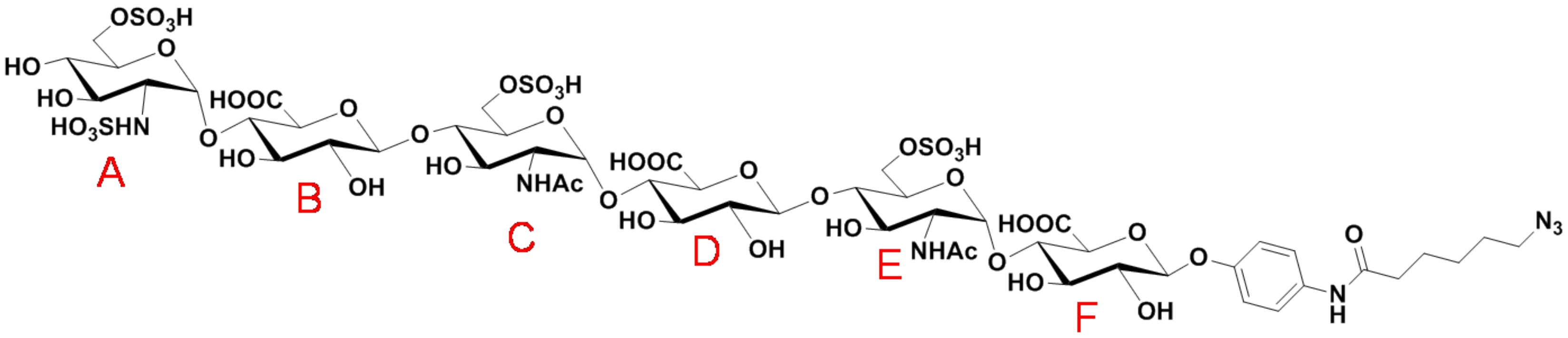

	1	2	3	4	5	6a	6b
A	5.55	3.18	3.56	3.49	3.80	4.08	4.27
B	4.52	3.30	3.78	3.68	3.74	----------	----------
C	5.33	3.85	3.77	3.66	3.97	4.11	4.37
D	4.51	3.27	3.74	3.63	3.70	----------	----------
E	5.36	3.86	3.78	3.66	3.97	4.12	4.38
F	5.01	3.52	3.71	3.77	3.86	----------	----------

Supplementary Fig. S7. ^{1}H NMR chemical shift assignments (in ppm) of compound **3**. The structure of compound **3** is shown. The protons of *p*NA-N_3 are not listed in the table.

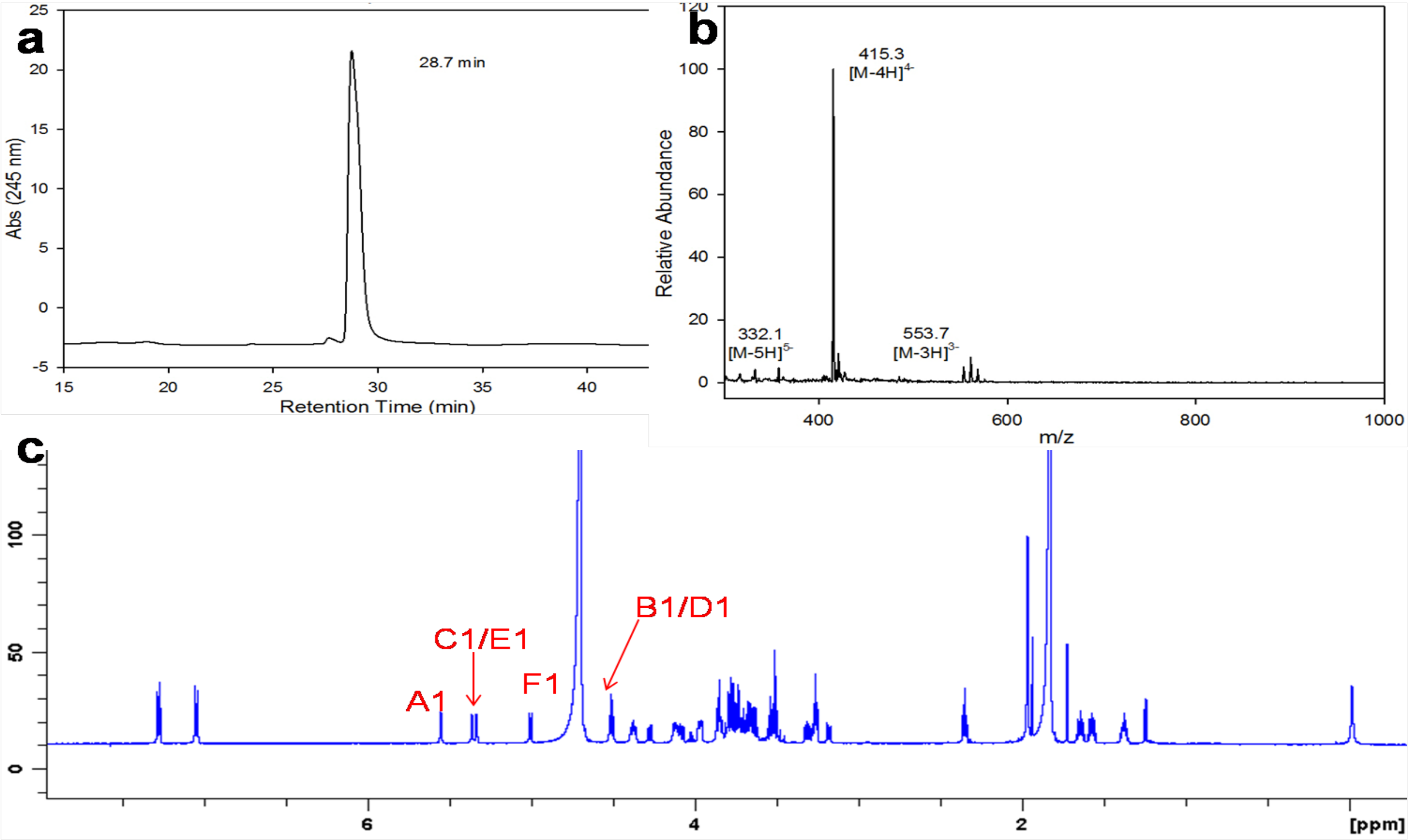

Supplementary Fig. S8. HPLC analysis (a), MS spectrum (b) and ^{1}H NMR spectrum (c) of compound **3**.

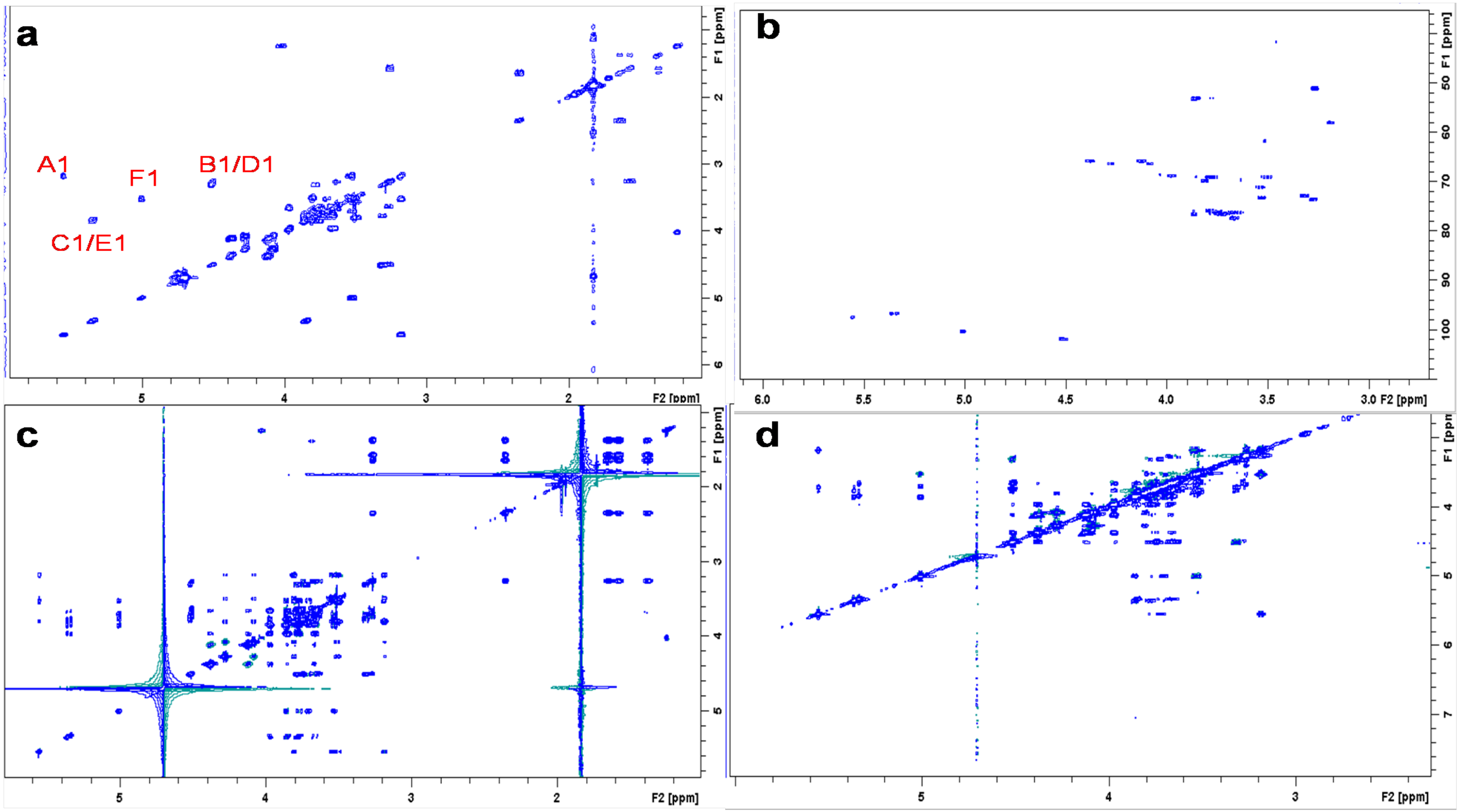

Supplementary Fig. S9. 2D NMR COSY (a), HSQC (b), TOCSY (c) and NOE (d) spectra of compound **3**.

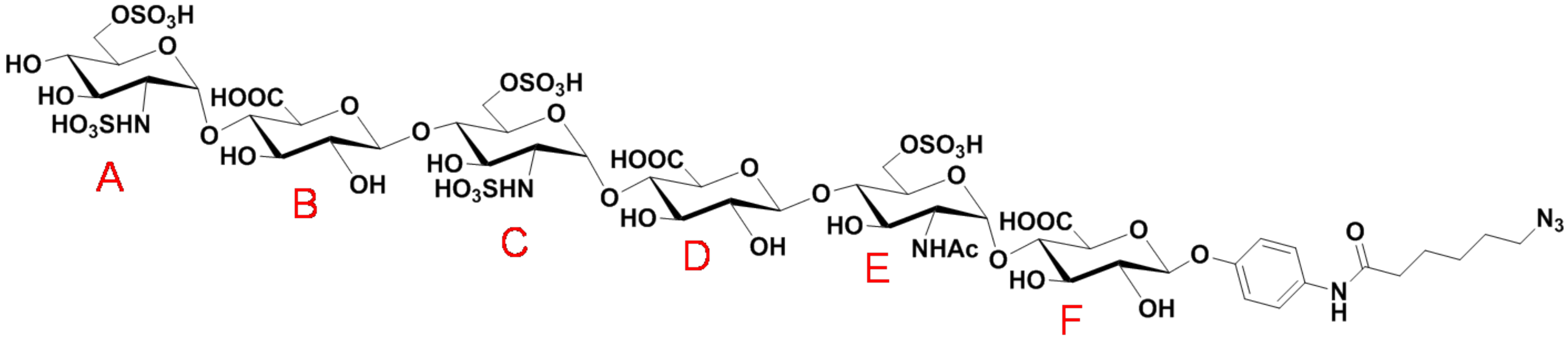

	1	2	3	4	5	6a	6b
A	5.59	3.22	3.58	3.52	3.85	4.12	4.31
B	4.56	3.35	3.82	3.72	3.80	----------	----------
C	5.58	3.26	3.65	3.70	3.98	4.15	4.41
D	4.56	3.35	3.82	3.72	3.80	----------	----------
E	5.39	3.89	3.82	3.71	4.01	4.15	4.41
F	5.06	3.56	3.77	3.79	3.92	----------	----------

Supplementary Fig. S10. ^{1}H NMR chemical shift assignments (in ppm) of compound **4**. The structure of compound **4** is shown. The protons of *p*NA-N_3 are not listed in the table.

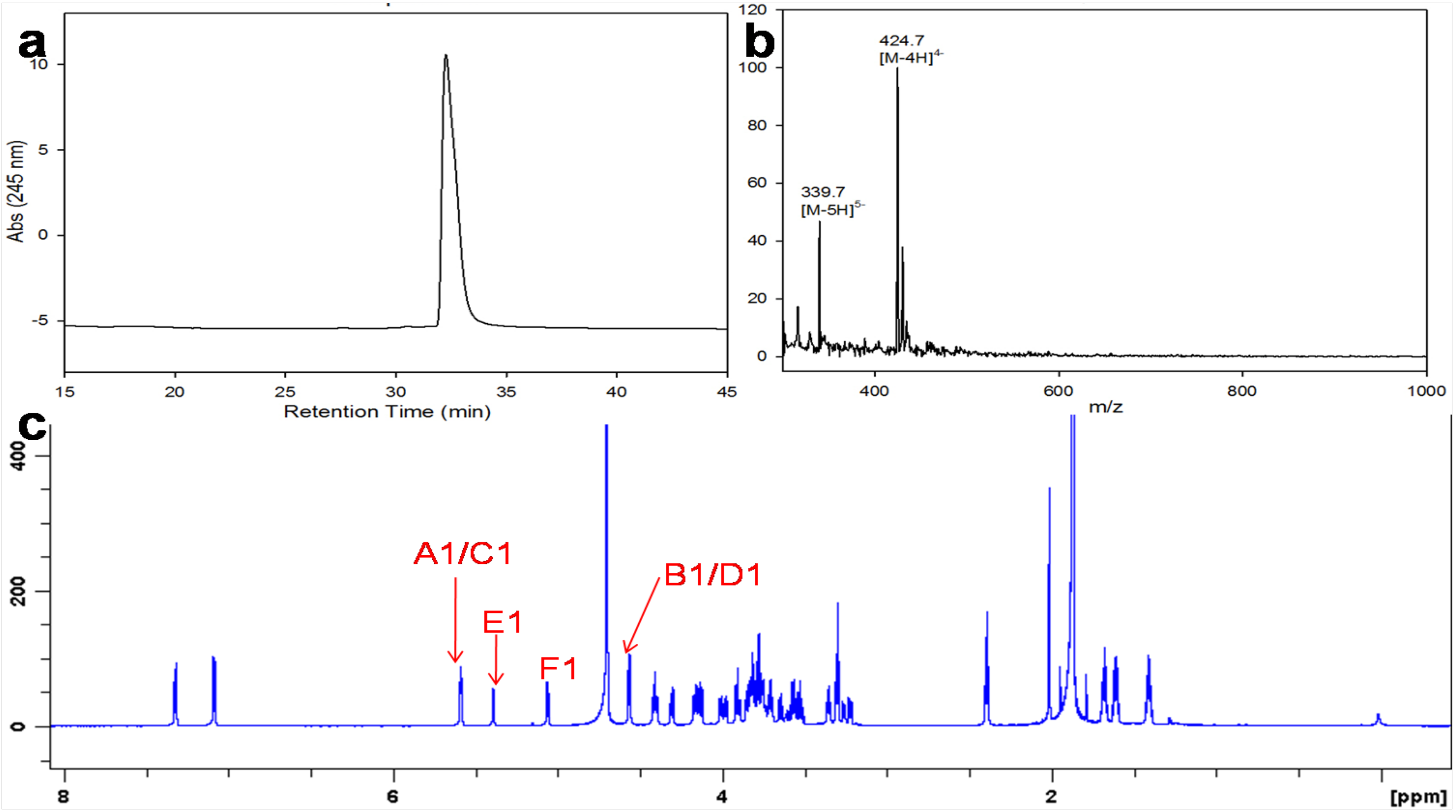

Supplementary Fig. S11. HPLC analysis (a), MS spectrum (b) and ^{1}H NMR spectrum (c) of compound **4**.

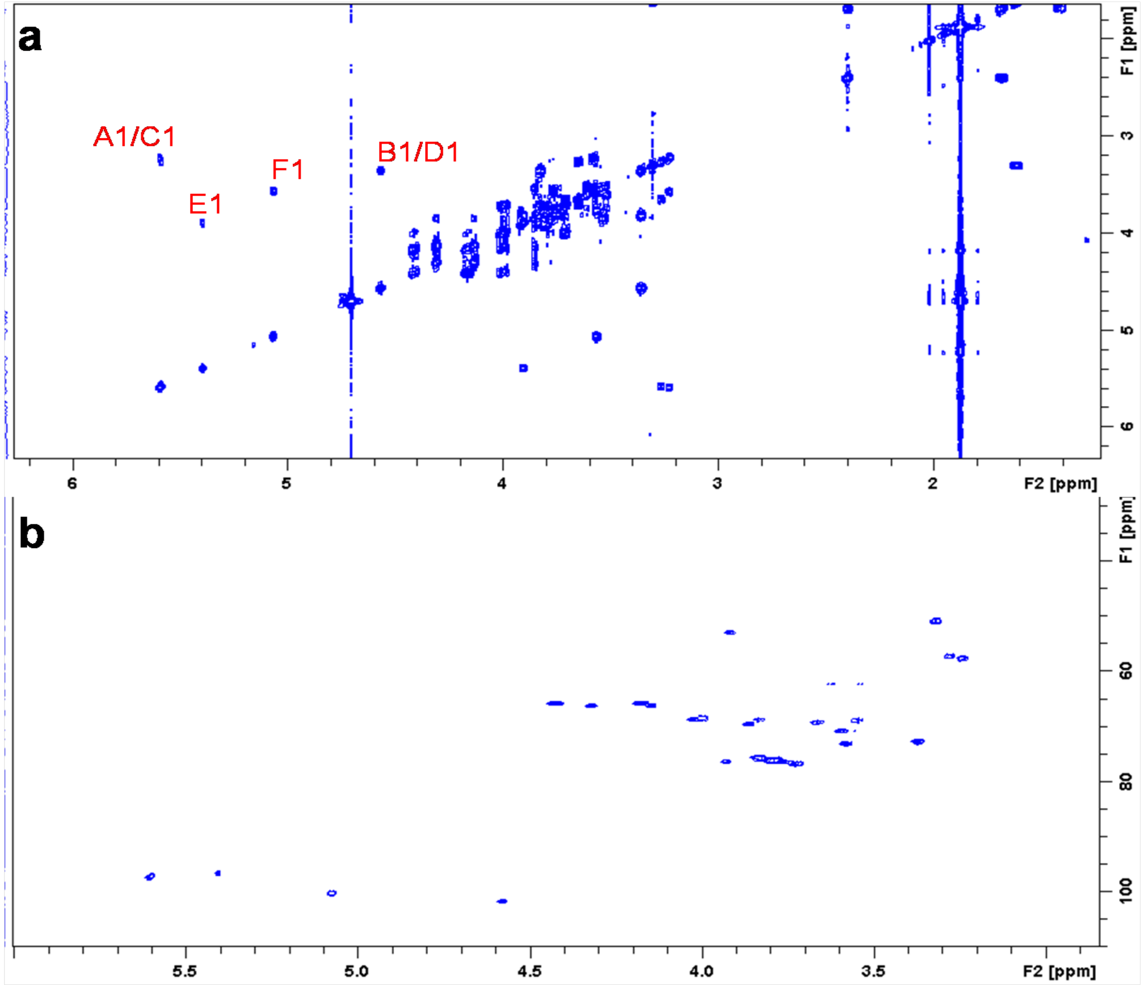

Supplementary Fig. S12. 2D NMR COSY (a) and HSQC (b) spectra of compound **4**.

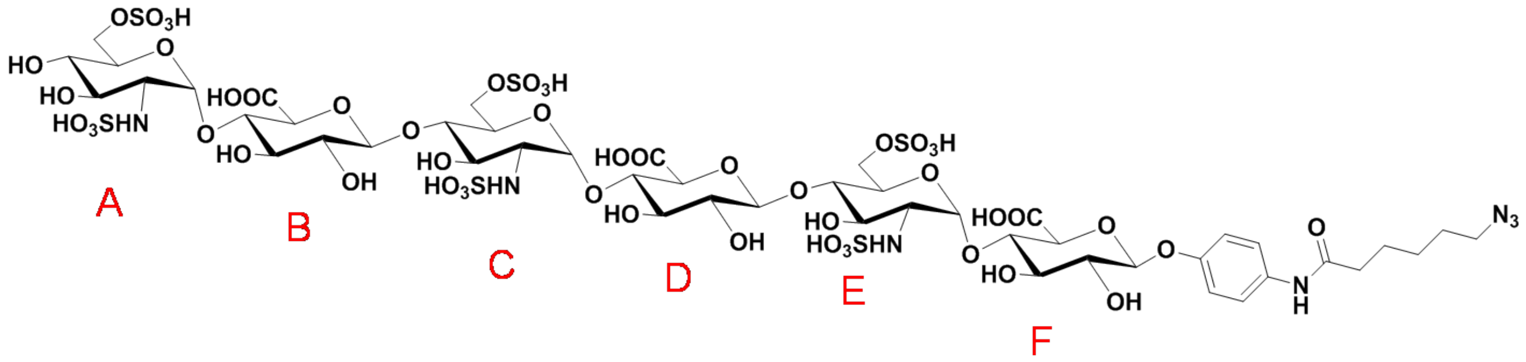

	1	2	3	4	5	6a	6b
A	5.57	3.20	3.55	3.51	3.82	4.11	4.28
B	4.55	3.32	3.79	3.74	3.72	----------	----------
C	5.56	3.23	3.62	3.66	3.96	4.12	4.38
D	4.55	3.32	3.79	3.74	3.72	----------	----------
E	5.60	3.25	3.62	3.66	3.96	4.12	4.38
F	5.06	3.57	3.87	3.81	3.89	----------	----------

Supplementary Fig. S13. ^{1}H NMR chemical shift assignments (in ppm) of compound **5**. The structure of compound **5** is shown. The protons of *p*NA-N_3 are not listed in the table.

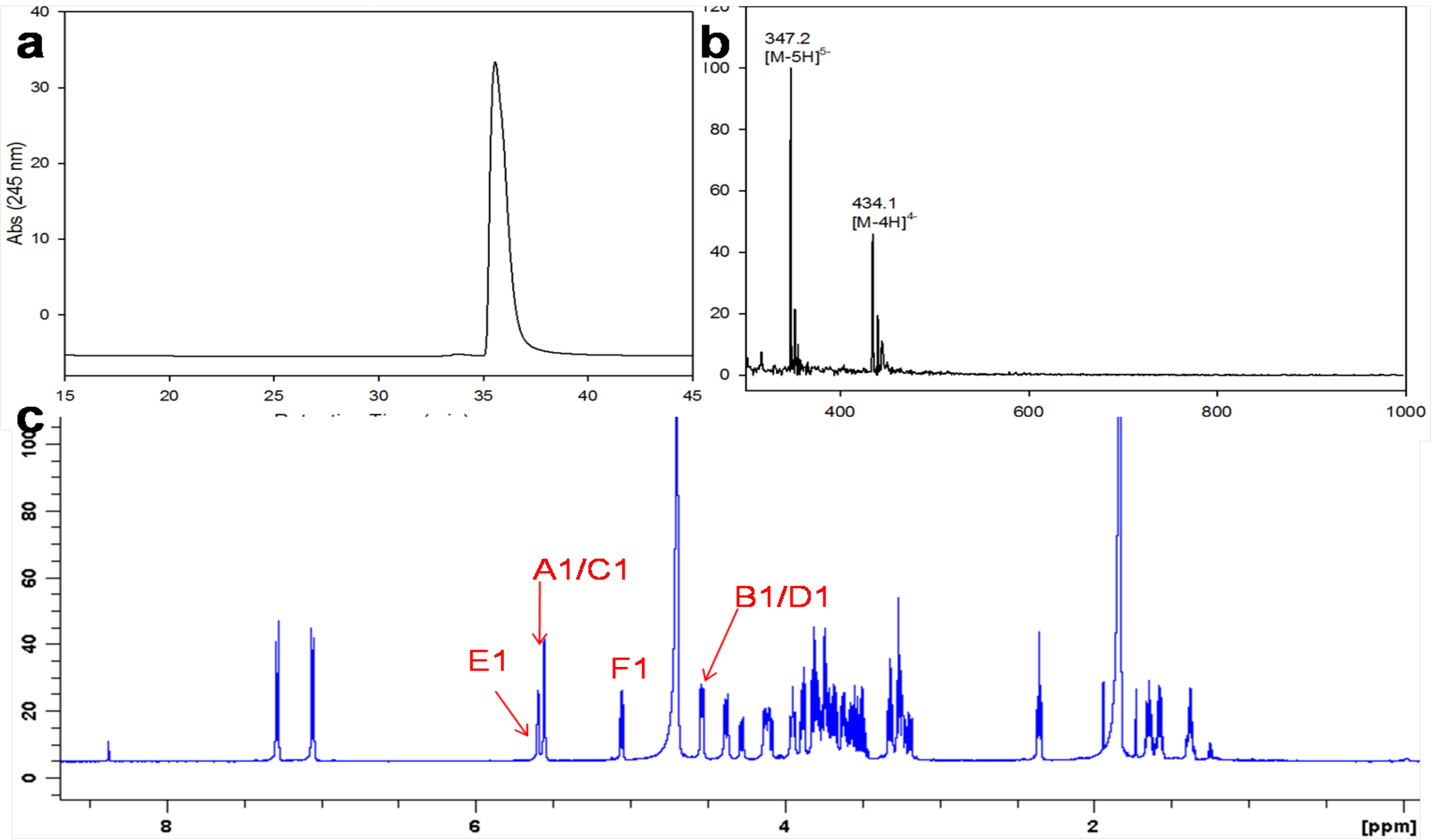

Supplementary Fig. S14. HPLC analysis (a), MS spectrum (b) and ^{1}H NMR spectrum (c) of compound **5**.

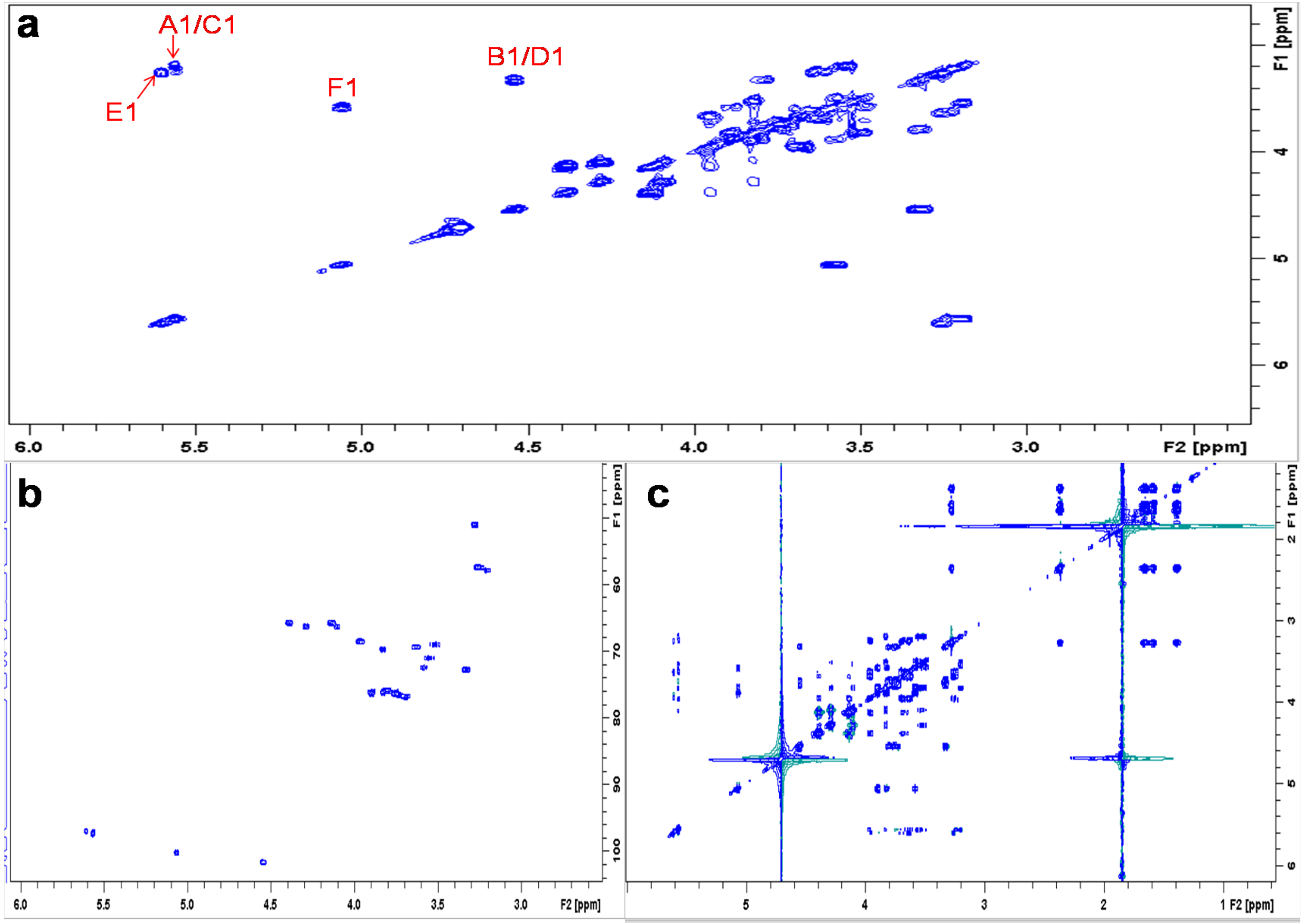

Supplementary Fig. S15. 2D NMR COSY (a), HSQC (b) and TOCSY (c) spectra of compound **5**.

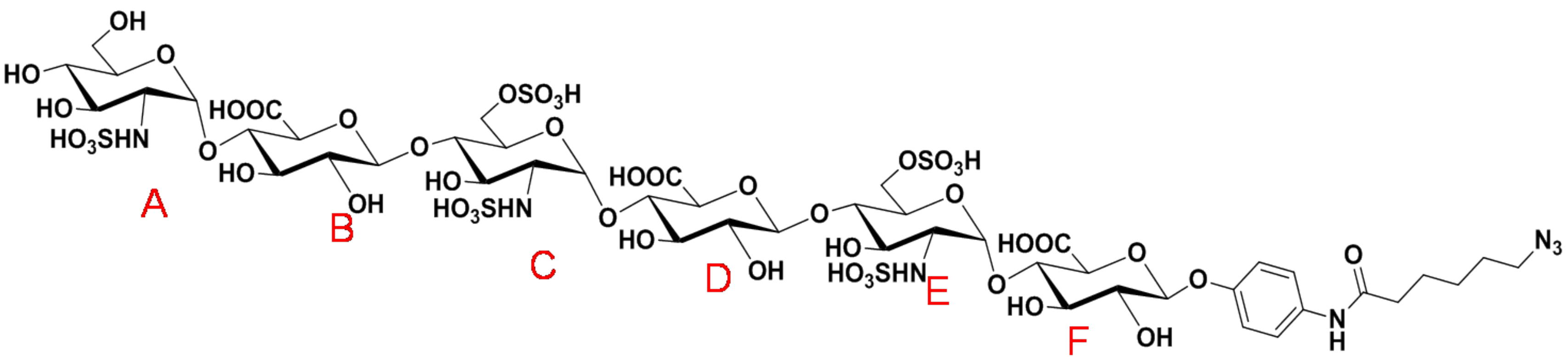

	1	2	3	4	5	6a	6b
A	5.54	3.14	3.53	3.39	3.65	3.65	3.71
B	4.54	3.30	3.78	3.73	3.75	-----------	-----------
C	5.56	3.22	3.60	3.67	3.95	4.11	4.37
D	4.54	3.30	3.78	3.73	3.75	-----------	-----------
E	5.60	3.24	3.61	3.68	3.94	4.12	4.37
F	5.05	3.56	3.85	3.78	3.88	-----------	-----------

Supplementary Fig. S16. ^{1}H NMR chemical shift assignments (in ppm) of compound **6**. The structure of compound **6** is shown. The protons of *p*NA-N_3 are not listed in the table.

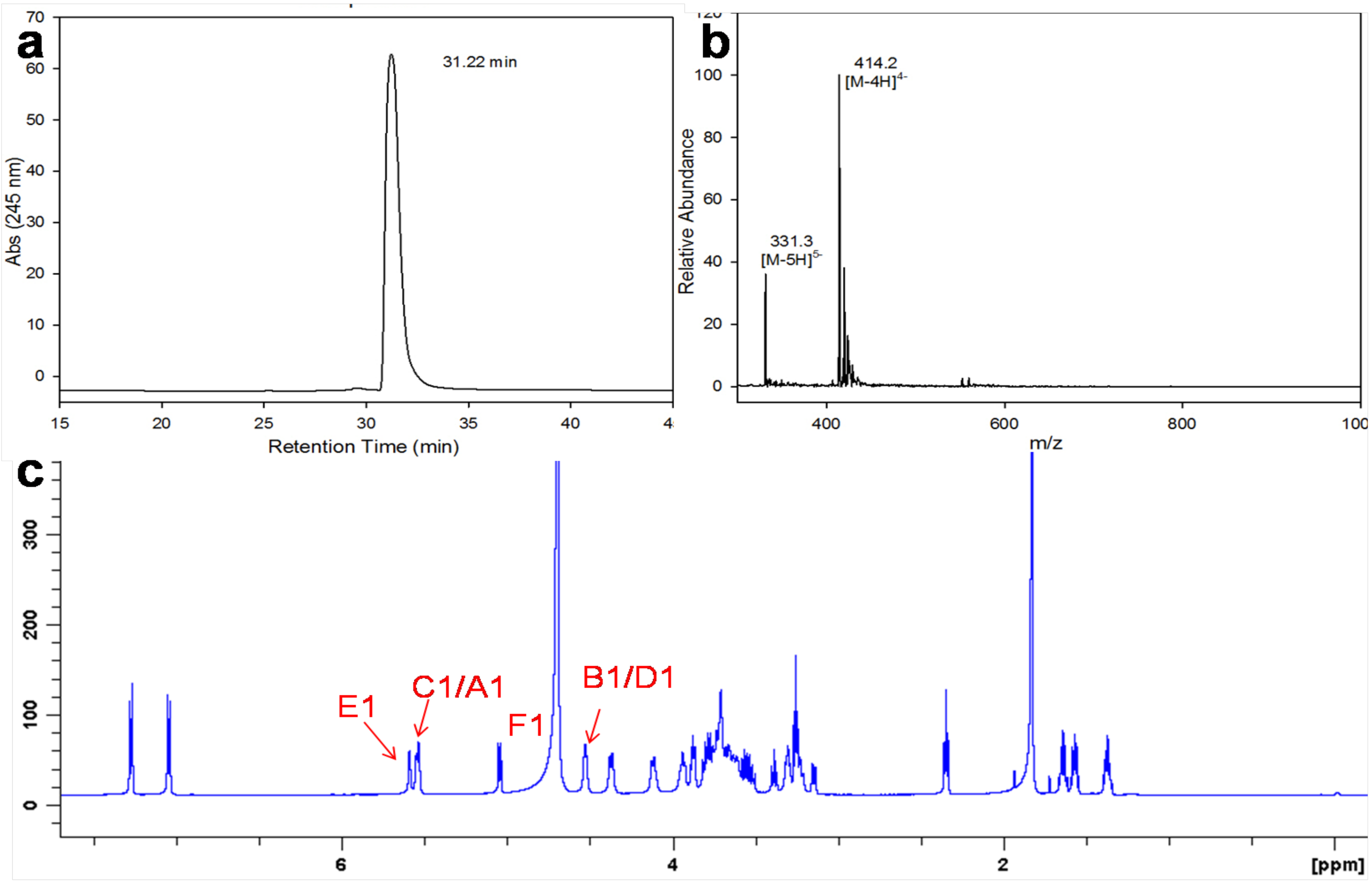

Supplementary Fig. S17. HPLC analysis (a), MS spectrum (b) and ^{1}H NMR spectrum (c) of compound **6**.

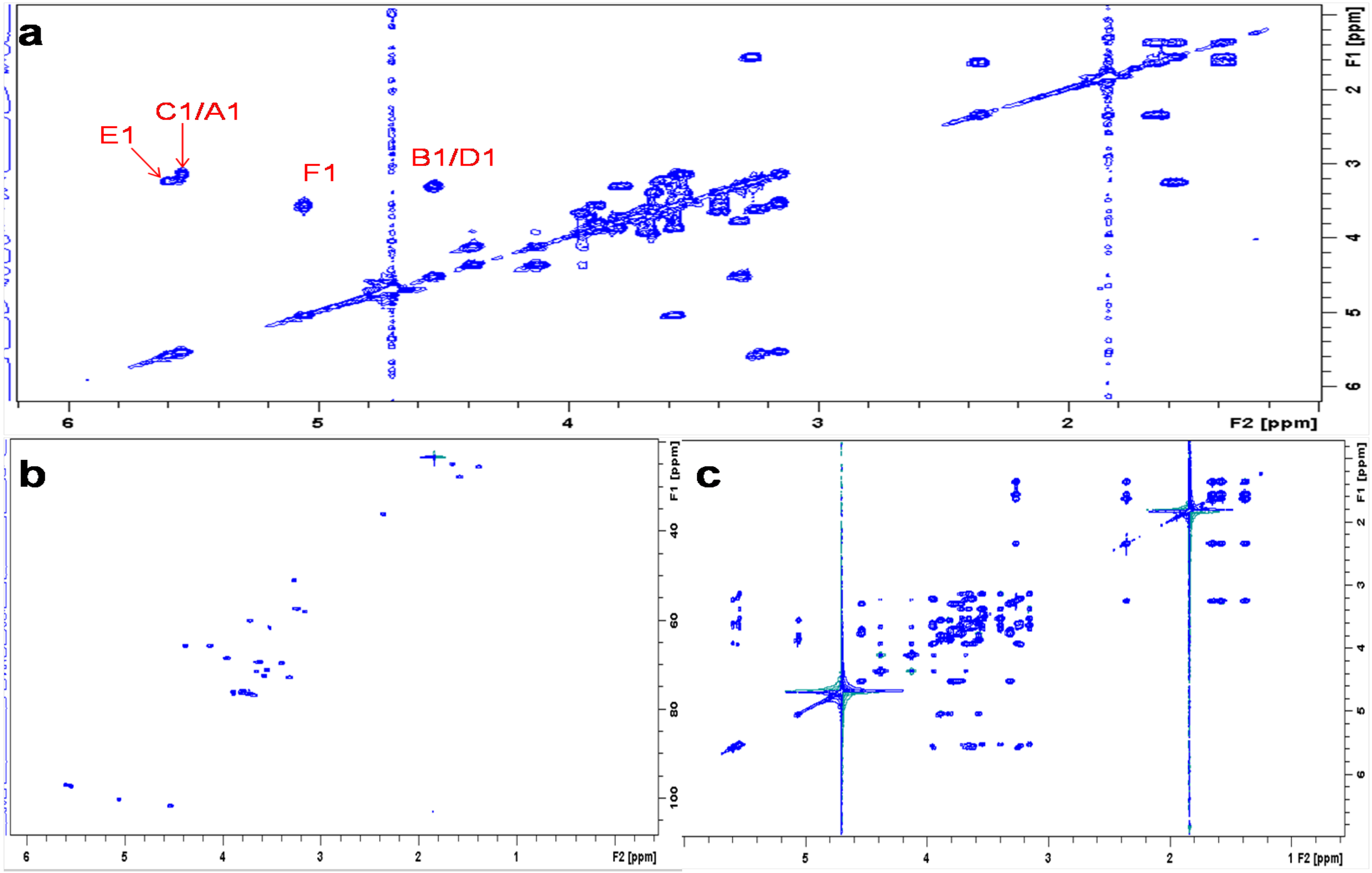

Supplementary Fig. S18. 2D NMR COSY (a), HSQC (b) and TOCSY (c) spectra of compound **6**.

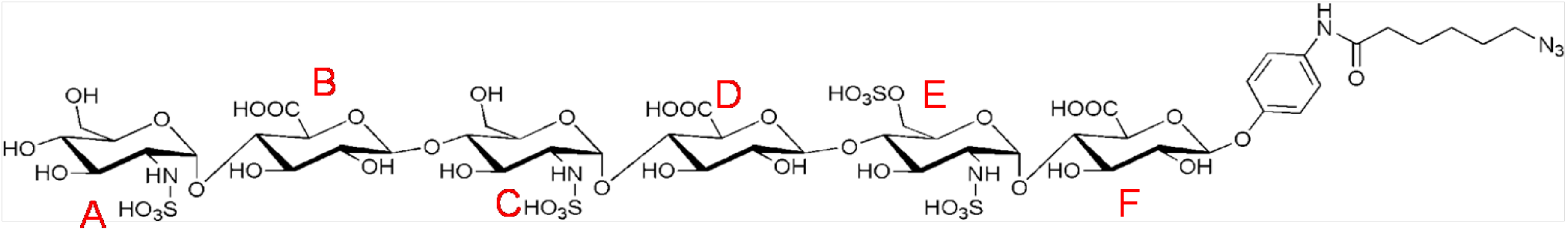

	1	2	3	4	5	6a	6b
A	5.54	3.12	3.50	3.41	3.57	3.63	3.70
B	4.49	3.32	3.75	3.74	3.87	----------	----------
C	5.51	3.18	3.62	3.68	3.70	3.72	3.81
D	4.57	3.30	3.78	3.73	3.91	----------	----------
E	5.56	3.23	3.61	3.67	3.85	4.12	4.35
F	5.08	3.58	3.88	3.83	4.02	----------	----------

Supplementary Fig. S19. ^{1}H NMR chemical shift assignments (in ppm) of compound **7**. The structure of compound **7** is shown. The protons of *p*NA-N_3 are not listed in the table.

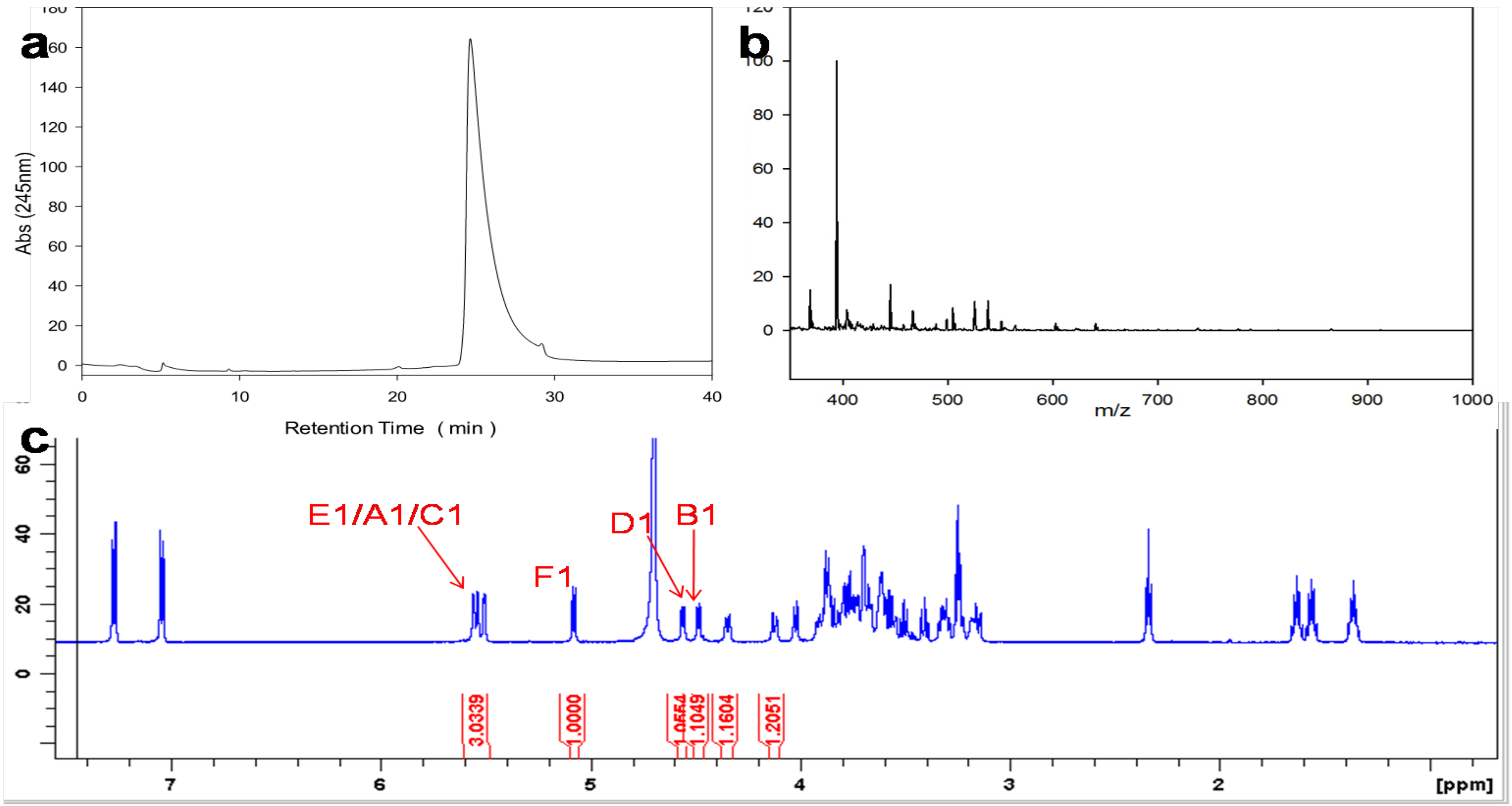

Supplementary Fig. S20. HPLC analysis (a), MS spectrum (b) and ^{1}H NMR spectrum (c) of compound **7**.

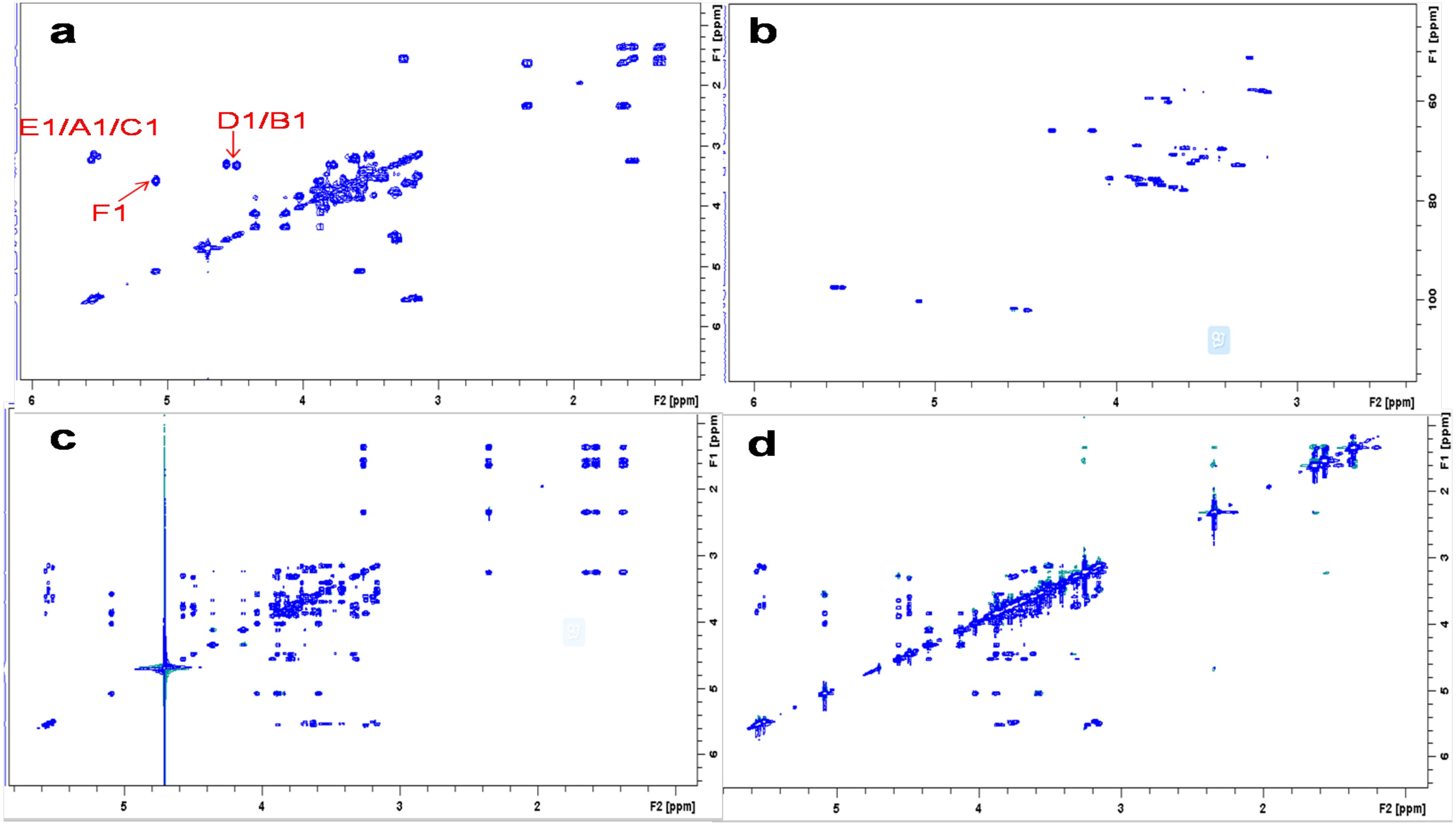

Supplementary Fig. S21. 2D NMR COSY (a), HSQC (b), TOCSY (c) and NOE (d) spectra of compound **7**.

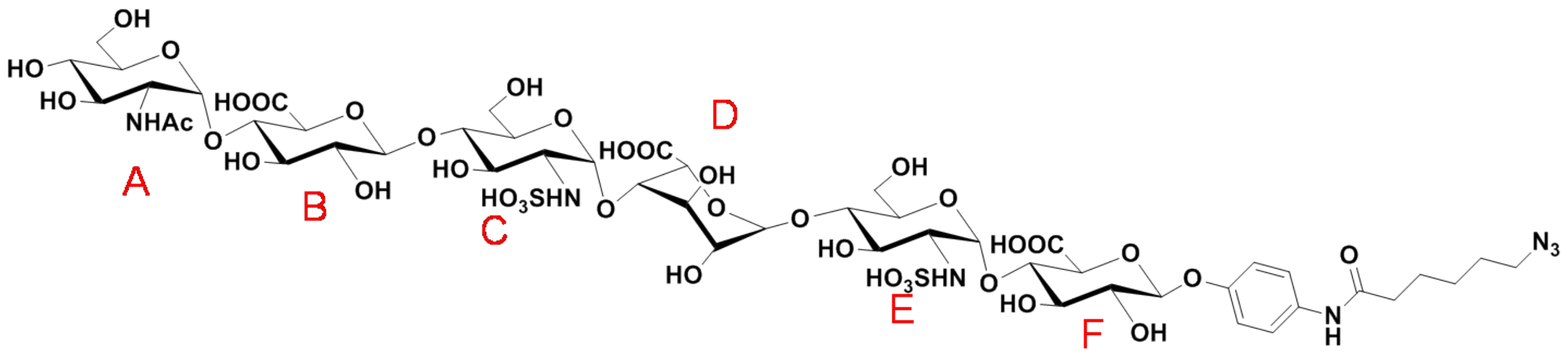

	1	2	3	4	5	6a	6b
A	5.34	3.80	3.65	3.40	3.72	3.72	3.81
B	4.43	3.28	3.63	3.68	3.73	----------	----------
C	5.29	3.15	3.61	3.78	3.73	3.73	3.80
D	4.88	3.64	4.02	3.98	4.71	----------	----------
E	5.54	3.19	3.57	3.63	3.73	3.79	3.86
F	5.04	3.55	3.85	3.80	3.88	----------	----------

Supplementary Fig. S22. ^{1}H NMR chemical shift assignments (in ppm) of compound **8**. The structure of compound **8** is shown. The protons of *p*NA-N_3 are not listed in the table.

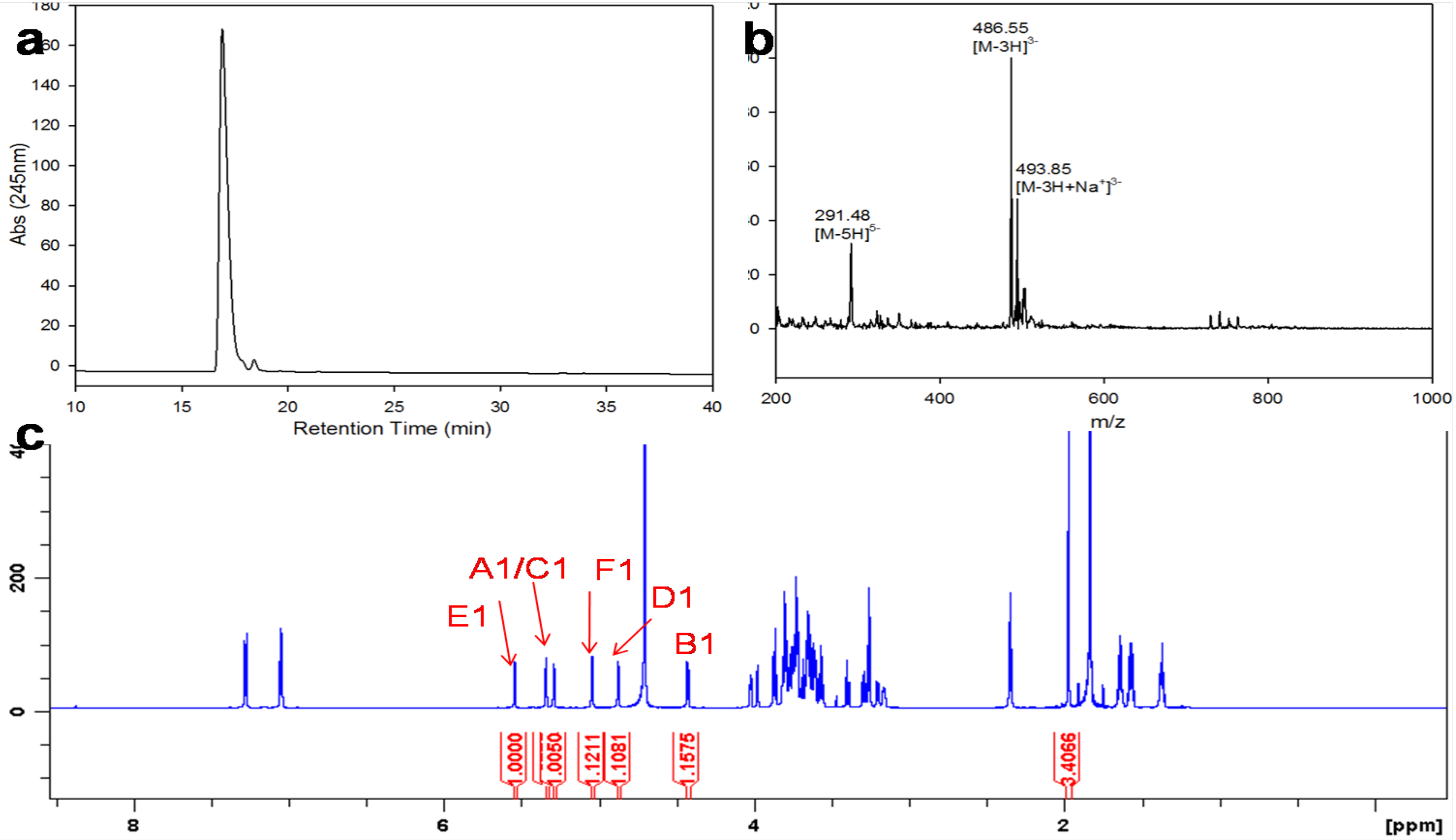

Supplementary Fig. S23. HPLC analysis (a), MS spectrum (b) and ^{1}H NMR spectrum (c) of compound **8**.

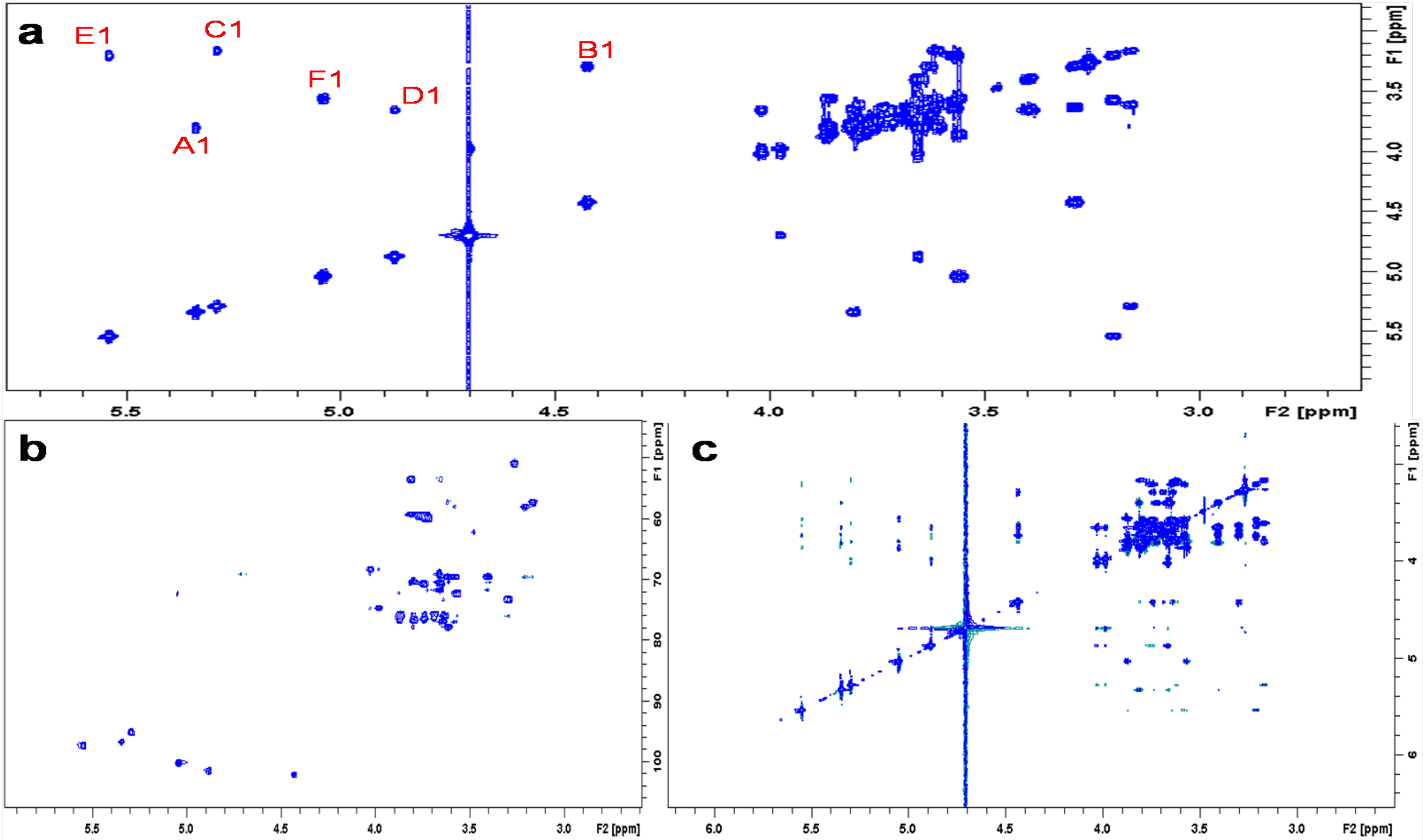

Supplementary Fig. S24. 2D NMR COSY (a), HSQC (b) and TOCSY (c) spectra of compound **8**.

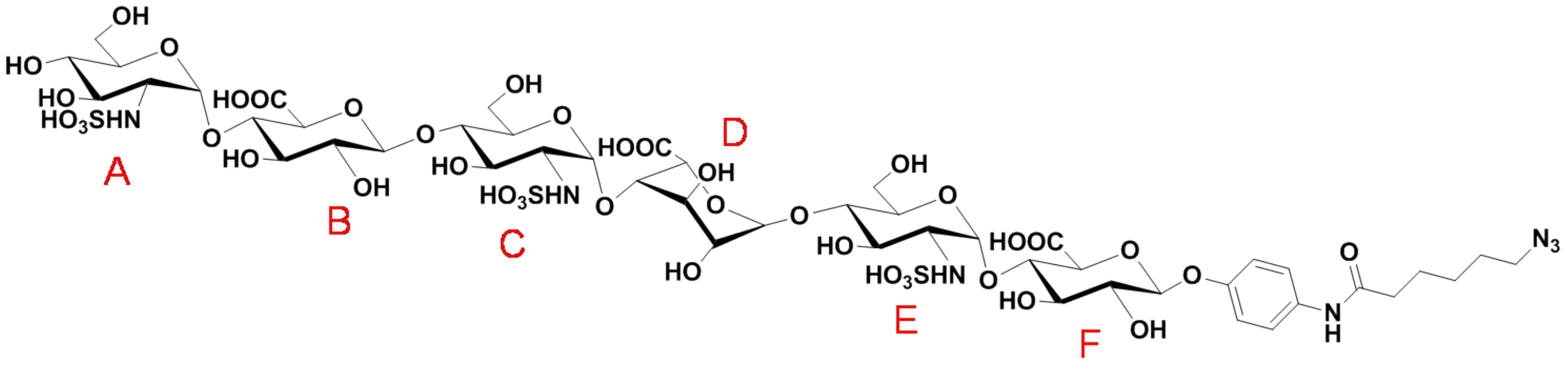

	1	2	3	4	5	6a	6b
A	5.54	3.15	3.51	3.43	3.60	3.65	3.71
B	4.50	3.34	3.76	3.80	3.83	----------	----------
C	5.28	3.17	3.58	3.65	3.68	3.65	3.70
D	4.96	3.71	4.07	3.99	4.98	----------	----------
E	5.54	3.16	3.60	3.65	3.65	3.65	3.72
F	5.06	3.57	3.89	3.79	3.96	----------	----------

Supplementary Fig. S25. ^{1}H NMR chemical shift assignments (in ppm) of compound **9**. The structure of compound **9** is shown. The protons of *p*NA-N_3 are not listed in the table.

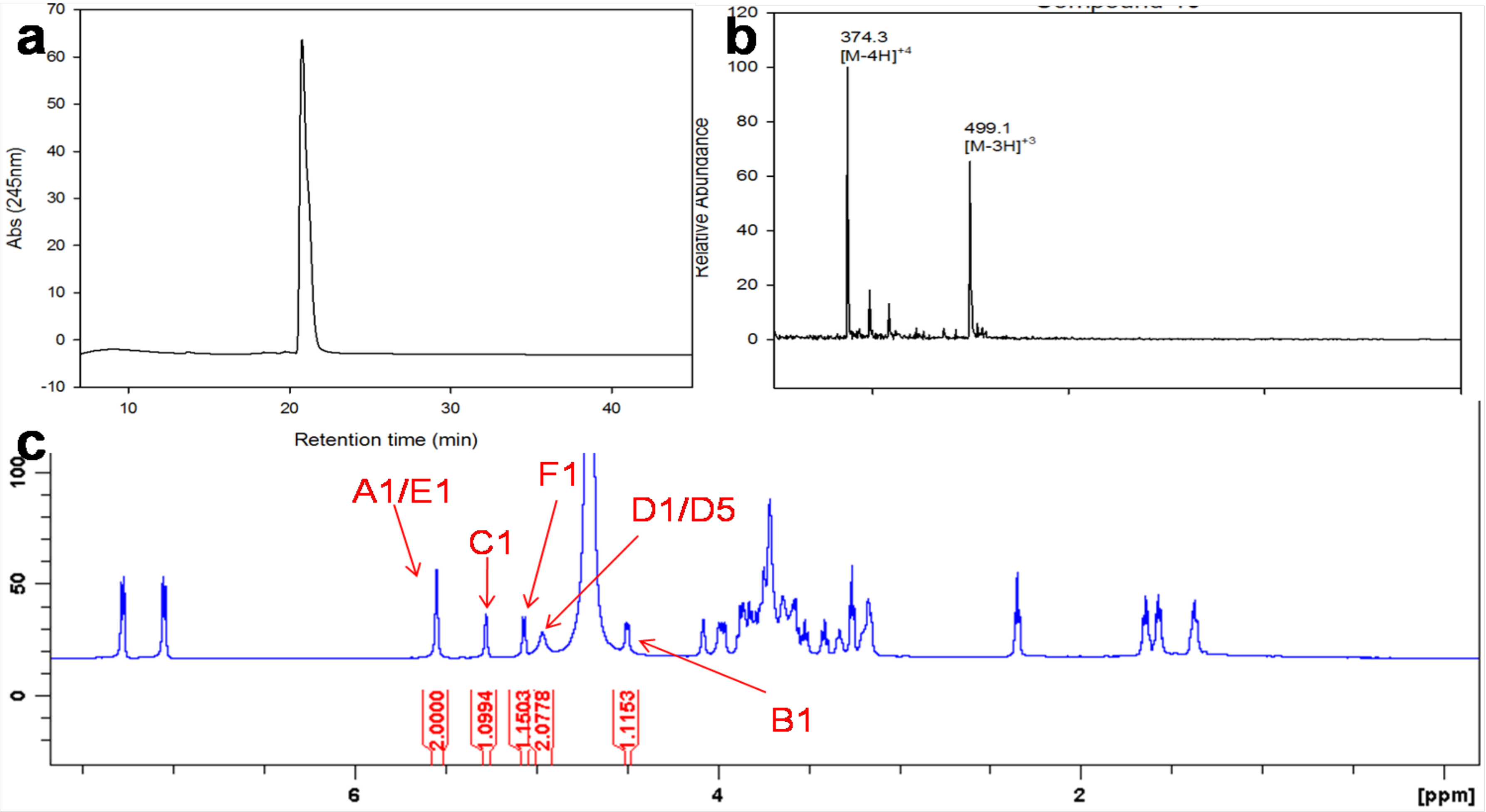

Supplementary Fig. S26. HPLC analysis (a), MS spectrum (b) and ^{1}H NMR spectrum (c) of compound **9**.

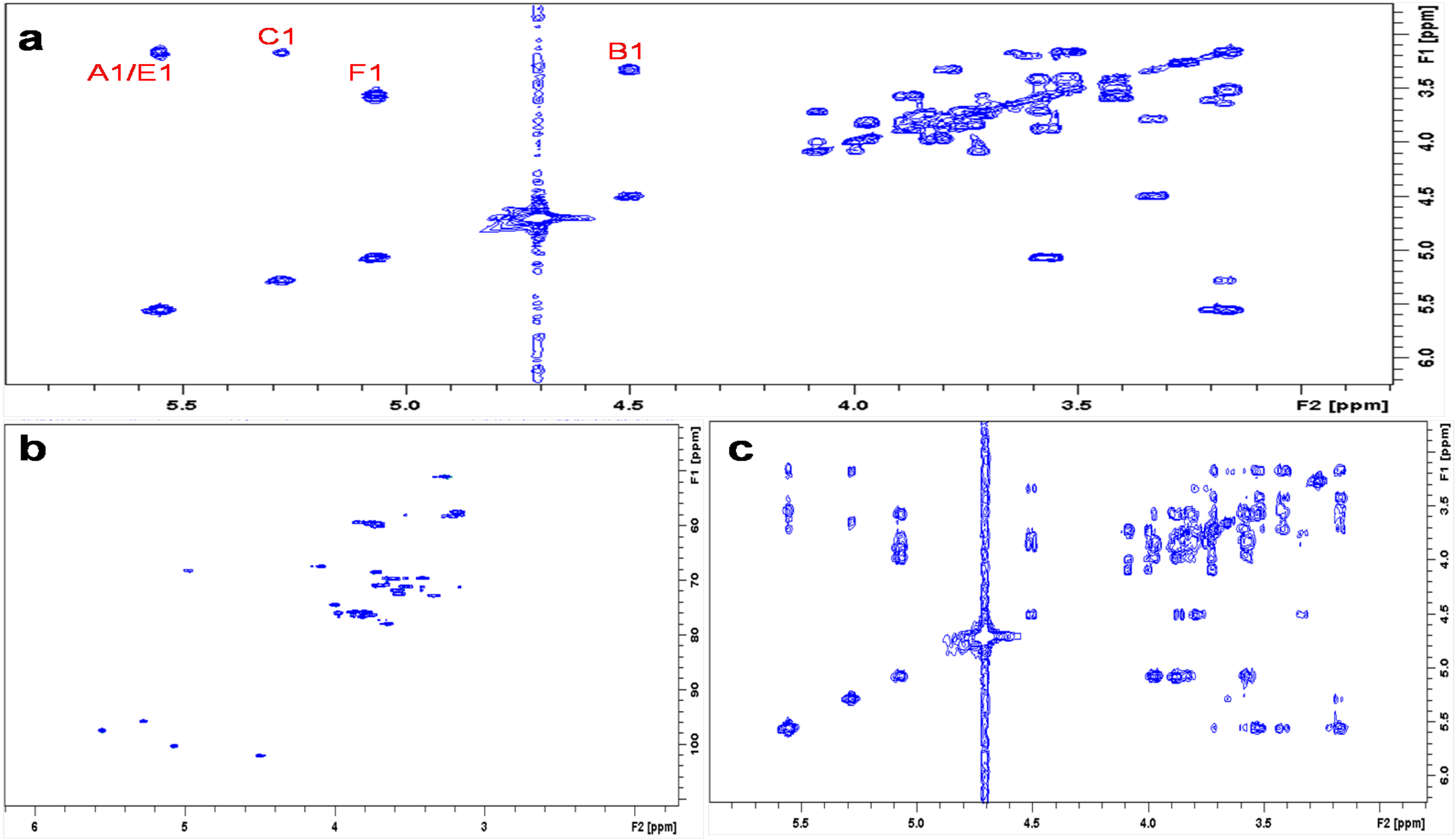

Supplementary Fig. S27. 2D NMR COSY (a), HSQC (b) and TOCSY (c) spectra of compound **9**.

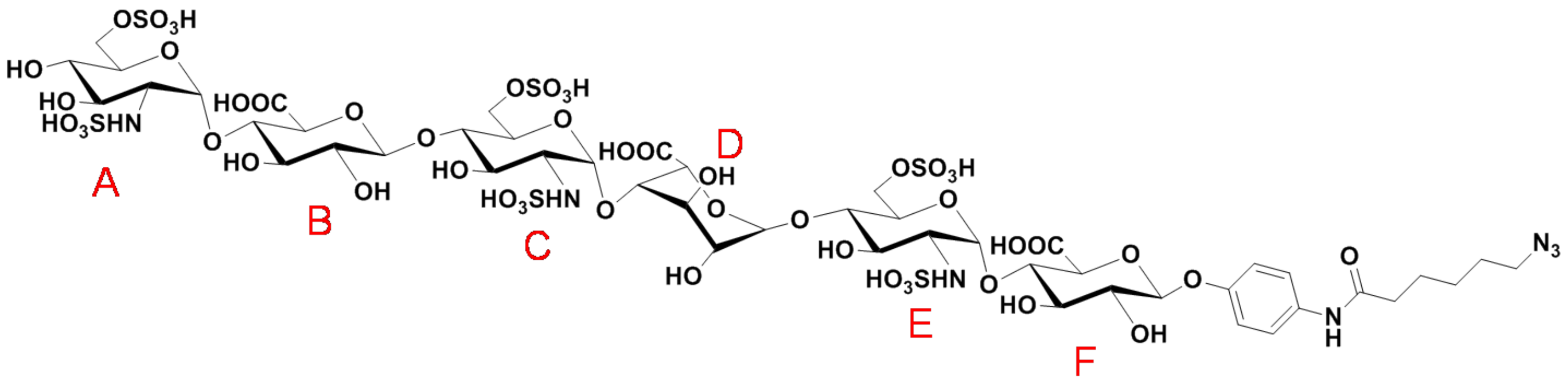

	1	2	3	4	5	6a	6b
A	5.57	3.18	3.56	3.49	3.82	4.10	4.28
B	4.55	3.33	3.81	3.75	3.75	----------	----------
C	5.27	3.20	3.65	3.73	3.95	4.13	4.40
D	4.97	3.73	4.06	4.00	4.77	----------	----------
E	5.56	3.23	3.62	3.68	3.95	4.10	4.28
F	5.06	3.58	3.85	3.81	3.89	----------	----------

Supplementary Fig. S28. ^{1}H NMR chemical shift assignments (in ppm) of compound **10**. The structure of compound **10** is shown. The protons of *p*NA-N_3 are not listed in the table.

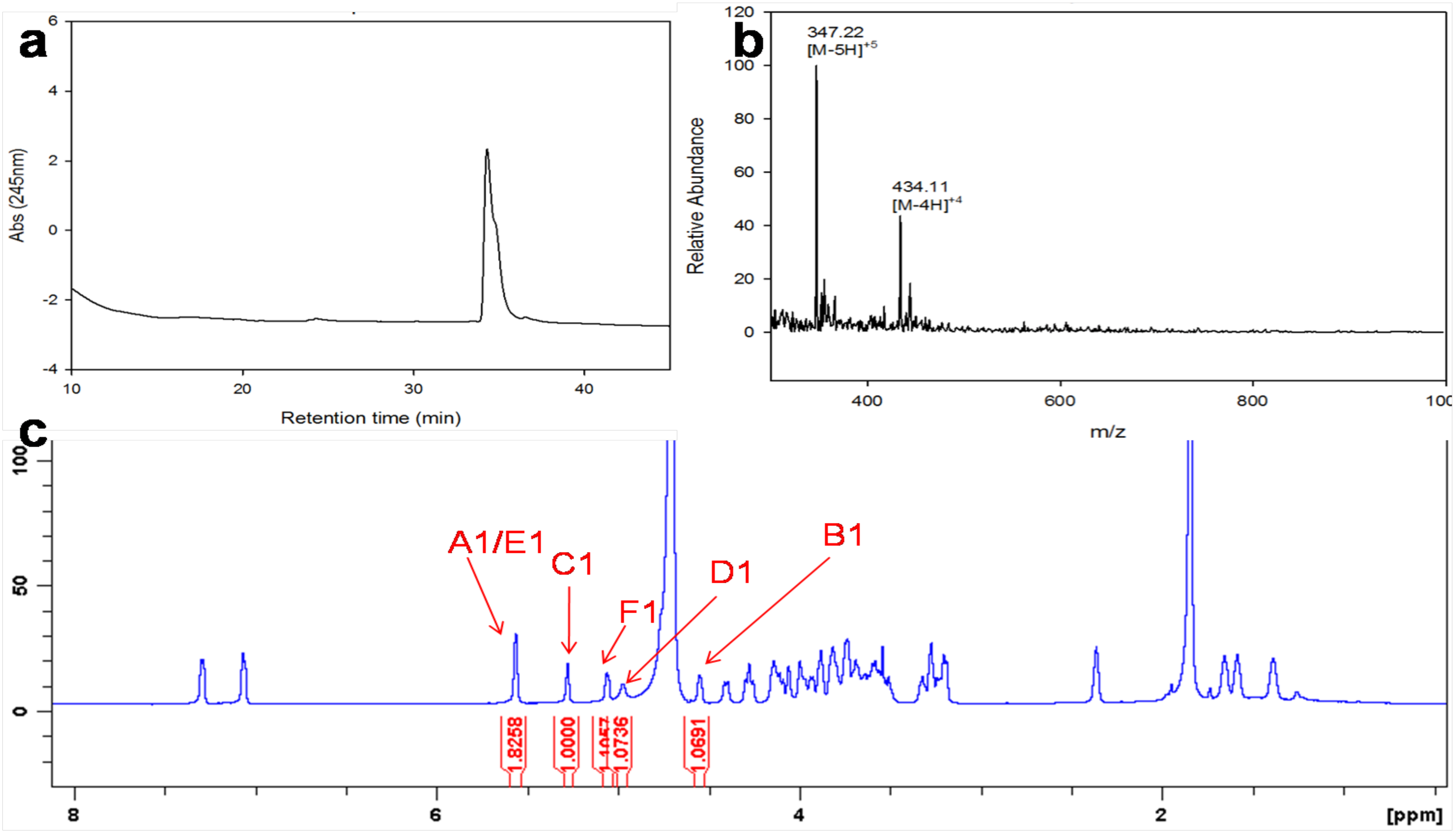

Supplementary Fig. S29. HPLC analysis (a), MS spectrum (b) and ^{1}H NMR spectrum (c) of compound **10**.

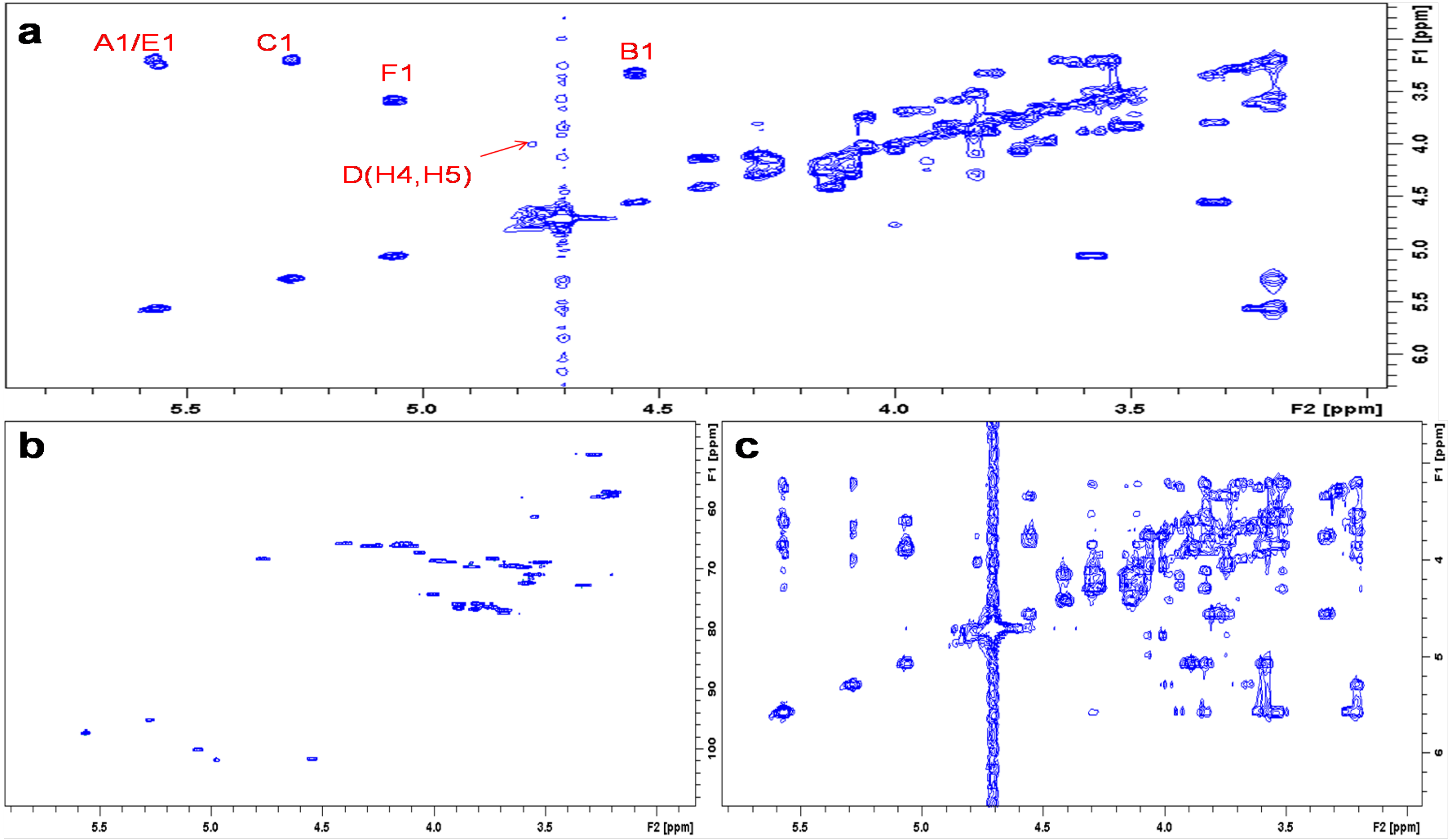

Supplementary Fig. S30. 2D NMR COSY (a), HSQC (b) and TOCSY (c) spectra of compound **10**.

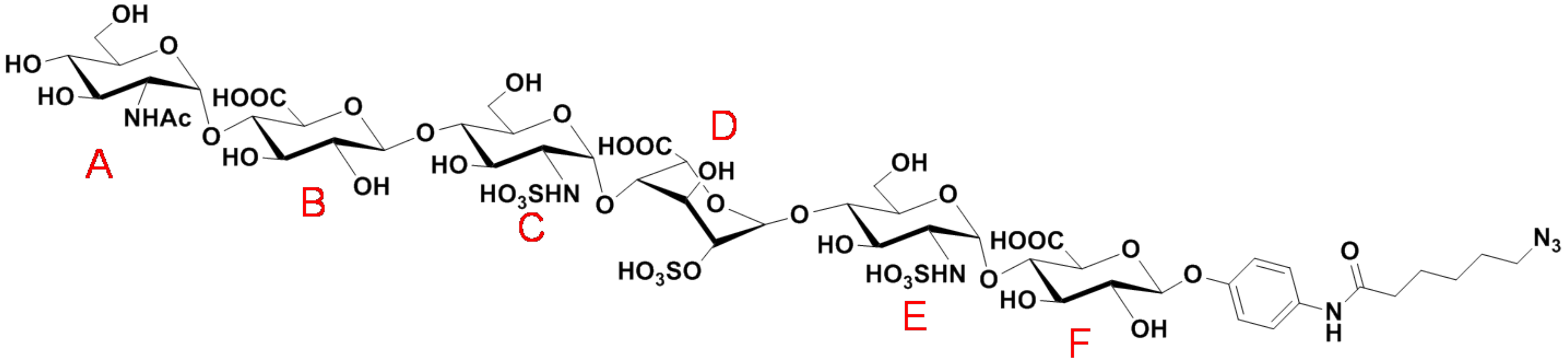

	1	2	3	4	5	6a	6b
A	5.34	3.81	3.66	3.41	3.66	3.73	3.79
B	4.44	3.30	3.65	3.70	3.75	-----------	-----------
C	5.24	3.19	3.64	3.72	3.72	3.62	3.77
D	5.20	4.25	4.17	3.97	4.79	-----------	-----------
E	5.53	3.20	3.61	3.65	3.80	3.75	3.86
F	5.06	3.56	3.85	3.81	3.89	-----------	-----------

Supplementary Fig. S31. ^{1}H NMR chemical shift assignments (in ppm) of compound **11**. The structure of compound **11** is shown. The protons of *p*NA-N_3 are not listed in the table.

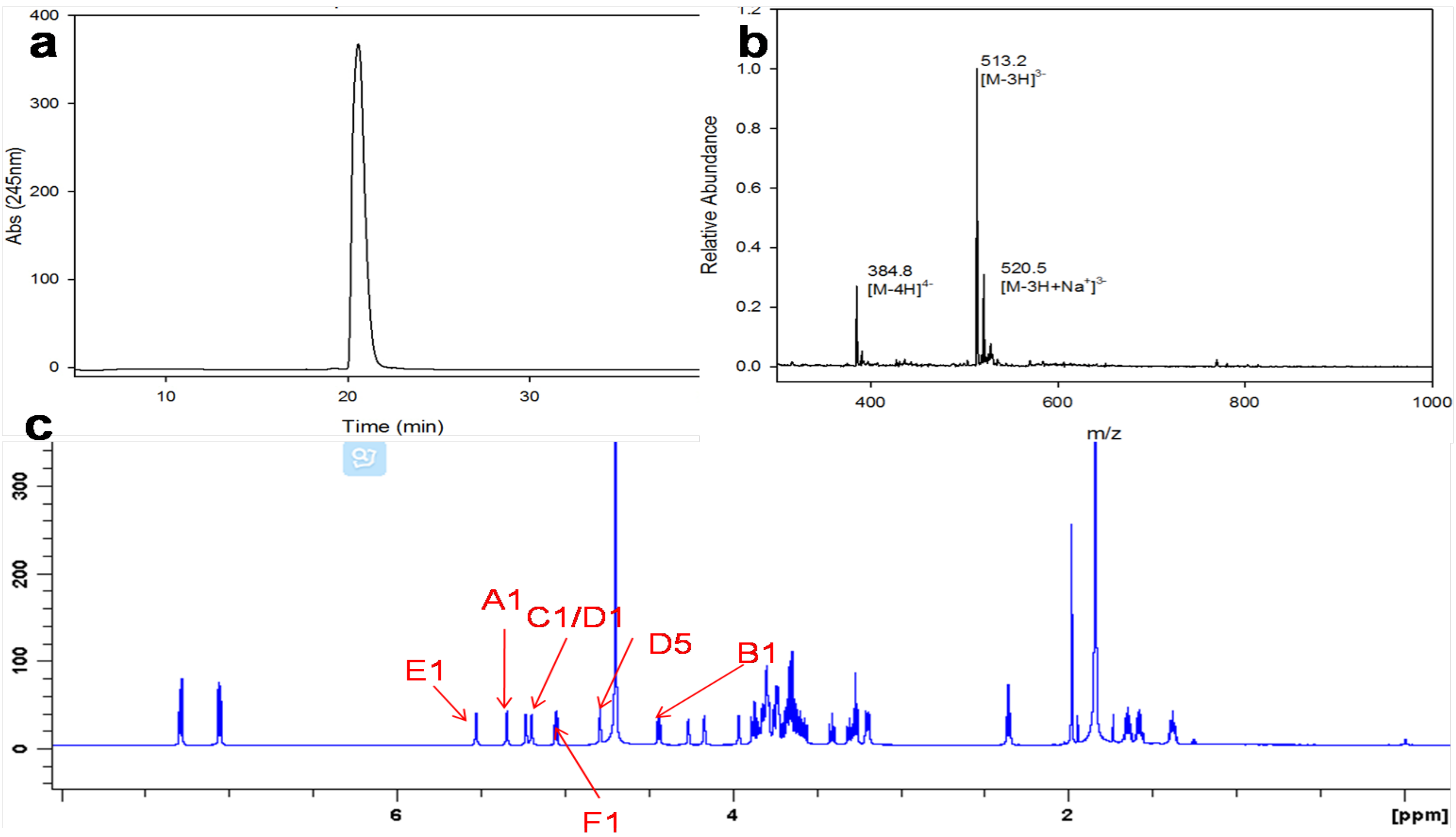

Supplementary Fig. S32. HPLC analysis (a), MS spectrum (b) and ^{1}H NMR spectrum (c) of compound **11**.

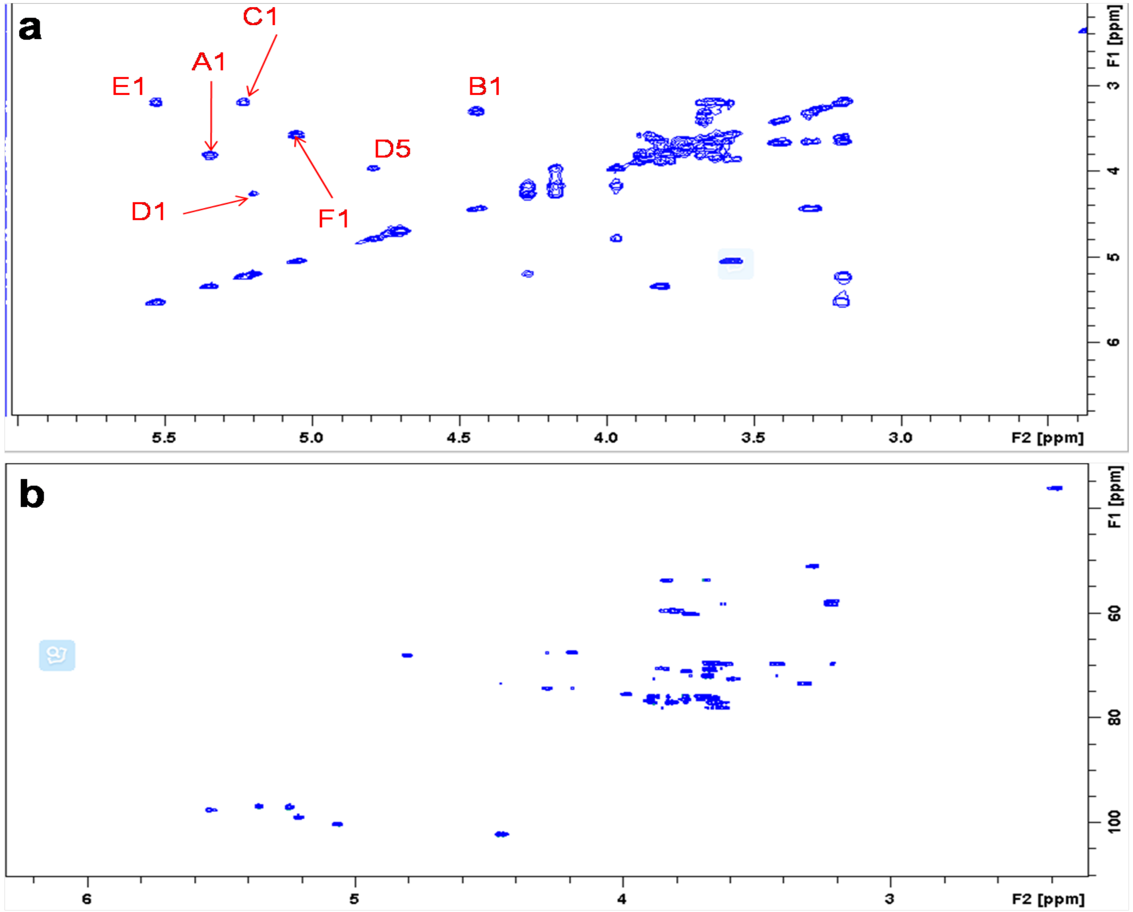

Supplementary Fig. S33. 2D NMR COSY (a) and HSQC (b) spectra of compound **11**.

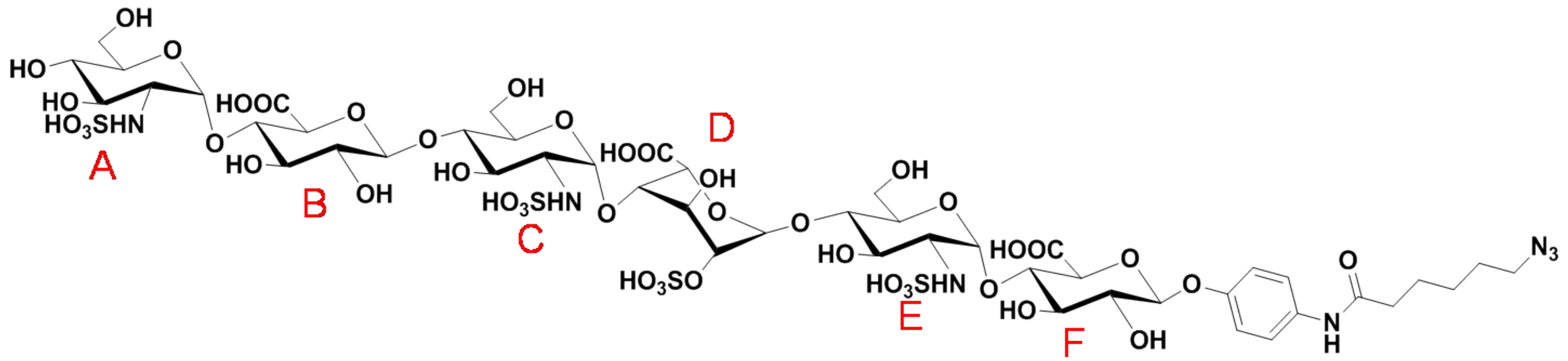

	1	2	3	4	5	6a	6b
A	5.57	3.15	3.51	3.39	3.63	3.66	3.70
B	4.45	3.32	3.76	3.73	3.78	-----------	-----------
C	5.23	3.17	3.64	3.73	3.79	3.73	3.83
D	5.18	4.23	4.15	3.96	4.77	-----------	-----------
E	5.51	3.18	3.59	3.63	3.73	3.63	3.78
F	5.04	3.56	3.83	3.78	3.87	-----------	-----------

Supplementary Fig. S34. ^{1}H NMR chemical shift assignments (in ppm) of compound **12**. The structure of compound **12** is shown. The protons of *p*NA-N_3 are not listed in the table.

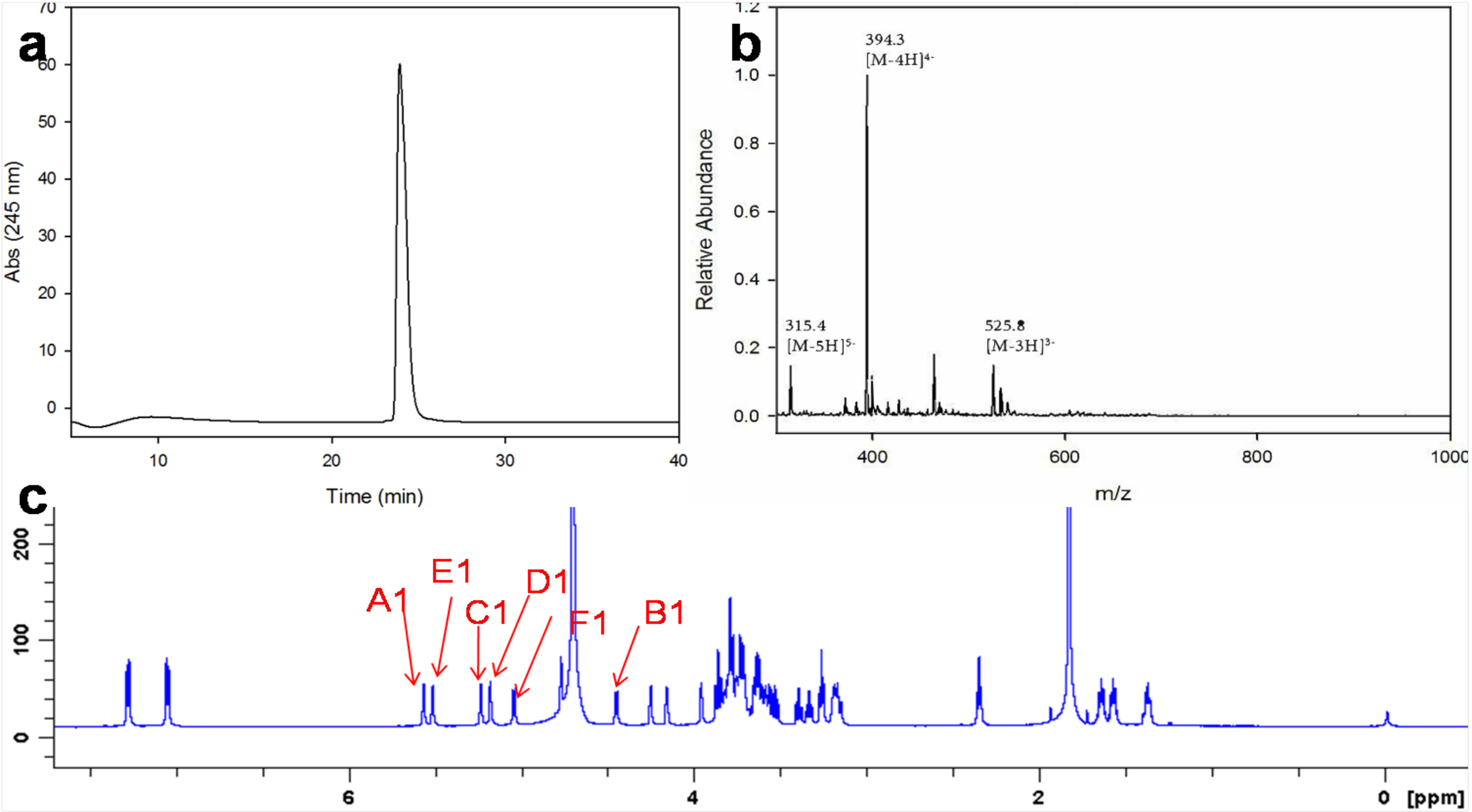

Supplementary Fig. S35. HPLC analysis (a), MS spectrum (b) and ^{1}H NMR spectrum (c) of compound **12**.

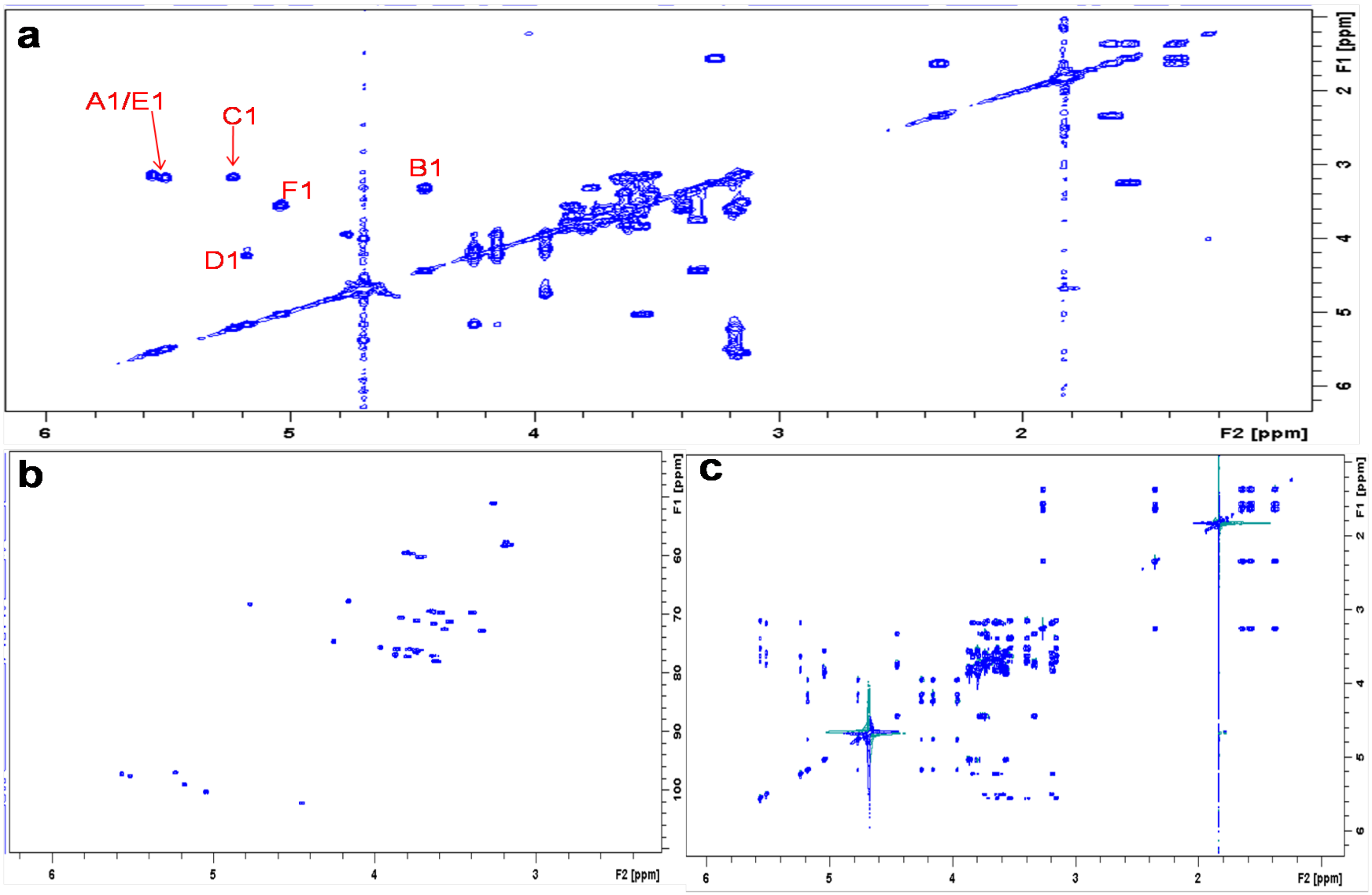

Supplementary Fig. S36. 2D NMR COSY (a), HSQC (b) and TOCSY (c) spectra of compound **12**.

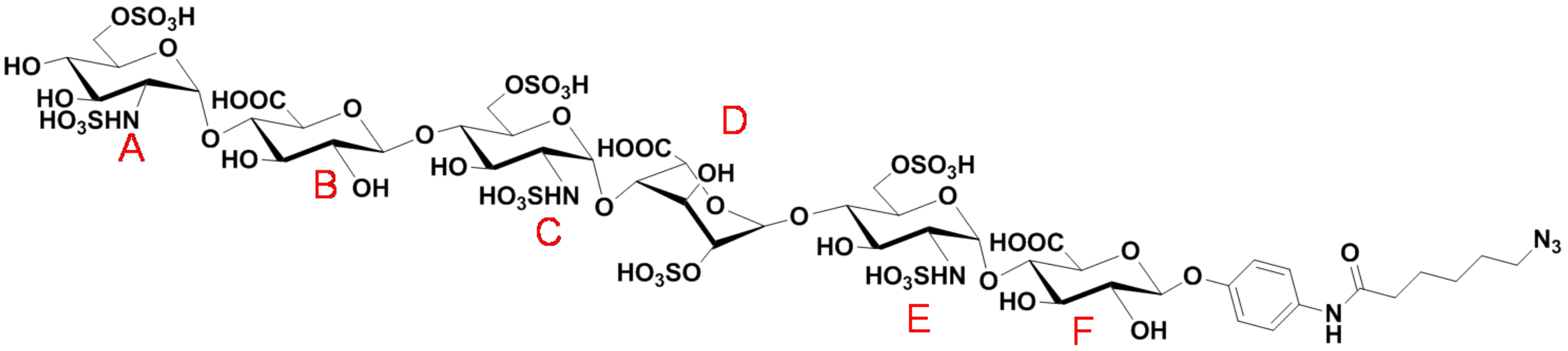

	1	2	3	4	5	6a	6b
A	5.55	3.18	3.56	3.50	3.83	4.09	4.29
B	4.54	3.31	3.77	3.75	3.79	-----------	-----------
C	5.35	3.21	3.63	3.67	4.02	4.16	4.39
D	5.18	4.26	4.13	4.03	4.75	-----------	-----------
E	5.53	3.24	3.61	3.74	3.92	4.16	4.33
F	5.05	3.56	3.85	3.81	3.88	-----------	-----------

Supplementary Fig. S37. ^{1}H NMR chemical shift assignments (in ppm) of compound **13**. The structure of compound **13** is shown. The protons of *p*NA-N_3 are not listed in the table.

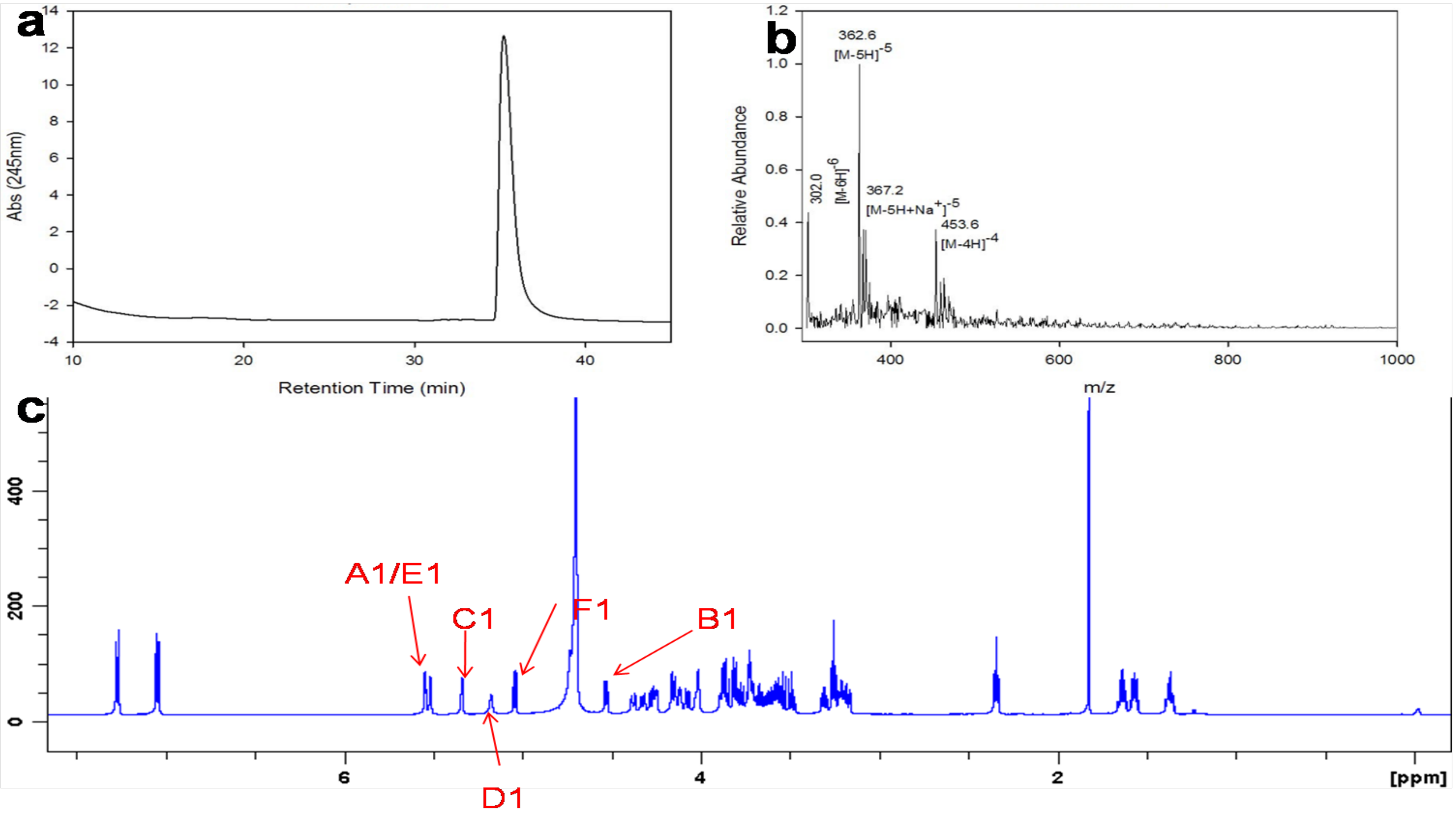

Supplementary Fig. S38. HPLC analysis (a), MS spectrum (b) and ^{1}H NMR spectrum (c) of compound **13**.

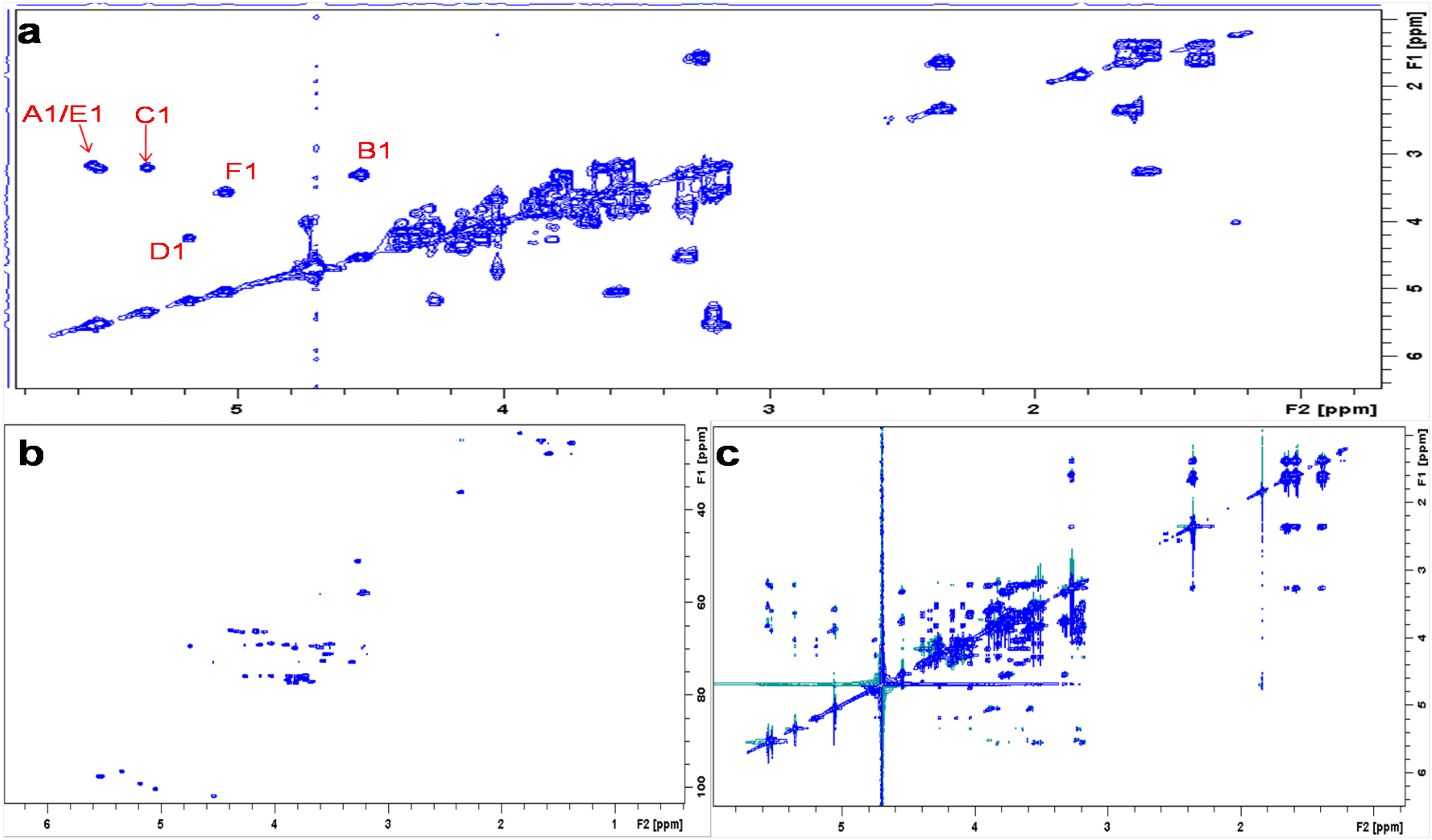

Supplementary Fig. S39. 2D NMR COSY (a), HSQC (b) and TOCSY (c) spectra of compound **13**.

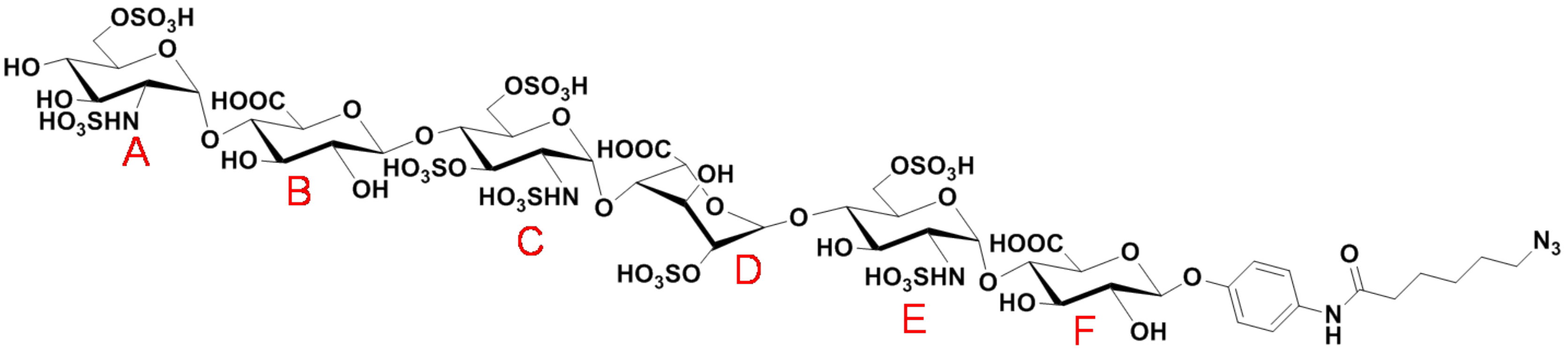

	1	2	3	4	5	6a	6b
A	5.58	3.20	3.56	3.51	3.84	4.12	4.32
B	4.56	3.36	3.78	3.88	3.80	----------	----------
C	5.43	3.39	4.33	3.92	4.09	4.13	4.34
D	5.16	4.28	4.15	4.08	4.76	----------	----------
E	5.56	3.25	3.63	3.74	3.92	4.21	4.41
F	5.07	3.59	3.88	3.81	3.91	----------	----------

Supplementary Fig. S40. ^{1}H NMR chemical shift assignments (in ppm) of compound **14**. The structure of compound **14** is shown. The protons of *p*NA-N_3 are not listed in the table.

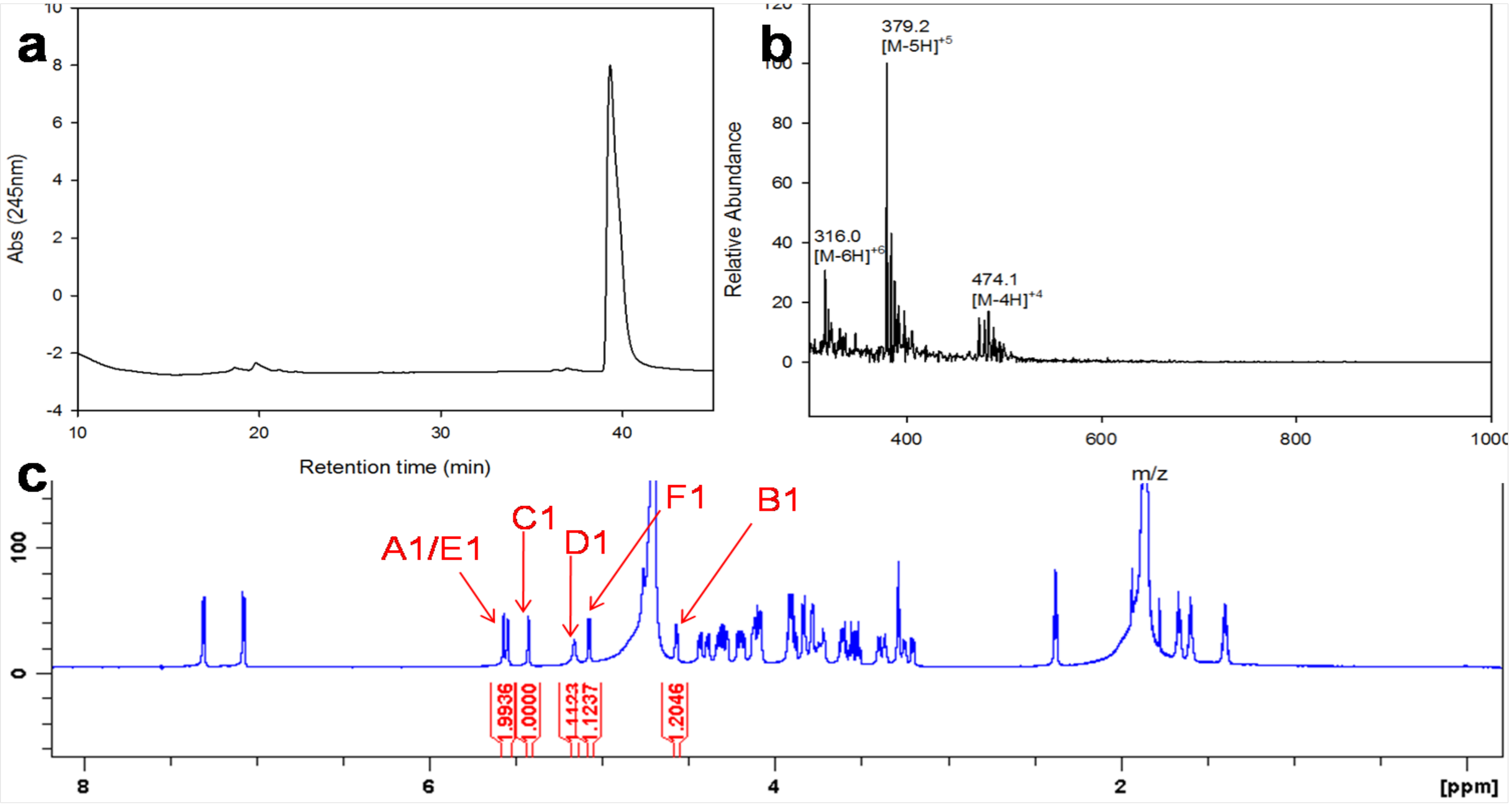

Supplementary Fig. S41. HPLC analysis (a), MS spectrum (b) and ^{1}H NMR spectrum (c) of compound **14.**

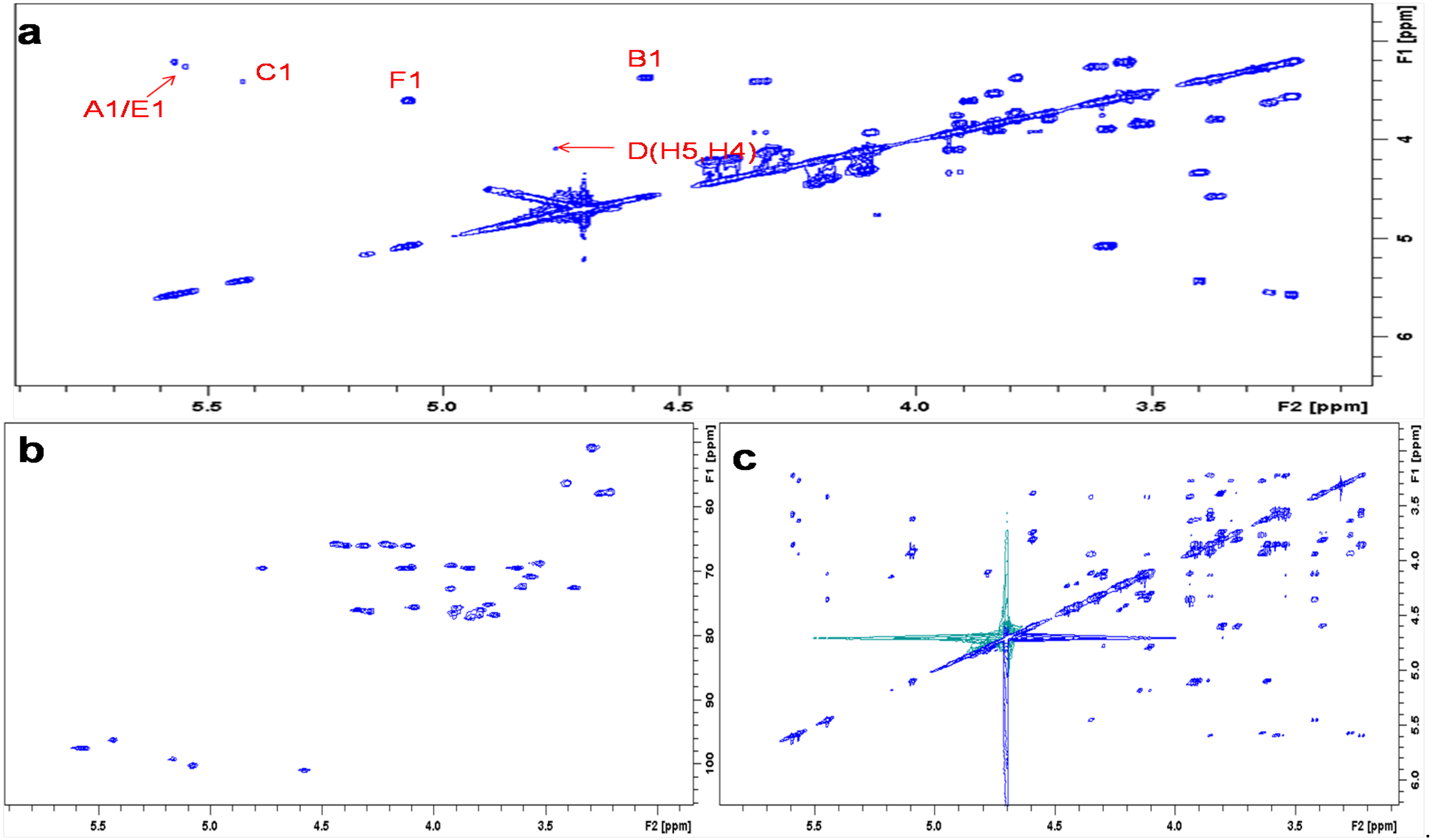

Supplementary Fig. S42. 2D NMR COSY (a), HSQC (b) and TOCSY (c) spectra of compound **14**.

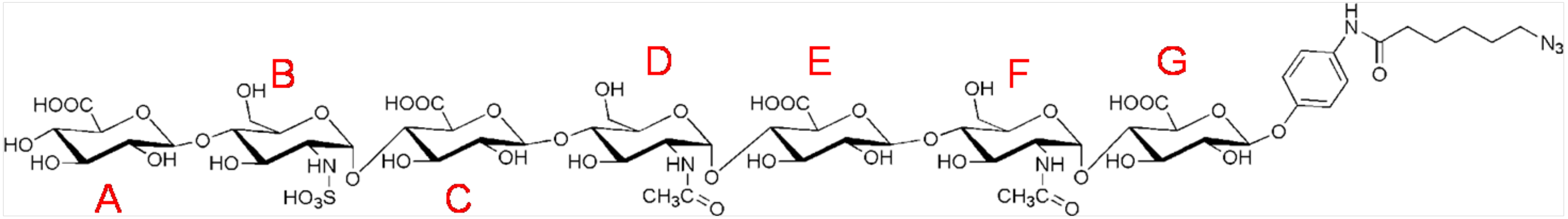

	1	2	3	4	5	6a	6b
A	4.45	3.30	3.46	3.48	3.78	----------	----------
B	5.53	3.15	3.62	3.71	3.76	3.62	3.80
C	4.45	3.30	3.77	3.79	3.80	----------	----------
D	5.29	3.80	3.74	3.77	3.77	3.62	3.82
E	4.45	3.30	3.64	3.69	3.78	----------	----------
F	5.32	3.80	3.77	3.77	3.77	3.62	3.83
G	5.01	3.50	3.70	3.76	3.90	----------	----------

Supplementary Fig. S43. ^{1}H NMR chemical shift assignments (in ppm) of compound **15**. The structure of compound **15** is shown. The protons of *p*NA-N_3 are not listed in the table.

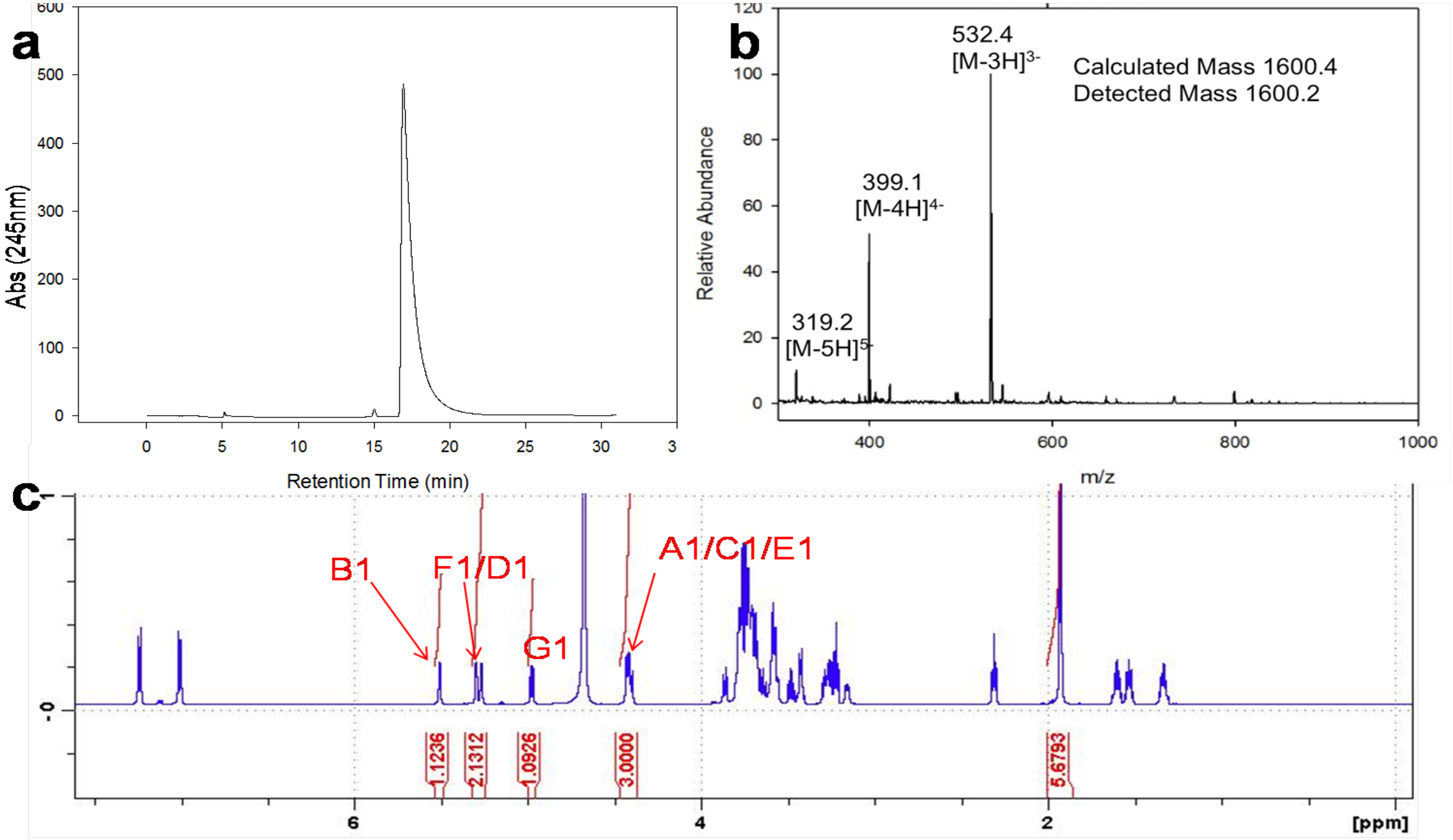

Supplementary Fig. S44. HPLC analysis (a), MS spectrum (b) and ^{1}H NMR spectrum (c) of compound **15**.

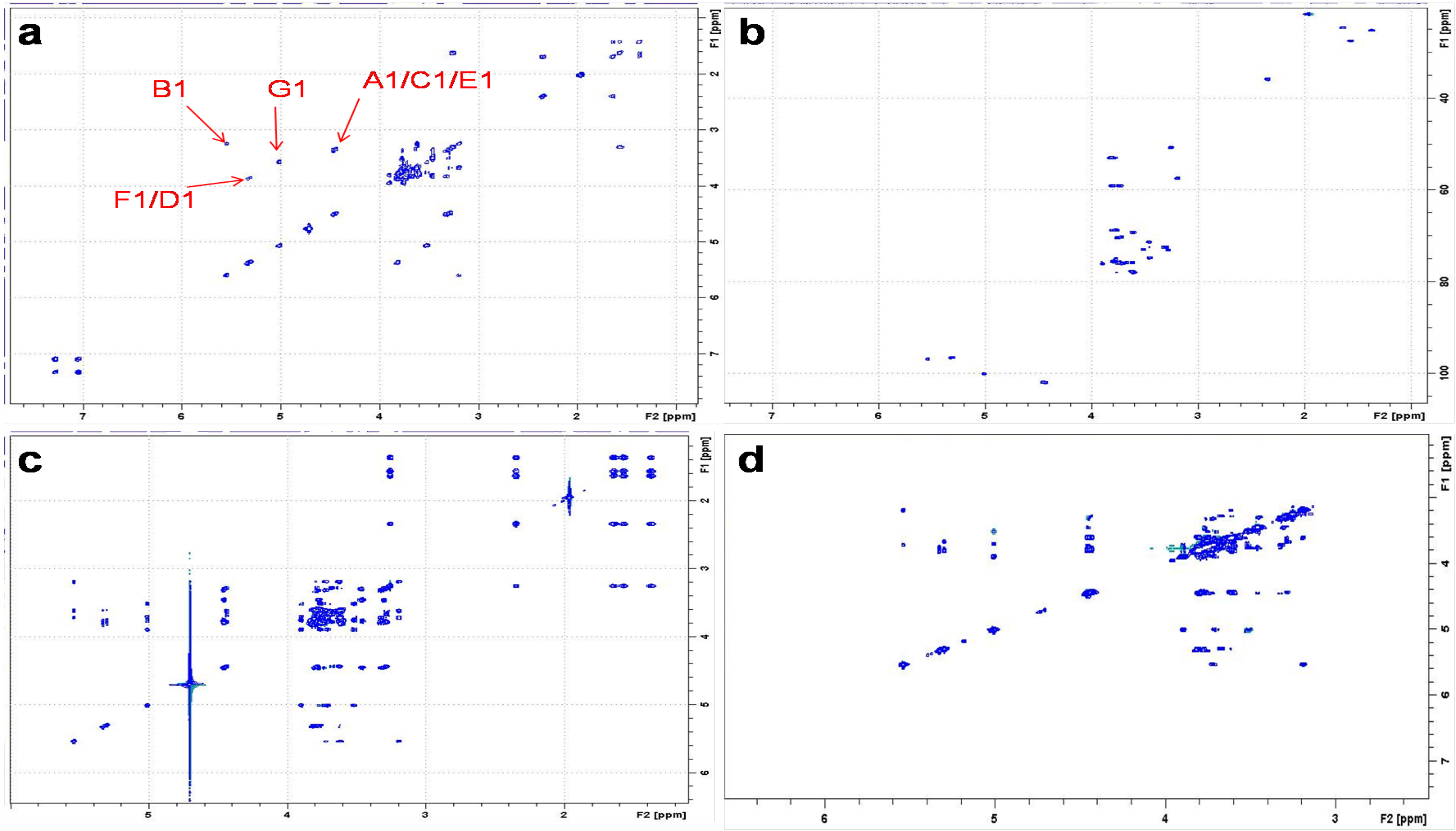

Supplementary Fig. S45. 2D NMR COSY (a), HSQC (b), TOCSY (c) and NOE (d) spectra of compound **15**.

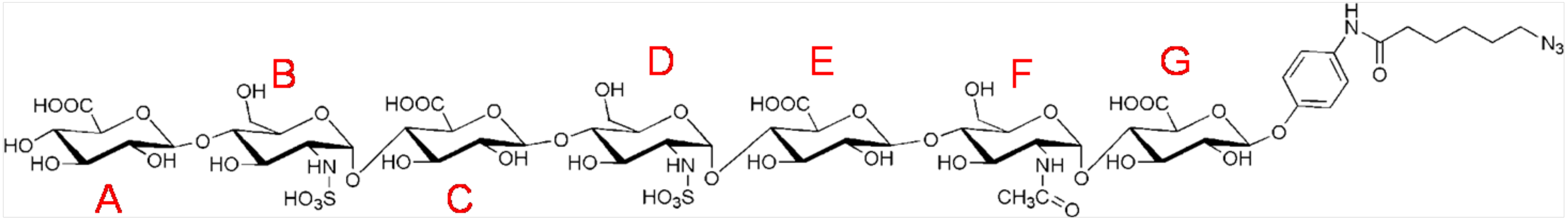

	1	2	3	4	5	6a	6b
A	4.45	3.31	3.46	3.47	3.75	----------	----------
B	5.54	3.20	3.62	3.73	3.79	3.75	3.81
C	4.45	3.31	3.78	3.79	3.79	----------	----------
D	5.54	3.20	3.62	3.73	3.79	3.75	3.81
E	4.45	3.31	3.78	3.79	3.79	----------	----------
F	5.32	3.80	3.75	3.79	3.79	3.62	3.83
G	5.01	3.51	3.72	3.77	3.88	----------	----------

Supplementary Fig. S46. ^{1}H NMR chemical shift assignments (in ppm) of compound **16**. The structure of compound **16** is shown. The protons of *p*NA-N_3 are not listed in the table.

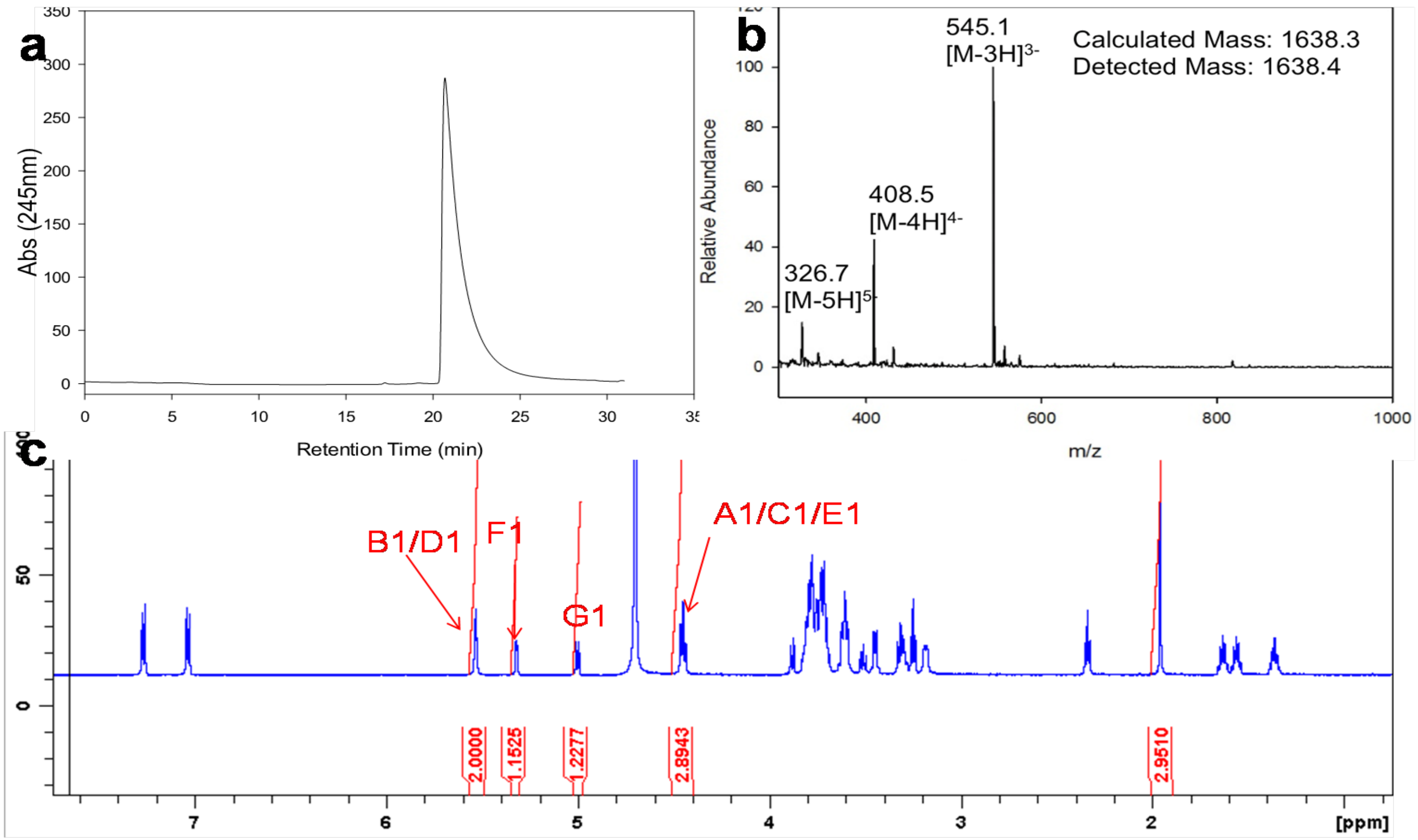

Supplementary Fig. S47. HPLC analysis (a), MS spectrum (b) and ^{1}H NMR spectrum (c) of compound **16**.

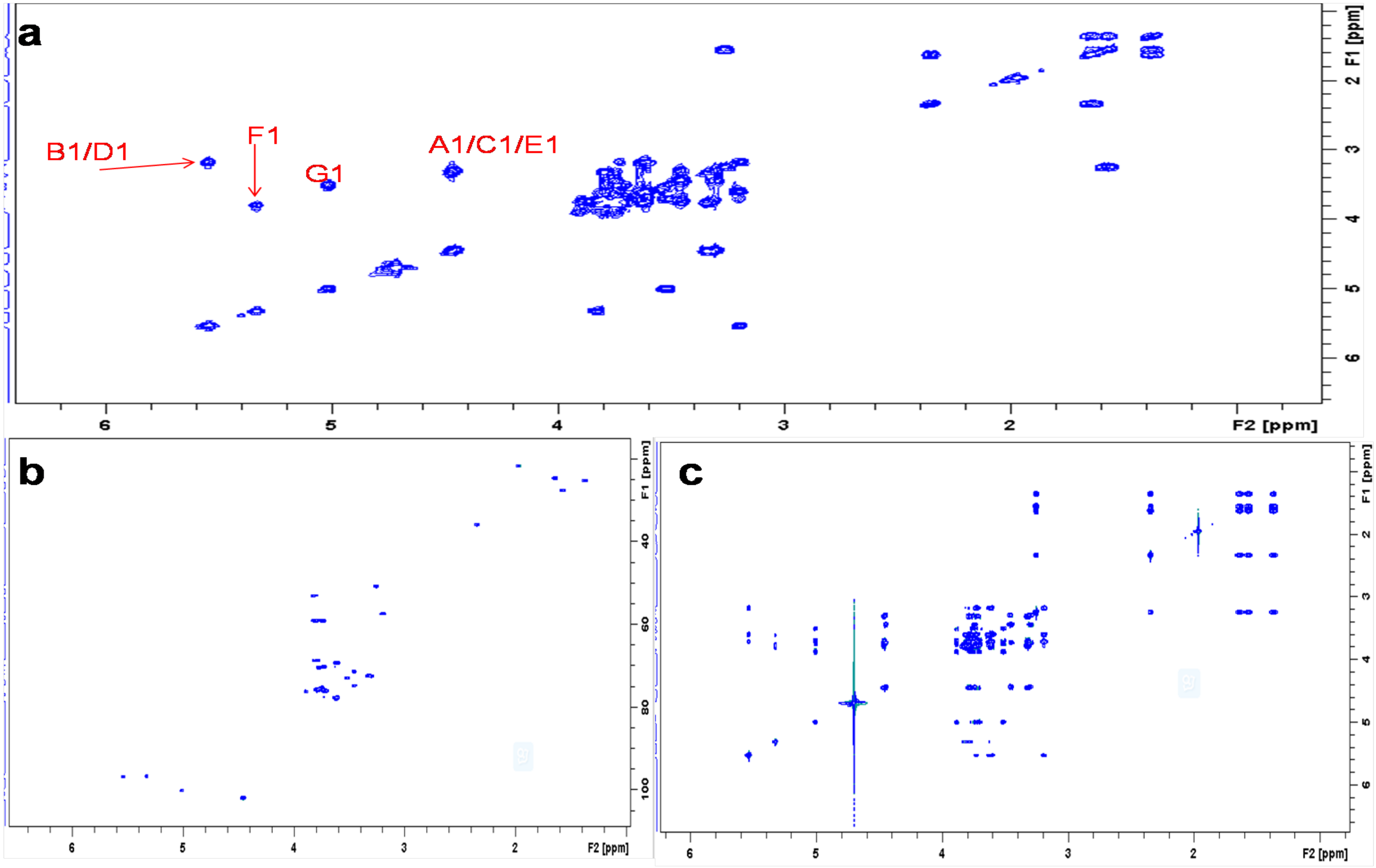

Supplementary Fig. S48. 2D NMR COSY (a), HSQC (b) and TOCSY (c) spectra of compound **16**.

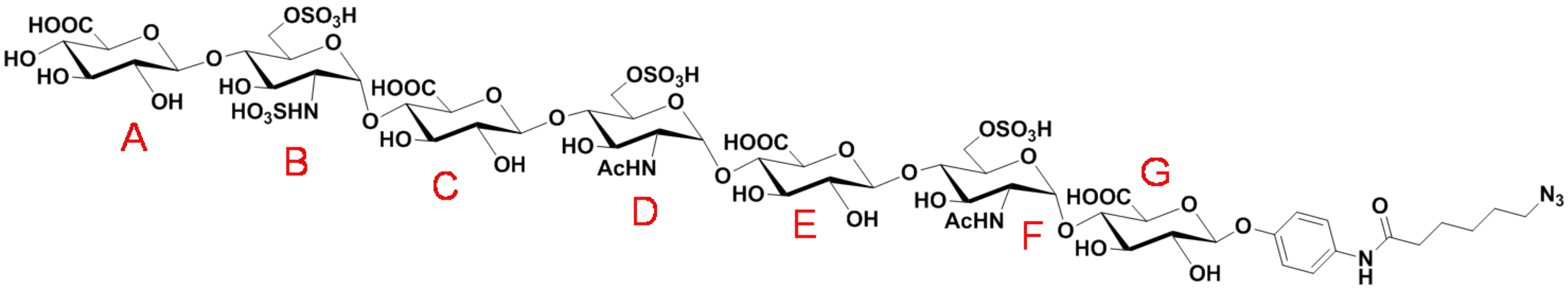

	1	2	3	4	5	6a	6b
A	4.51	3.26	3.46	3.43	3.69	----------	----------
B	5.55	3.21	3.61	3.67	3.94	4.10	4.37
C	4.51	3.30	3.78	3.71	3.74	----------	----------
D	5.33	3.83	3.76	3.65	3.97	4.11	4.37
E	4.50	3.26	3.63	3.68	3.73	----------	----------
F	5.36	3.85	3.78	3.65	3.96	4.11	4.37
G	5.00	3.52	3.70	3.76	3.86	----------	----------

Supplementary Fig. S49. ^{1}H NMR chemical shift assignments (in ppm) of compound **17**. The structure of compound **17** is shown. The protons of *p*NA-N_3 are not listed in the table.

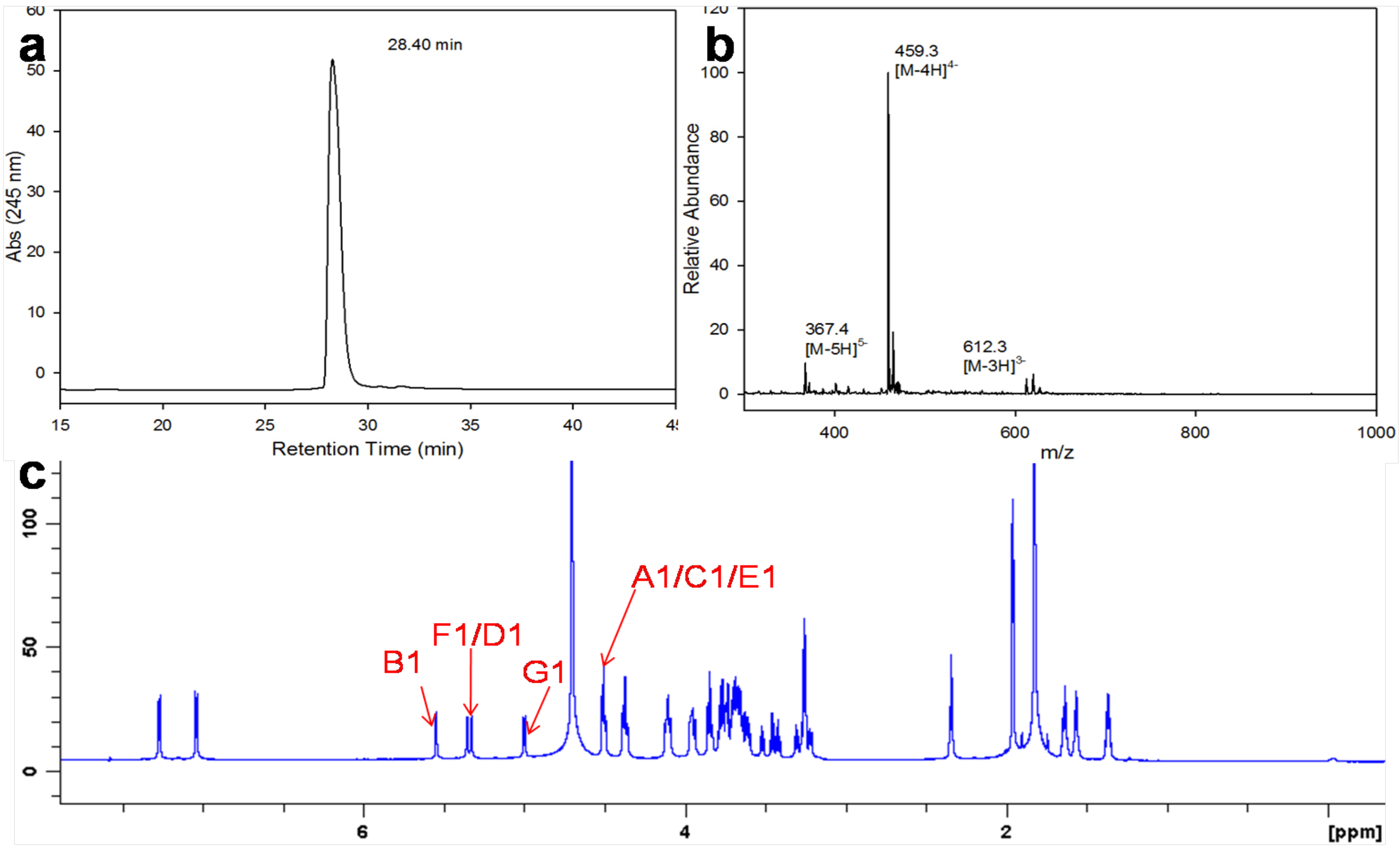

Supplementary Fig. S50. HPLC analysis (a), MS spectrum (b) and ^{1}H NMR spectrum (c) of compound **17**.

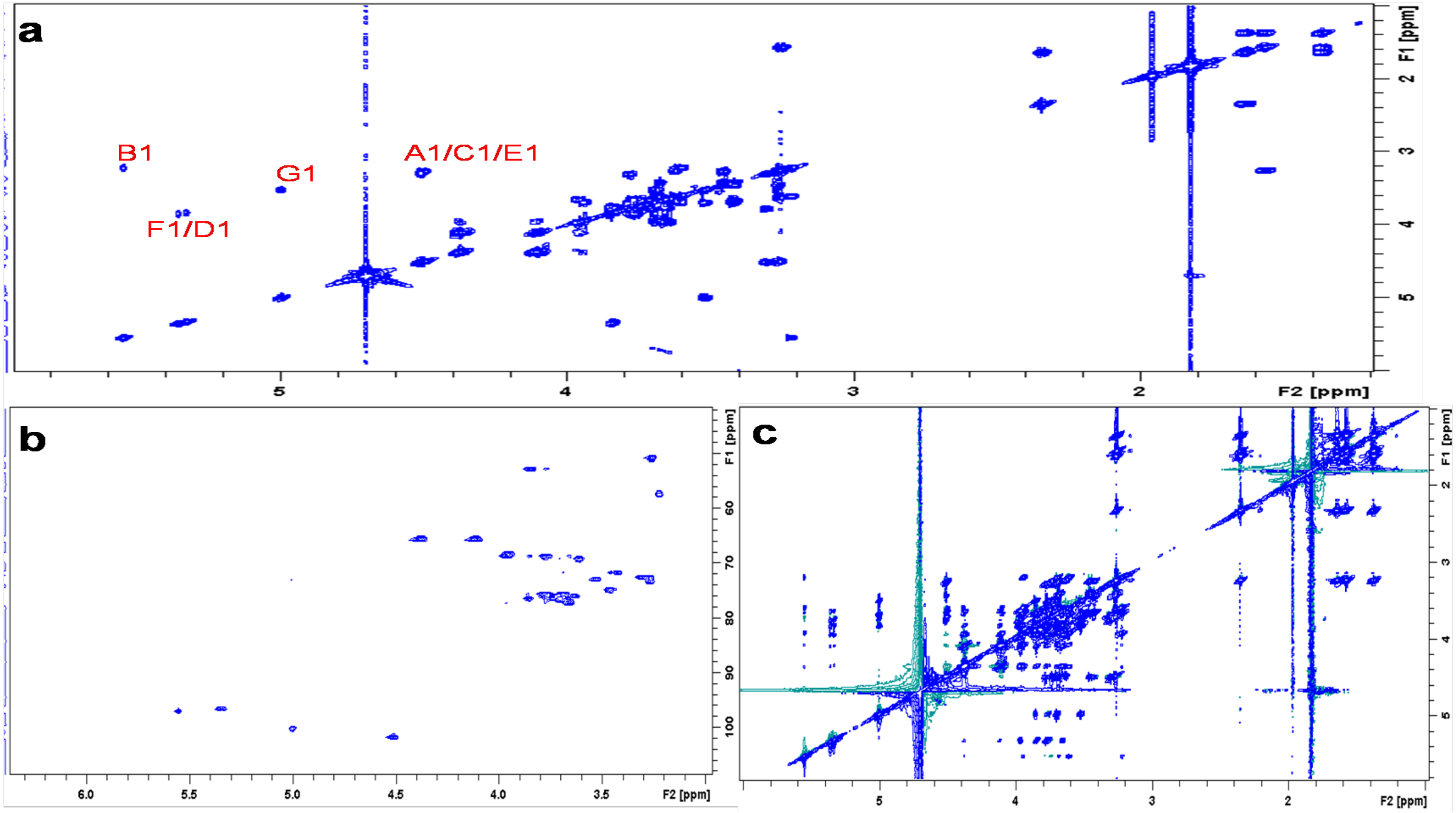

Supplementary Fig. S51. 2D NMR COSY (a), HSQC (b) and TOCSY (c) spectra of compound **17**.

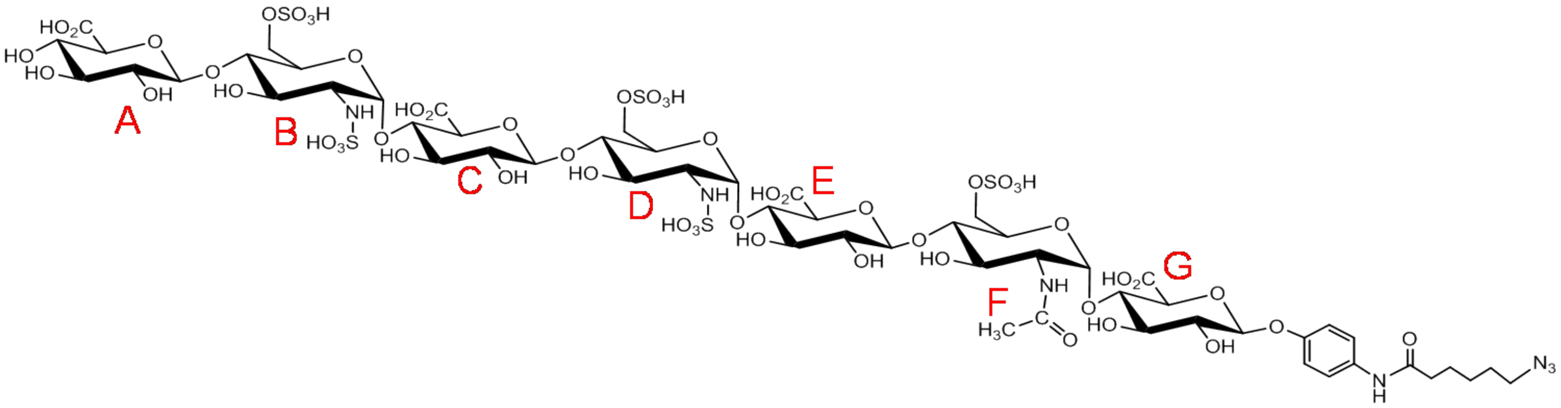

	1	2	3	4	5	6a	6b
A	4.53	3.27	3.45	3.46	3.78	----------	----------
B	5.51	3.21	3.60	3.66	3.87	4.11	4.35
C	4.55	3.31	3.79	3.76	3.92	----------	----------
D	5.51	3.21	3.60	3.66	3.87	4.11	4.35
E	4.55	3.31	3.79	3.76	3.92	----------	----------
F	5.32	3.86	3.77	3.66	3.92	4.13	4.36
G	5.02	3.52	3.71	3.78	3.97	----------	----------

Supplementary Fig. S52. ^{1}H NMR chemical shift assignments (in ppm) of compound **18**. The structure of compound **18** is shown. The protons of *p*NA-N_3 are not listed in the table.

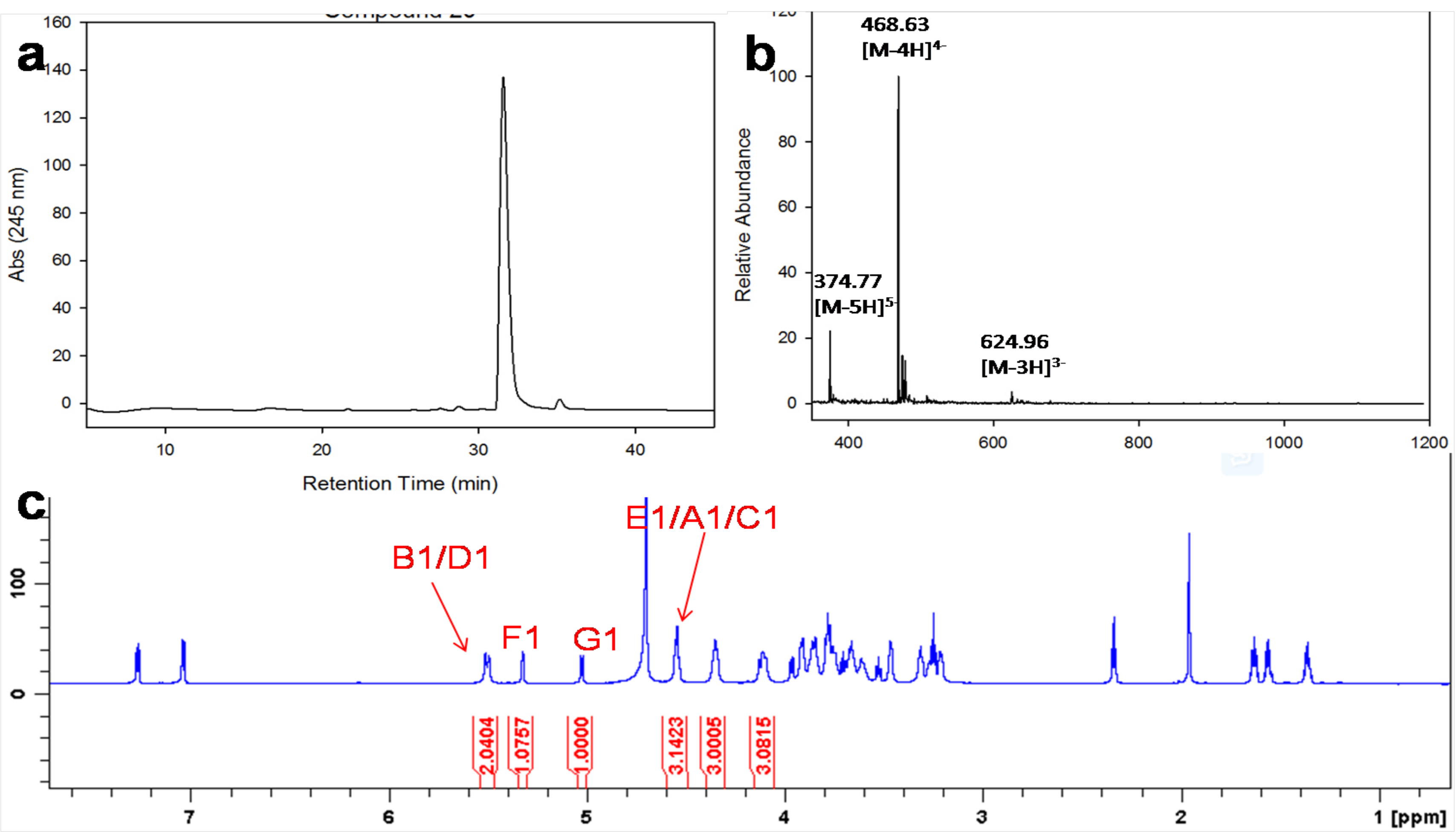

Supplementary Fig. S53. HPLC analysis (a), MS spectrum (b) and ^{1}H NMR spectrum (c) of compound **18**.

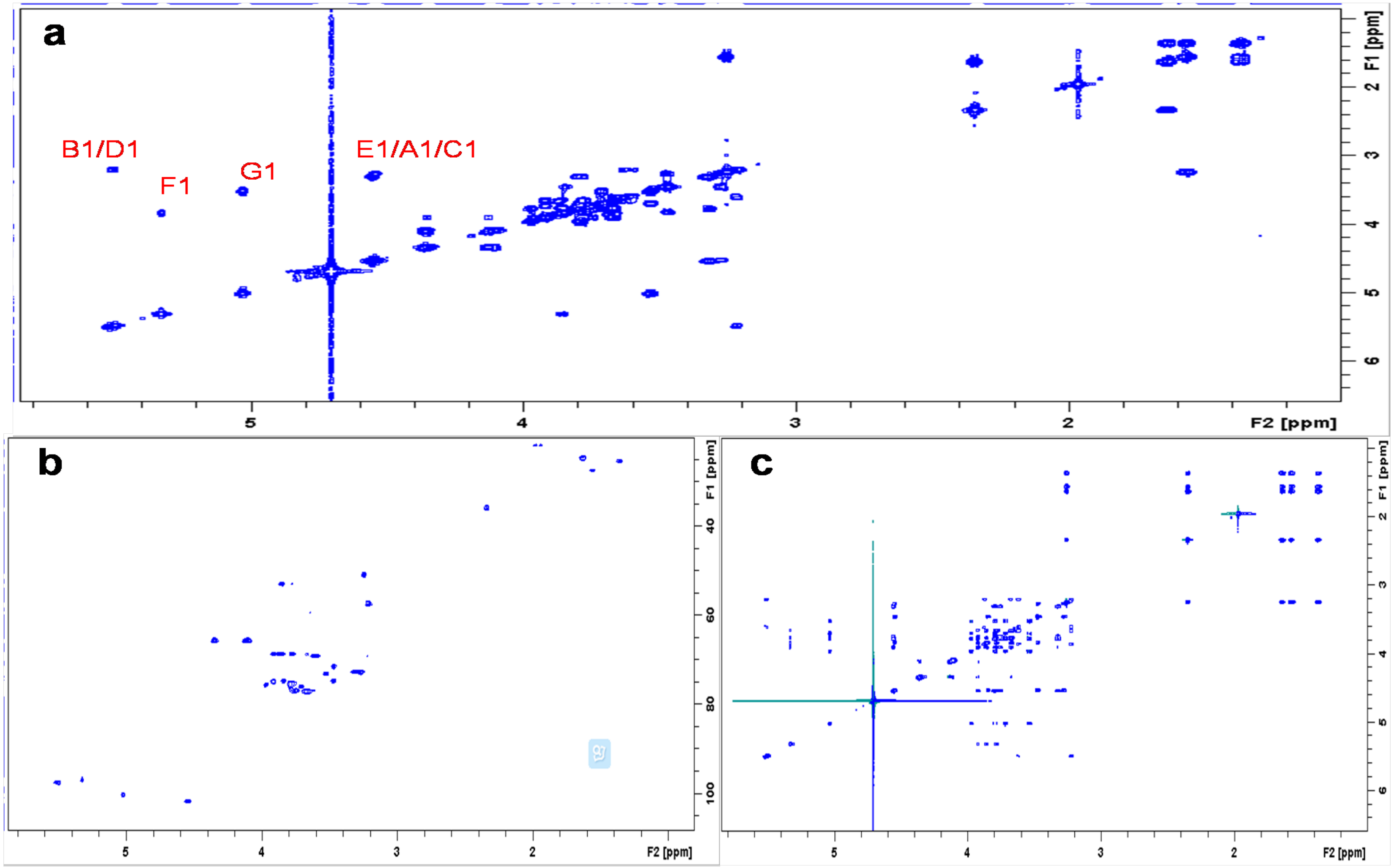

Supplementary Fig. S54. 2D NMR COSY (a), HSQC (b) and TOCSY (c) spectra of compound **18**.

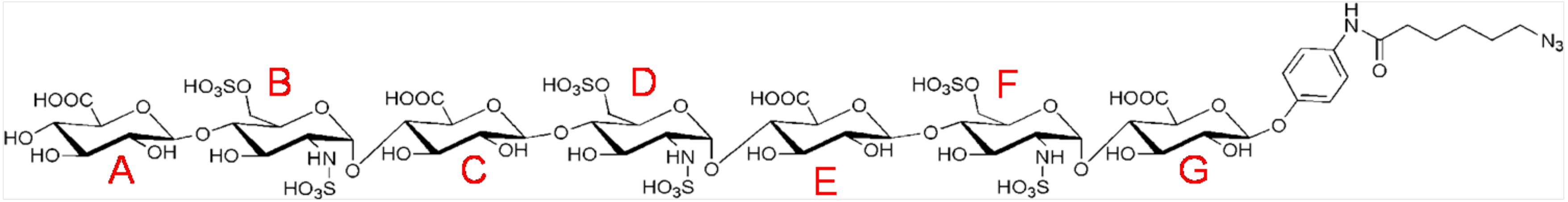

	1	2	3	4	5	6a	6b
A	4.45	3.19	3.38	3.39	3.75	----------	----------
B	5.44	3.15	3.54	3.60	3.82	4.06	4.30
C	4.48	3.23	3.71	3.70	3.80	----------	----------
D	5.44	3.15	3.54	3.60	3.82	4.06	4.30
E	4.48	3.23	3.71	3.70	3.80	----------	----------
F	5.49	3.16	3.55	3.62	3.82	4.06	4.30
G	5.00	3.49	3.80	3.75	3.91	----------	----------

Supplementary Fig. S55. ^{1}H NMR chemical shift assignments (in ppm) of compound **19**. The structure of compound **19** is shown. The protons of *p*NA-N_3 are not listed in the table.

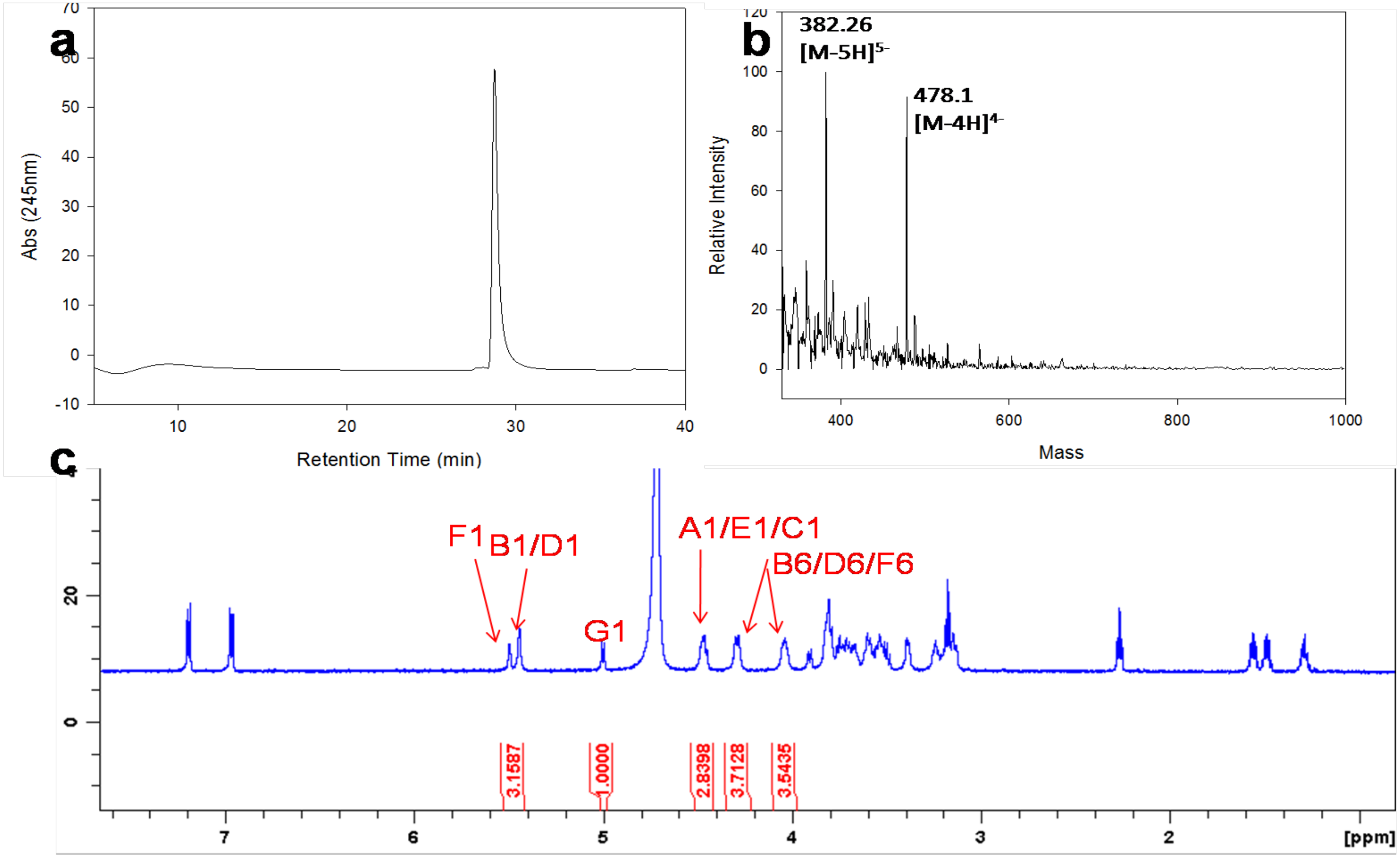

Supplementary Fig. S56. HPLC analysis (a), MS spectrum (b) and ^{1}H NMR spectrum (c) of compound **19**.

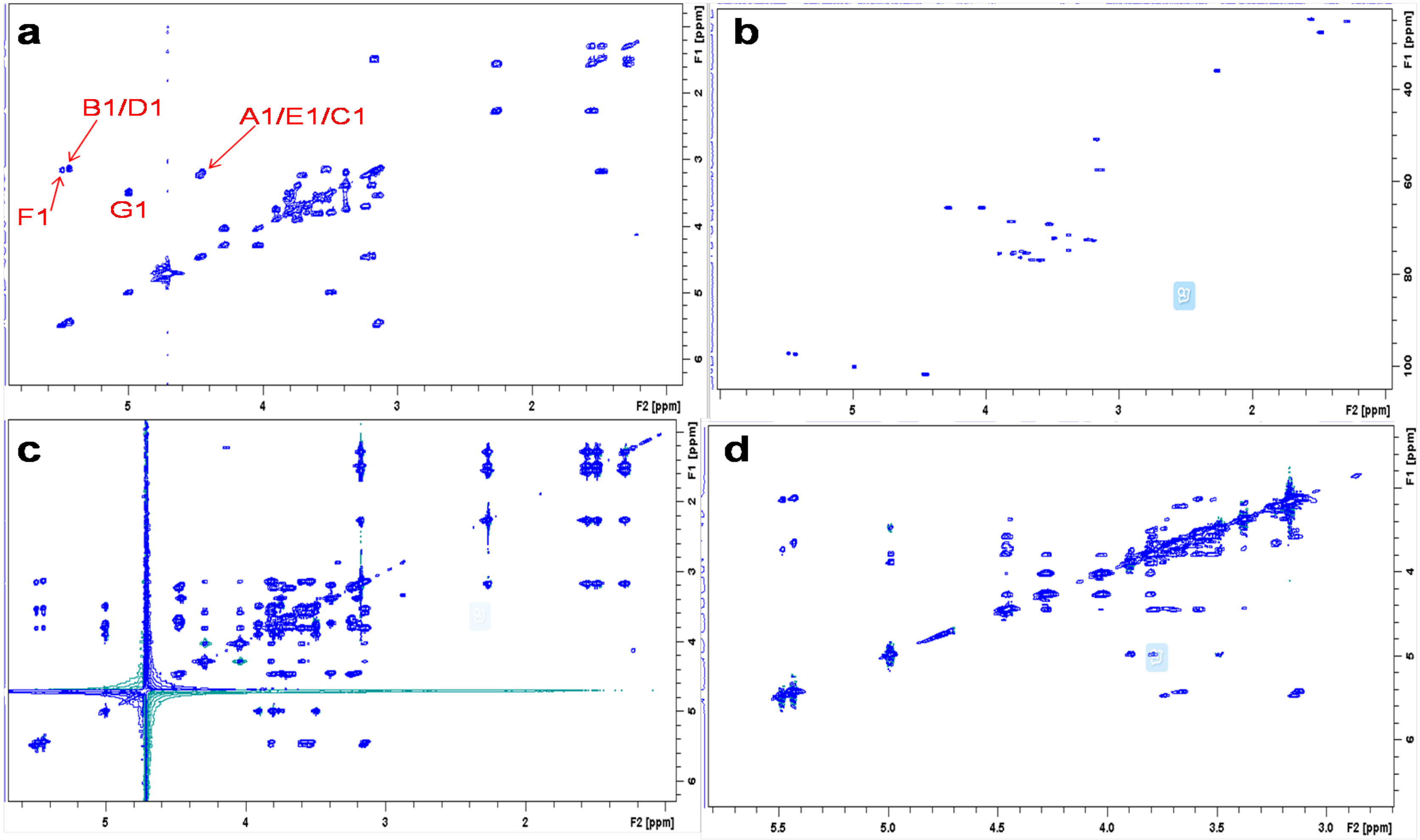

Supplementary Fig. S57. 2D NMR COSY (a), HSQC (b), TOCSY (c) and NOE (d) spectra of compound **19**.

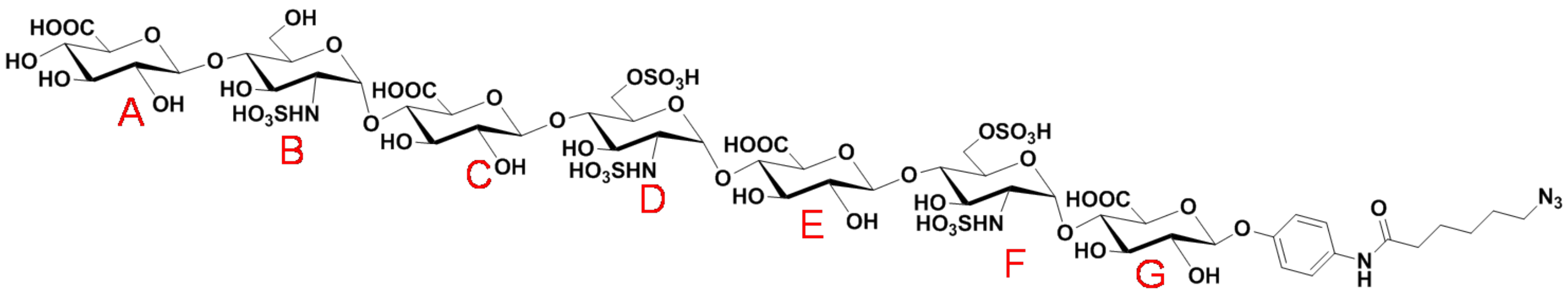

	1	2	3	4	5	6a	6b
A	4.43	3.30	3.44	3.45	3.67	----------	----------
B	5.52	3.18	3.61	3.75	3.77	3.72	3.79
C	4.52	3.29	3.78	3.74	3.74	----------	----------
D	5.54	3.21	3.60	3.65	3.92	4.11	4.36
E	4.52	3.29	3.78	3.74	3.74	----------	----------
F	5.59	3.23	3.60	3.66	3.94	4.10	4.36
G	5.04	3.55	3.86	3.79	3.87	----------	----------

Supplementary Fig. S58. ^{1}H NMR chemical shift assignments (in ppm) of compound **20**. The structure of compound **20** is shown. The protons of *p*NA-N_3 are not listed in the table.

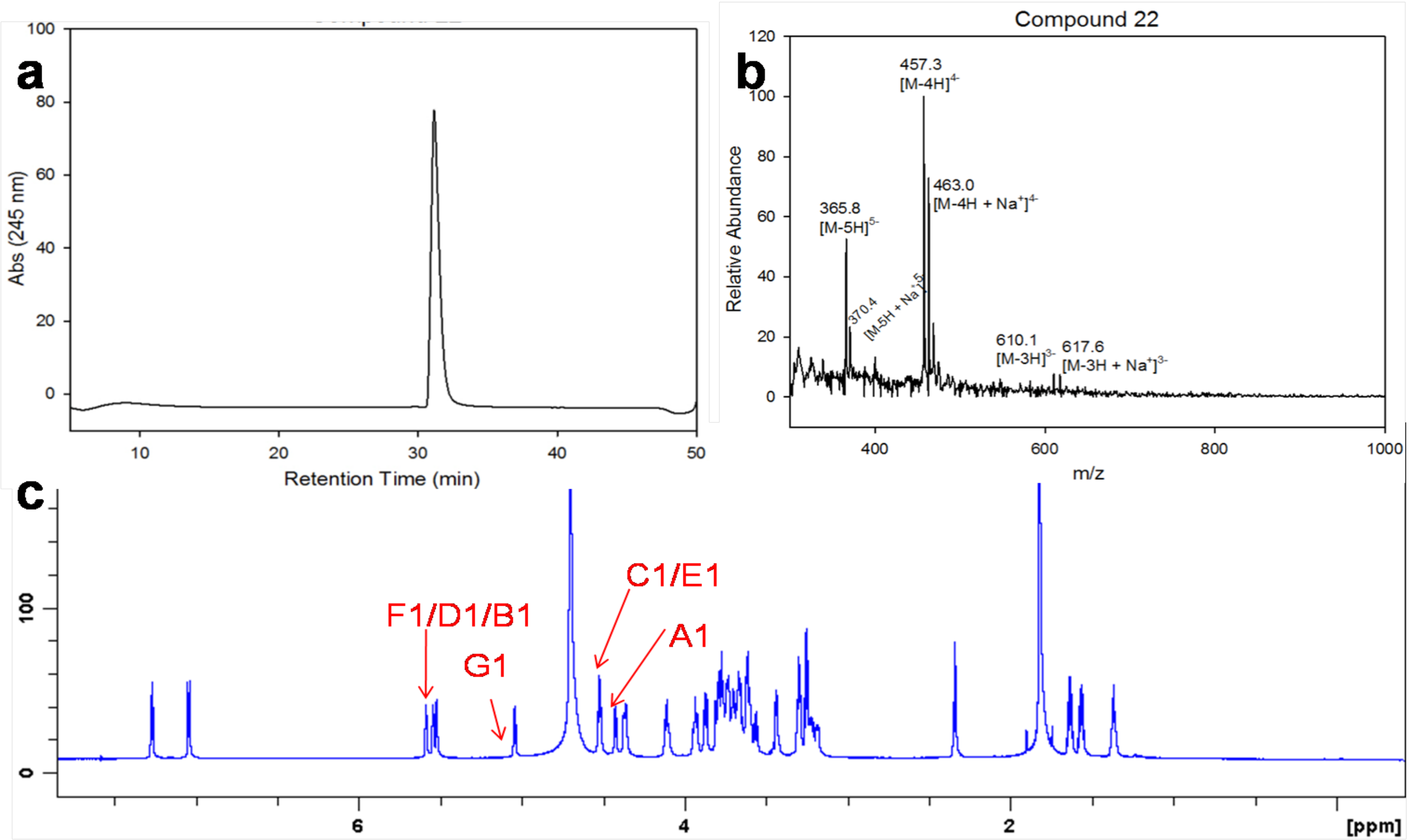

Supplementary Fig. S59. HPLC analysis (a), MS spectrum (b) and ^{1}H NMR spectrum (c) of compound **20**.

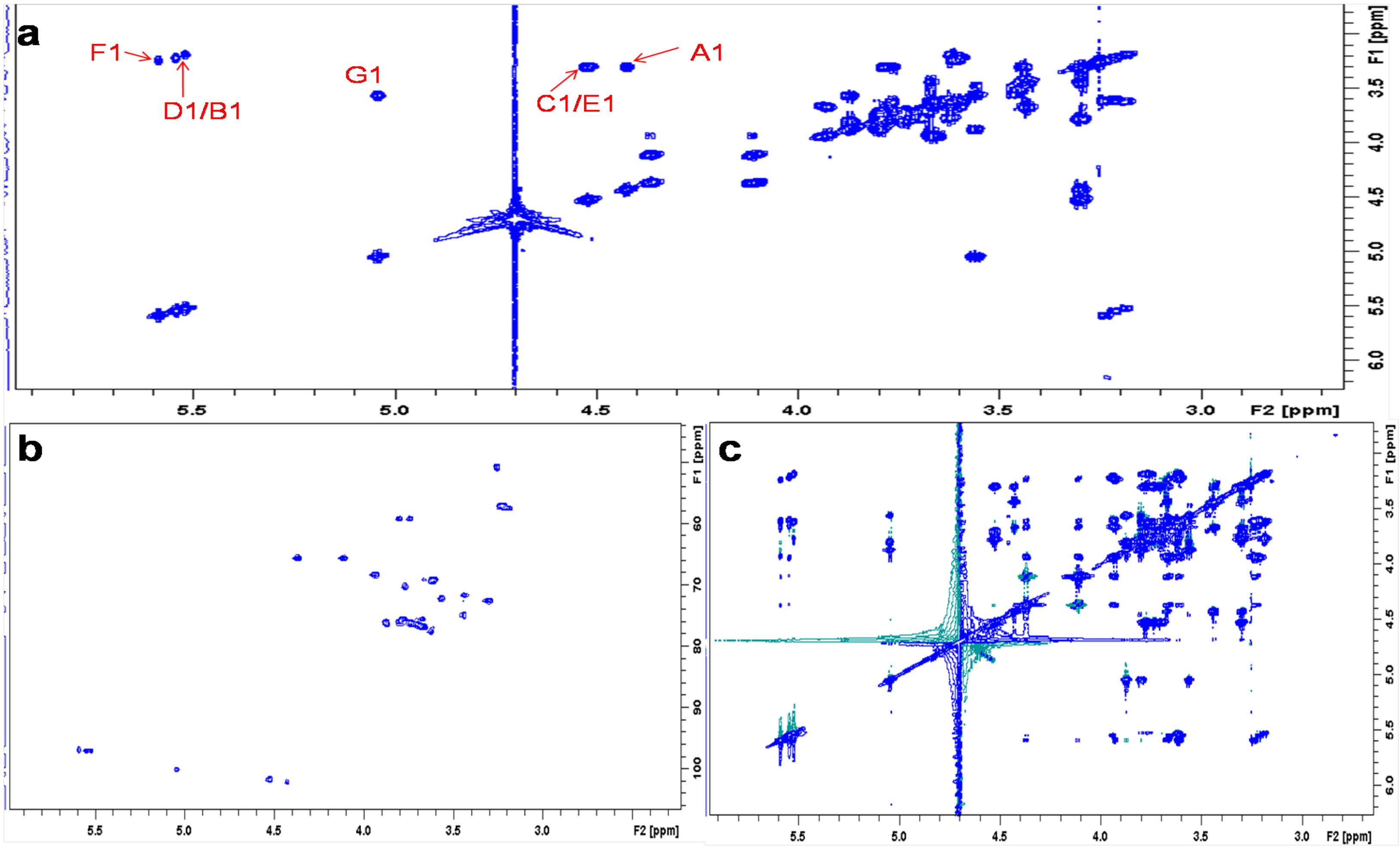

Supplementary Fig. S60. 2D NMR COSY (a), HSQC (b) and TOCSY (c) spectra of compound **20**.

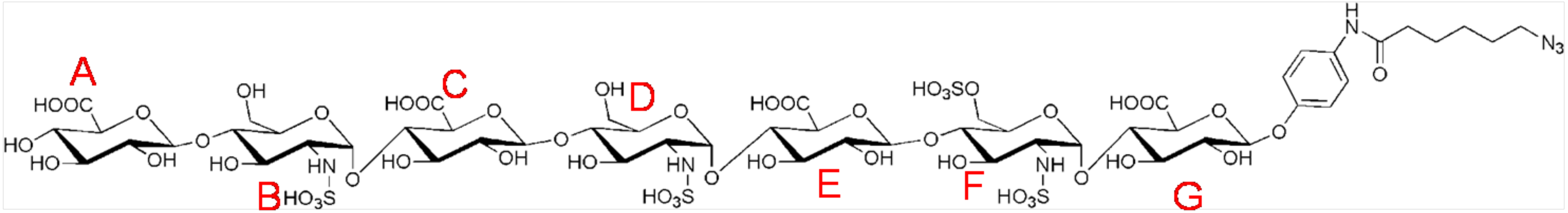

	1	2	3	4	5	6a	6b
A	4.44	3.31	3.45	3.46	3.69	----------	----------
B	5.55	3.20	3.62	3.71	3.78	3.75	3.82
C	4.46	3.32	3.75	3.78	3.78	----------	----------
D	5.55	3.20	3.62	3.71	3.78	3.75	3.82
E	4.53	3.31	3.79	3.72	3.75	----------	----------
F	5.61	3.24	3.62	3.68	3.94	4.13	4.38
G	5.05	3.57	3.85	3.79	3.89	----------	----------

Supplementary Fig. S61. ^{1}H NMR chemical shift assignments (in ppm) of compound **21**. The structure of compound **21** is shown. The protons of *p*NA-N_3 are not listed in the table.

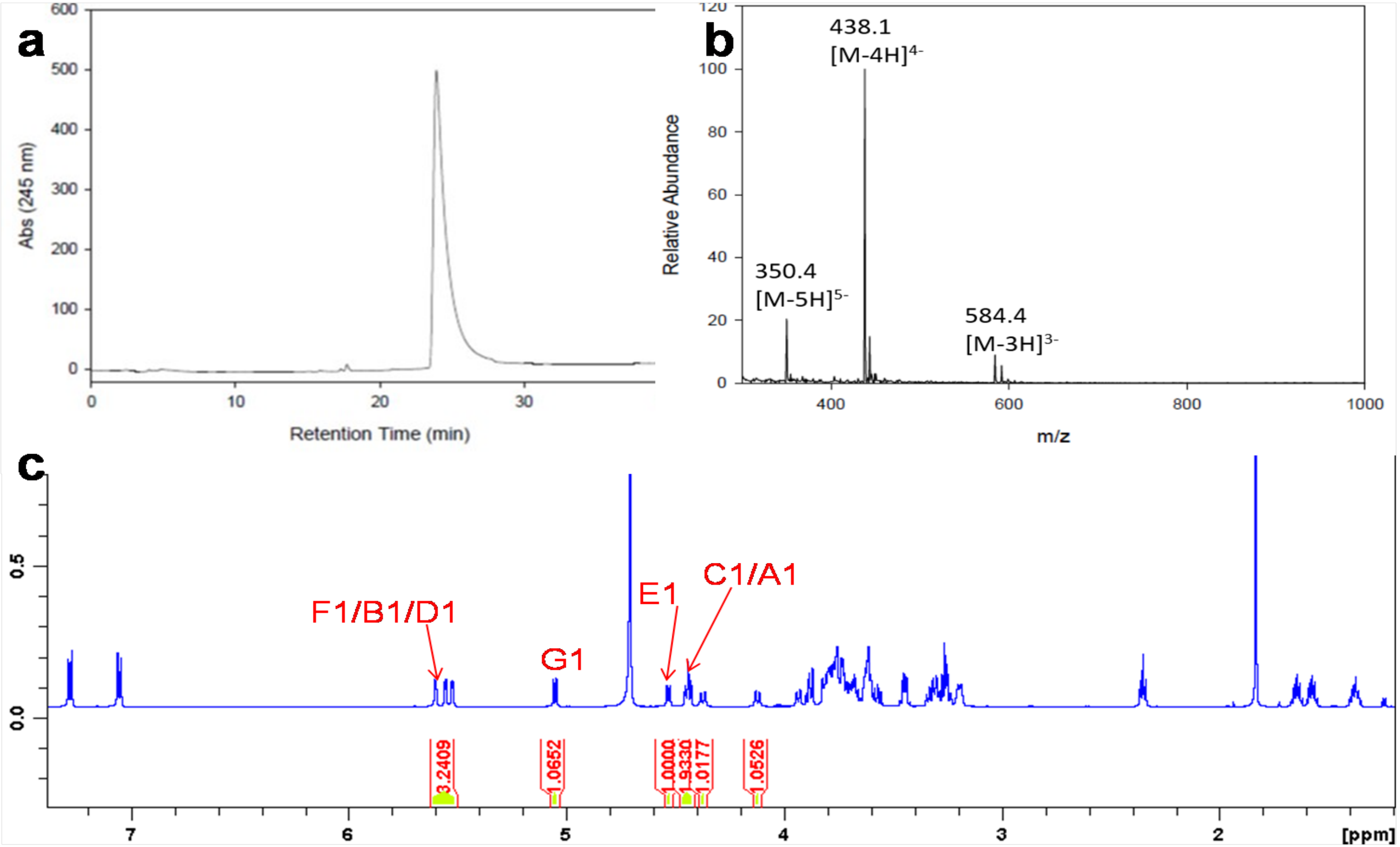

Supplementary Fig. S62. HPLC analysis (a), MS spectrum (b) and ^{1}H NMR spectrum (c) of compound **21**.

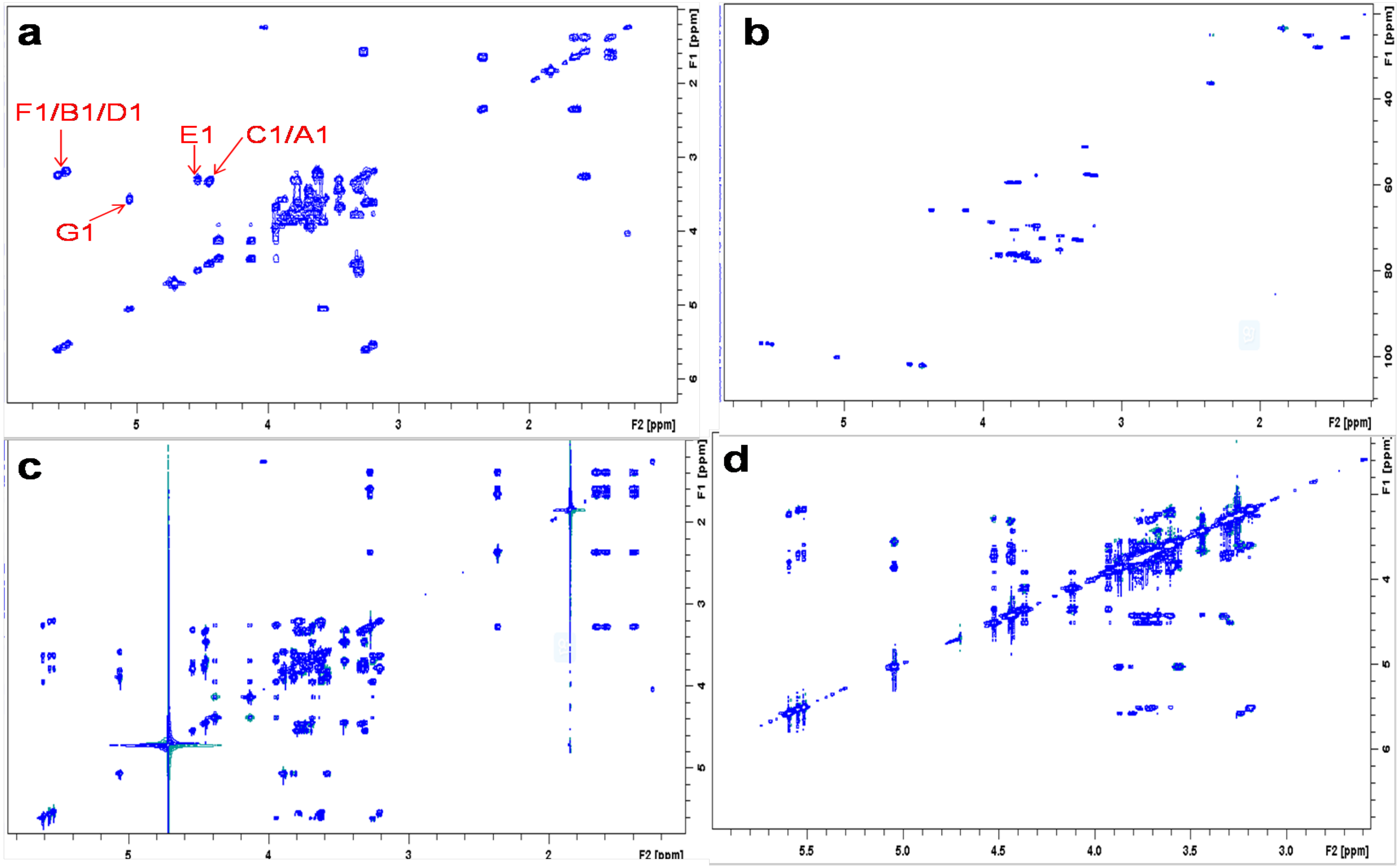

Supplementary Fig. S63. 2D NMR COSY (a), HSQC (b), TOCSY (c) and NOE (d) spectra of compound **21**.

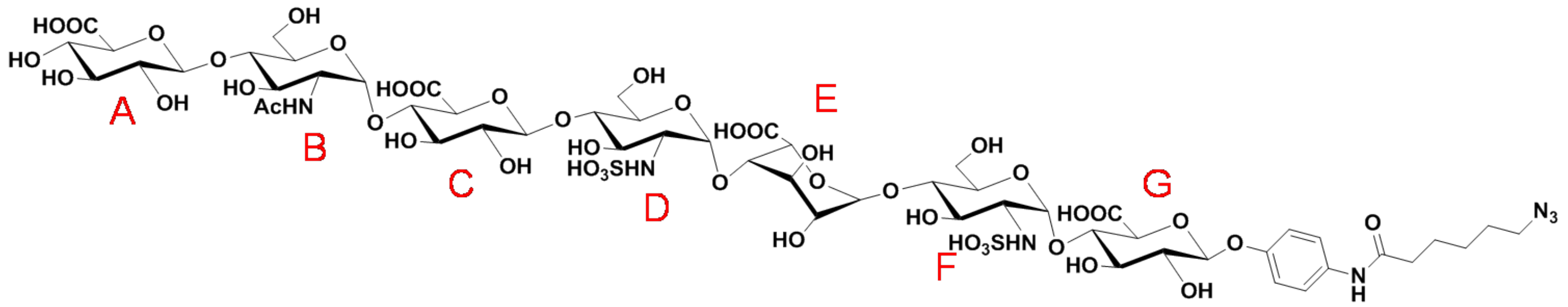

	1	2	3	4	5	6a	6b
A	4.42	3.28	3.44	3.67	3.72	----------	----------
B	5.31	3.80	3.75	3.78	3.76	3.61	3.76
C	4.42	3.29	3.61	3.65	3.72	----------	----------
D	5.28	3.14	3.61	3.78	3.75	3.72	3.85
E	4.87	3.64	4.02	3.96	4.69	----------	----------
F	5.53	3.19	3.55	3.63	3.72	3.74	3.82
G	5.04	3.55	3.86	3.78	3.87	----------	----------

Supplementary Fig. S64. ^{1}H NMR chemical shift assignments (in ppm) of compound **22**. The structure of compound **22** is shown. The protons of *p*NA-N_3 are not listed in the table.

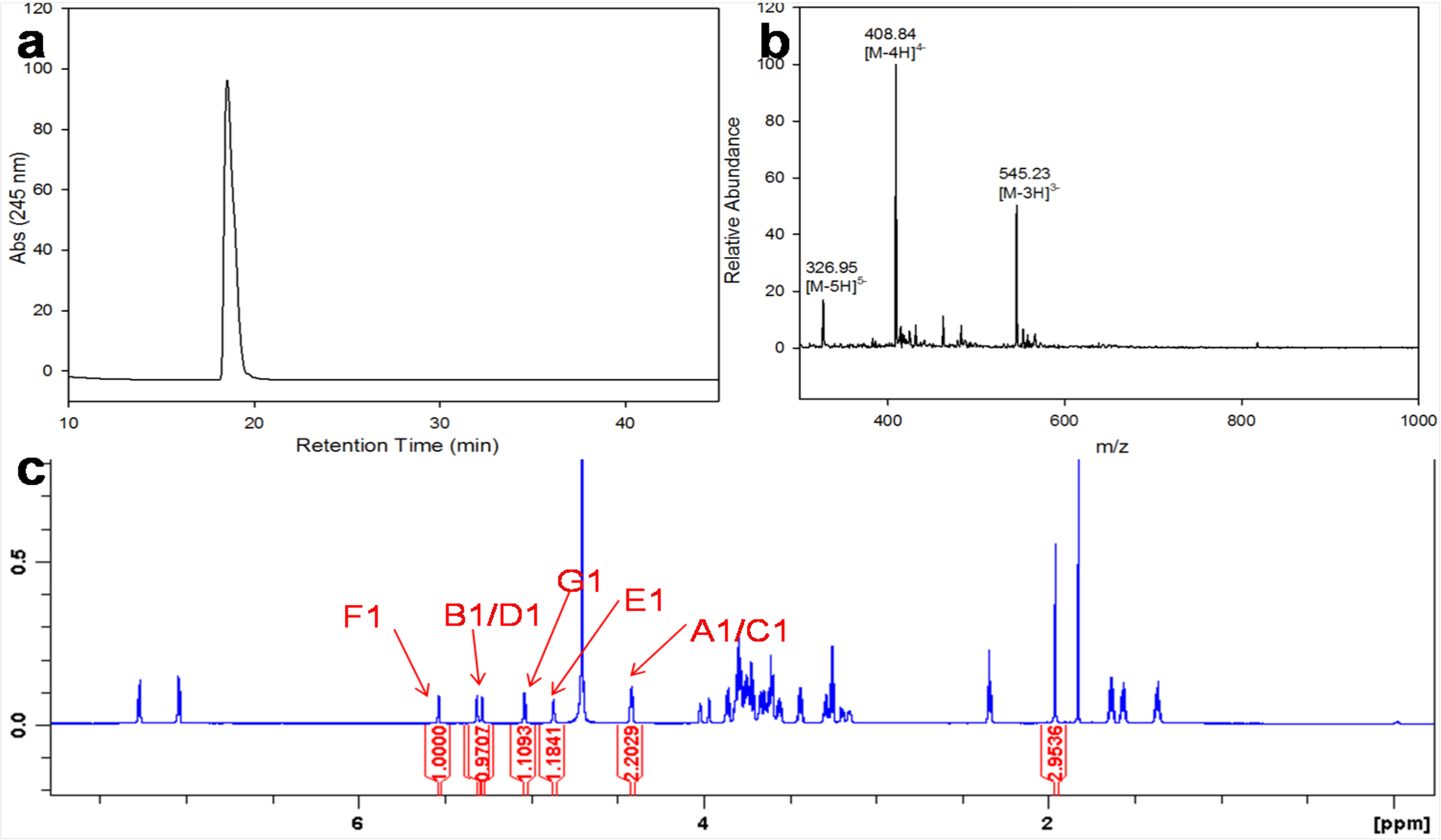

Supplementary Fig. S65. HPLC analysis (a), MS spectrum (b) and ^{1}H NMR spectrum (c) of compound **22**.

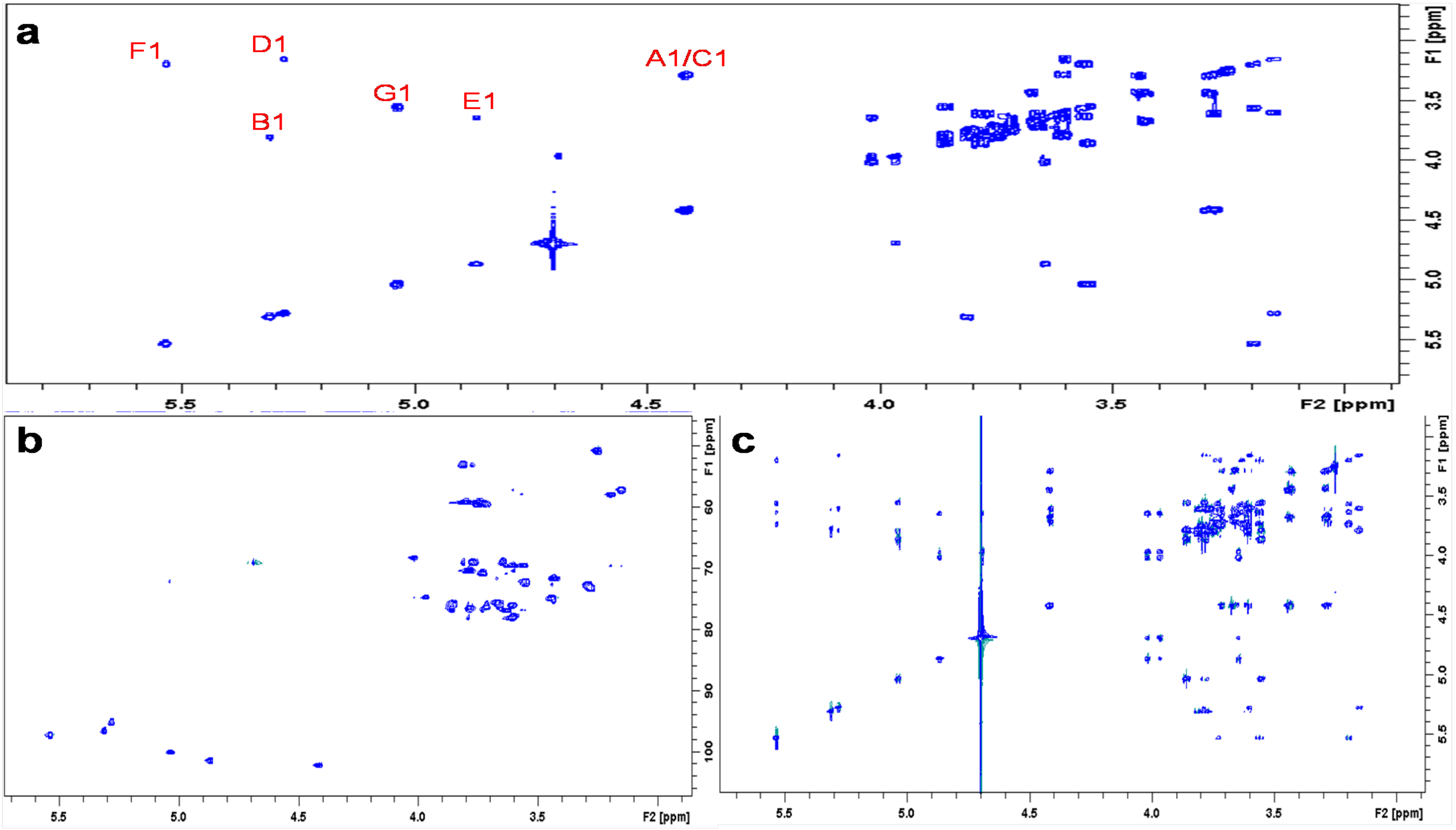

Supplementary Fig. S66. 2D NMR COSY (a), HSQC (b) and TOCSY (c) spectra of compound **22**.

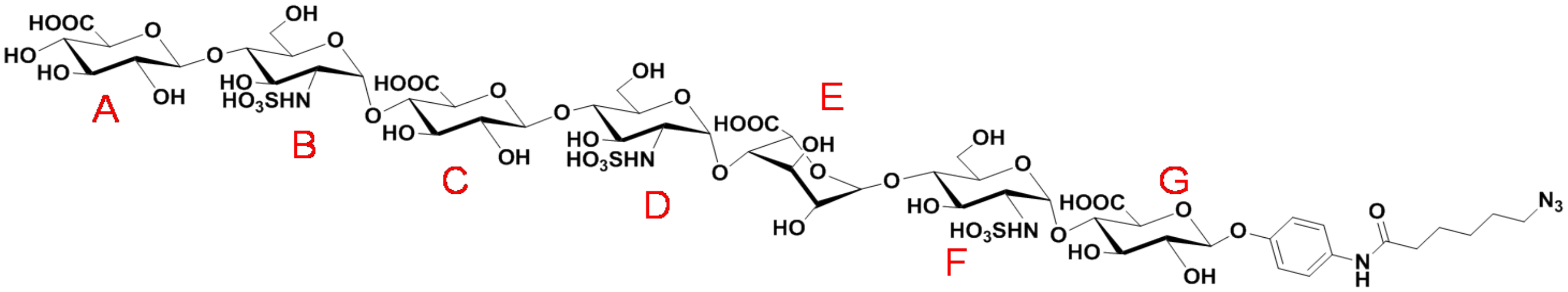

	1	2	3	4	5	6a	6b
A	4.45	3.33	3.48	3.65	3.70	----------	----------
B	5.55	3.22	3.61	3.63	3.72	3.72	3.75
C	4.48	3.33	3.76	3.64	3.81	----------	----------
D	5.29	3.18	3.65	3.72	3.79	3.72	3.80
E	4.91	3.67	4.03	3.97	4.73	----------	----------
F	5.55	3.22	3.61	3.63	3.72	3.72	3.75
G	5.05	3.57	3.86	3.78	3.88	----------	----------

Supplementary Fig. S67. ^{1}H NMR chemical shift assignments (in ppm) of compound **23**. The structure of compound **23** is shown. The protons of *p*NA-N_3 are not listed in the table.

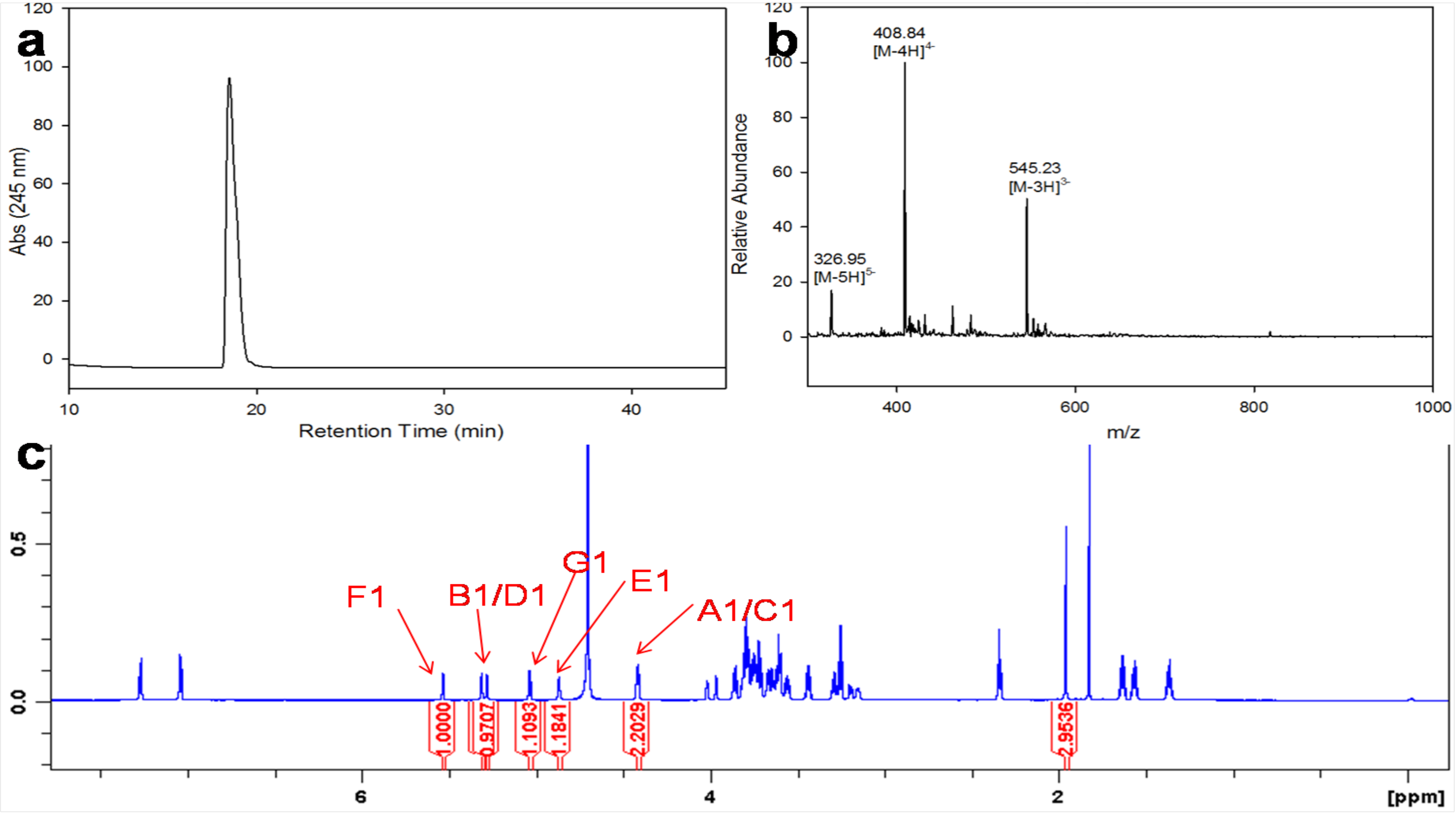

Supplementary Fig. S68. HPLC analysis (a), MS spectrum (b) and ^{1}H NMR spectrum (c) of compound **23**.

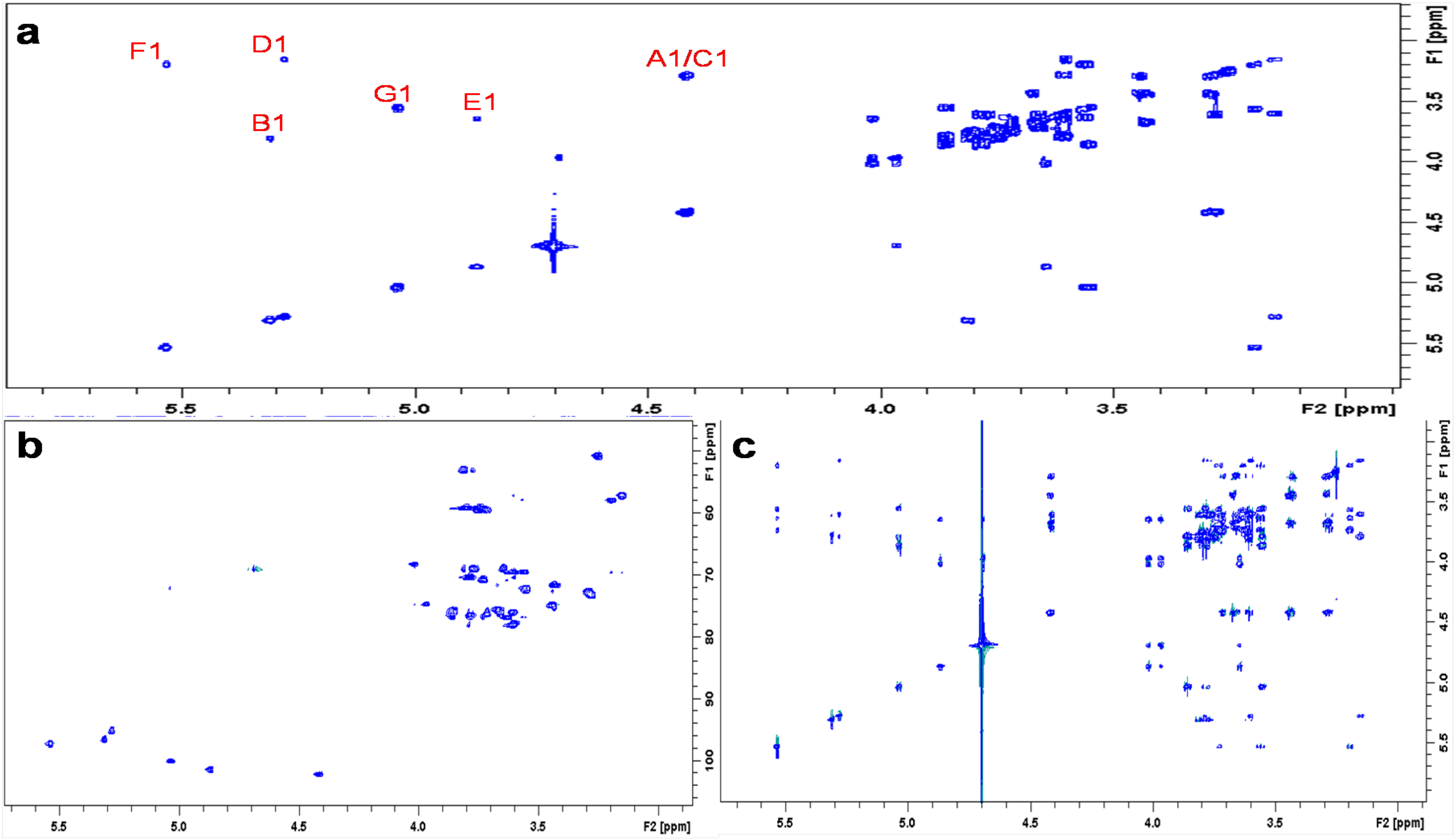

Supplementary Fig. S69. 2D NMR COSY (a), HSQC (b) and TOCSY (c) spectra of compound **23.**

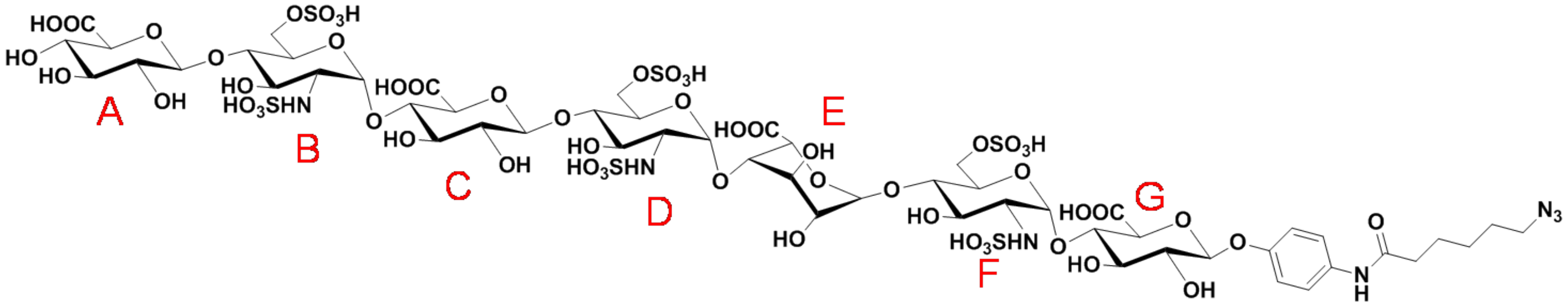

	1	2	3	4	5	6a	6b
A	4.52	3.27	3.45	3.43	3.69	----------	----------
B	5.56	3.22	3.62	3.69	3.96	4.12	4.37
C	4.54	3.30	3.79	3.71	3.75	----------	----------
D	5.26	3.19	3.65	3.72	3.92	4.12	4.24
E	4.98	3.73	4.05	3.99	4.78	----------	----------
F	5.56	3.22	3.62	3.69	3.96	4.12	4.37
G	5.05	3.58	3.87	3.81	3.88	----------	----------

Supplementary Fig. S70. ^{1}H NMR chemical shift assignments (in ppm) of compound **24**. The structure of compound **24** is shown. The protons of *p*NA-N_3 are not listed in the table.

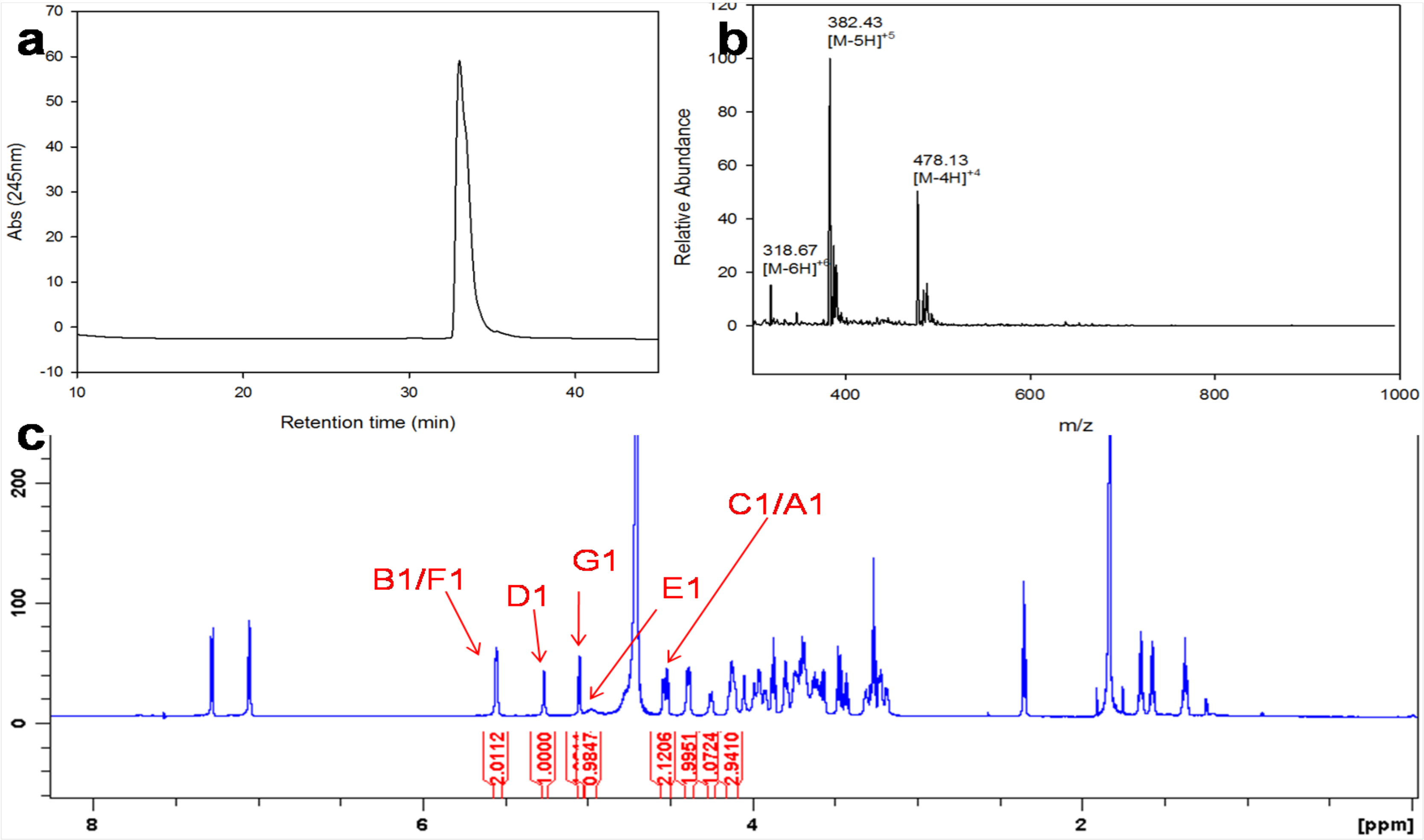

Supplementary Fig. S71. HPLC analysis (a), MS spectrum (b) and ^{1}H NMR spectrum (c) of compound **24**.

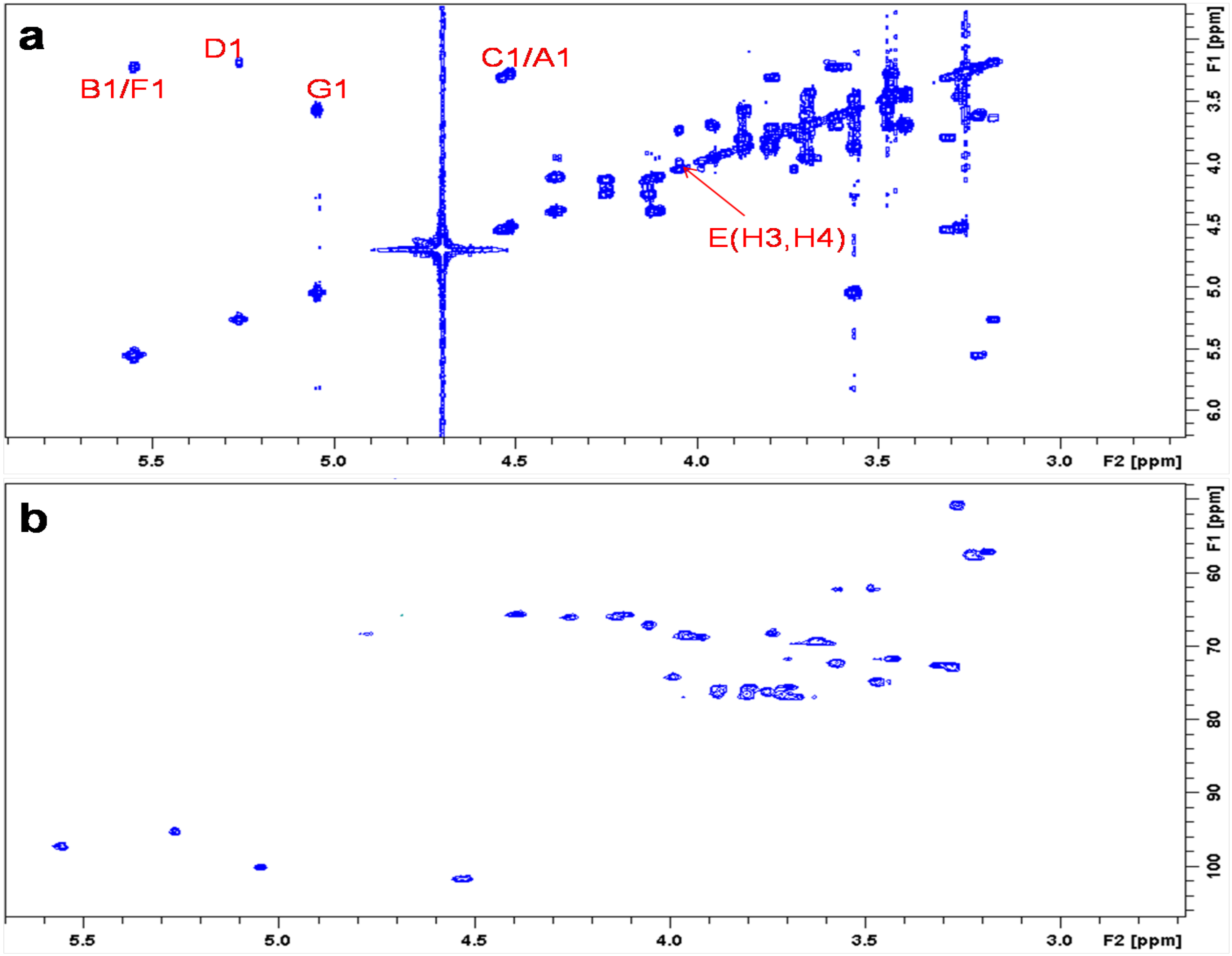

Supplementary Fig. S72. 2D NMR COSY (a) and HSQC (b) spectra of compound **24.**

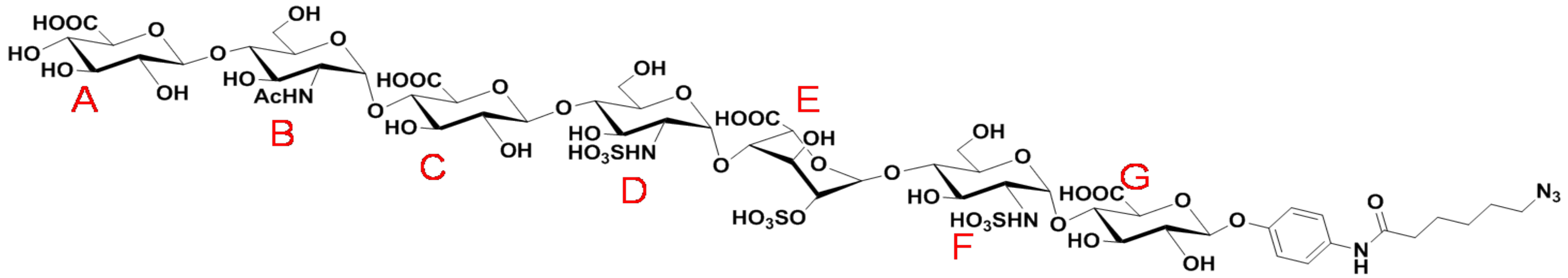

	1	2	3	4	5	6a	6b
A	4.43	3.31	3.45	3.45	3.69	----------	----------
B	5.32	3.83	3.62	3.72	3.74	3.74	3.80
C	4.43	3.31	3.63	3.67	3.74	----------	----------
D	5.23	3.19	3.62	3.65	3.80	3.75	3.85
E	5.19	4.26	4.16	3.97	4.78	----------	----------
F	5.52	3.20	3.61	3.65	3.67	3.65	3.74
G	5.05	3.58	3.86	3.81	3.89	----------	----------

Supplementary Fig. S73. ^{1}H NMR chemical shift assignments (in ppm) of compound **25**. The structure of compound **25** is shown. The protons of *p*NA-N_3 are not listed in the table.

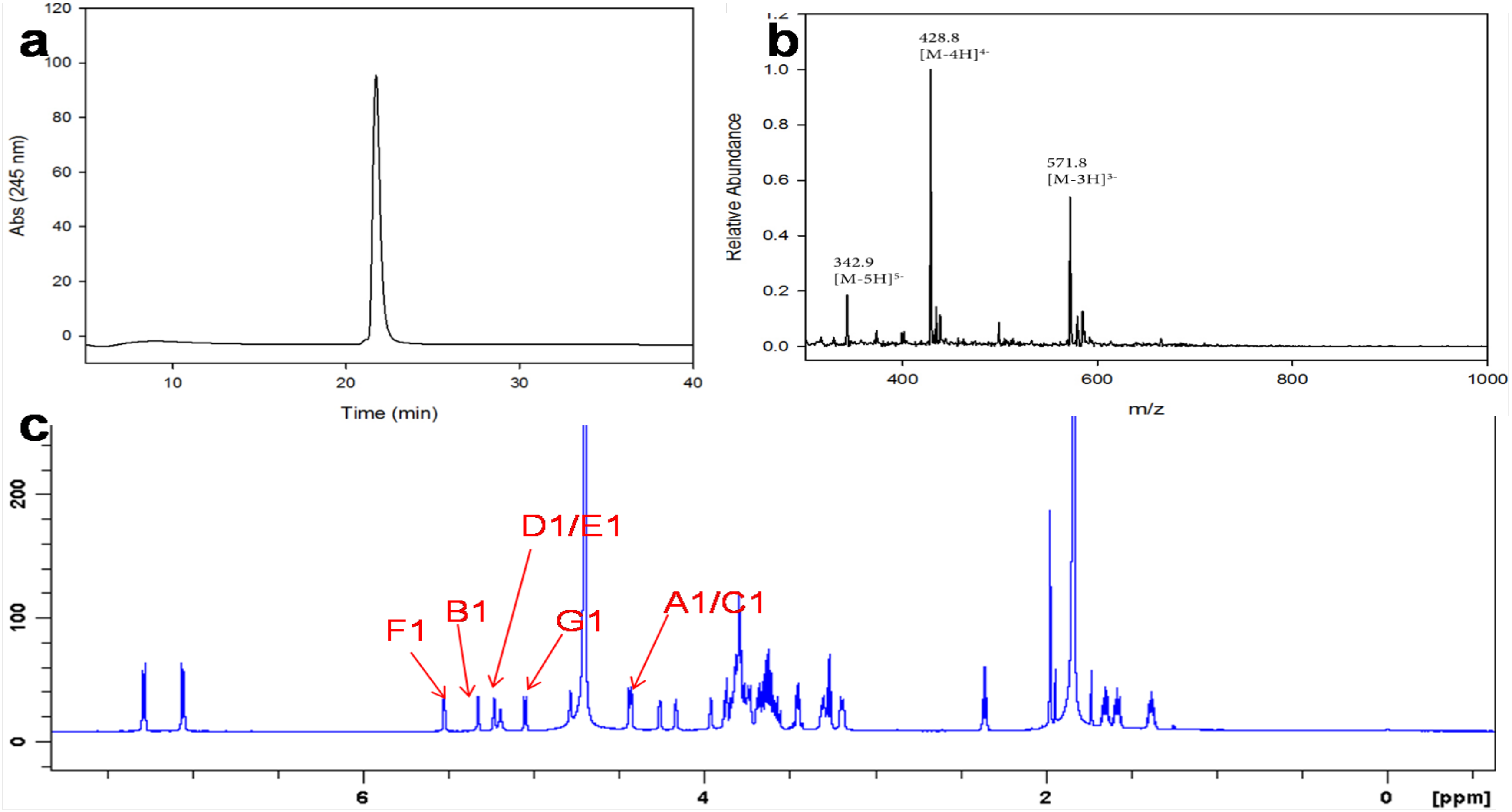

Supplementary Fig. S74. HPLC analysis (a), MS spectrum (b) and ^{1}H NMR spectrum (c) of compound **25**.

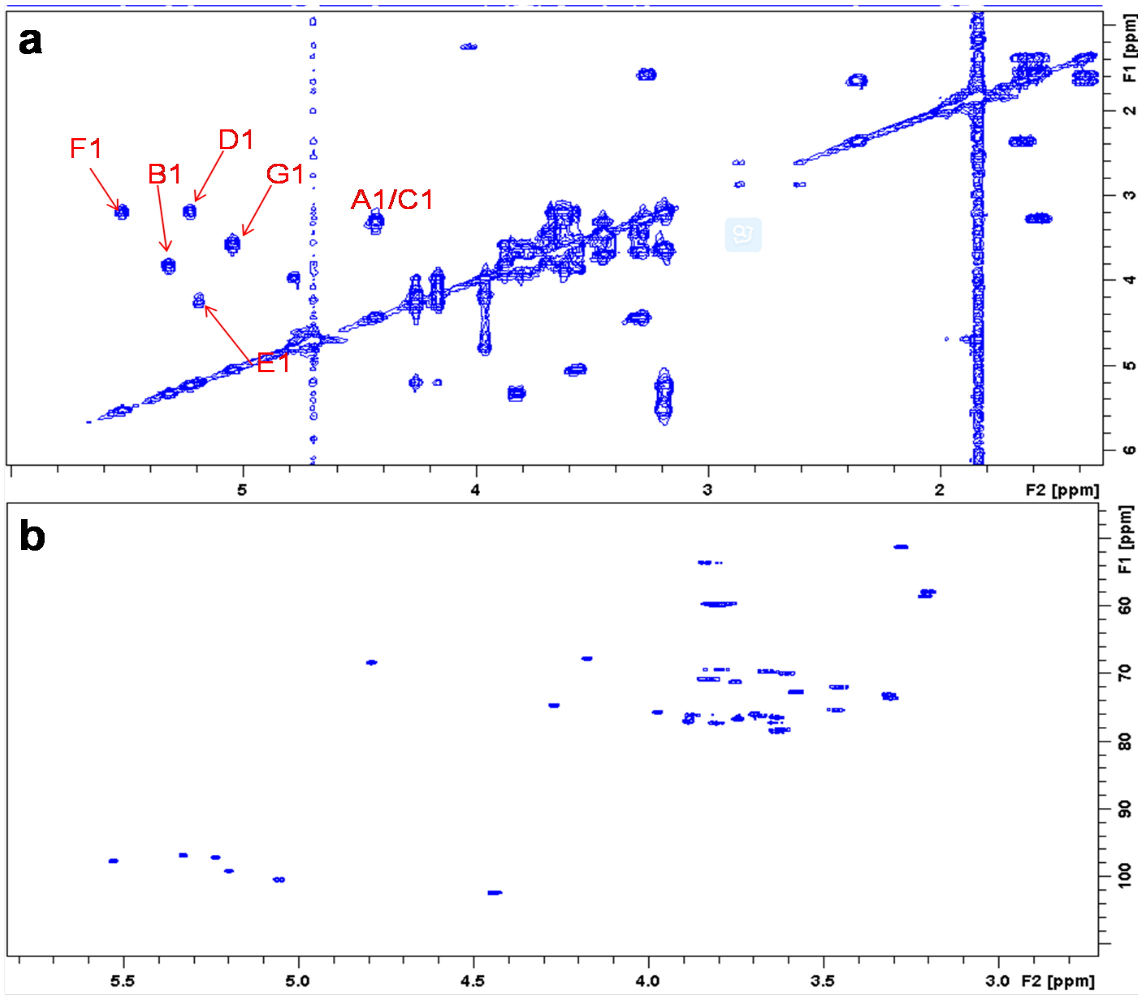

Supplementary Fig. S75. 2D NMR COSY (a) and HSQC (b) spectra of compound **25.**

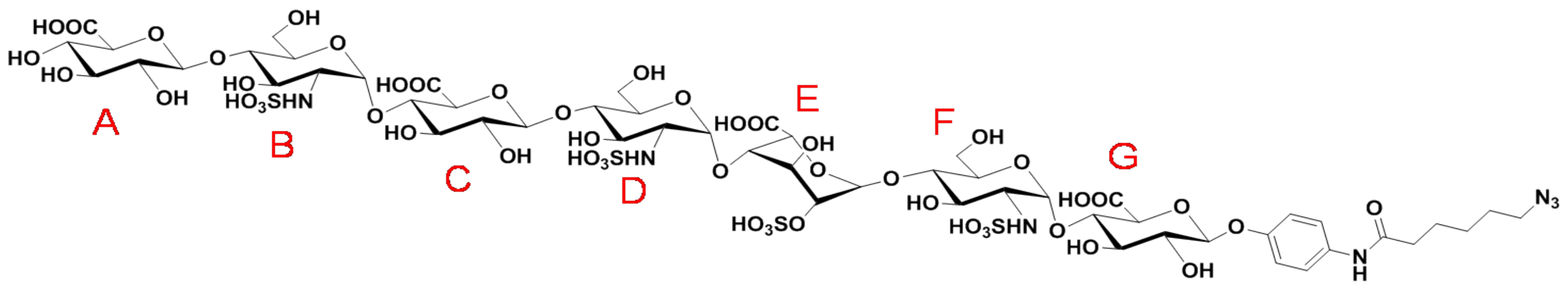

	1	2	3	4	5	6a	6b
A	4.44	3.29	3.44	3.45	3.70	----------	----------
B	5.54	3.19	3.62	3.74	3.74	3.72	3.80
C	4.46	3.33	3.74	3.76	3.76	----------	----------
D	5.24	3.17	3.63	3.76	3.76	3.65	3.81
E	5.18	4.24	4.18	3.96	4.90	----------	----------
F	5.51	3.19	3.58	3.63	3.72	3.72	3.77
G	5.04	3.56	3.85	3.76	3.90	----------	----------

Supplementary Fig. S76. ^{1}H NMR chemical shift assignments (in ppm) of compound **26**. The structure of compound **26** is shown. The protons of *p*NA-N_3 are not listed in the table.

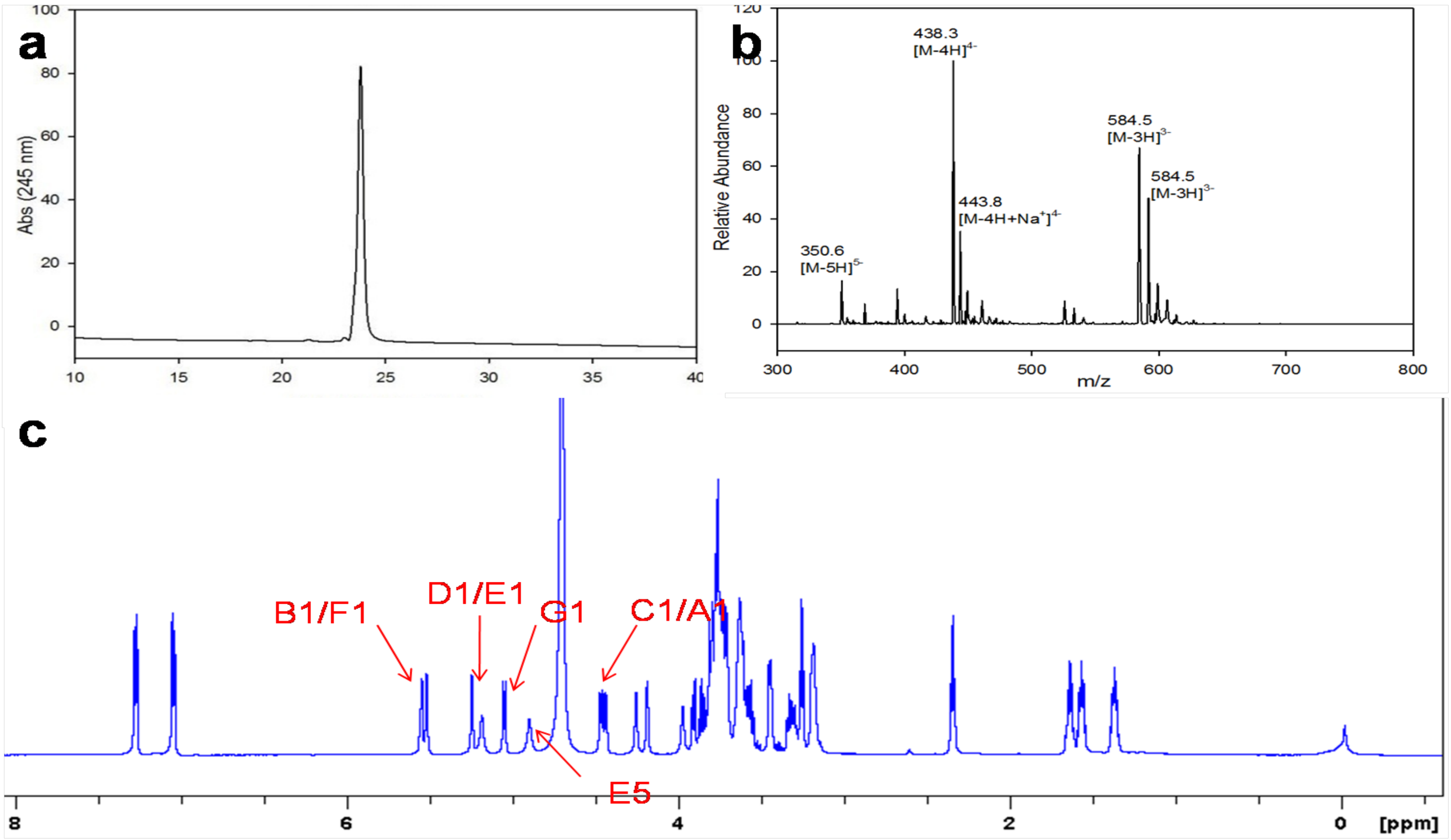

Supplementary Fig. S77. HPLC analysis (a), MS spectrum (b) and ^{1}H NMR spectrum (c) of compound **26**.

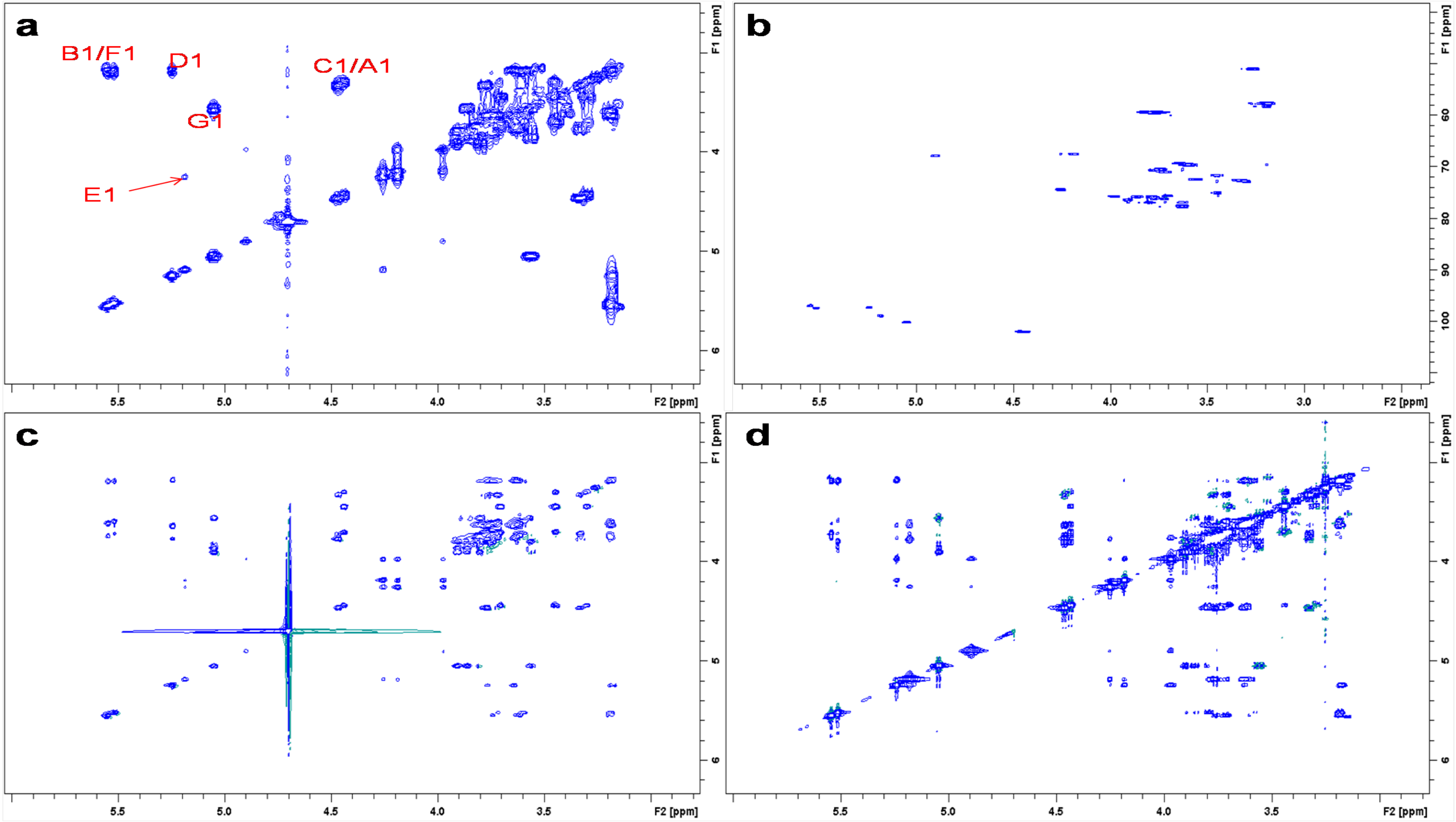

Supplementary Fig. S78. 2D NMR COSY (a), HSQC (b), TOCSY (c) and NOE (d) spectra of compound **26.**

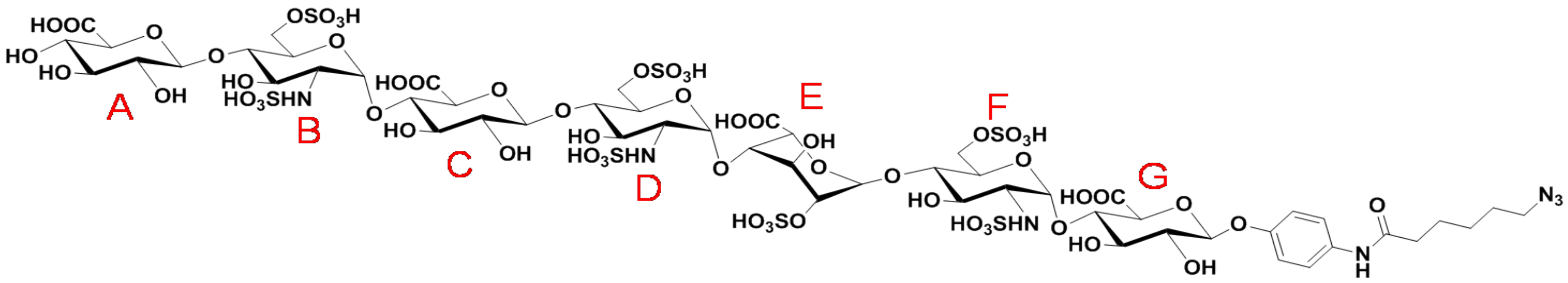

	1	2	3	4	5	6a	6b
A	4.52	3.27	3.46	3.42	3.69	----------	----------
B	5.55	3.23	3.62	3.70	3.96	4.11	4.39
C	4.54	3.32	3.80	3.73	3.75	----------	----------
D	5.33	3.21	3.64	3.67	4.03	4.16	4.38
E	5.17	4.26	4.13	4.03	4.75	----------	----------
F	5.53	3.23	3.60	3.72	3.90	4.16	4.33
G	5.05	3.58	3.86	3.83	3.89	----------	----------

Supplementary Fig. S79. ^{1}H NMR chemical shift assignments (in ppm) of compound **27**. The structure of compound **27** is shown. The protons of *p*NA-N_3 are not listed in the table.

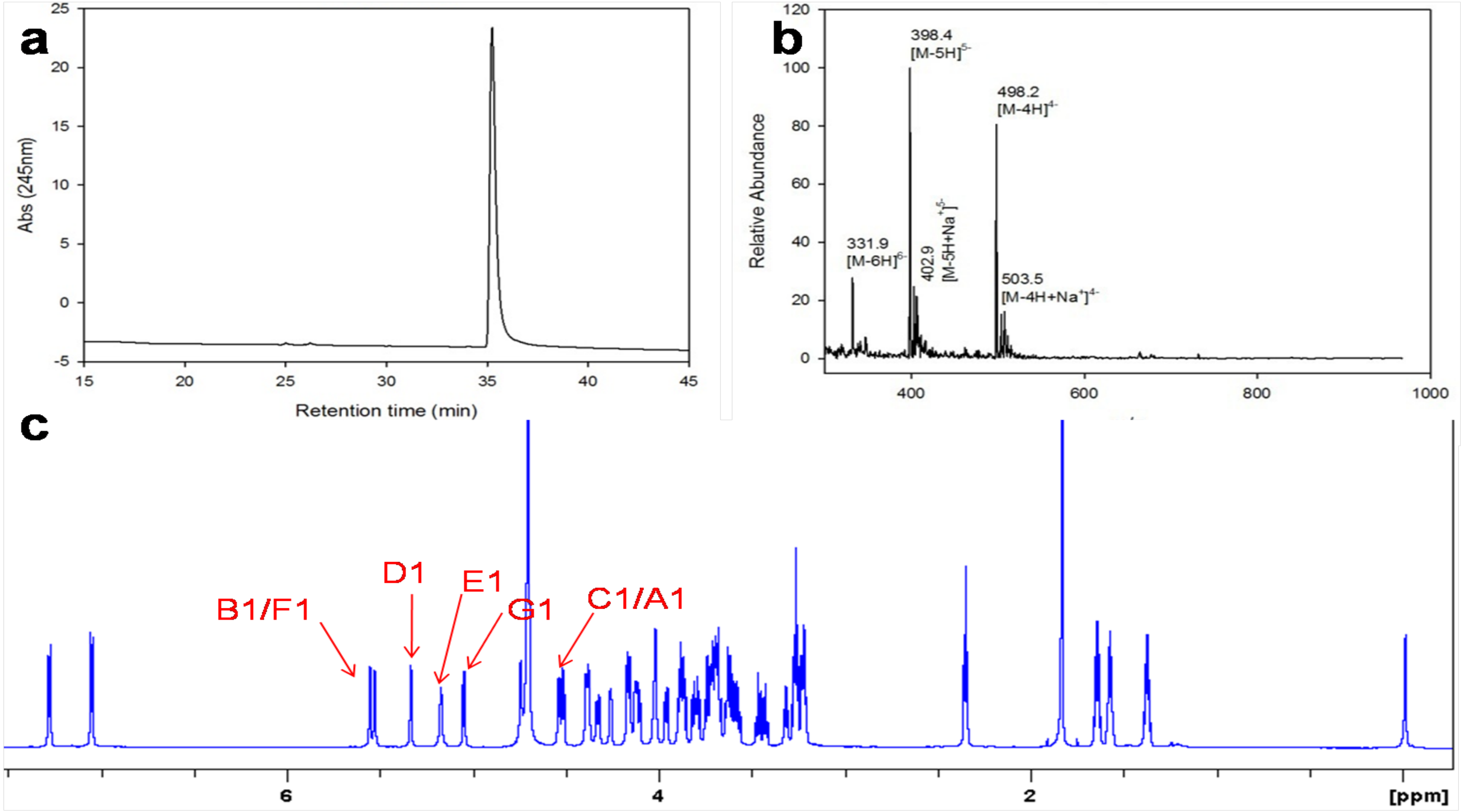

Supplementary Fig. S80. HPLC analysis (a), MS spectrum (b) and ^{1}H NMR spectrum (c) of compound **27**.

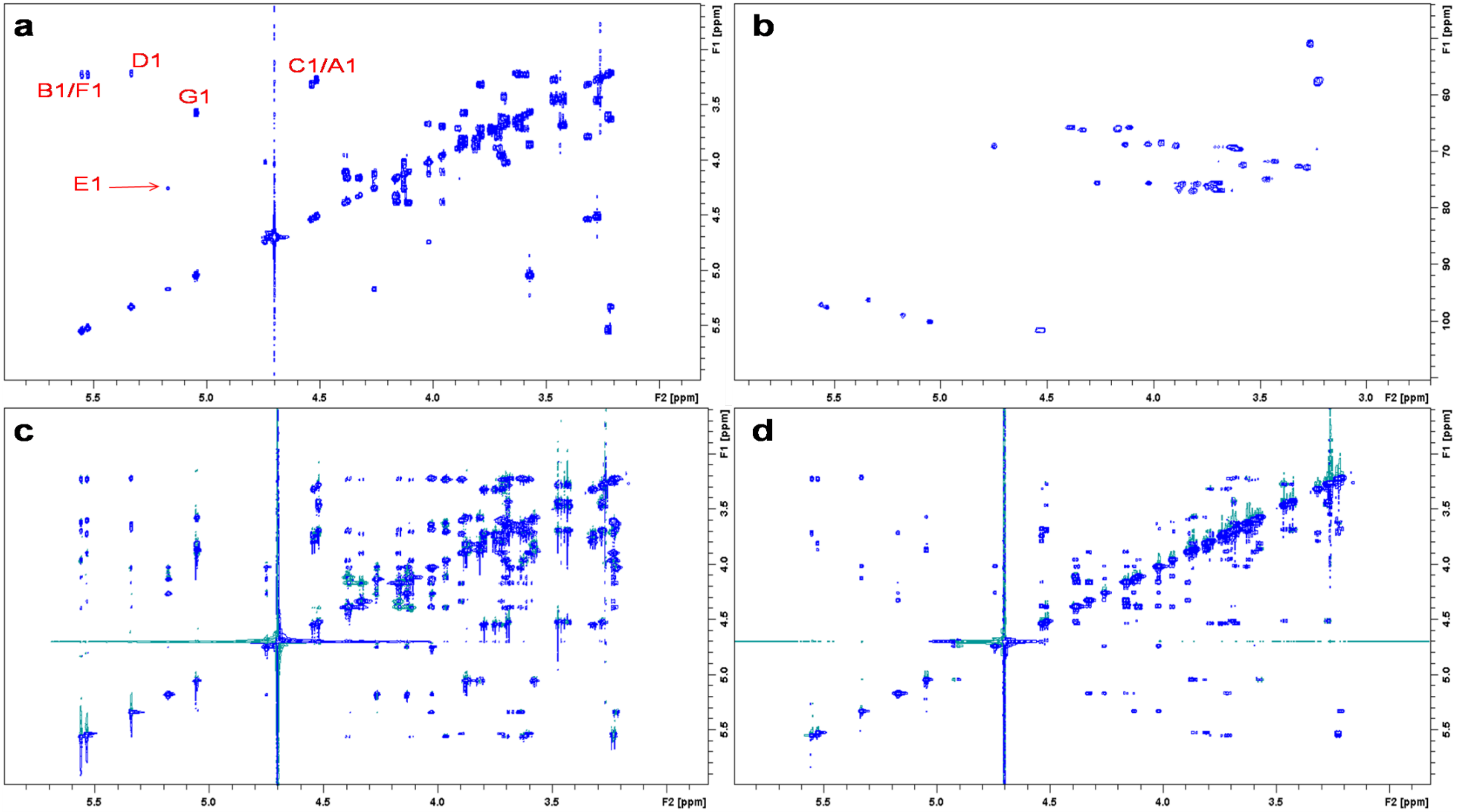

Supplementary Fig. S81. 2D NMR COSY (a), HSQC (b), TOCSY (c) and NOE (d) spectra of compound **27.**

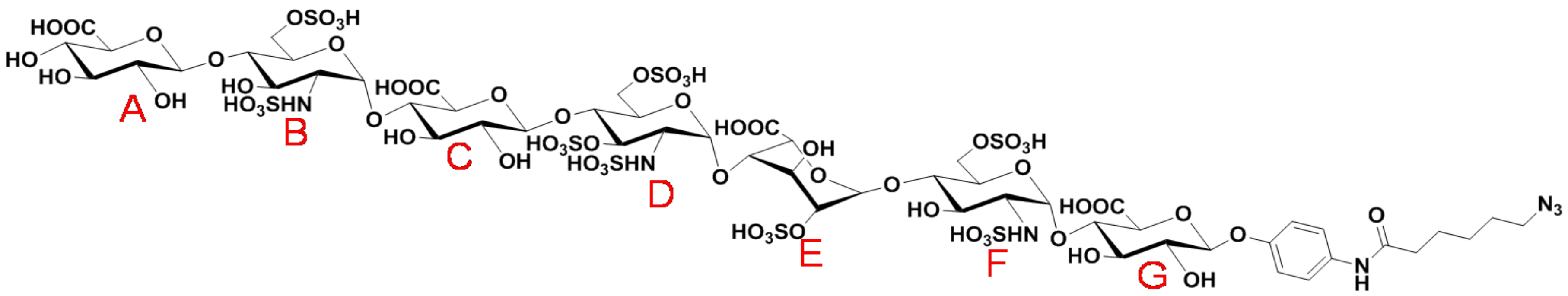

	1	2	3	4	5	6a	6b
A	4.53	3.28	3.46	3.42	3.69	----------	----------
B	5.56	3.22	3.61	3.70	3.96	4.11	4.40
C	4.56	3.34	3.77	3.71	3.75	----------	----------
D	5.43	3.38	4.30	3.91	4.08	4.18	4.42
E	5.13	4.25	4.09	4.06	4.74	----------	----------
F	5.53	3.23	3.60	3.72	3.89	4.16	4.38
G	5.05	3.58	3.85	3.80	3.88	----------	----------

Supplementary Fig. S82. ^{1}H NMR chemical shift assignments (in ppm) of compound **28**. The structure of compound **28** is shown. The protons of *p*NA-N_3 are not listed in the table.

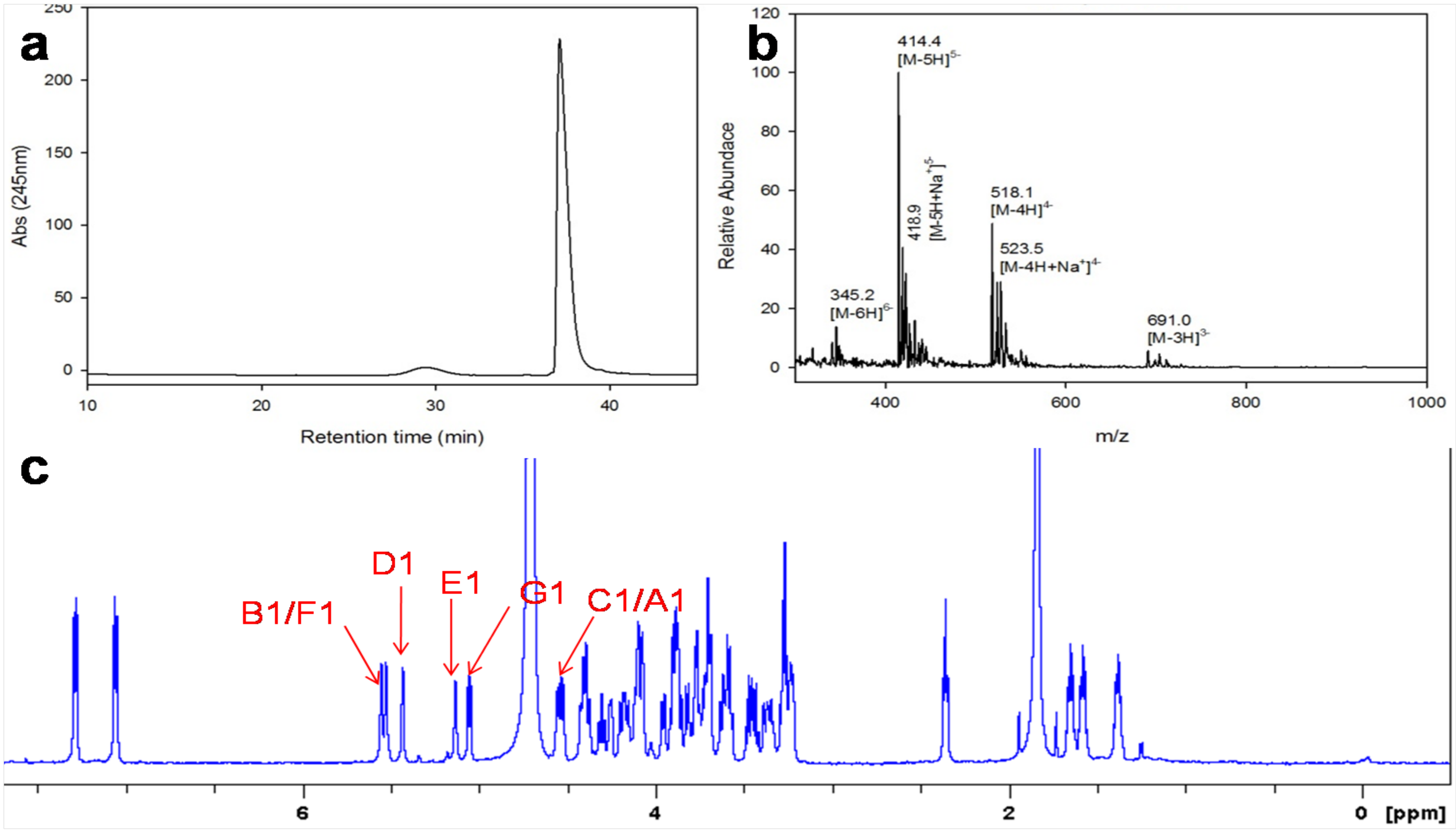

Supplementary Fig. S83. HPLC analysis (a), MS spectrum (b) and ^{1}H NMR spectrum (c) of compound **28**.

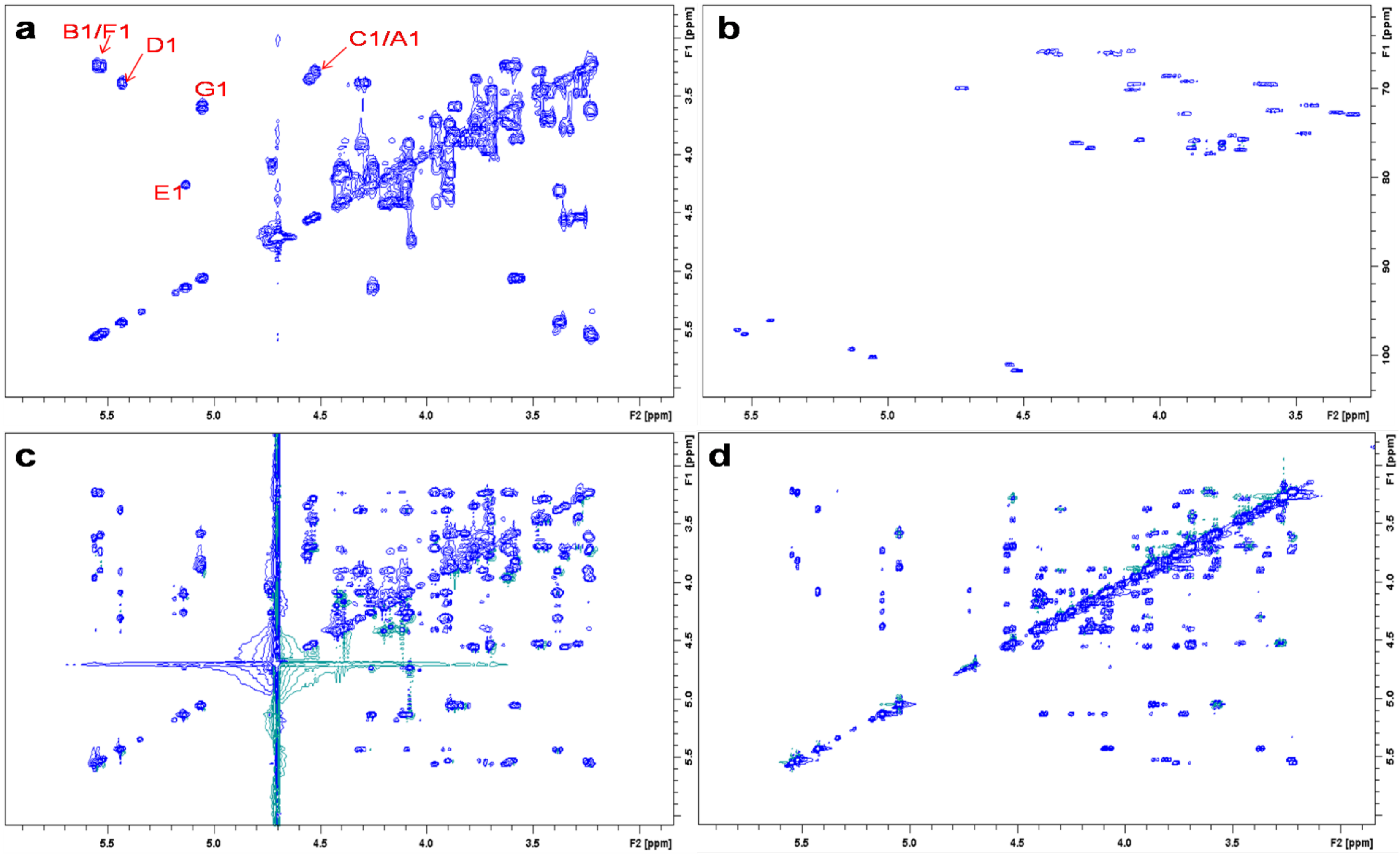

Supplementary Fig. S84. 2D NMR COSY (a), HSQC (b), TOCSY (c) and NOE (d) spectra of compound **28.**

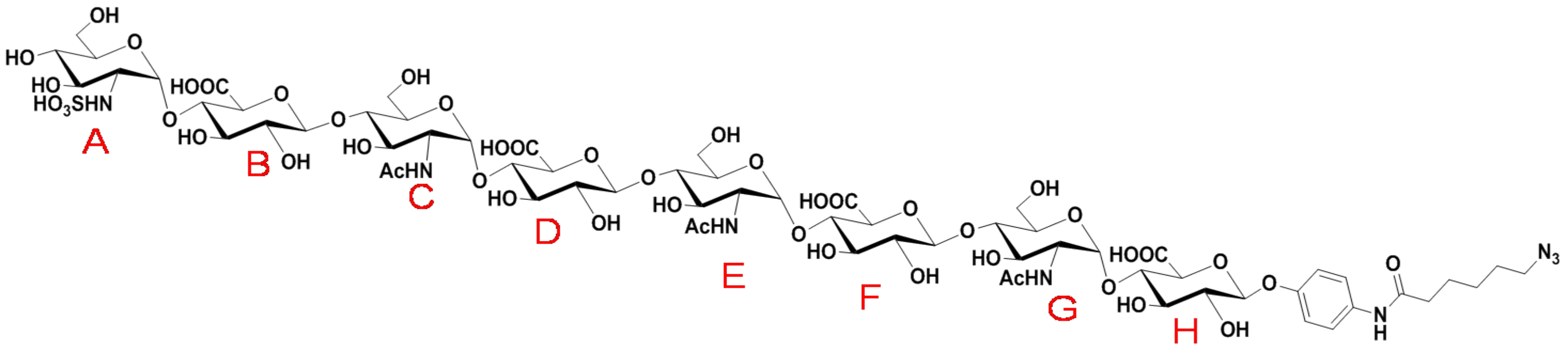

	1	2	3	4	5	6a	6b
A	5.55	3.14	3.51	3.39	3.62	3.68	3.72
B	4.44	3.30	3.77	3.72	3.82	----------	----------
C	5.31	3.79	3.72	3.77	3.80	3.61	3.78
D	4.42	3.26	3.62	3.69	3.78	----------	----------
E	5.31	3.79	3.72	3.77	3.80	3.61	3.78
F	4.42	3.26	3.62	3.69	3.78	----------	----------
G	5.34	3.80	3.75	3.79	3.81	3.61	3.78
H	5.00	3.50	3.72	3.75	3.86	----------	----------

Supplementary Fig. S85. ^{1}H NMR chemical shift assignments (in ppm) of compound **29**. The structure of compound **29** is shown. The protons of *p*NA-N_3 are not listed in the table.

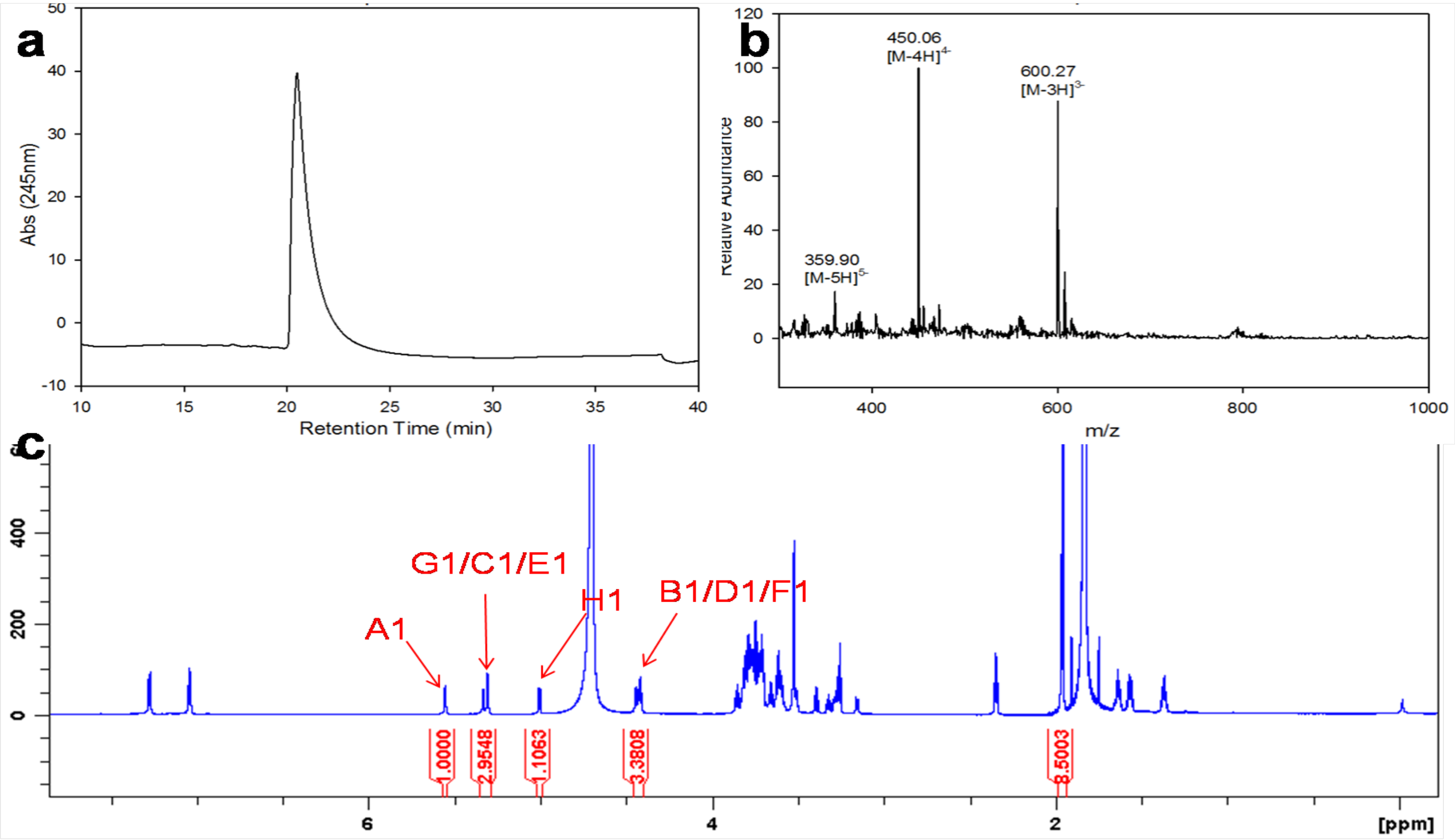

Supplementary Fig. S86. HPLC analysis (a), MS spectrum (b) and ^{1}H NMR spectrum (c) of compound **29**.

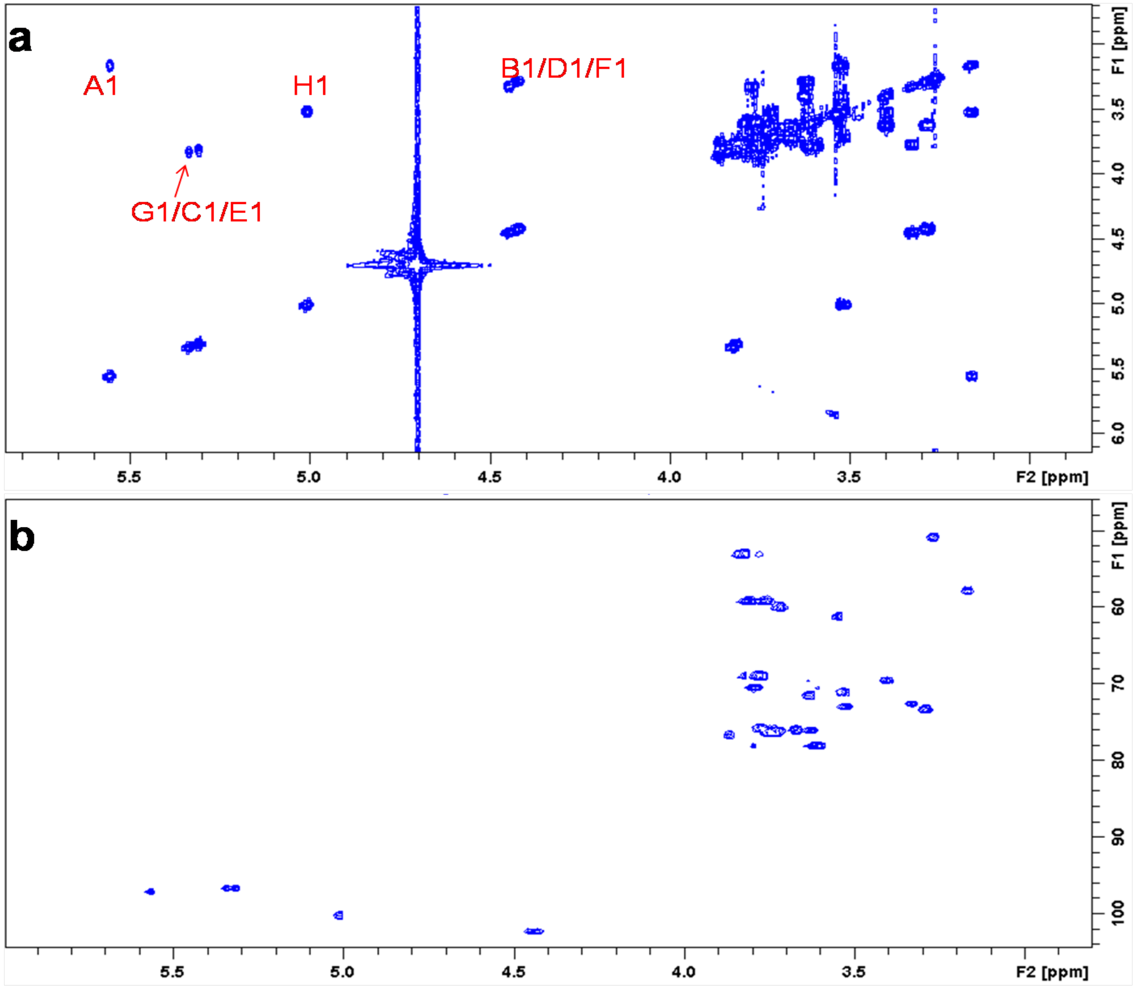

Supplementary Fig. S87. 2D NMR COSY (a) and HSQC (b) spectra of compound **29.**

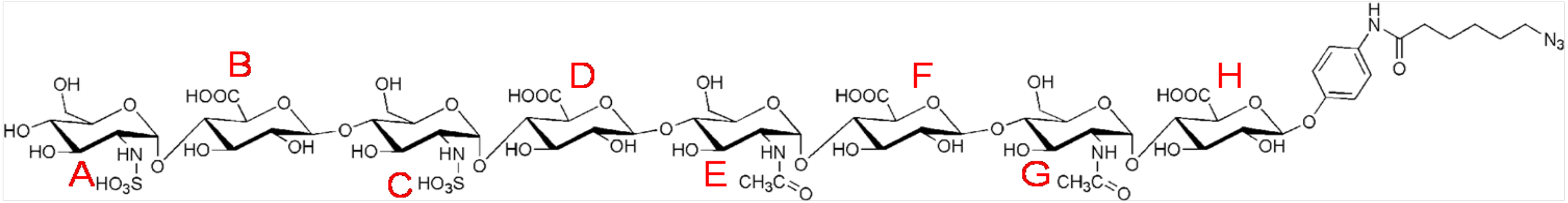

	1	2	3	4	5	6a	6b
A	5.54	3.14	3.50	3.41	3.57	3.62	3.70
B	4.46	3.31	3.77	3.73	3.82	----------	----------
C	5.53	3.17	3.60	3.70	3.71	3.71	3.79
D	4.46	3.31	3.77	3.73	3.82	----------	----------
E	5.29	3.81	3.72	3.77	3.80	3.60	3.80
F	4.43	3.28	3.62	3.71	3.80	----------	----------
G	5.31	3.82	3.75	3.79	3.81	3.61	3.83
H	5.00	3.50	3.70	3.75	3.91	----------	----------

Supplementary Fig. S88. ^{1}H NMR chemical shift assignments (in ppm) of compound **30**. The structure of compound **30** is shown. The protons of *p*NA-N_3 are not listed in the table.

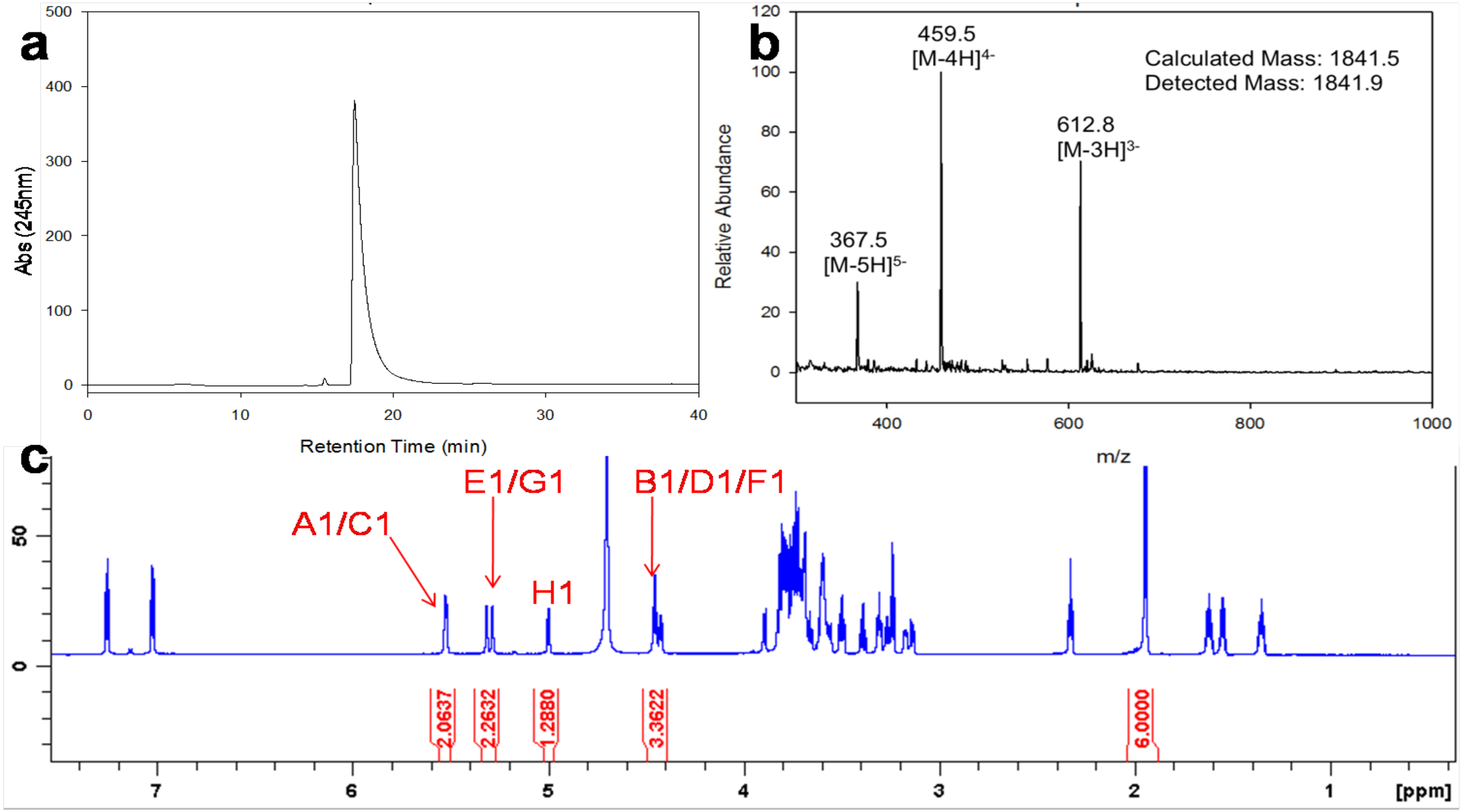

Supplementary Fig. S89. HPLC analysis (a), MS spectrum (b) and ^{1}H NMR spectrum (c) of compound **30**.

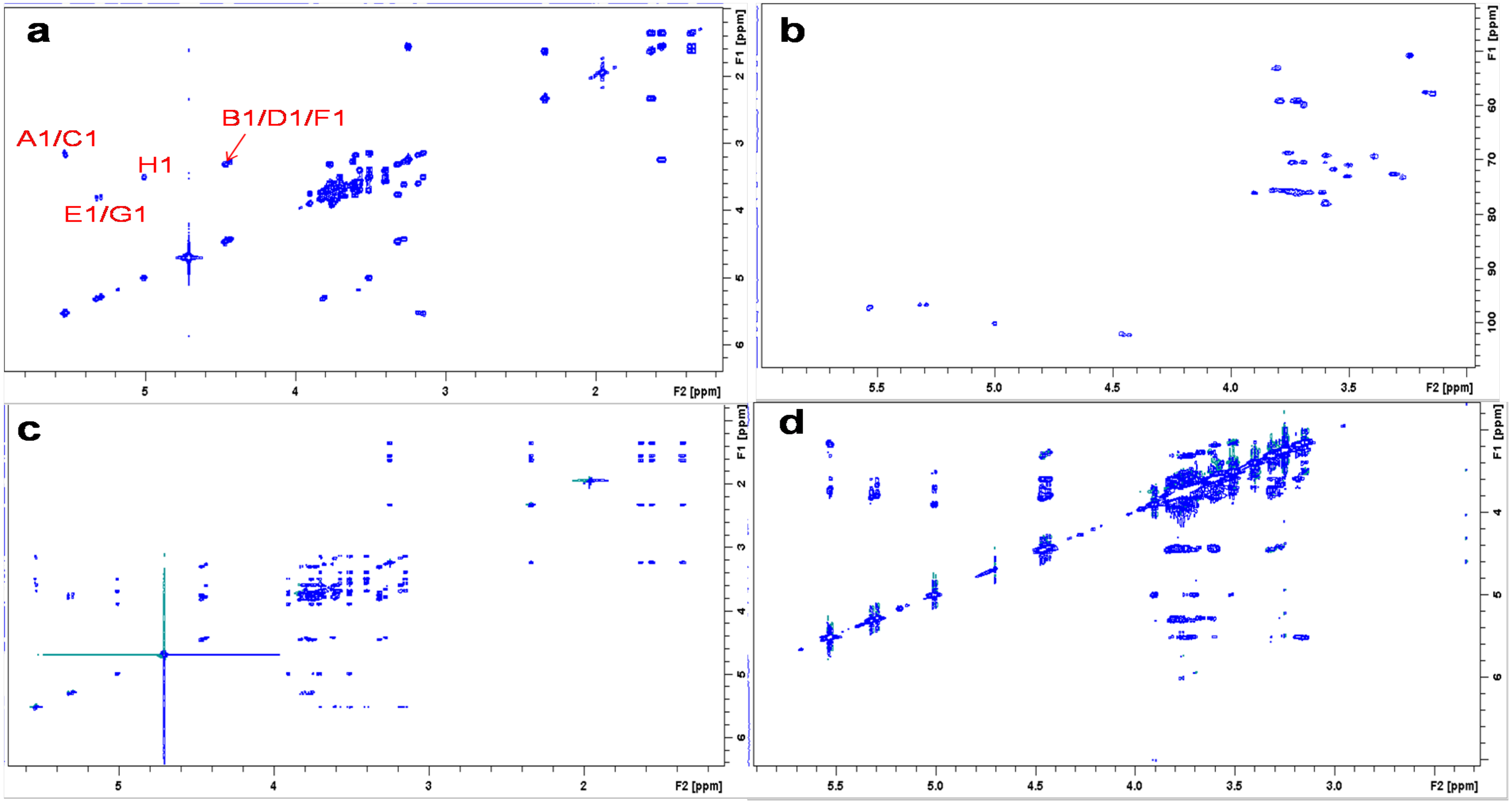

Supplementary Fig. S90. 2D NMR COSY (a), HSQC (b), TOCSY (c) and NOE (d) spectra of compound **30.**

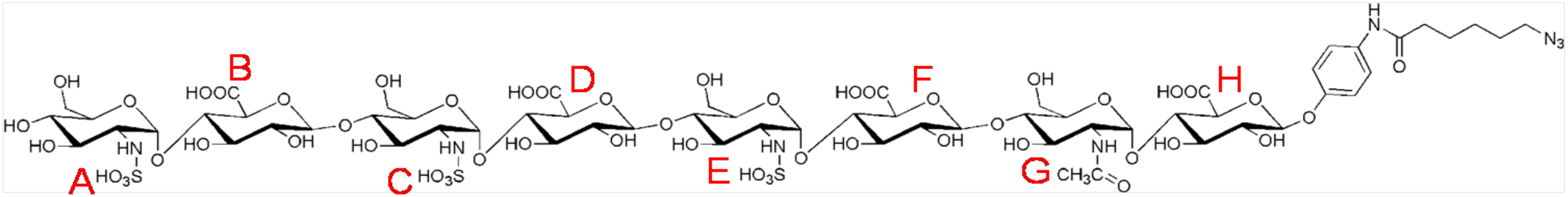

	1	2	3	4	5	6a	6b
A	5.54	3.15	3.50	3.40	3.58	3.70	3.72
B	4.46	3.32	3.75	3.79	3.82	----------	----------
C	5.52	3.17	3.61	3.69	3.70	3.70	3.81
D	4.46	3.32	3.75	3.79	3.82	----------	----------
E	5.52	3.17	3.61	3.69	3.70	3.70	3.81
F	4.46	3.32	3.75	3.79	3.82	----------	----------
G	5.31	3.81	3.78	3.62	3.75	3.60	3.83
H	5.01	3.51	3.71	3.76	3.91	----------	----------

Supplementary Fig. S91. ^{1}H NMR chemical shift assignments (in ppm) of compound **31**. The structure of compound **31** is shown. The protons of *p*NA-N_3 are not listed in the table.

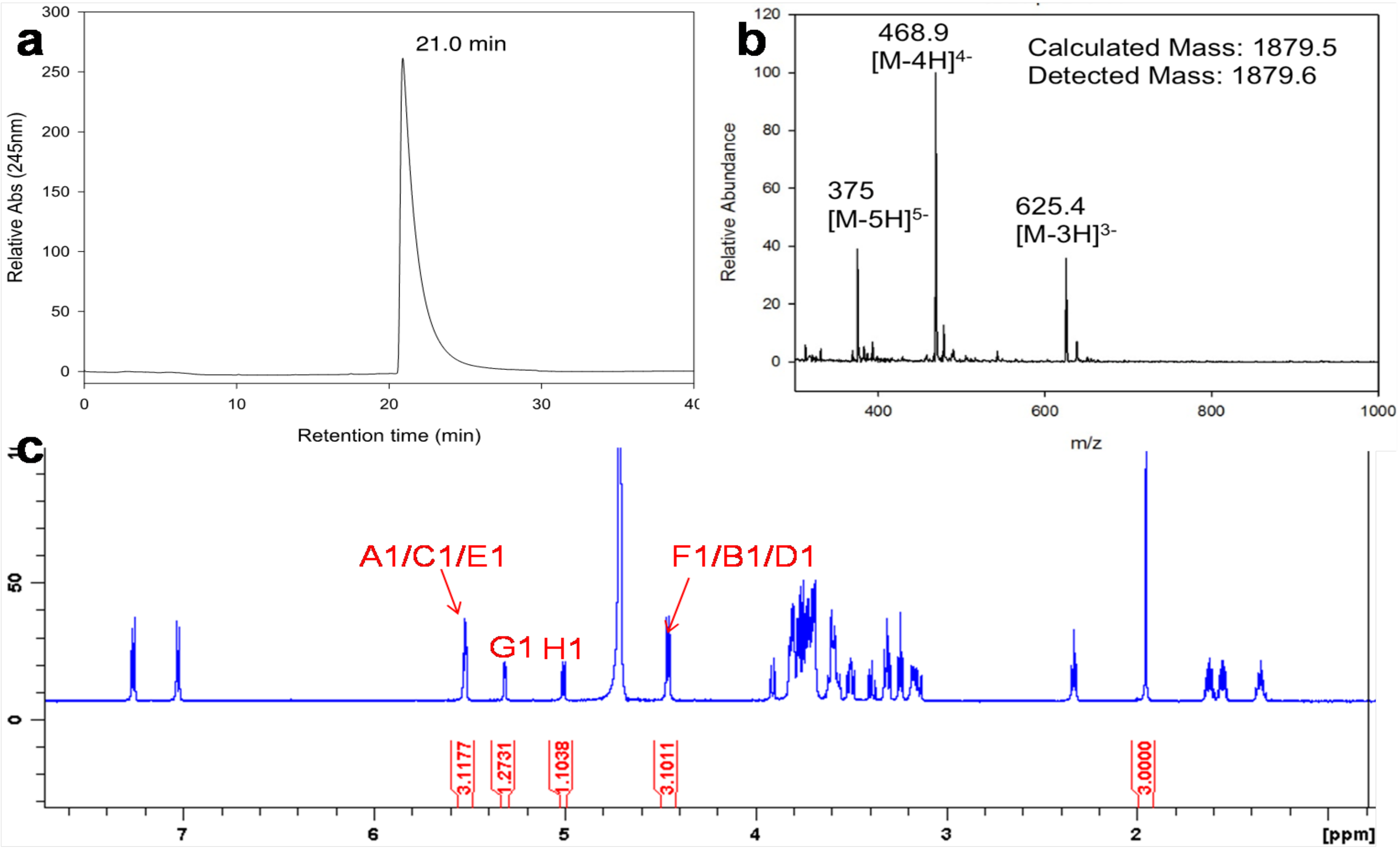

Supplementary Fig. S92. HPLC analysis (a), MS spectrum (b) and ^{1}H NMR spectrum (c) of compound **31**.

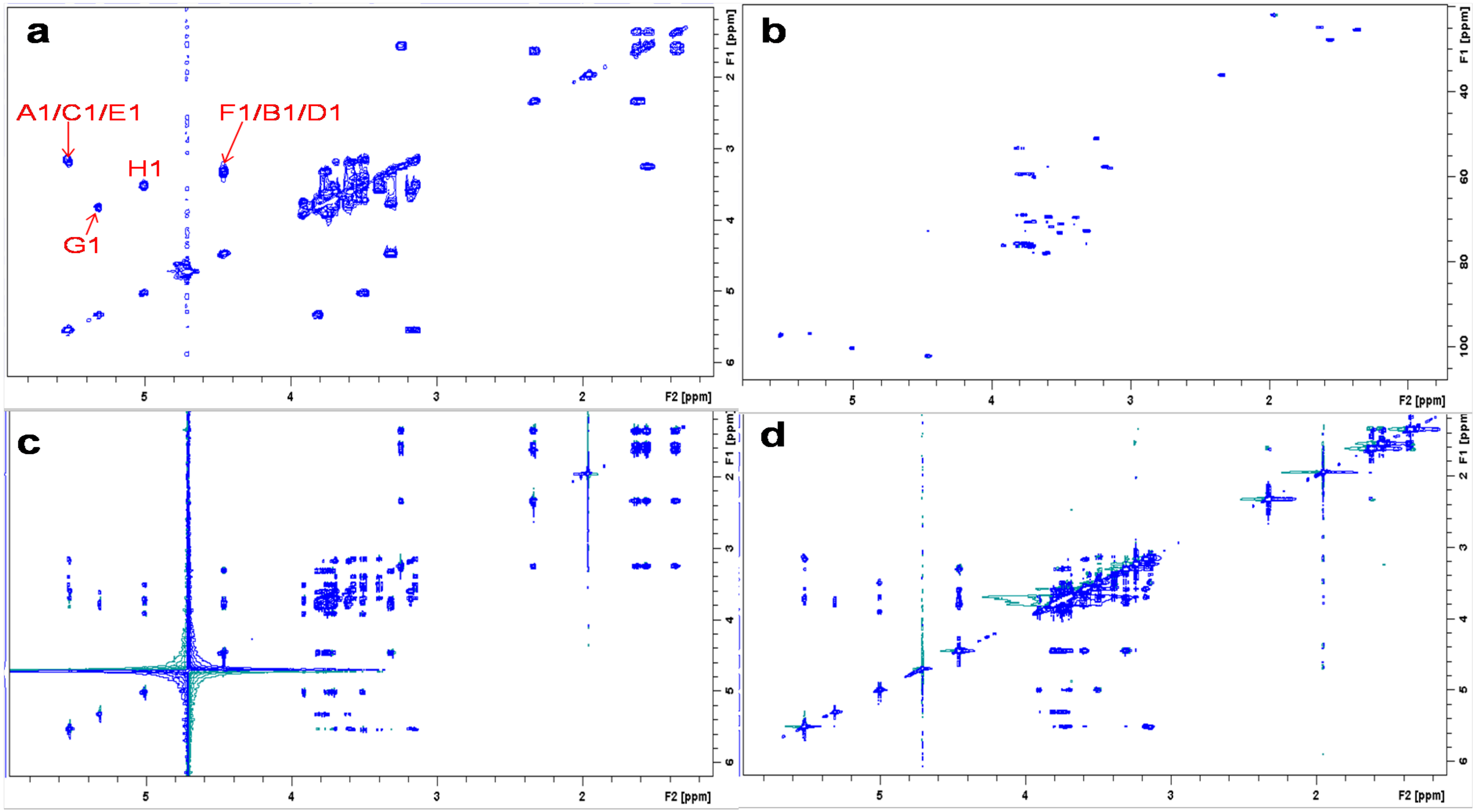

Supplementary Fig. S93. 2D NMR COSY (a), HSQC (b), TOCSY (c) and NOE (d) spectra of compound **31.**

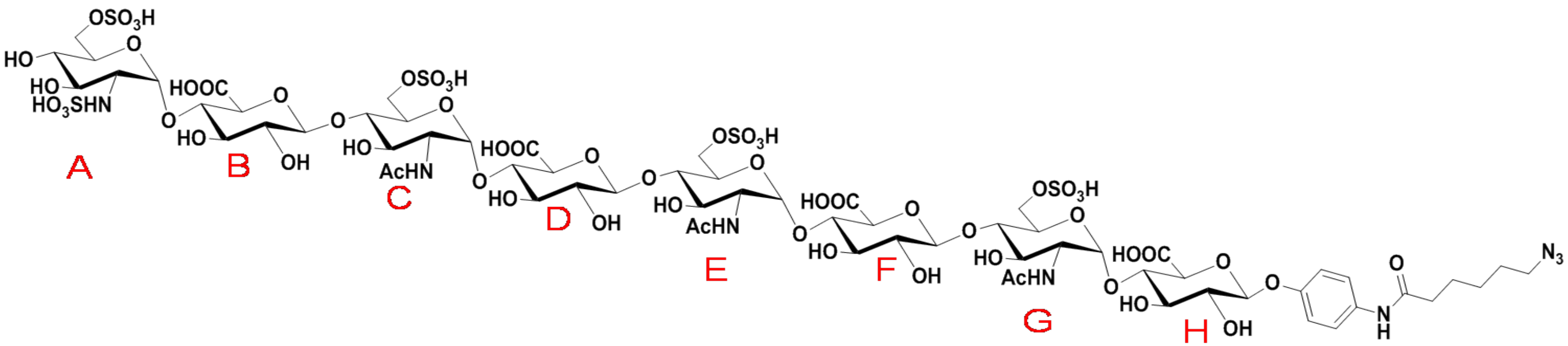

	1	2	3	4	5	6a	6b
A	5.55	3.18	3.54	3.50	3.81	4.08	4.27
B	4.52	3.29	3.78	3.82	3.77	----------	----------
C	5.33	3.83	3.75	3.67	3.96	4.13	4.37
D	4.51	3.26	3.63	3.67	3.75	----------	----------
E	5.33	3.83	3.75	3.67	3.96	4.13	4.37
F	4.51	3.26	3.63	3.67	3.75	----------	----------
G	5.36	3.86	3.78	3.73	3.98	4.10	4.37
H	5.00	3.52	3.70	3.77	3.87	----------	----------

Supplementary Fig. S94. ^{1}H NMR chemical shift assignments (in ppm) of compound **32**. The structure of compound **32** is shown. The protons of *p*NA-N_3 are not listed in the table.

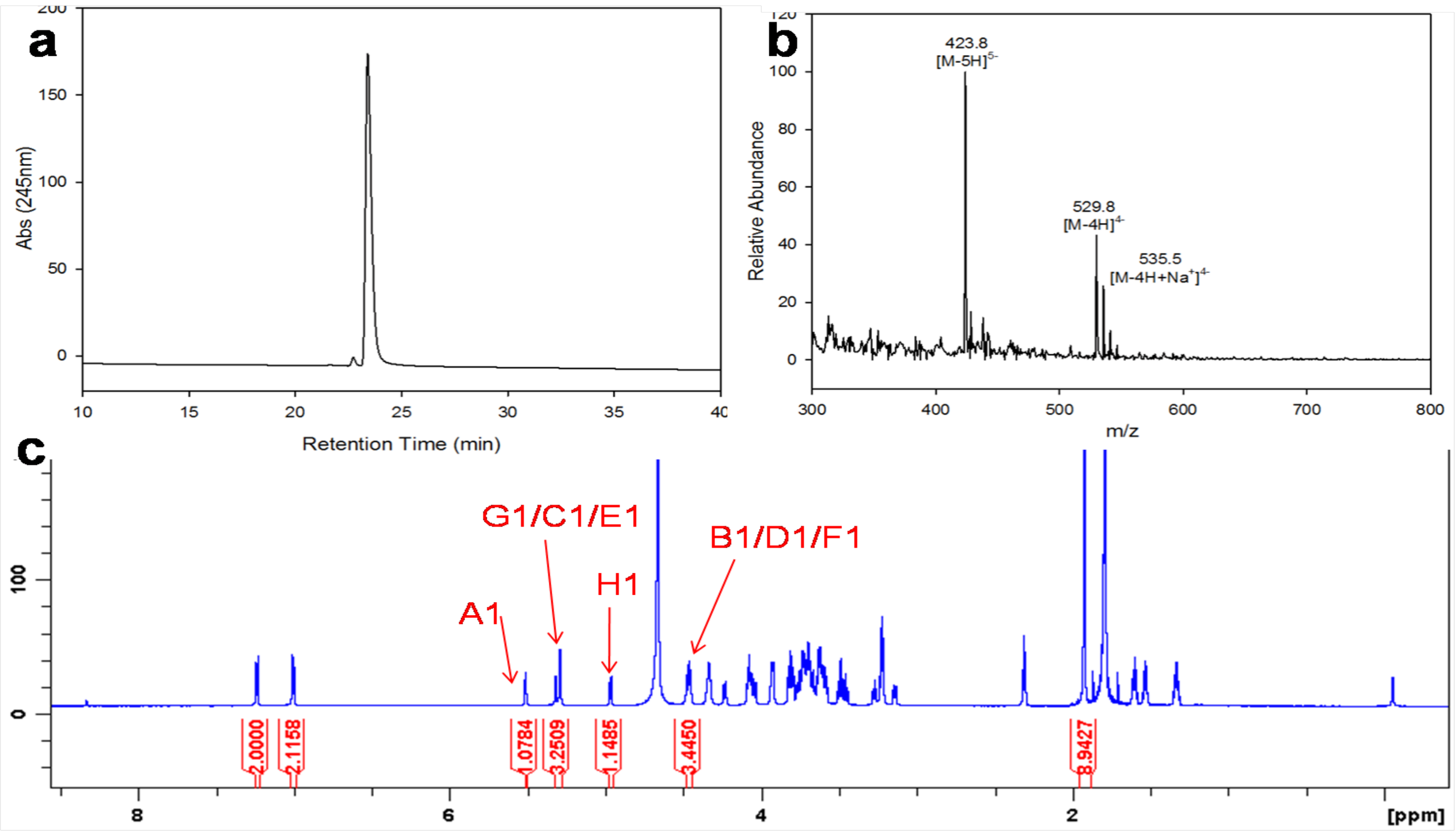

Supplementary Fig. S95. HPLC analysis (a), MS spectrum (b) and ^{1}H NMR spectrum (c) of compound **32**.

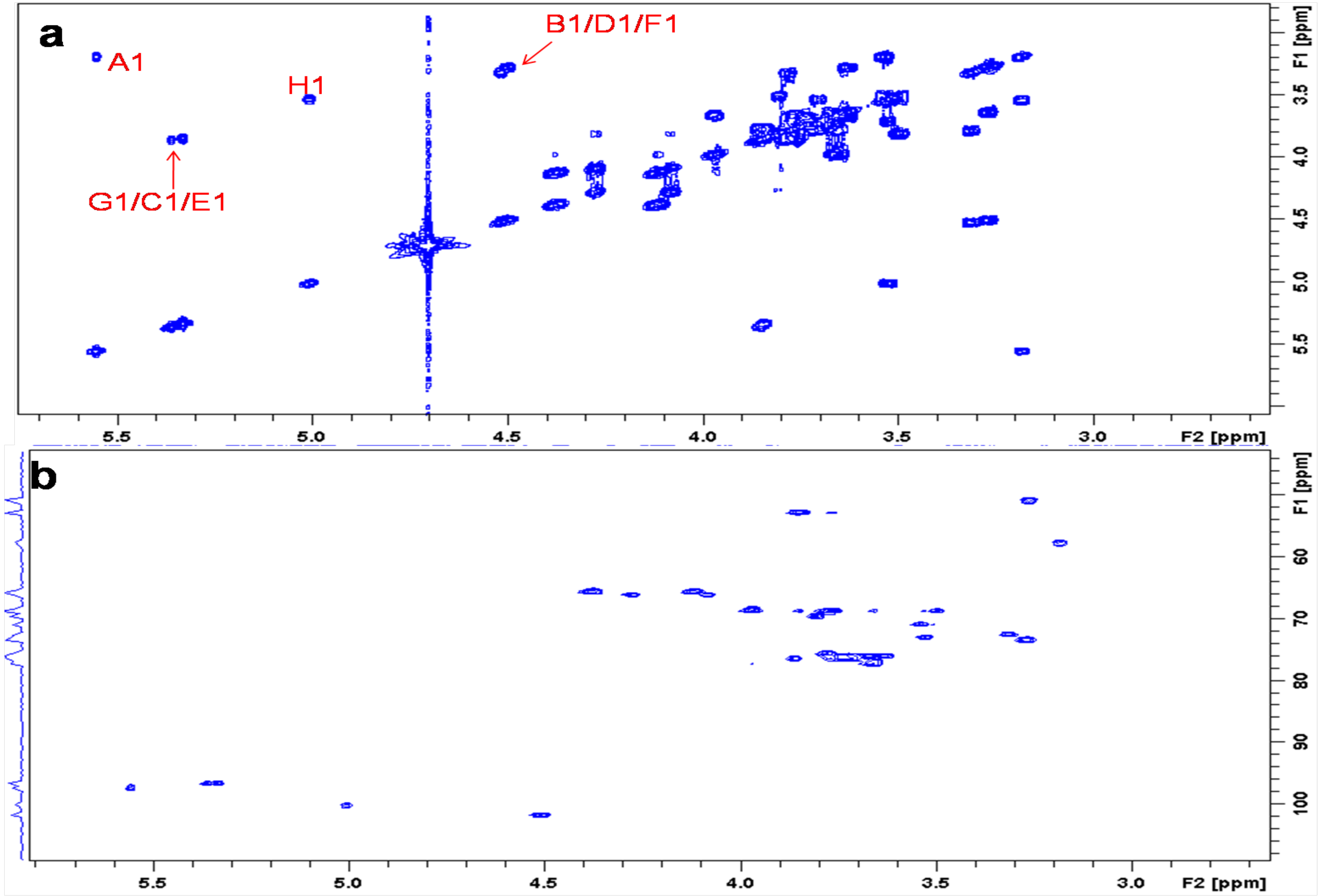

Supplementary Fig. S96. 2D NMR COSY (a) and HSQC (b) spectra of compound **32.**

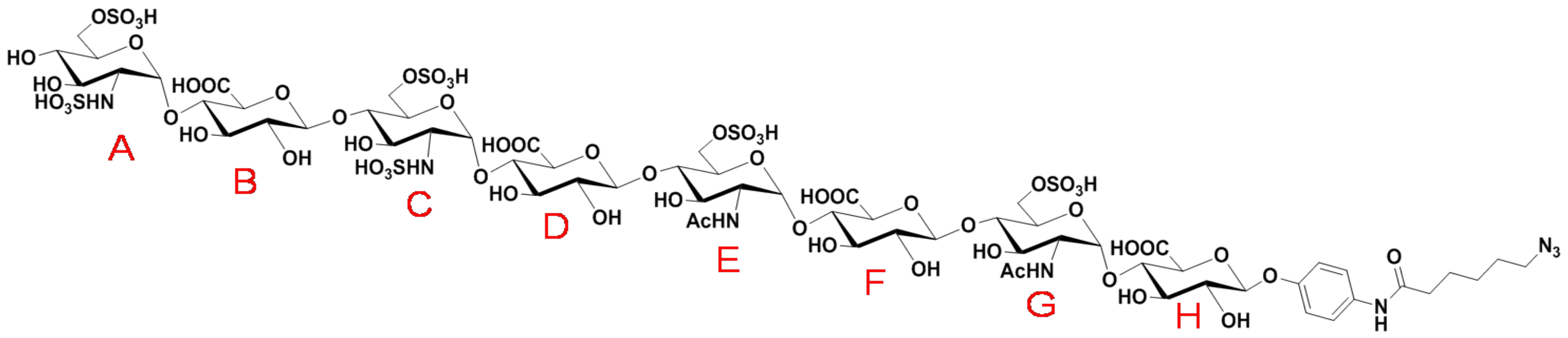

	1	2	3	4	5	6a	6b
A	5.57	3.19	3.55	3.50	3.73	4.10	4.28
B	4.52	3.27	3.64	3.73	3.79	----------	----------
C	5.57	3.19	3.58	3.66	3.98	4.11	4.39
D	4.52	3.27	3.64	3.73	3.79	----------	----------
E	5.37	3.86	3.79	3.67	3.97	4.11	4.39
F	4.52	3.27	3.64	3.73	3.79	----------	----------
G	5.37	3.86	3.79	3.67	3.97	4.11	4.39
H	5.01	3.53	3.69	3.76	3.86	----------	----------

Supplementary Fig. S97. ^{1}H NMR chemical shift assignments (in ppm) of compound **33**. The structure of compound **33** is shown. The protons of *p*NA-N_3 are not listed in the table.

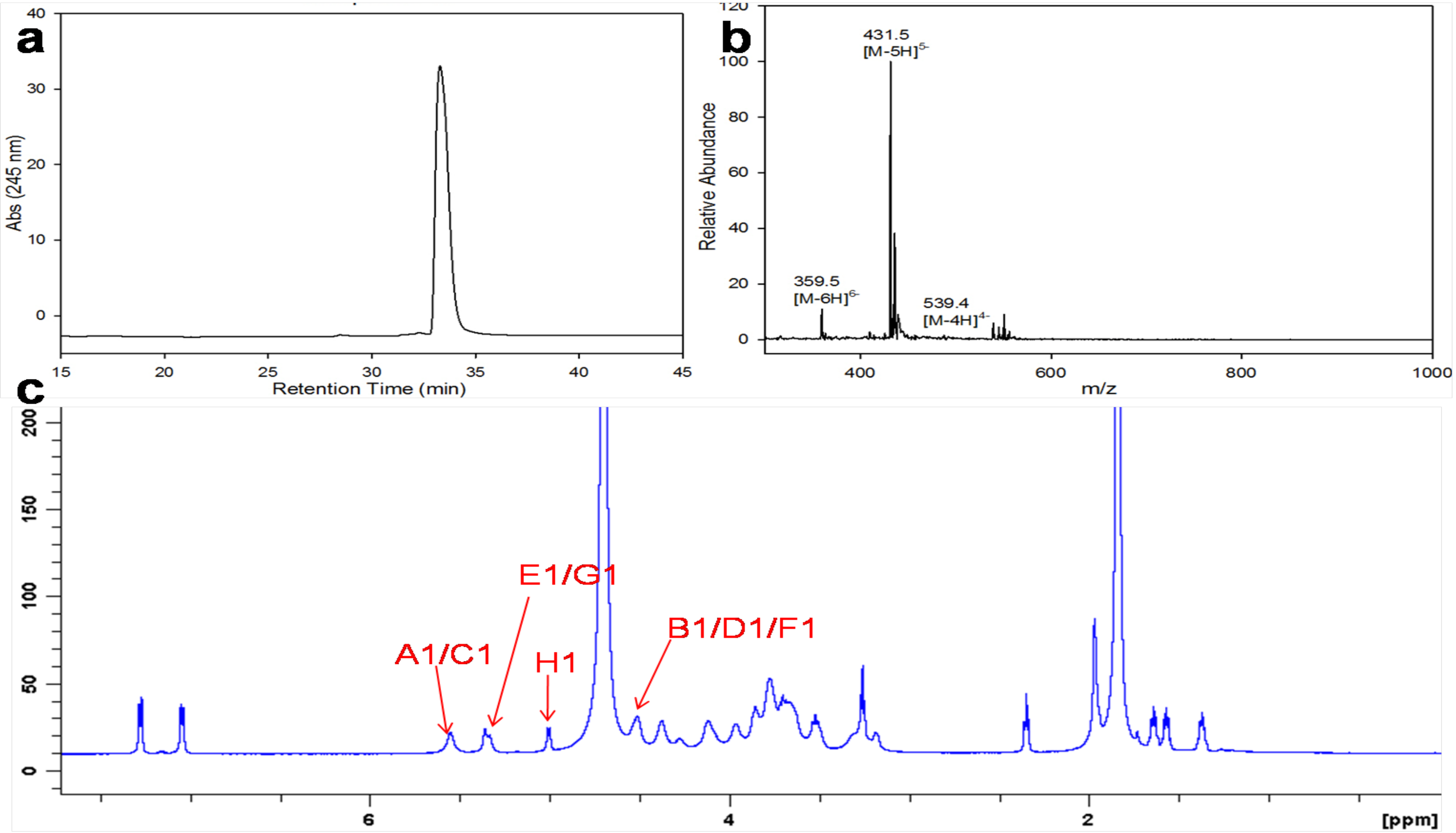

Supplementary Fig. S98. HPLC analysis (a), MS spectrum (b) and ^{1}H NMR spectrum (c) of compound **33**.

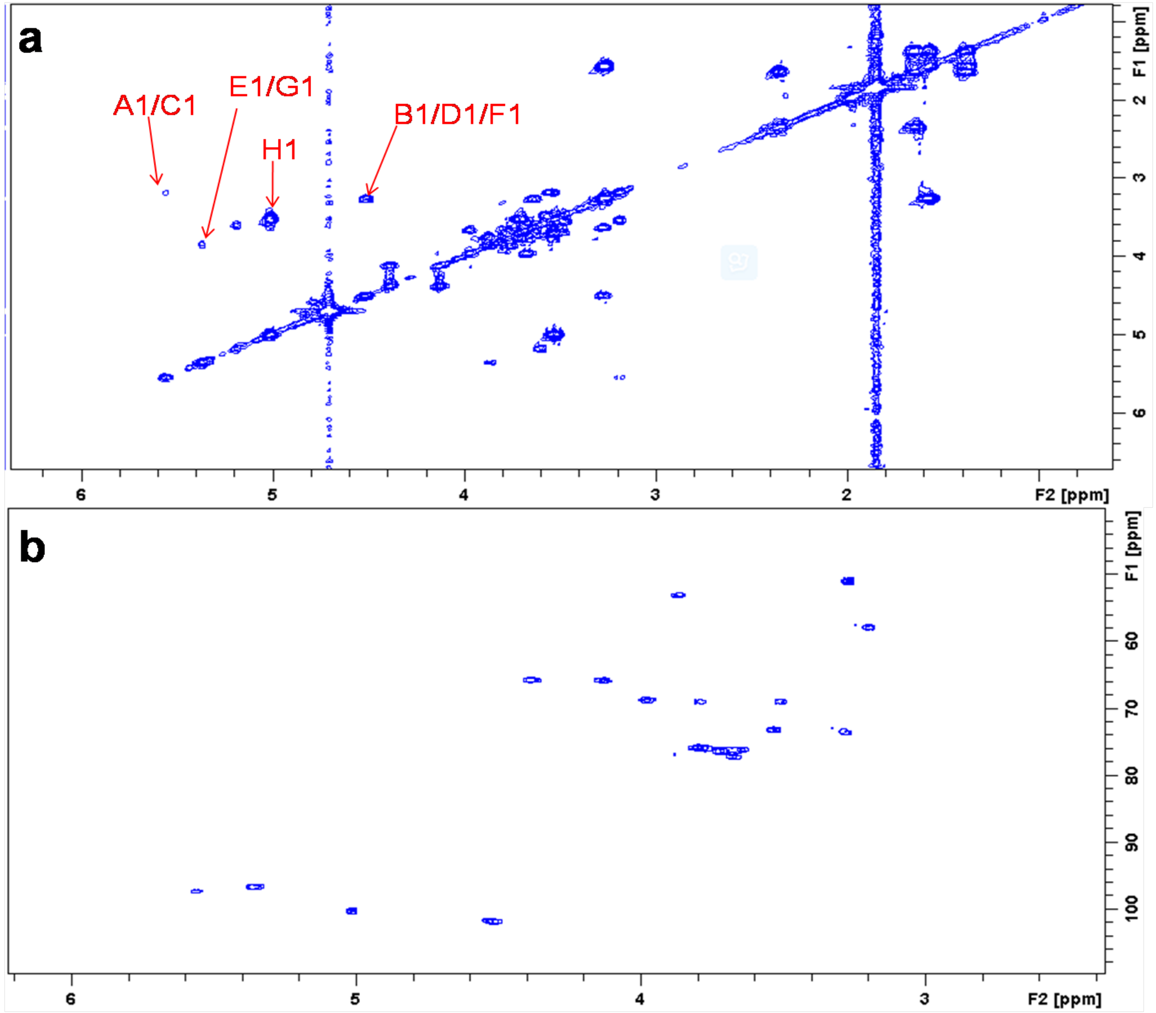

Supplementary Fig. S99. 2D NMR COSY (a) and HSQC (b) spectra of compound **33.**

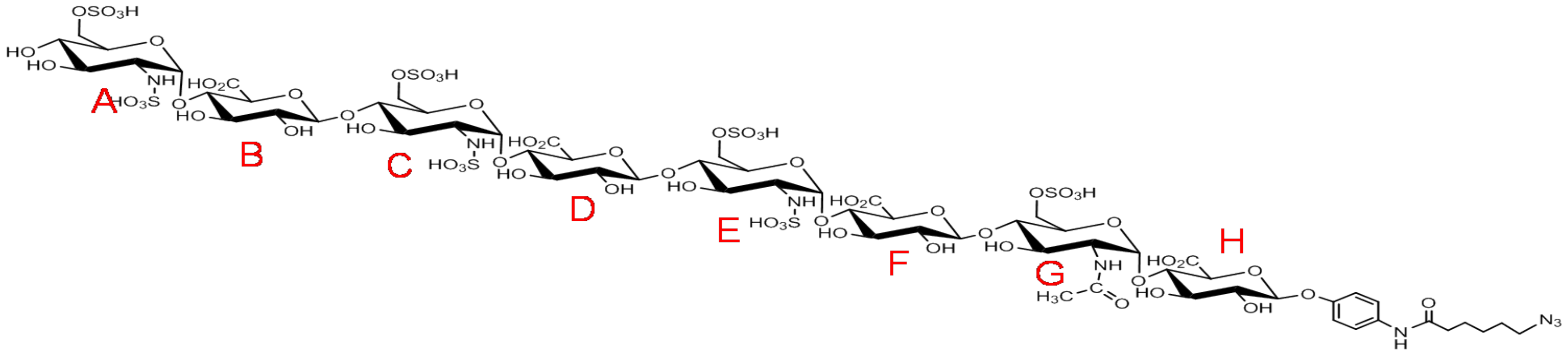

	1	2	3	4	5	6a	6b
A	5.57	3.19	3.55	3.49	3.83	4.08	4.29
B	4.54	3.31	3.80	3.71	3.76	----------	----------
C	5.56	3.23	3.62	3.66	3.96	4.11	4.39
D	4.54	3.31	3.80	3.71	3.76	----------	----------
E	5.56	3.23	3.62	3.66	3.96	4.11	4.39
F	4.54	3.31	3.80	3.71	3.76	----------	----------
G	5.37	3.87	3.81	3.69	3.95	4.14	4.39
H	5.02	3.54	3.73	3.77	3.87	----------	----------

Supplementary Fig. S100. ^{1}H NMR chemical shift assignments (in ppm) of compound **34**. The structure of compound **34** is shown. The protons of *p*NA-N_3 are not listed in the table.

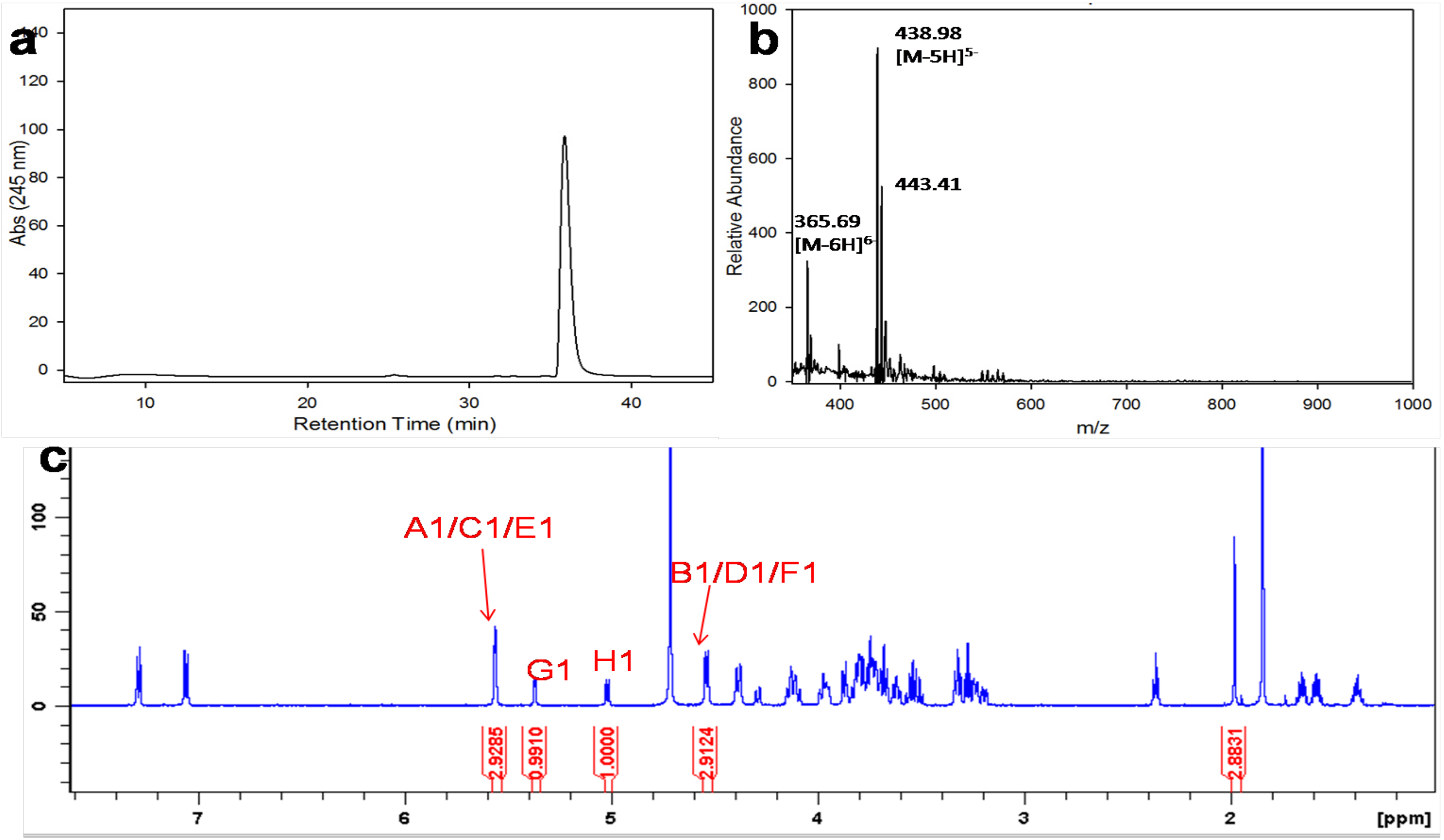

Supplementary Fig. S101. HPLC analysis (a), MS spectrum (b) and ^{1}H NMR spectrum (c) of compound **34**.

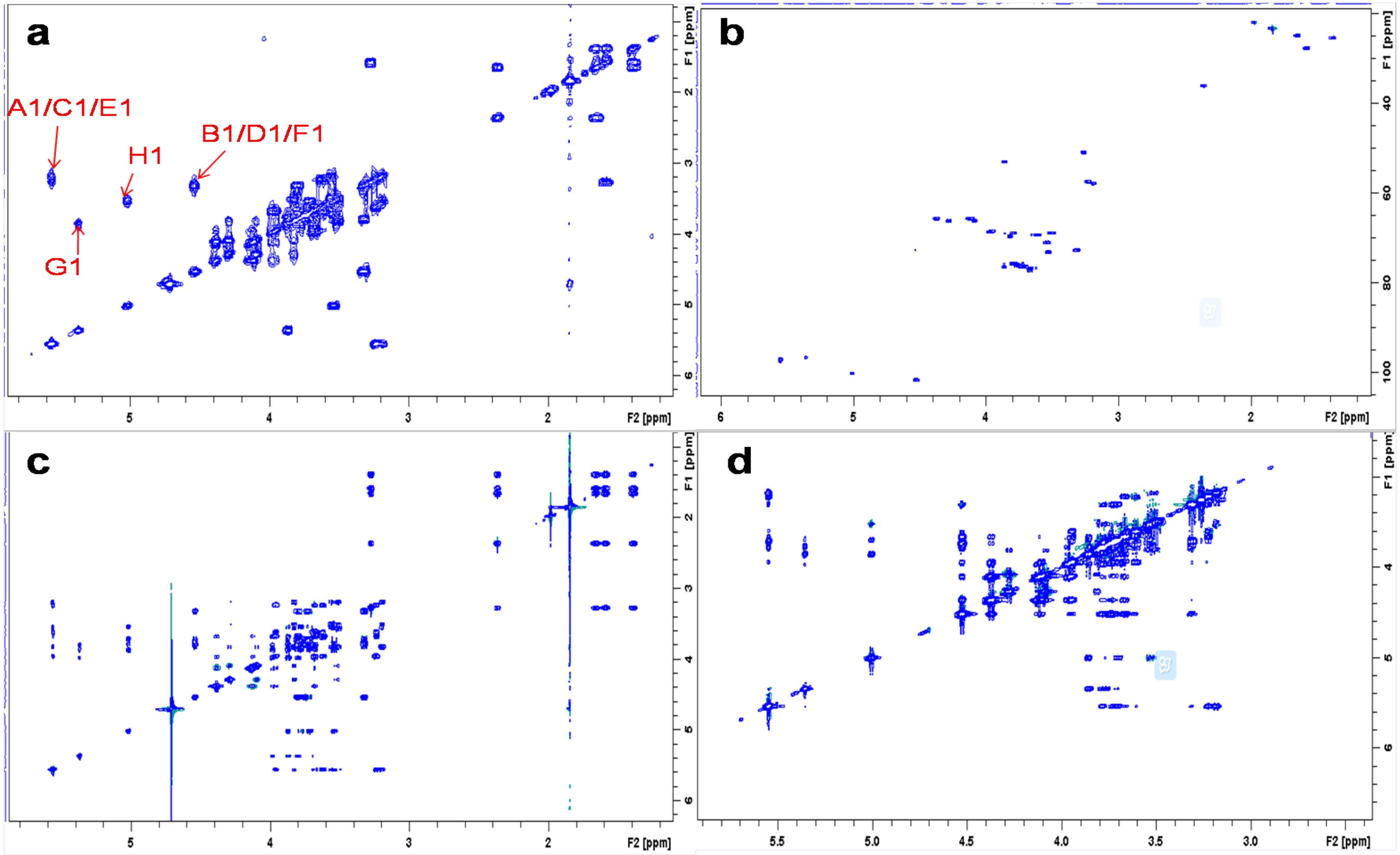

Supplementary Fig. S102. 2D NMR COSY (a), HSQC (b), TOCSY (c) and NOE (d) spectra of compound **34.**

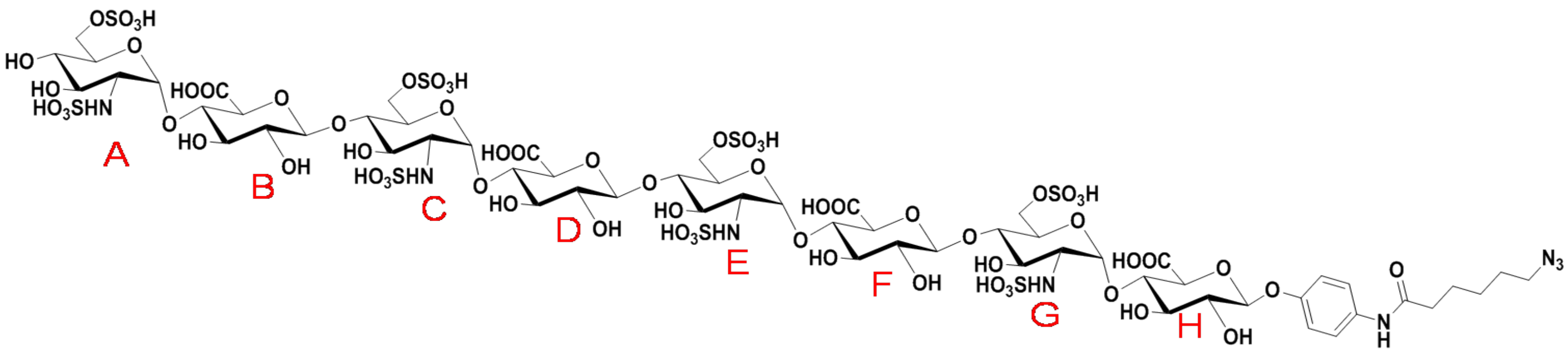

	1	2	3	4	5	6a	6b
A	5.55	3.17	3.54	3.49	3.81	4.08	4.26
B	4.52	3.30	3.78	3.70	3.79	----------	----------
C	5.55	3.19	3.62	3.66	3.94	4.11	4.37
D	4.52	3.30	3.78	3.70	3.79	----------	----------
E	5.55	3.19	3.62	3.66	3.94	4.11	4.37
F	4.52	3.30	3.78	3.70	3.79	----------	----------
G	5.59	3.20	3.60	3.66	3.94	4.11	4.37
H	5.05	3.55	3.87	3.79	3.88	----------	----------

Supplementary Fig. S103. ^{1}H NMR chemical shift assignments (in ppm) of compound **35**. The structure of compound **35** is shown. The protons of *p*NA-N_3 are not listed in the table.

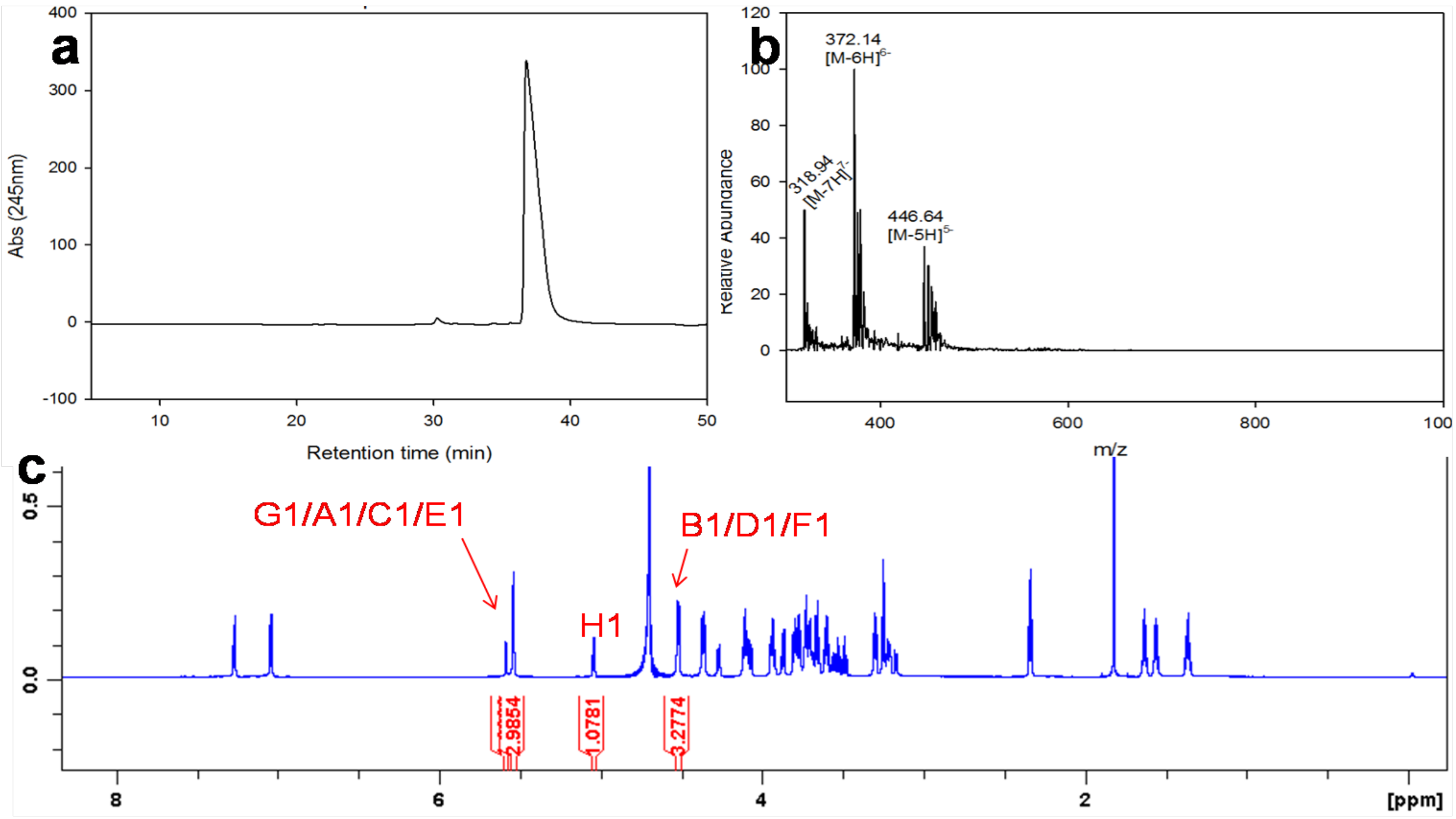

Supplementary Fig. S104. HPLC analysis (a), MS spectrum (b) and ^{1}H NMR spectrum (c) of compound **35**.

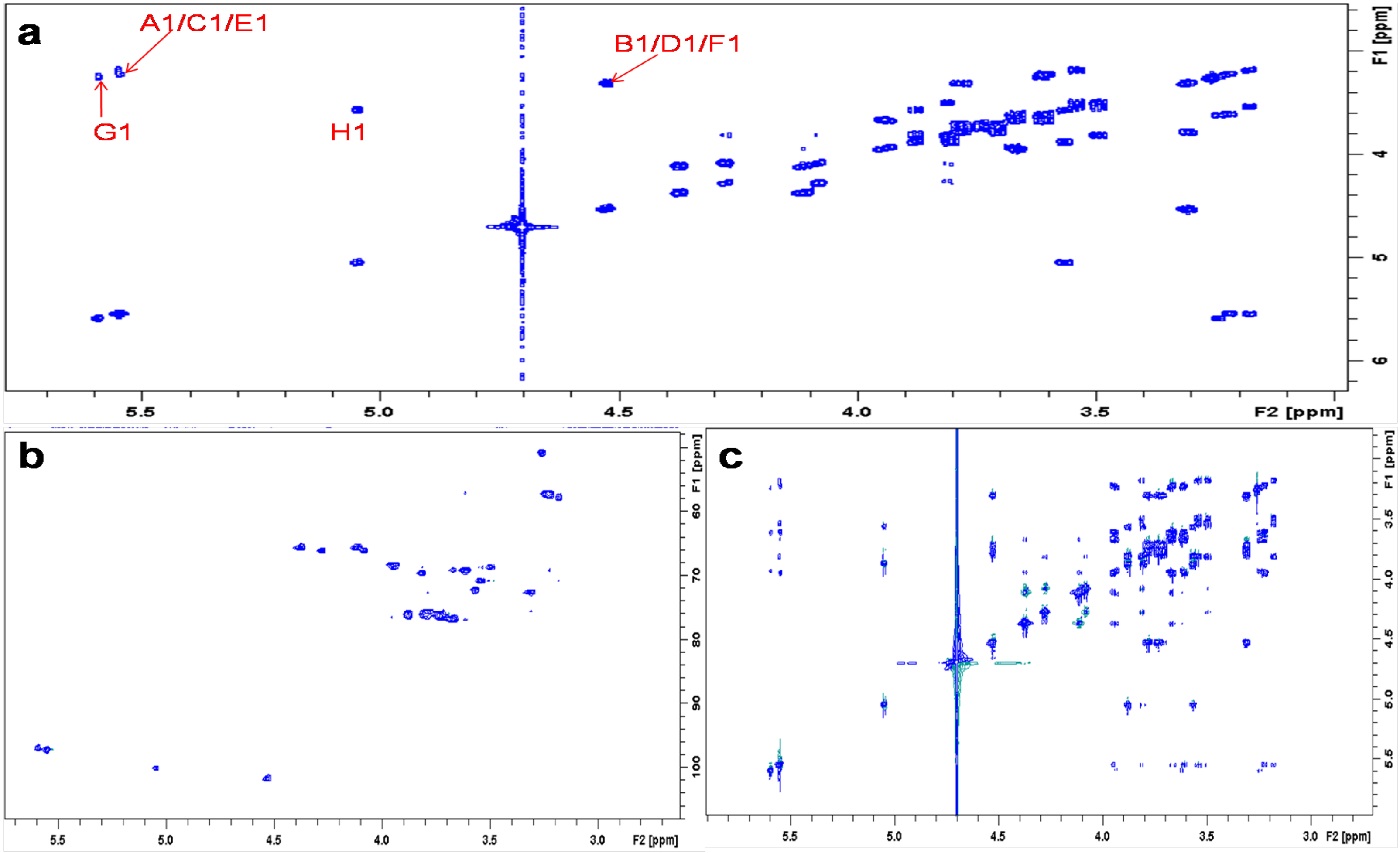

Supplementary Fig. S105. 2D NMR COSY (a), HSQC (b) and TOCSY (c) spectra of compound **35.**

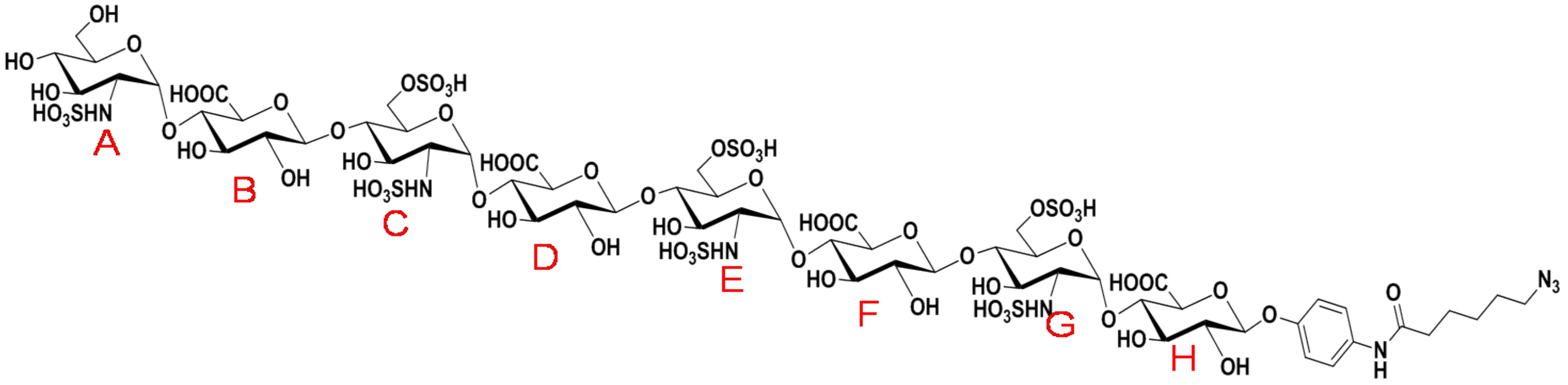

	1	2	3	4	5	6a	6b
A	5.53	3.13	3.53	3.37	3.64	3.65	3.71
B	4.52	3.30	3.78	3.72	3.79	----------	----------
C	5.54	3.21	3.60	3.65	3.93	4.10	4.36
D	4.52	3.30	3.78	3.72	3.79	----------	----------
E	5.54	3.21	3.60	3.65	3.93	4.10	4.36
F	4.52	3.30	3.78	3.72	3.79	----------	----------
G	5.59	3.24	3.61	3.67	3.93	4.11	4.37
H	5.05	3.56	3.86	3.81	3.87	----------	----------

Supplementary Fig. S106. ^{1}H NMR chemical shift assignments (in ppm) of compound **36**. The structure of compound **36** is shown. The protons of *p*NA-N_3 are not listed in the table.

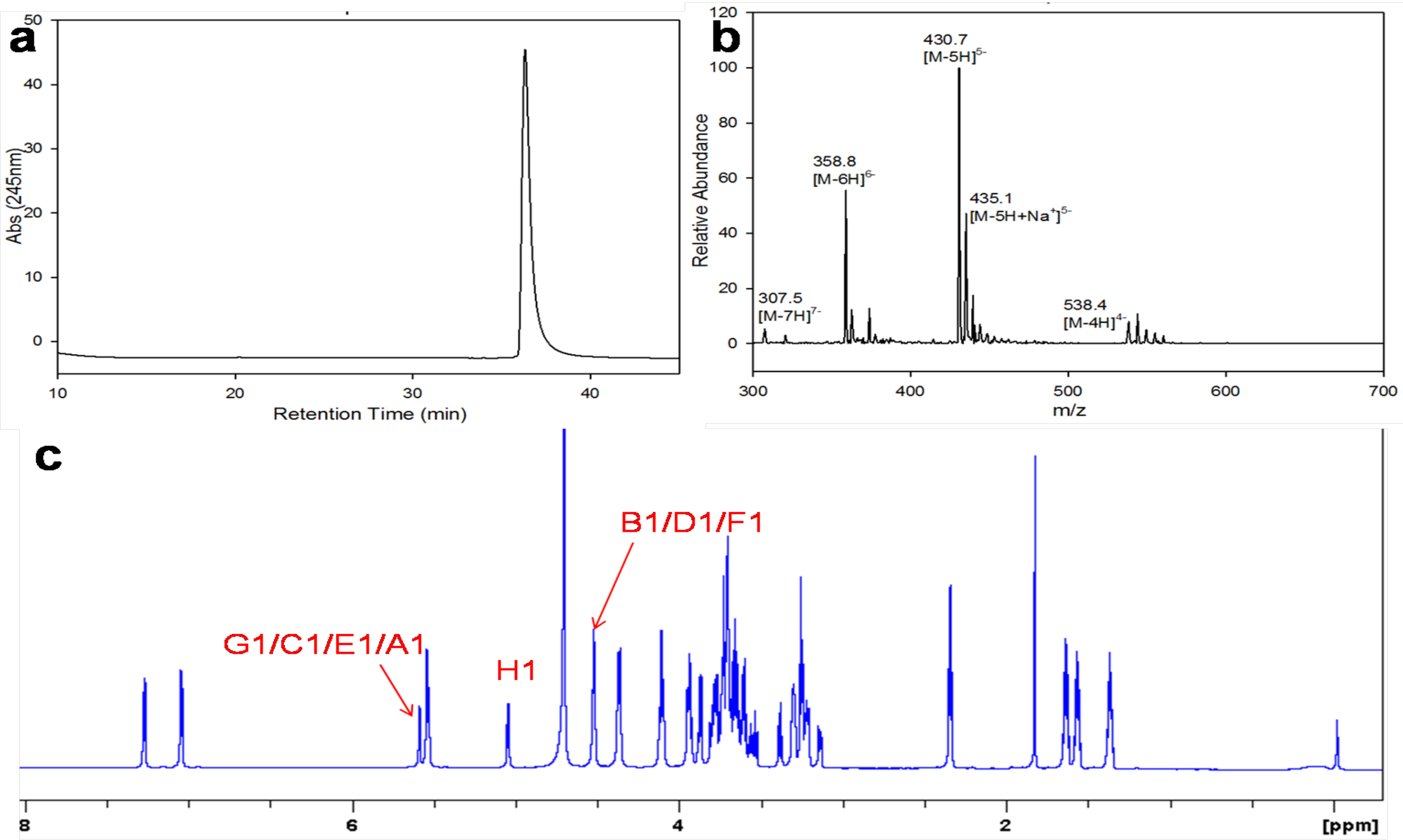

Supplementary Fig. S107. HPLC analysis (a), MS spectrum (b) and ^{1}H NMR spectrum (c) of compound **36**.

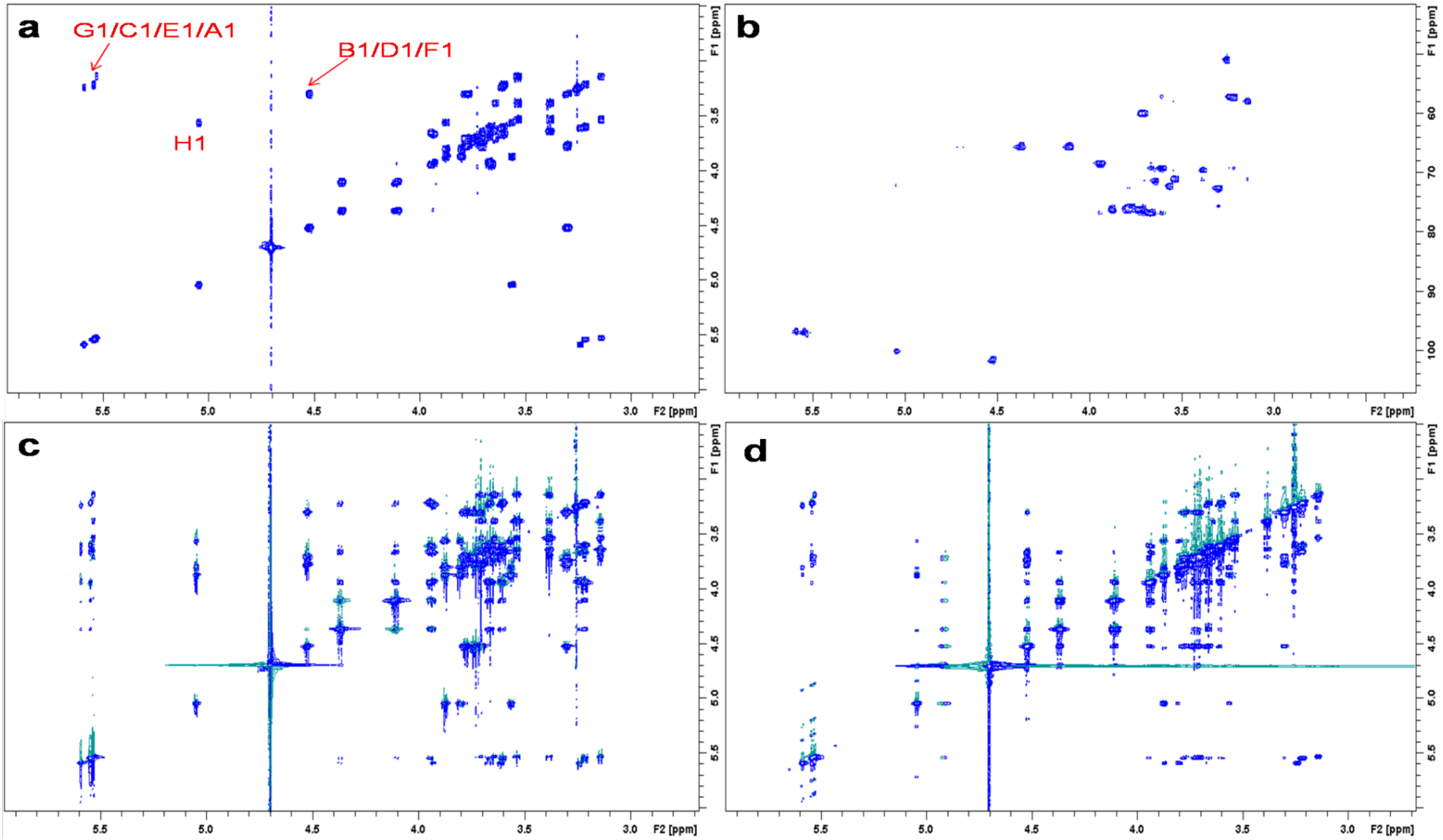

Supplementary Fig. S108. 2D NMR COSY (a), HSQC (b), TOCSY (c) and NOE (d) spectra of compound **36.**

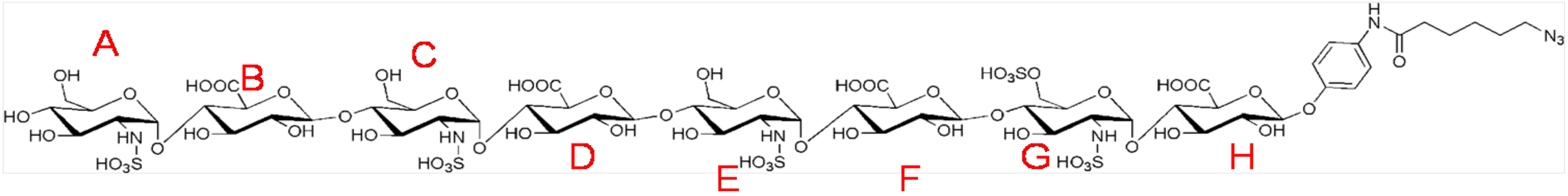

	1	2	3	4	5	6a	6b
A	5.54	3.15	3.50	3.41	3.57	3.61	3.71
B	4.48	3.34	3.75	3.74	3.86	----------	----------
C	5.51	3.17	3.62	3.68	3.70	3.72	3.81
D	4.48	3.34	3.75	3.74	3.86	----------	----------
E	5.51	3.17	3.62	3.68	3.70	3.72	3.81
F	4.56	3.30	3.80	3.75	3.90	----------	----------
G	5.56	3.24	3.62	3.67	3.88	4.13	4.36
H	5.06	3.58	3.88	3.83	3.99	----------	----------

Supplementary Fig. S109. ^{1}H NMR chemical shift assignments (in ppm) of compound **37**. The structure of compound **37** is shown. The protons of *p*NA-N_3 are not listed in the table.

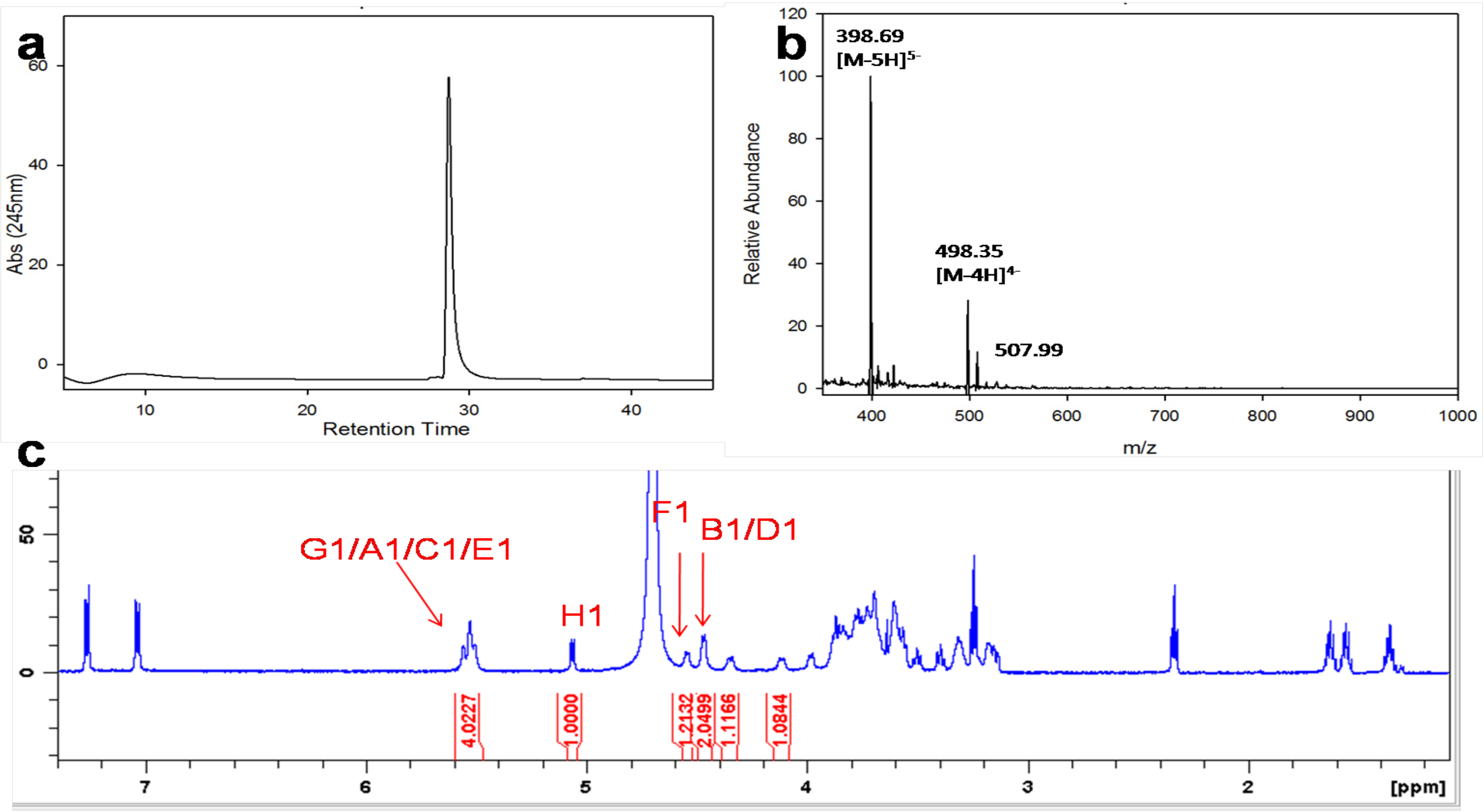

Supplementary Fig. S110. HPLC analysis (a), MS spectrum (b) and ^{1}H NMR spectrum (c) of compound **37**.

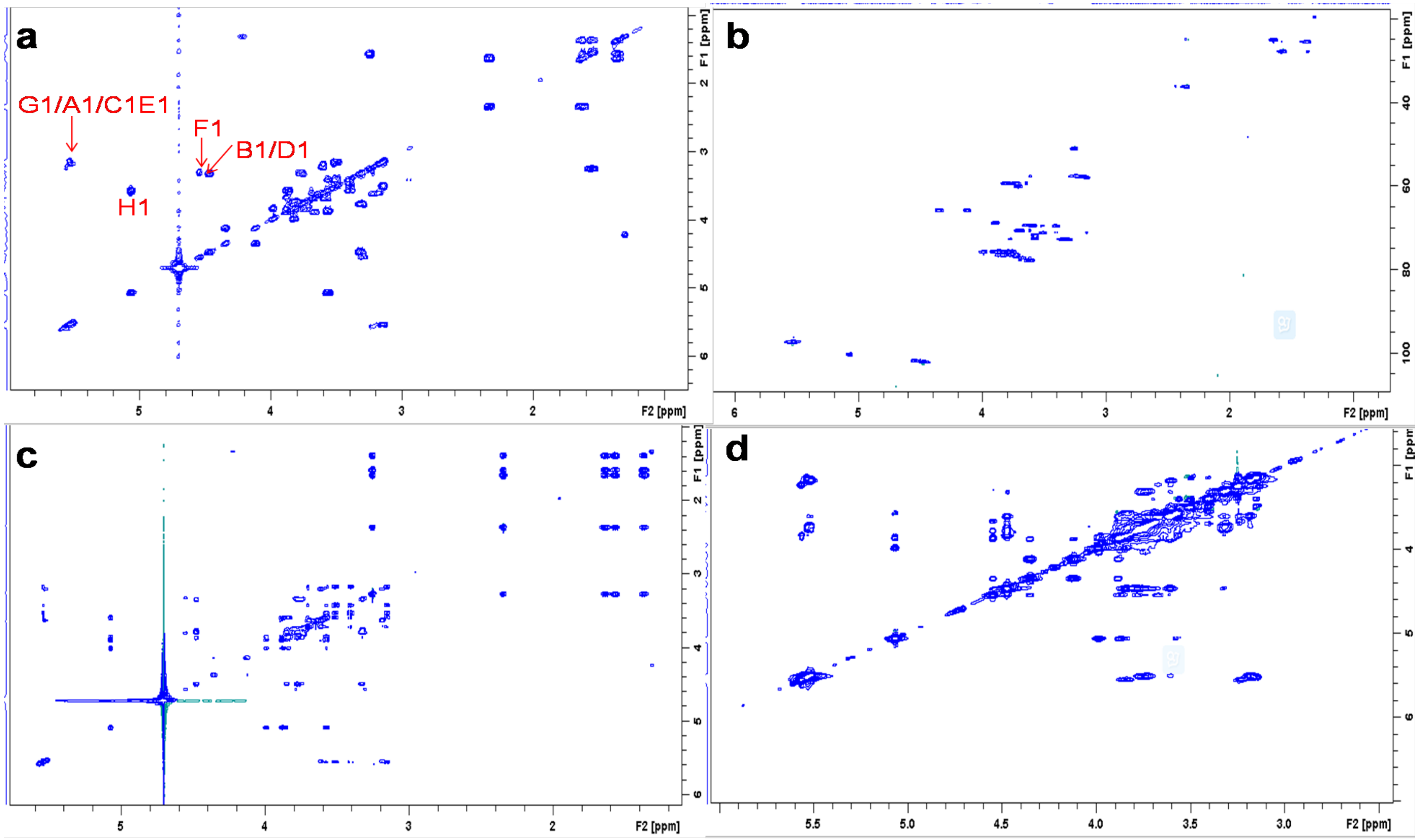

Supplementary Fig. S111. 2D NMR COSY (a), HSQC (b), TOCSY (c) and NOE (d) spectra of compound **37.**

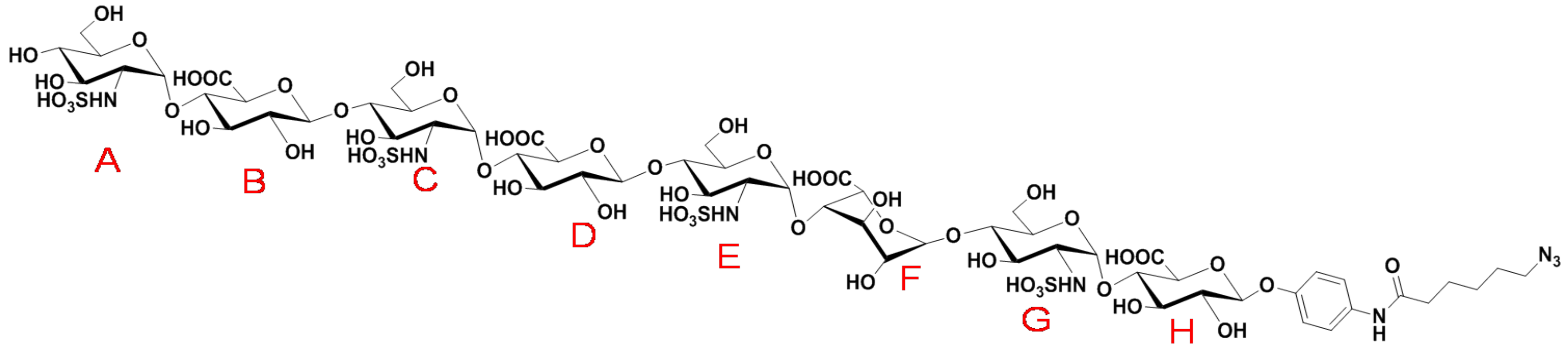

	1	2	3	4	5	6a	6b
A	5.55	3.15	3.52	3.39	3.62	3.62	3.70
B	4.44	3.31	3.76	3.71	3.76	----------	----------
C	5.53	3.17	3.60	3.64	3.64	3.64	3.72
D	4.44	3.31	3.76	3.71	3.76	----------	----------
E	5.28	3.13	3.59	3.75	3.70	3.70	3.75
F	4.88	3.64	4.02	3.96	4.71	----------	----------
G	5.53	3.17	3.60	3.64	3.64	3.64	3.72
H	5.04	3.55	3.85	3.77	3.87	----------	----------

Supplementary Fig. S112. ^{1}H NMR chemical shift assignments (in ppm) of compound **38**. The structure of compound **38** is shown. The protons of *p*NA-N_3 are not listed in the table.

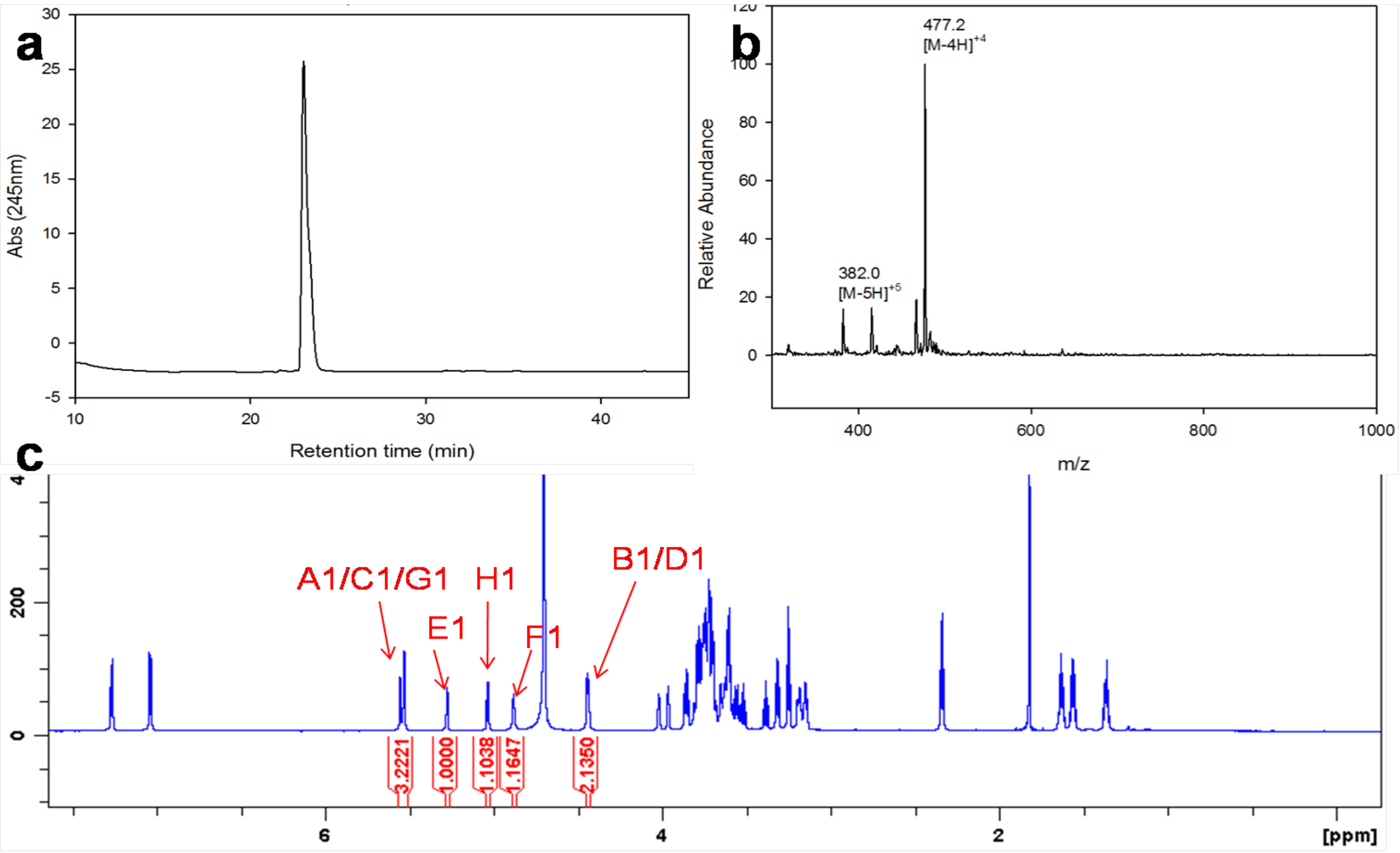

Supplementary Fig. S113. HPLC analysis (a), MS spectrum (b) and ^{1}H NMR spectrum (c) of compound **38**.

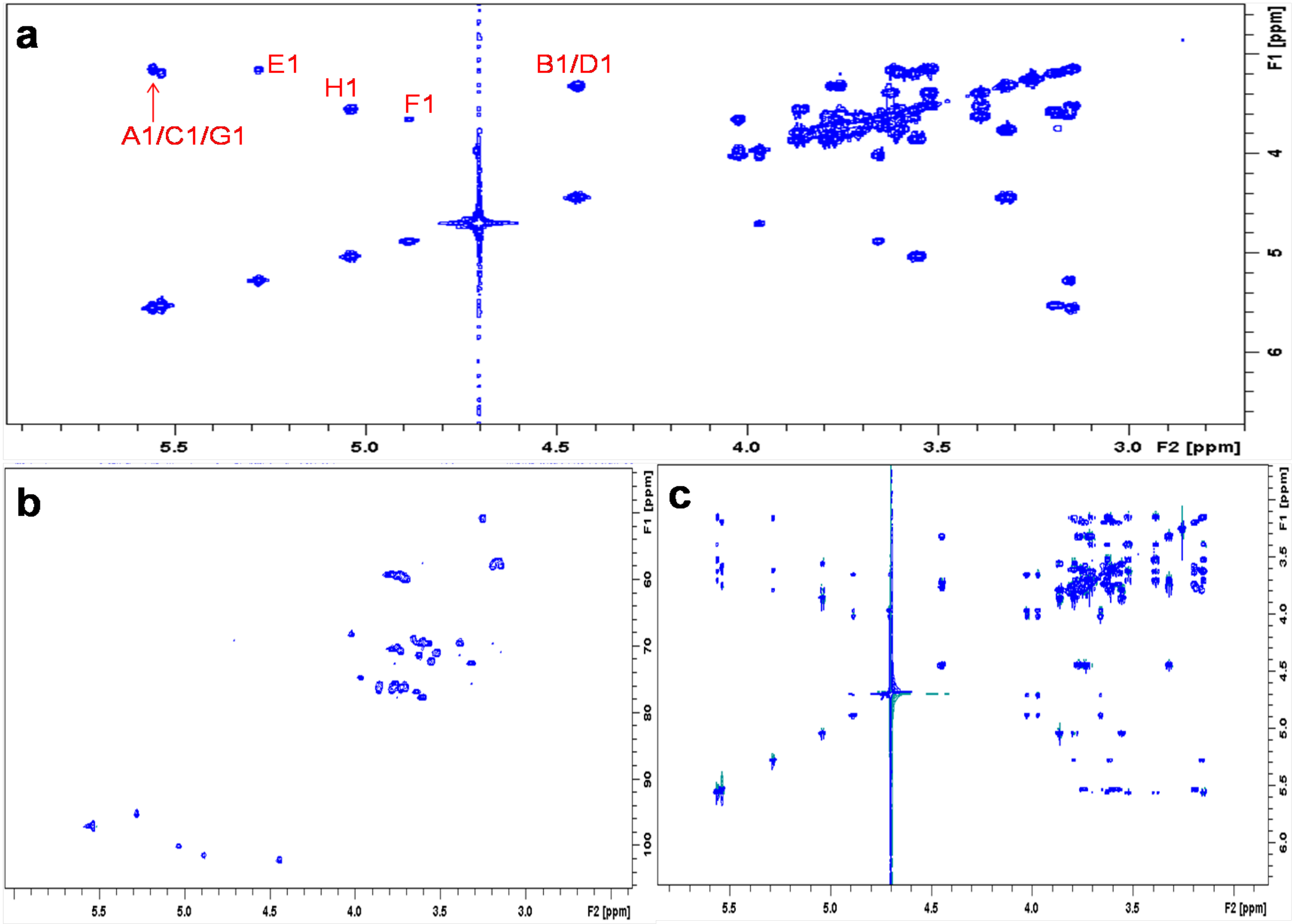

Supplementary Fig. S114. 2D NMR COSY (a), HSQC (b) and TOCSY (c) spectra of compound **38.**

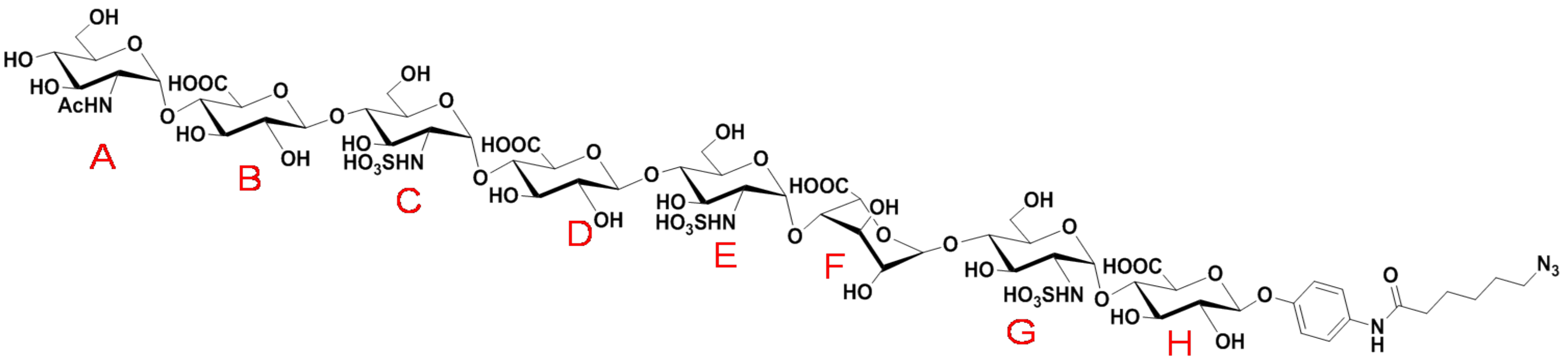

	1	2	3	4	5	6a	6b
A	5.46	3.91	3.76	3.51	3.78	3.84	3.90
B	4.54	3.39	3.75	3.80	3.85	----------	----------
C	5.65	3.31	3.70	3.75	3.72	3.84	3.92
D	4.57	3.44	3.88	3.80	3.85	----------	----------
E	5.41	3.28	3.73	3.91	3.76	3.83	3.87
F	5.00	3.77	4.14	4.10	4.81	----------	----------
G	5.65	3.31	3.70	3.75	3.72	3.84	3.92
H	5.15	3.68	3.96	3.92	3.97	----------	----------

Supplementary Fig. S115. ^{1}H NMR chemical shift assignments (in ppm) of compound **39**. The structure of compound **39** is shown. The protons of *p*NA-N_3 are not listed in the table.

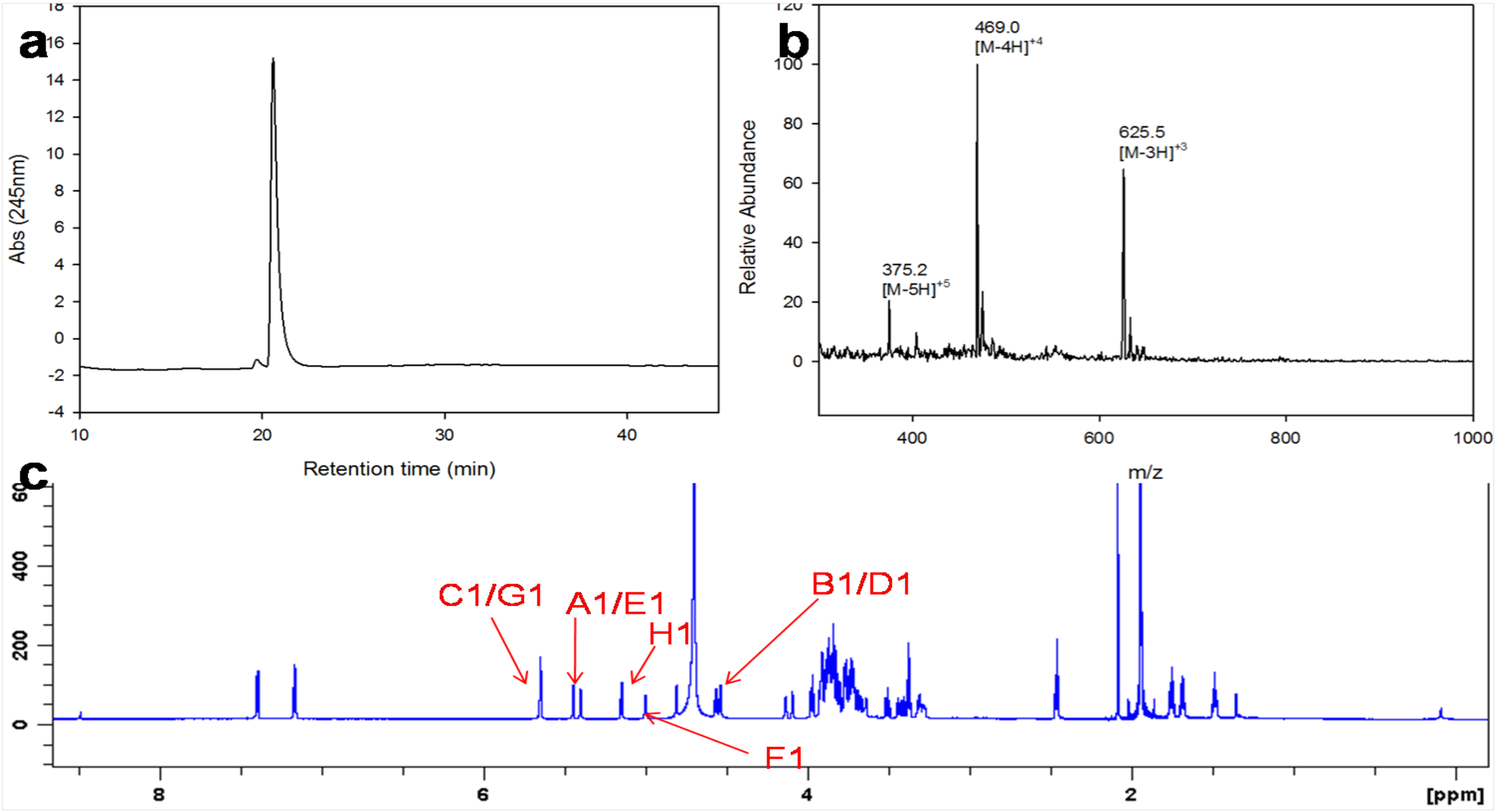

Supplementary Fig. S116. HPLC analysis (a), MS spectrum (b) and ^{1}H NMR spectrum (c) of compound **39**.

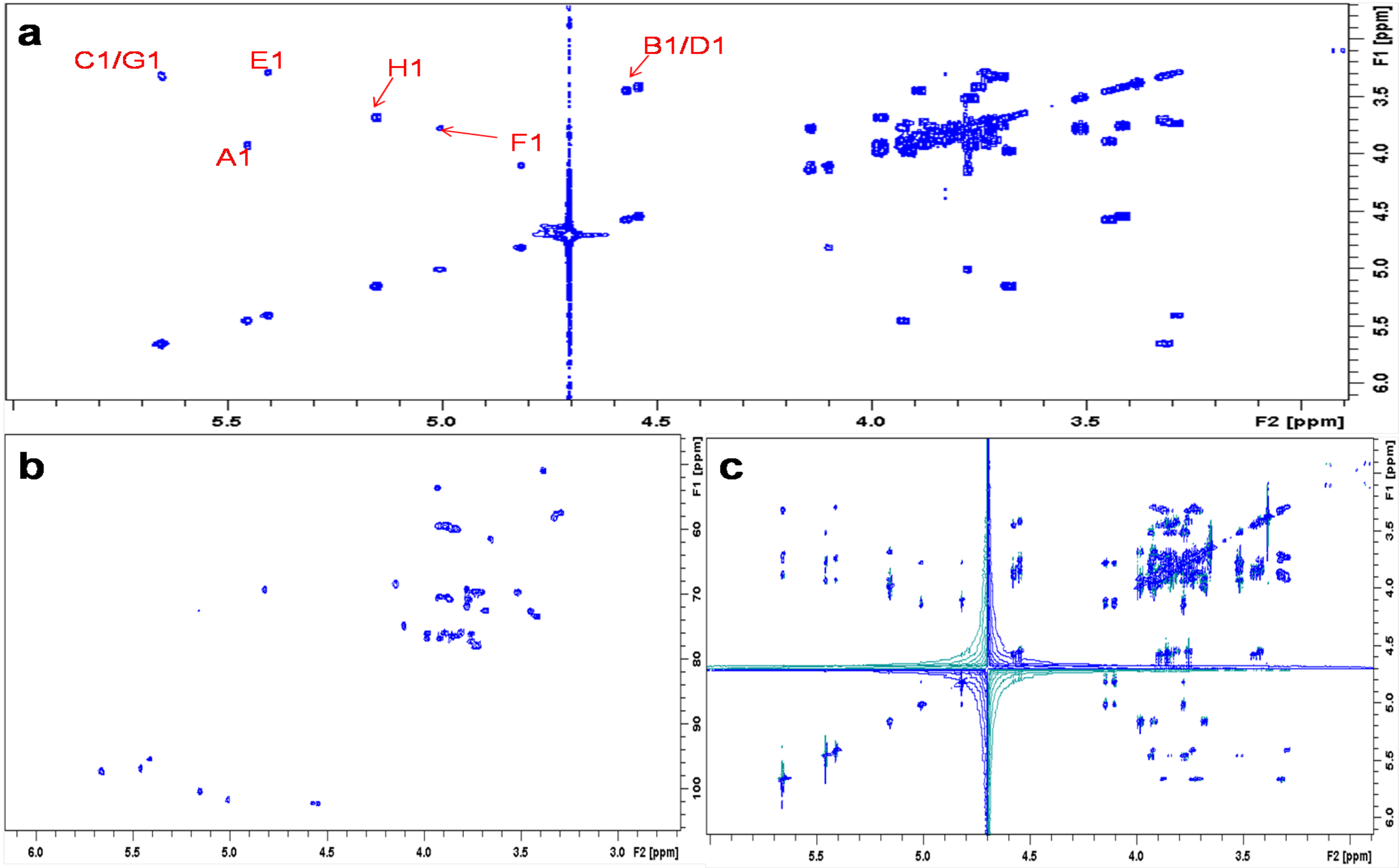

Supplementary Fig. S117. 2D NMR COSY (a), HSQC (b) and TOCSY (c) spectra of compound **39.**

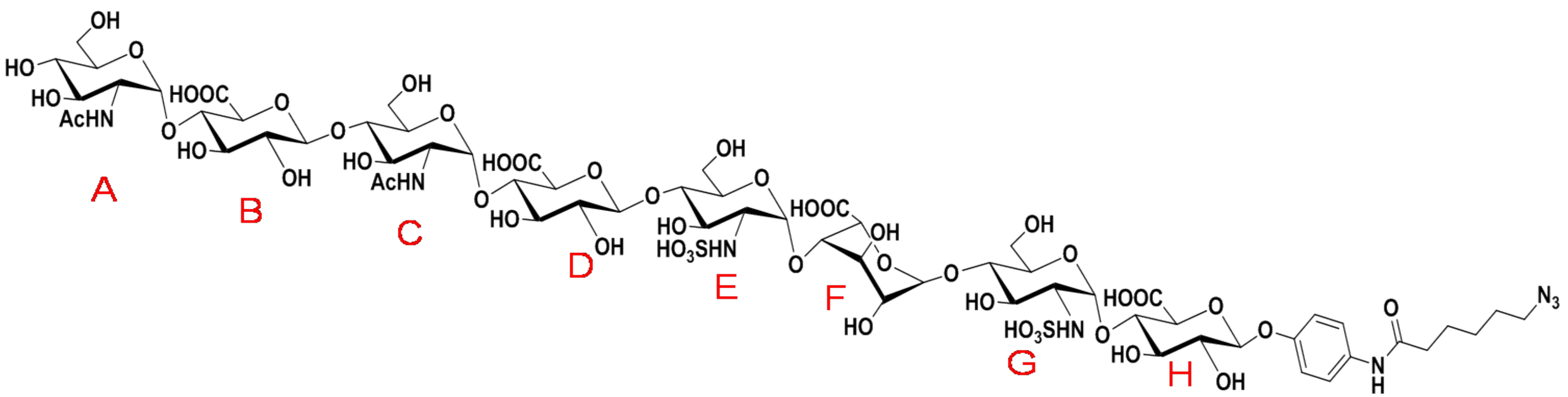

	1	2	3	4	5	6a	6b
A	5.45	3.90	3.76	3.50	3.78	3.84	3.89
B	4.53	3.40	3.73	3.77	3.83	----------	----------
C	5.41	3.90	3.76	3.70	3.80	3.84	3.89
D	4.53	3.40	3.73	3.77	3.83	----------	----------
E	5.39	3.27	3.73	3.89	3.87	3.84	3.92
F	4.99	3.75	4.13	4.06	4.79	----------	----------
G	5.64	3.31	3.67	3.73	3.84	3.84	3.92
H	5.14	3.66	3.96	3.90	3.98	----------	----------

Supplementary Fig. S118. ^{1}H NMR chemical shift assignments (in ppm) of compound **40**. The structure of compound **40** is shown. The protons of *p*NA-N_3 are not listed in the table.

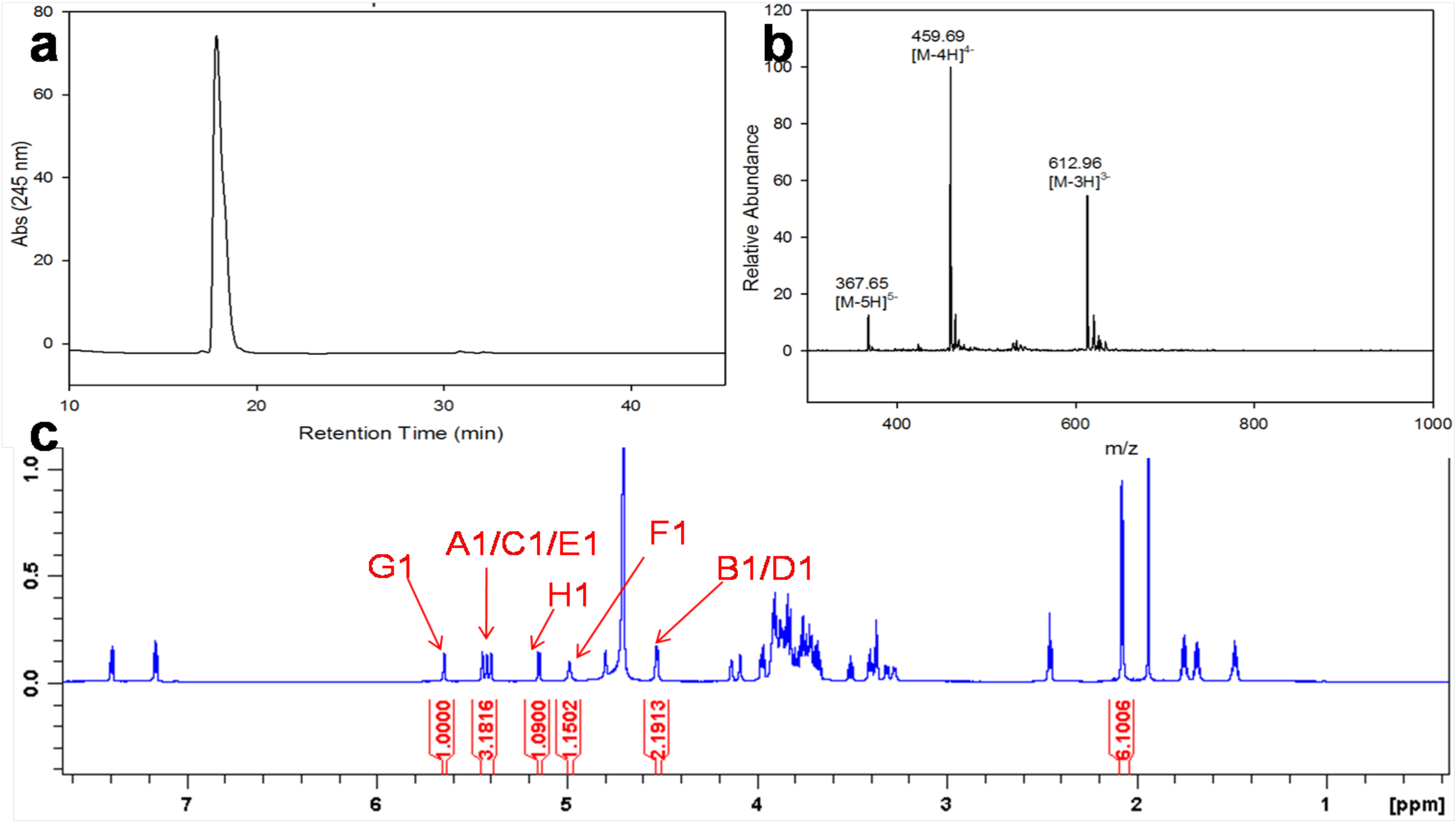

Supplementary Fig. S119. HPLC analysis (a), MS spectrum (b) and ^{1}H NMR spectrum (c) of compound **40**.

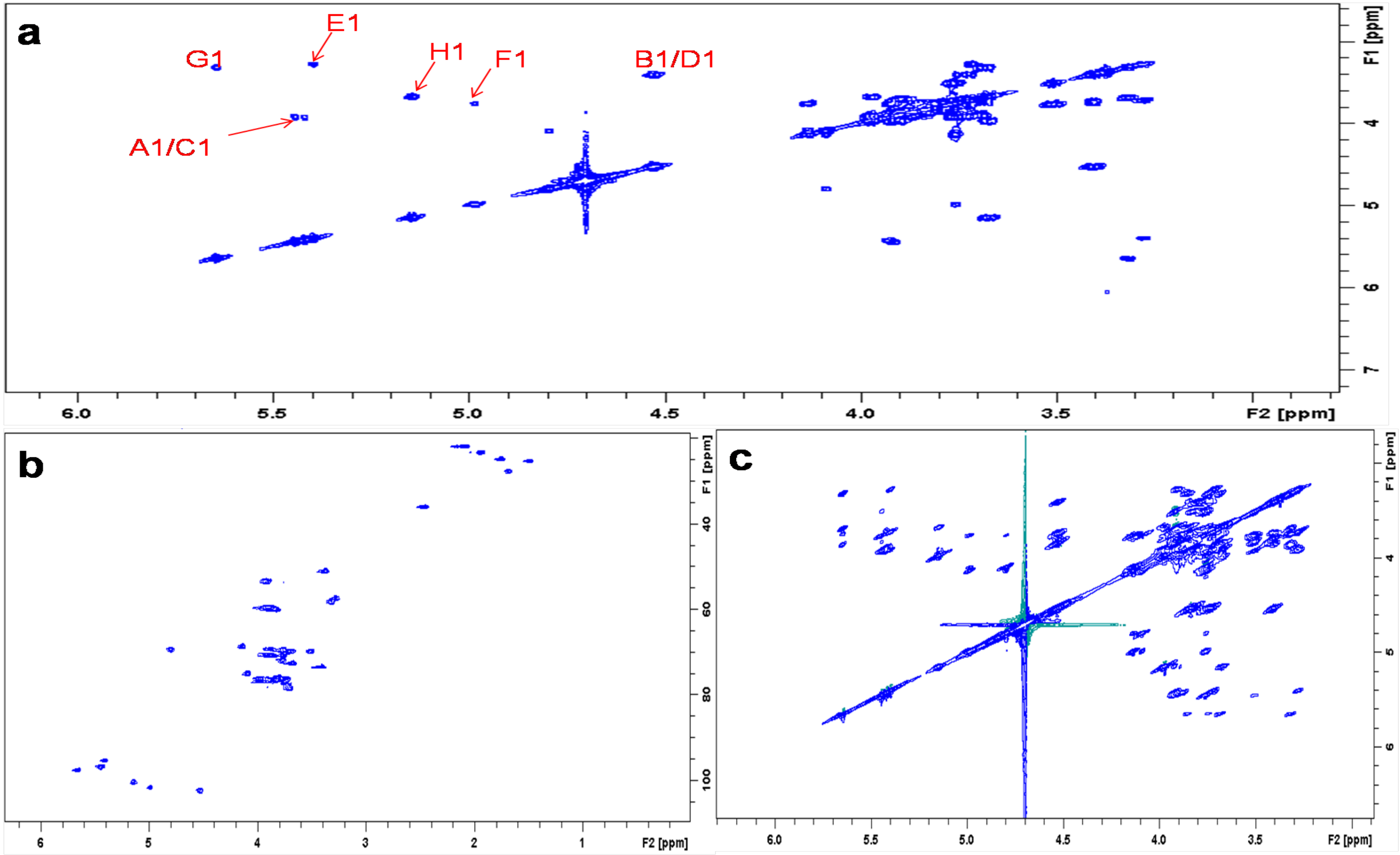

Supplementary Fig. S120. 2D NMR COSY (a), HSQC (b) and TOCSY (c) spectra of compound **40.**

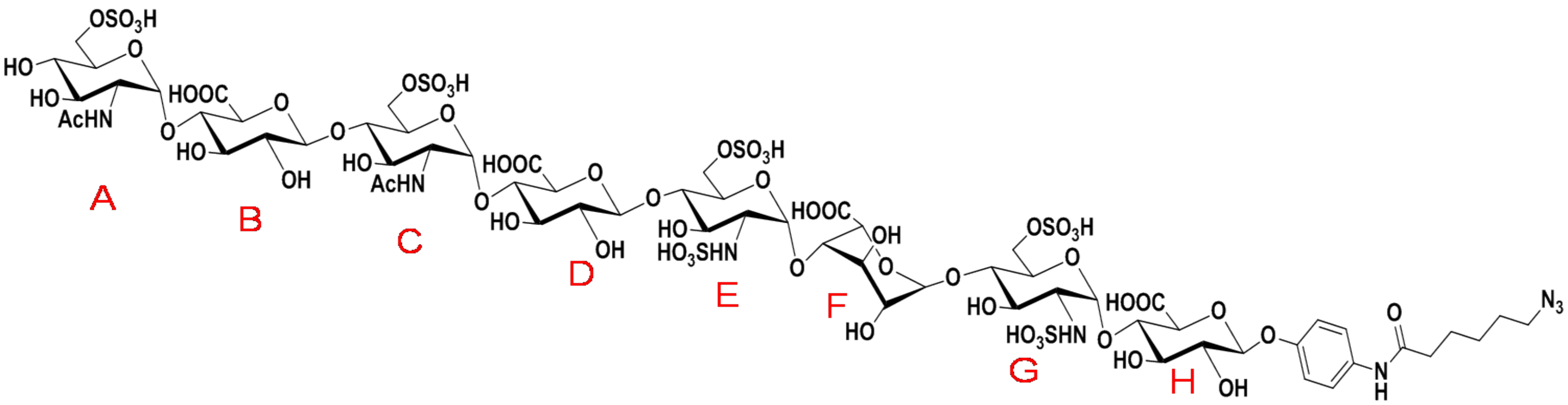

	1	2	3	4	5	6a	6b
A	5.28	3.83	3.66	3.51	3.78	4.11	4.22
B	4.54	3.29	3.68	3.72	3.95	----------	----------
C	5.28	3.85	3.75	3.66	3.88	4.08	4.34
D	4.54	3.29	3.68	3.72	3.95	----------	----------
E	5.26	3.18	3.62	3.85	3.75	4.11	4.37
F	5.00	3.75	4.12	4.00	5.06	----------	----------
G	5.52	3.22	3.61	3.75	3.86	4.11	4.22
H	5.10	3.59	3.88	3.86	4.09	----------	----------

Supplementary Fig. S121. ^{1}H NMR chemical shift assignments (in ppm) of compound **41**. The structure of compound **41** is shown. The protons of *p*NA-N_3 are not listed in the table.

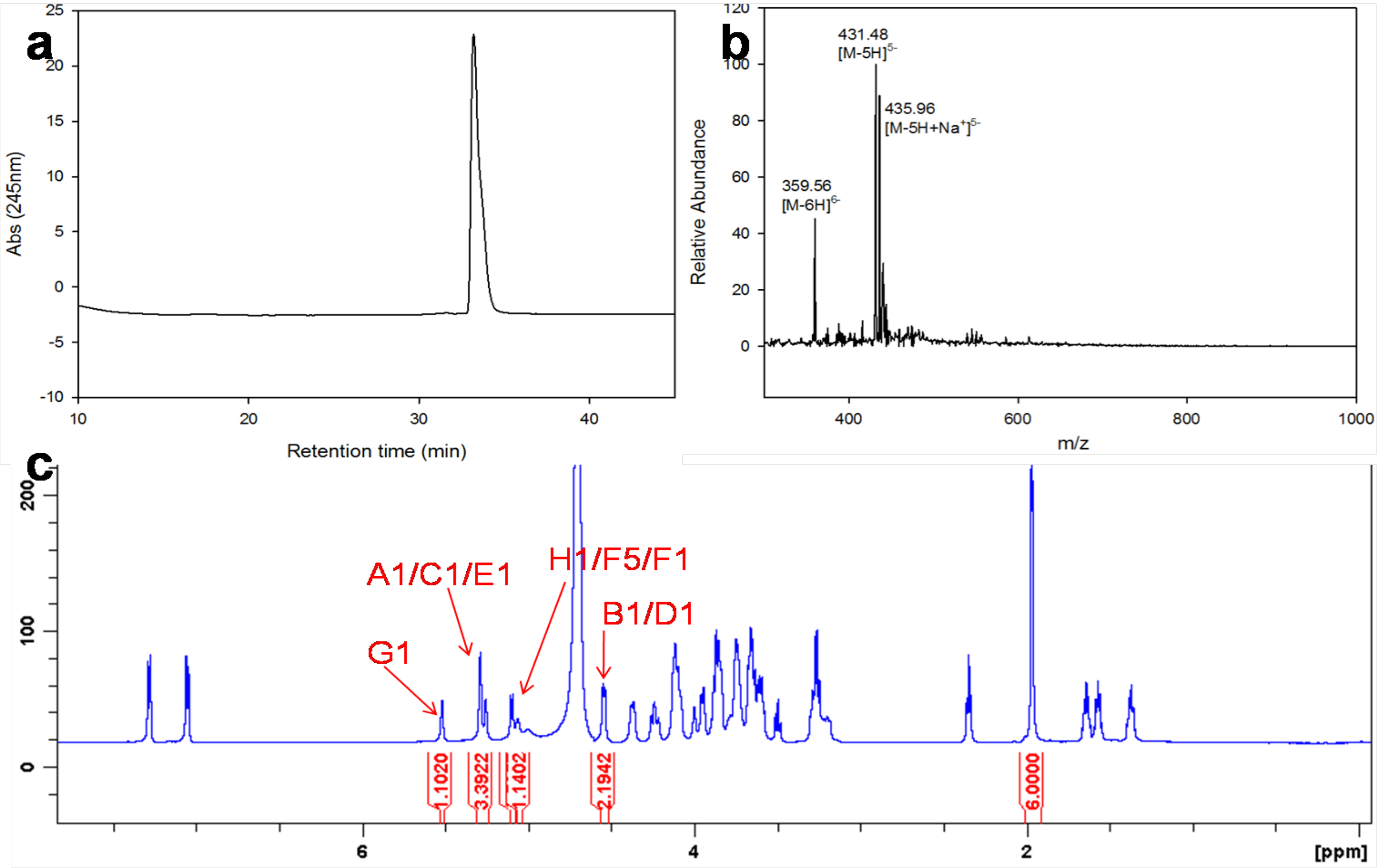

Supplementary Fig. S122. HPLC analysis (a), MS spectrum (b) and ^{1}H NMR spectrum (c) of compound **41**.

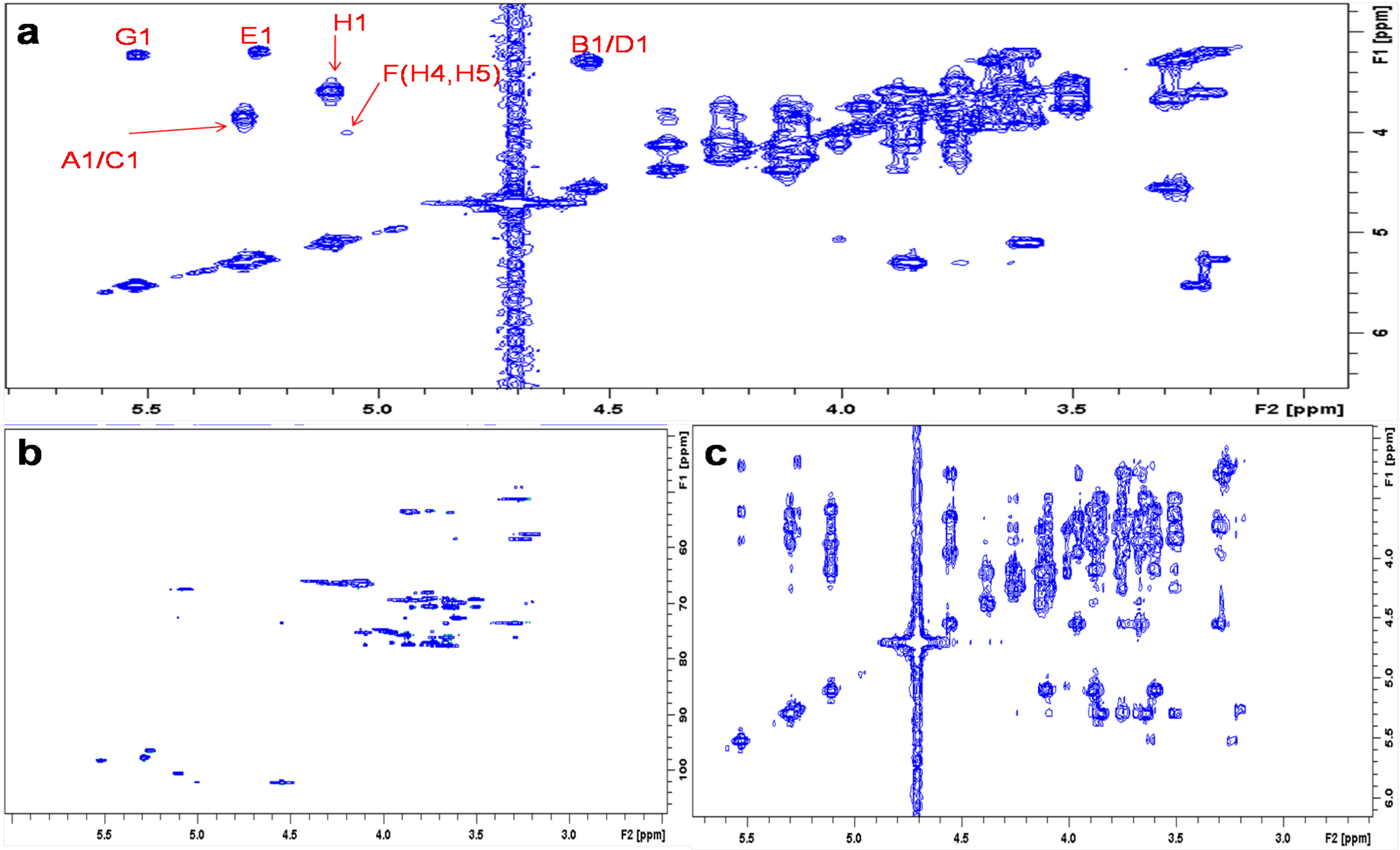

Supplementary Fig. S123. 2D NMR COSY (a), HSQC (b) and TOCSY (c) spectra of compound **41.**

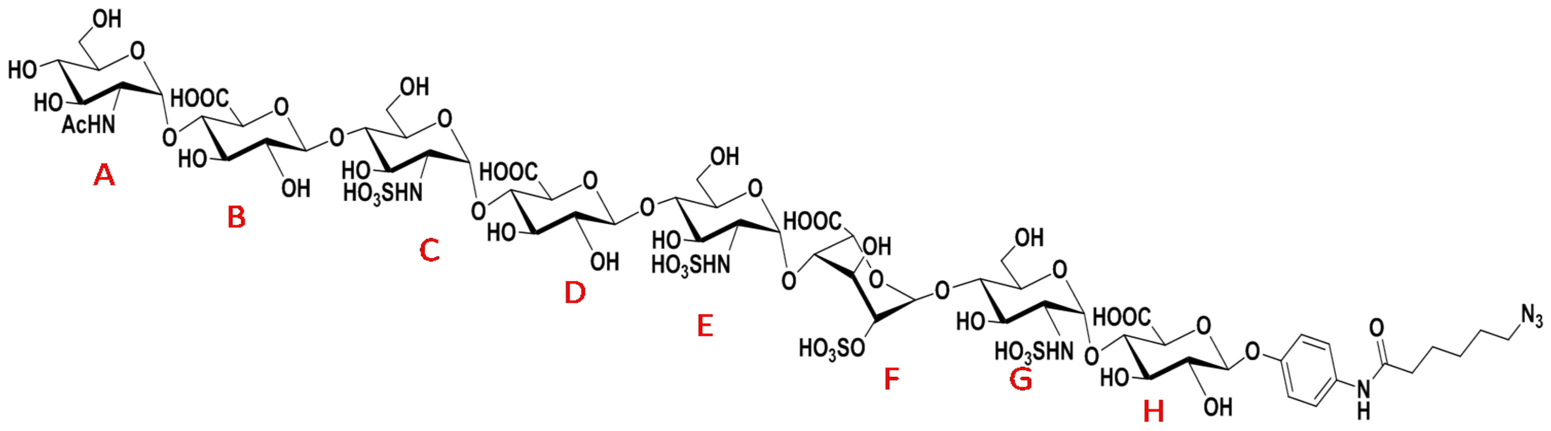

	1	2	3	4	5	6a	6b
A	5.32	3.79	3.62	3.38	3.62	3.70	3.79
B	4.41	3.28	3.62	3.72	3.72	----------	----------
C	5.52	3.17	3.59	3.62	3.70	3.62	3.74
D	4.44	3.31	3.72	3.75	3.75	----------	----------
E	5.21	3.17	3.63	3.65	3.65	3.65	3.80
F	5.17	4.23	4.14	3.94	4.75	----------	----------
G	5.49	3.17	3.57	3.62	3.70	3.62	3.72
H	5.02	3.55	3.80	3.76	3.85	----------	----------

Supplementary Fig. S124. ^{1}H NMR chemical shift assignments (in ppm) of compound **42**. The structure of compound **42** is shown. The protons of *p*NA-N_3 are not listed in the table.

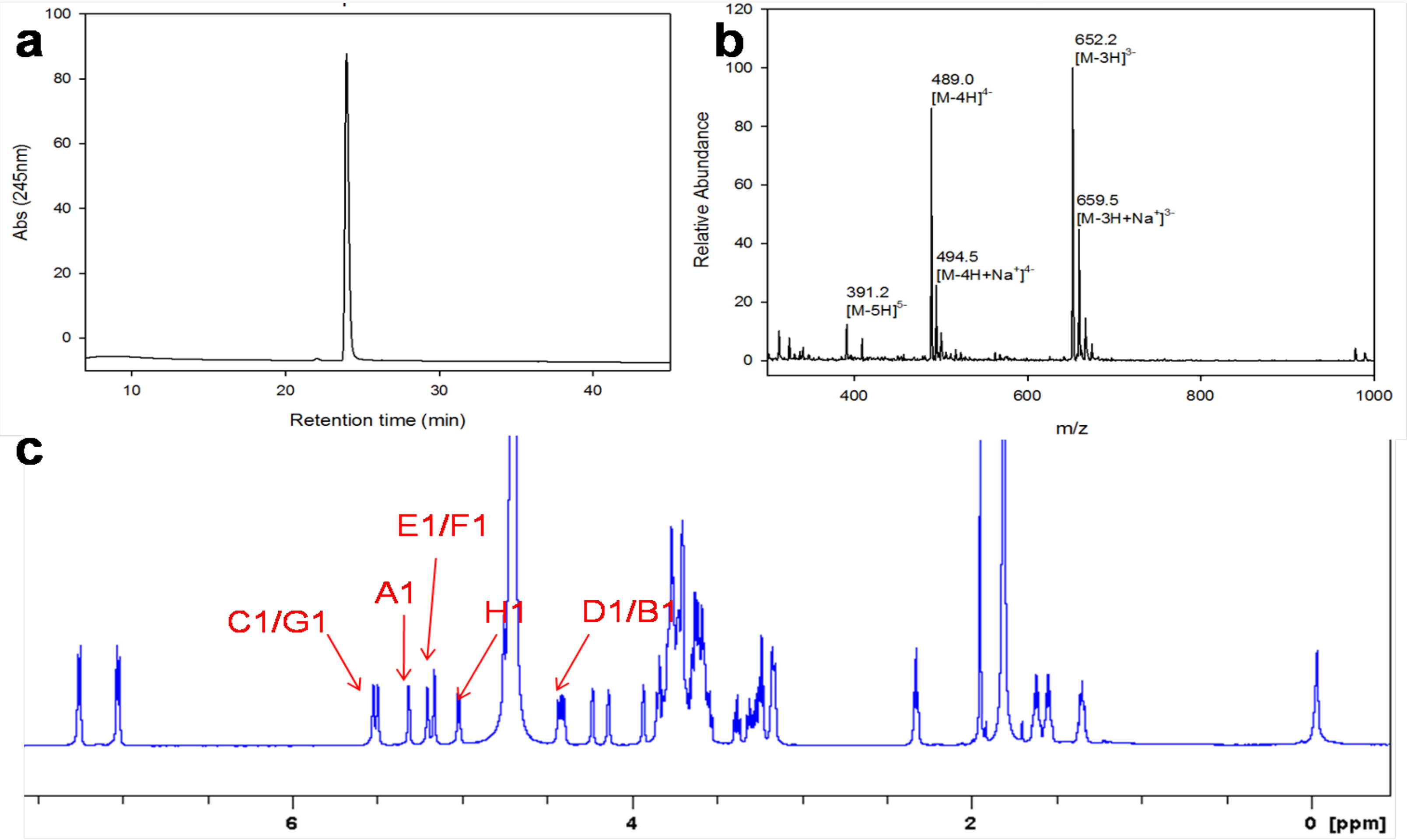

Supplementary Fig. S125. HPLC analysis (a), MS spectrum (b) and ^{1}H NMR spectrum (c) of compound **42**.

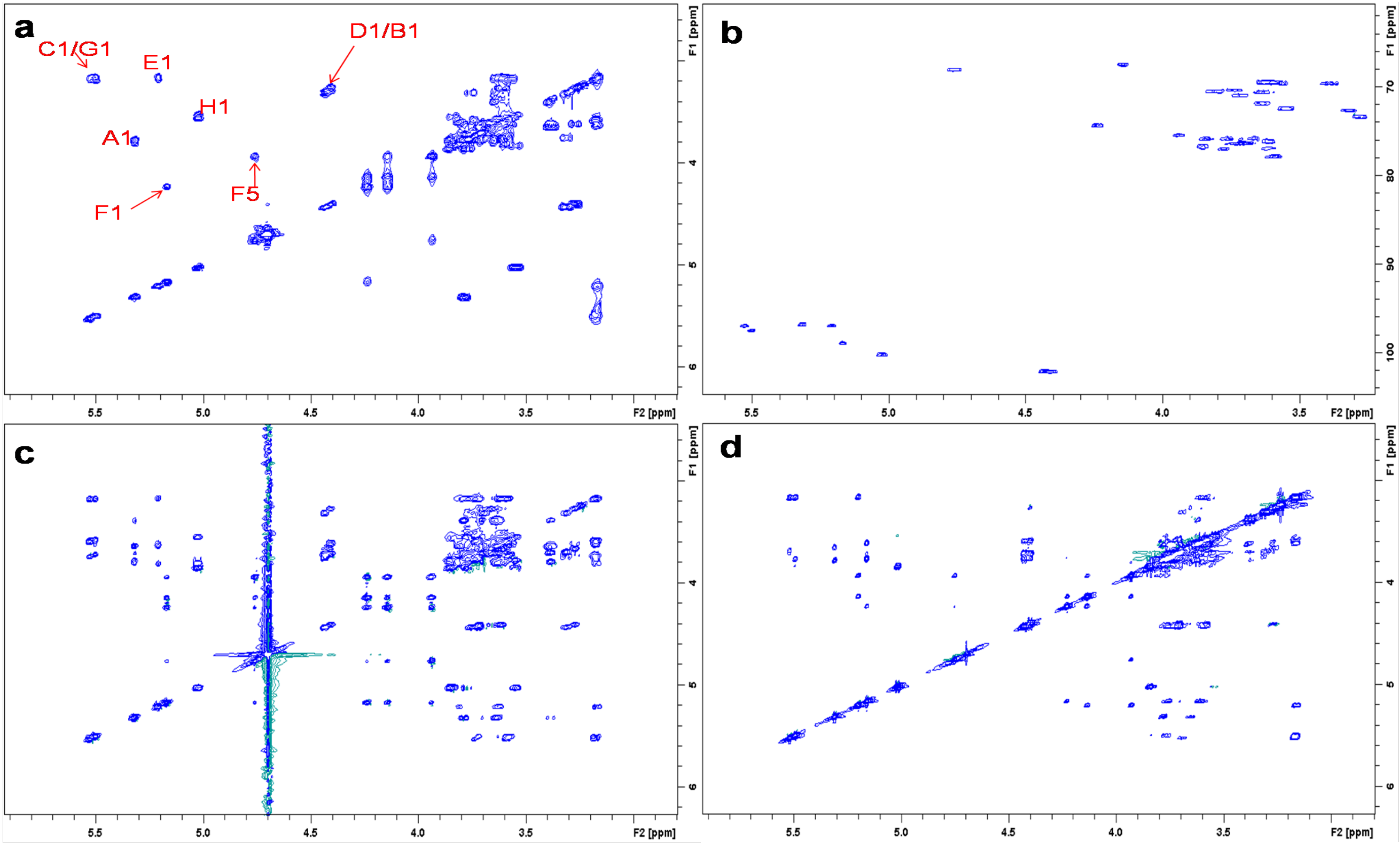

Supplementary Fig. S126. 2D NMR COSY (a), HSQC (b), TOCSY (c) and NOE (d) spectra of compound **42.**

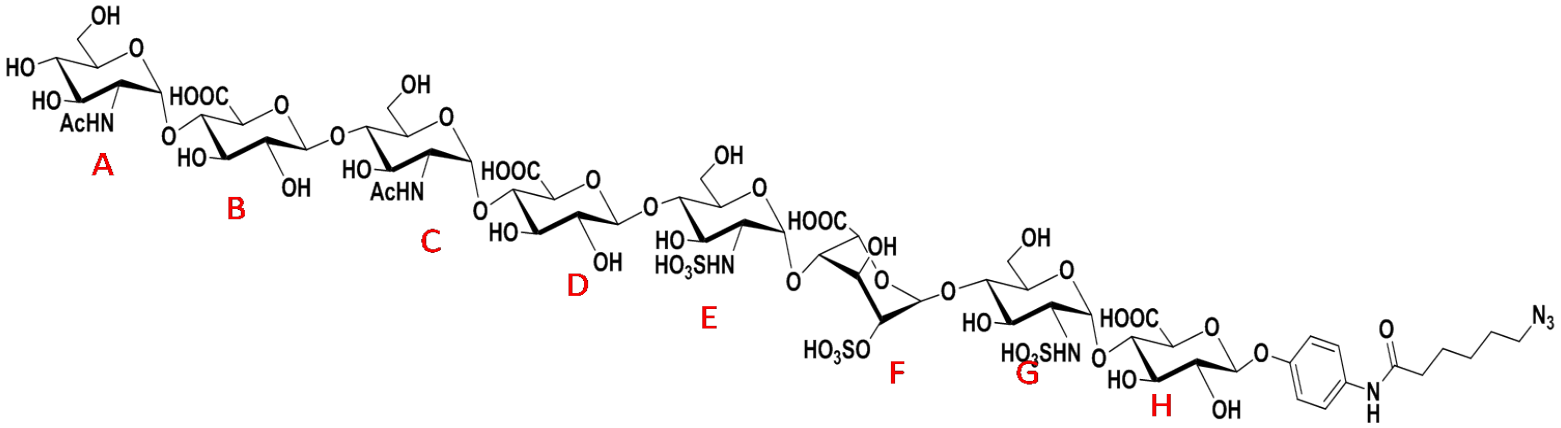

	1	2	3	4	5	6a	6b
A	5.34	3.81	3.65	3.40	3.66	3.71	3.77
B	4.42	3.29	3.62	3.67	3.73	----------	----------
C	5.31	3.81	3.59	3.78	3.78	3.72	3.78
D	4.42	3.29	3.62	3.67	3.73	----------	----------
E	5.23	3.18	3.61	3.67	3.79	3.67	3.82
F	5.19	4.24	4.16	3.96	4.78	----------	----------
G	5.52	3.19	3.59	3.64	3.74	3.64	3.77
H	5.04	3.56	3.84	3.78	3.87	----------	----------

Supplementary Fig. S127. ^{1}H NMR chemical shift assignments (in ppm) of compound **43**. The structure of compound **43** is shown. The protons of *p*NA-N_3 are not listed in the table.

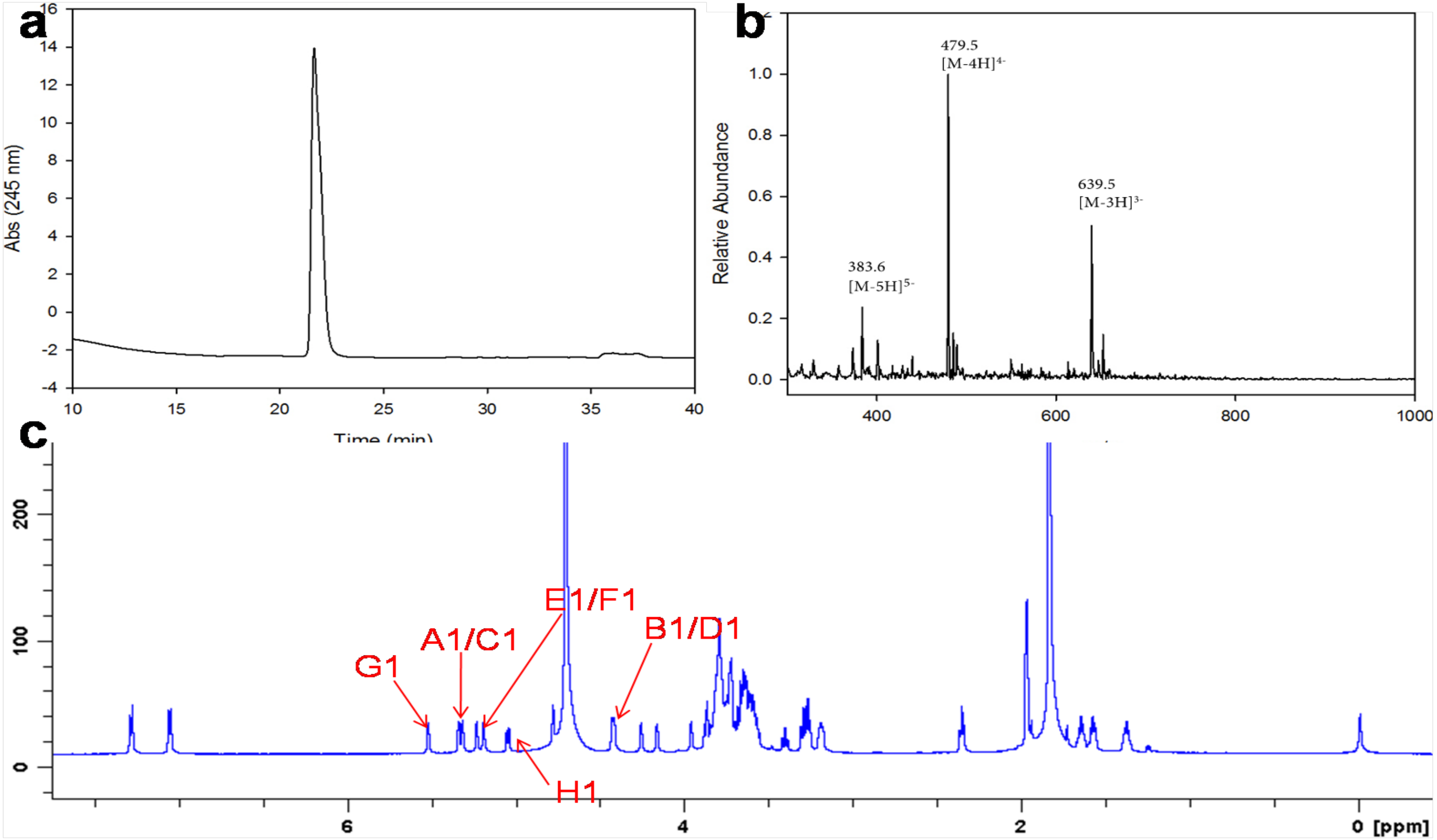

Supplementary Fig. S128. HPLC analysis (a), MS spectrum (b) and ^{1}H NMR spectrum (c) of compound **43**.

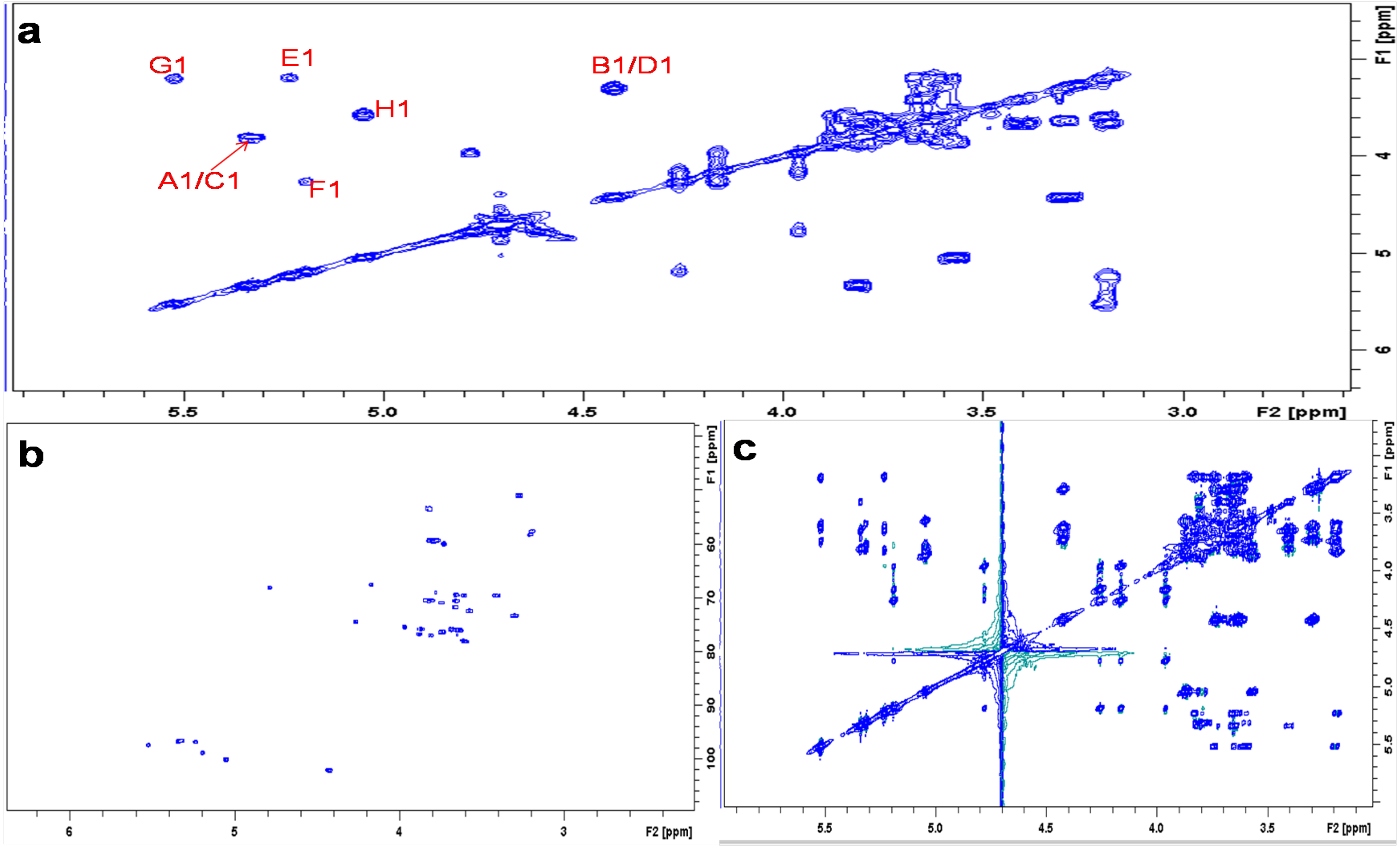

Supplementary Fig. S129. 2D NMR COSY (a), HSQC (b) and TOCSY (c) spectra of compound **43.**

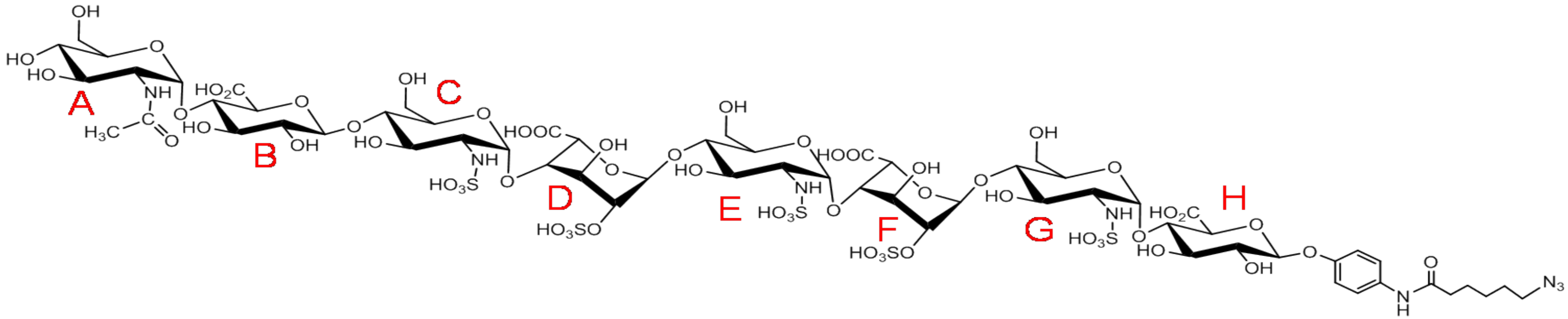

	1	2	3	4	5	6a	6b
A	5.34	3.81	3.65	3.40	3.68	3.71	3.79
B	4.44	3.30	3.63	3.70	3.76	----------	----------
C	5.24	3.18	3.64	3.72	3.72	3.62	3.78
D	5.19	4.25	4.19	3.99	4.93	----------	----------
E	5.24	3.18	3.64	3.72	3.72	3.62	3.78
F	5.19	4.25	4.19	3.99	4.93	----------	----------
G	5.51	3.19	3.60	3.63	3.72	3.63	3.77
H	5.05	3.58	3.87	3.80	3.91	----------	----------

Supplementary Fig. S130. ^{1}H NMR chemical shift assignments (in ppm) of compound **44**. The structure of compound **44** is shown. The protons of *p*NA-N_3 are not listed in the table.

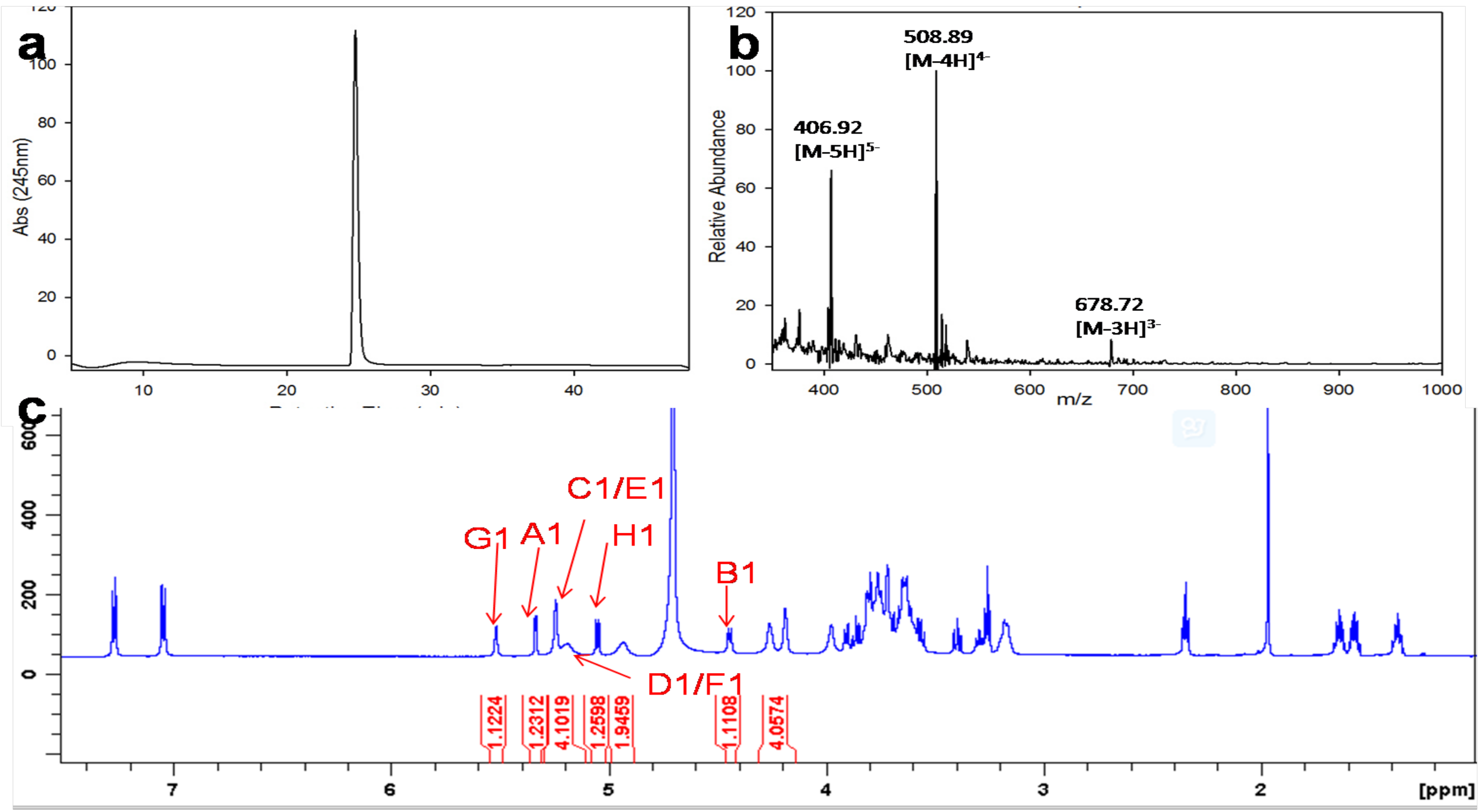

Supplementary Fig. S131. HPLC analysis (a), MS spectrum (b) and ^{1}H NMR spectrum (c) of compound **44**.

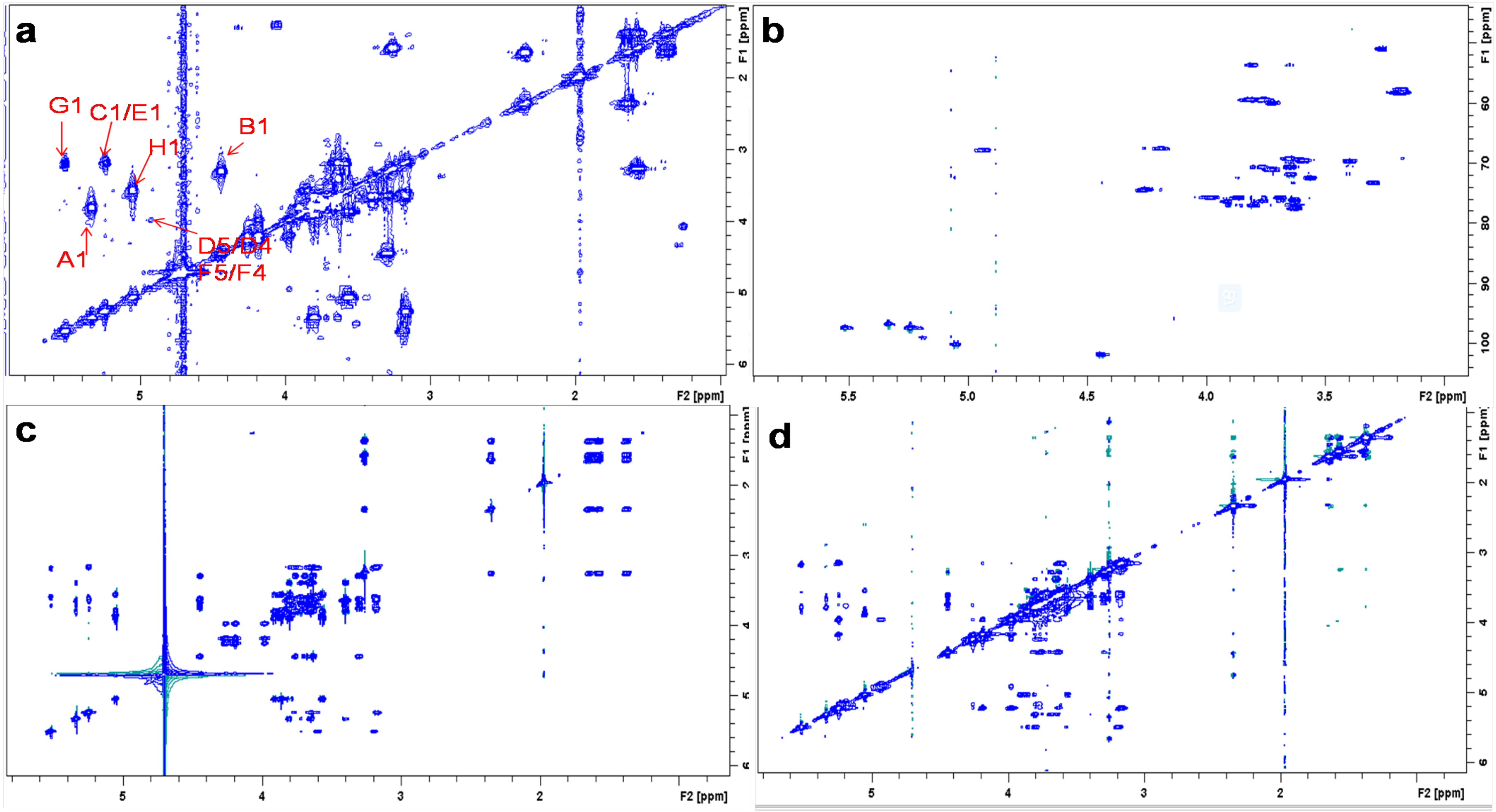

Supplementary Fig. S132. 2D NMR COSY (a), HSQC (b), TOCSY (c) and NOE (d) spectra of compound **44.**

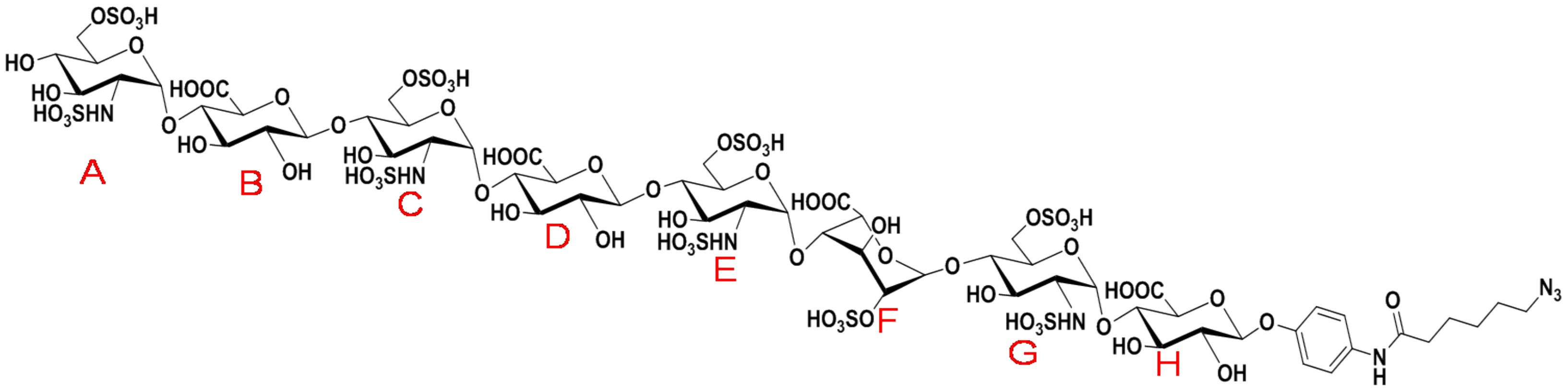

	1	2	3	4	5	6a	6b
A	5.54	3.16	3.54	3.46	3.81	4.07	4.27
B	4.52	3.31	3.77	3.73	3.78	----------	----------
C	5.54	3.21	3.62	3.67	3.83	4.10	4..37
D	4.52	3.31	3.77	3.73	3.78	----------	----------
E	5.34	3.20	3.62	3.67	3.83	4.10	4.37
F	5.17	4.26	4.13	4.03	4.72	----------	----------
G	5.52	3.23	3.60	3.65	3.95	4.10	4.37
H	5.04	3.56	3.86	3.81	3.88	----------	----------

Supplementary Fig. S133. ^{1}H NMR chemical shift assignments (in ppm) of compound **45**. The structure of compound **45** is shown. The protons of *p*NA-N_3 are not listed in the table.

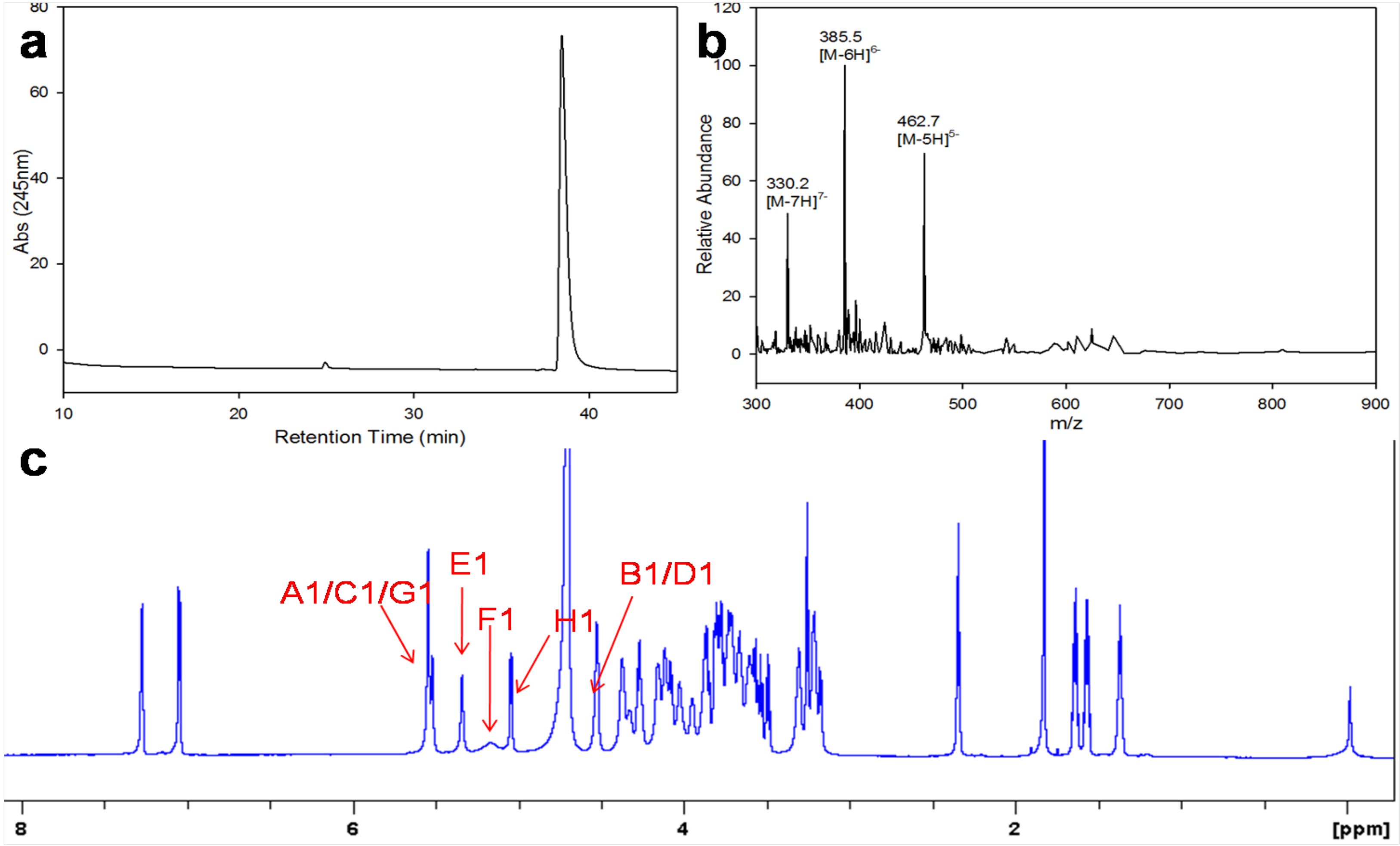

Supplementary Fig. S134. HPLC analysis (a), MS spectrum (b) and ^{1}H NMR spectrum (c) of compound **45**.

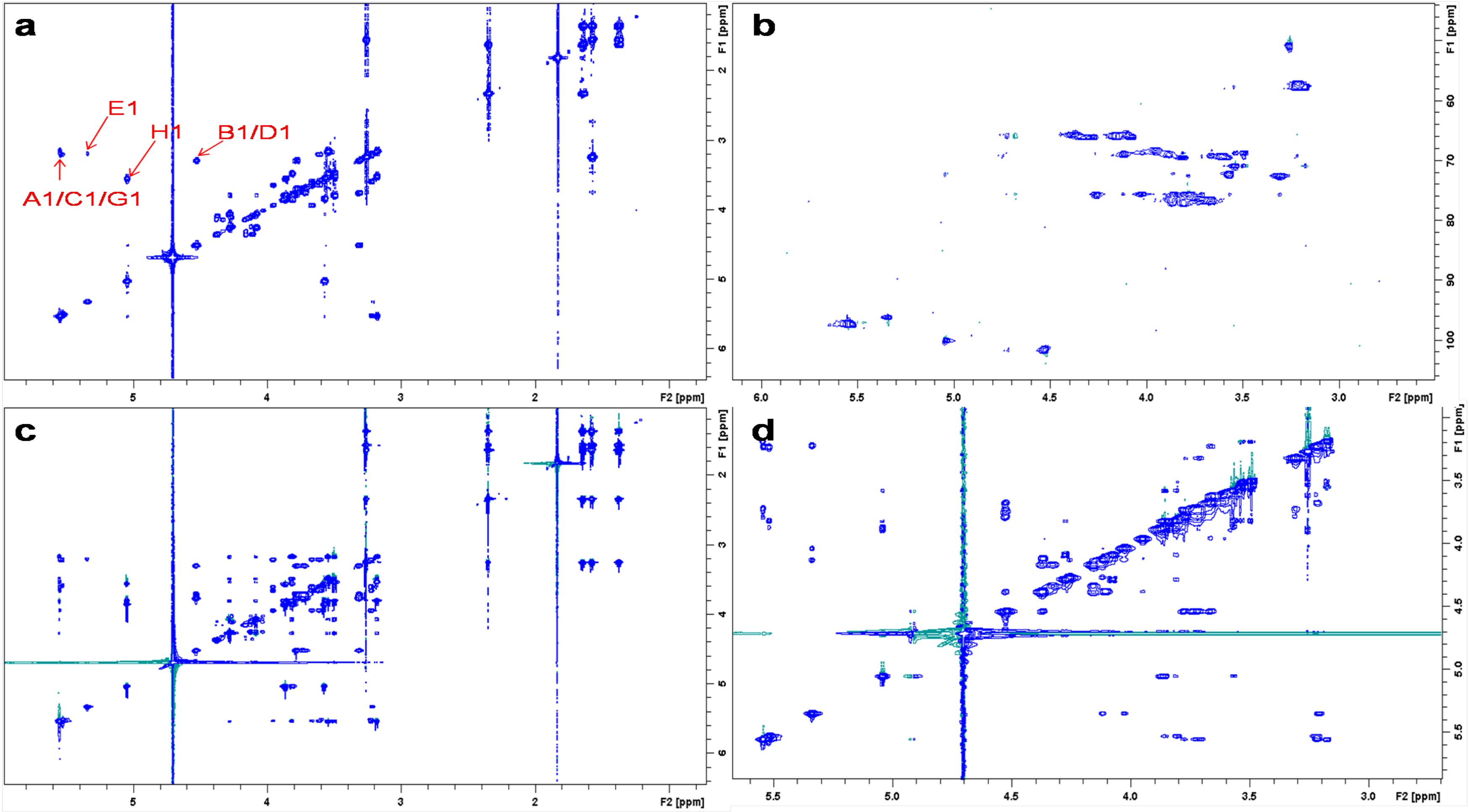

Supplementary Fig. S135. 2D NMR COSY (a), HSQC (b), TOCSY (c) and NOE (d) spectra of compound **45.**

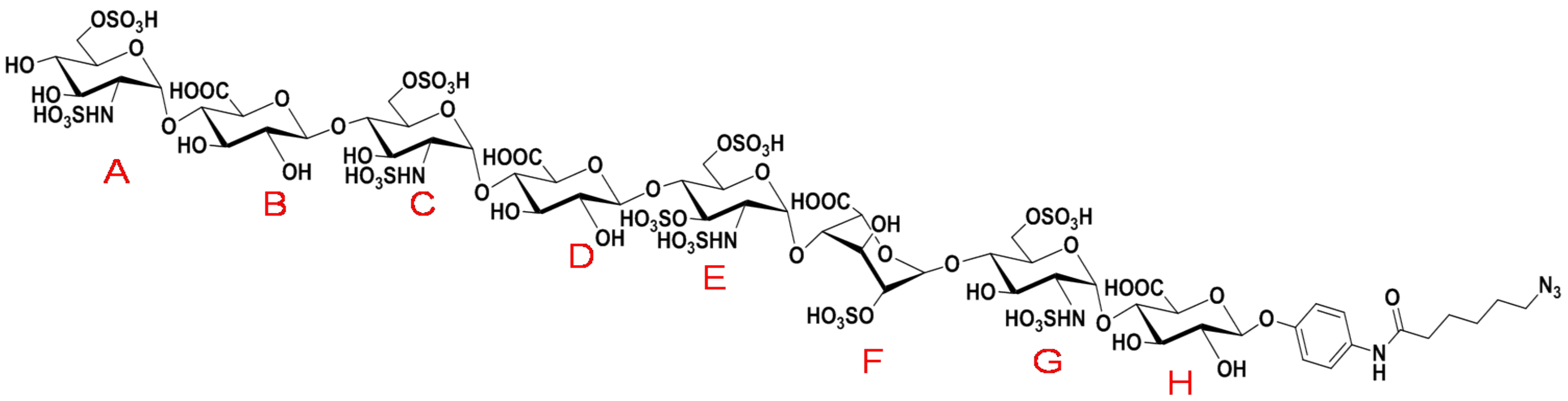

	1	2	3	4	5	6a	6b
A	5.54	3.17	3.59	3.53	3.81	4.07	4.29
B	4.53	3.32	3.75	3.68	3.81	----------	----------
C	5.34	3.20	3.62	3.67	4.00	4.15	4.37
D	4.53	3.32	3.75	3.68	3.81	----------	----------
E	5.48	3.37	4.31	3.89	4.00	4.13	4.39
F	5.16	4.25	4.12	4.02	4.73	----------	----------
G	5.51	3.22	3.57	3.71	3.87	4.15	4.32
H	5.04	3.57	3.84	3.81	3.87	----------	----------

Supplementary Fig. S136. ^{1}H NMR chemical shift assignments (in ppm) of compound **46**. The structure of compound **46** is shown. The protons of *p*NA-N_3 are not listed in the table.

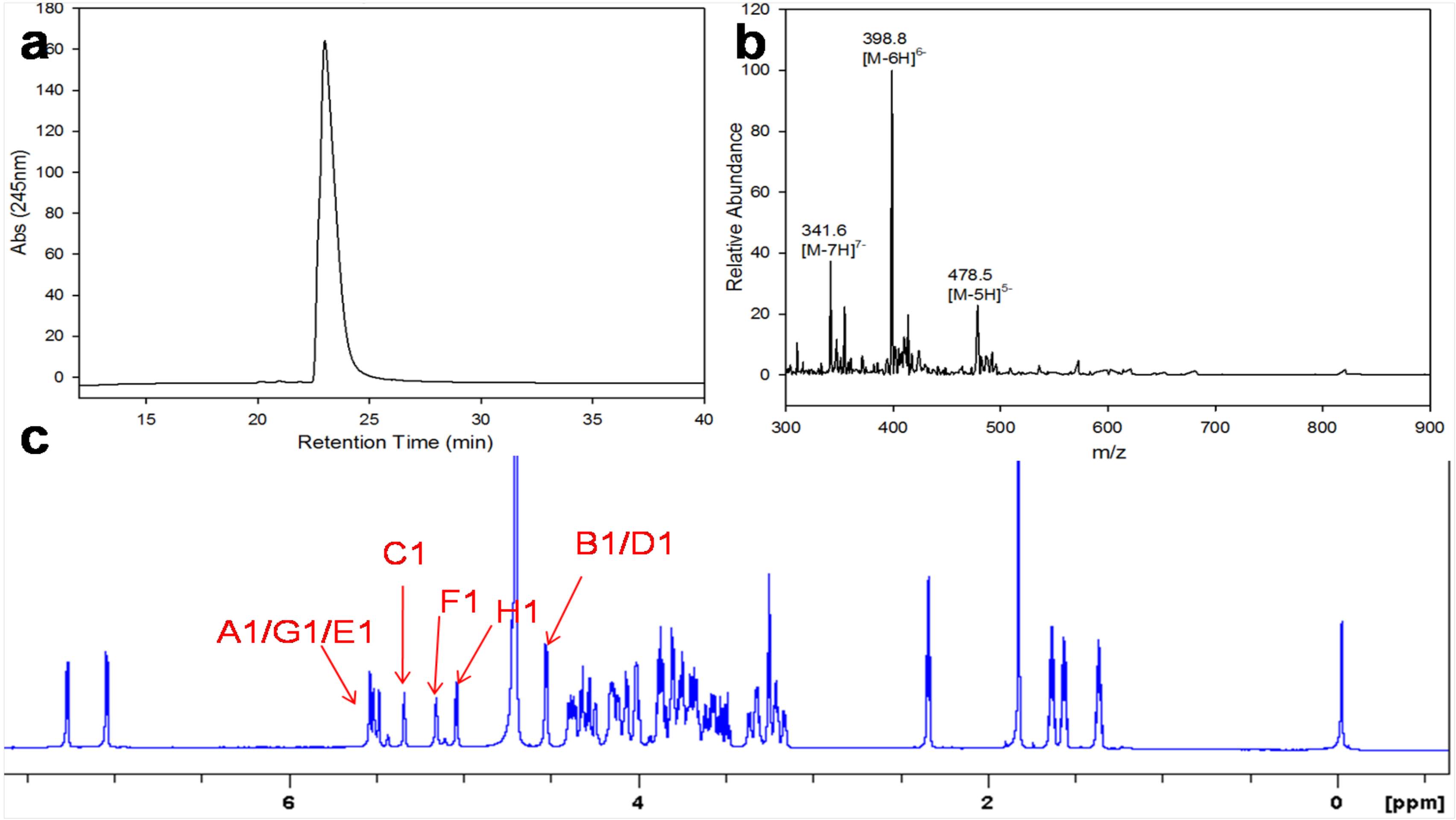

Supplementary Fig. S137. HPLC analysis (a), MS spectrum (b) and ^{1}H NMR spectrum (c) of compound **46**.

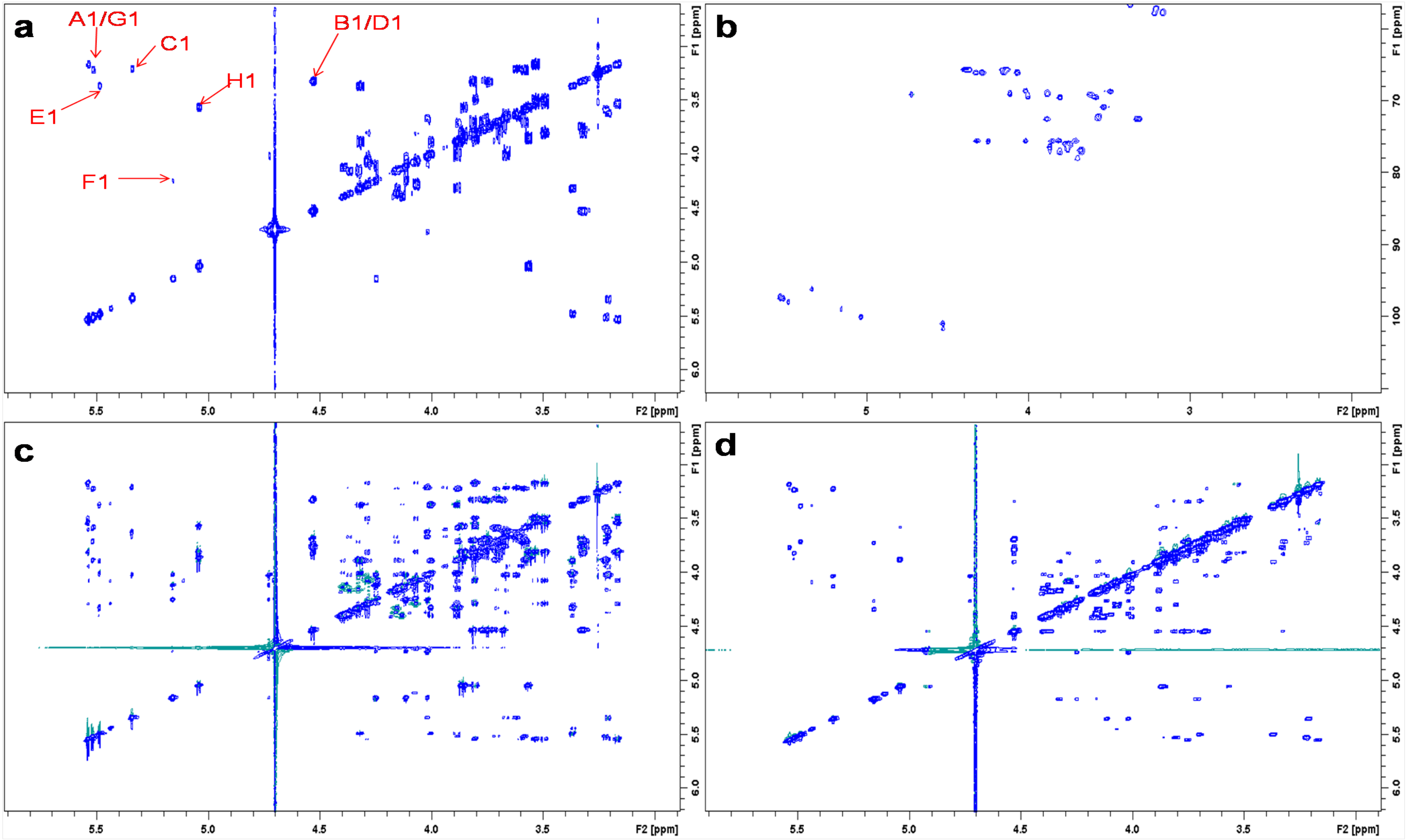

Supplementary Fig. S138. 2D NMR COSY (a), HSQC (b), TOCSY (c) and NOE (d) spectra of compound **46.**

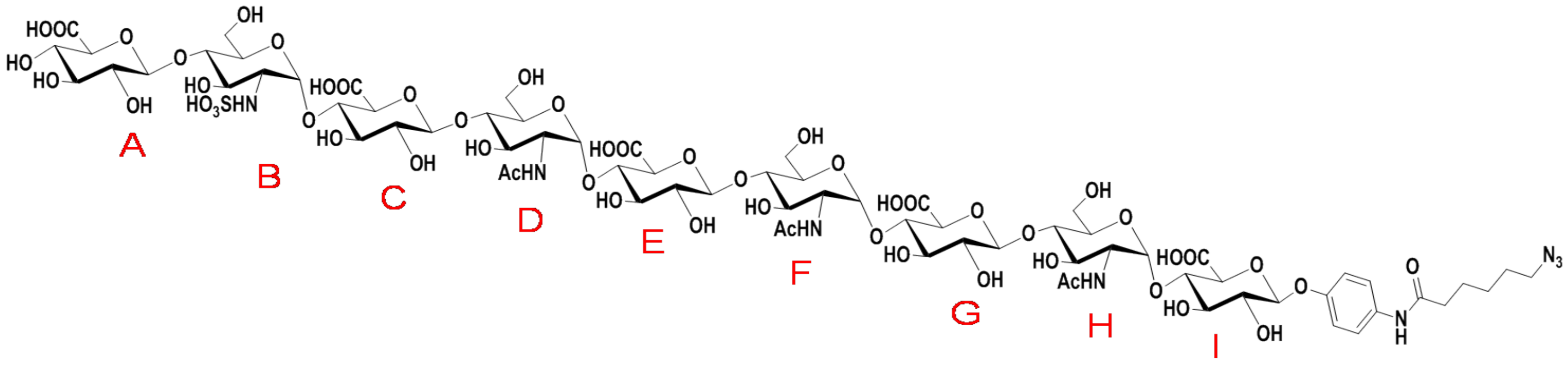

	1	2	3	4	5	6a	6b
A	4.45	3.29	3.44	3.45	3.78	----------	----------
B	5.53	3.17	3.60	3.70	3.78	3.72	3.76
C	4.45	3.31	3.77	3.79	3.80	----------	----------
D	5.29	3.78	3.74	3.74	3.74	3.58	3.74
E	4.42	3.26	3.61	3.67	3.77	----------	----------
F	5.29	3.78	3.74	3.74	3.74	3.58	3.74
G	4.42	3.26	3.61	3.67	3.77	----------	----------
H	5.32	3.79	3.76	3.76	3.76	3.59	3.76
I	5.00	3.50	3.70	3.74	3.88	----------	----------

Supplementary Fig. S139. ^{1}H NMR chemical shift assignments (in ppm) of compound **47**. The structure of compound **47** is shown. The protons of *p*NA-N_3 are not listed in the table.

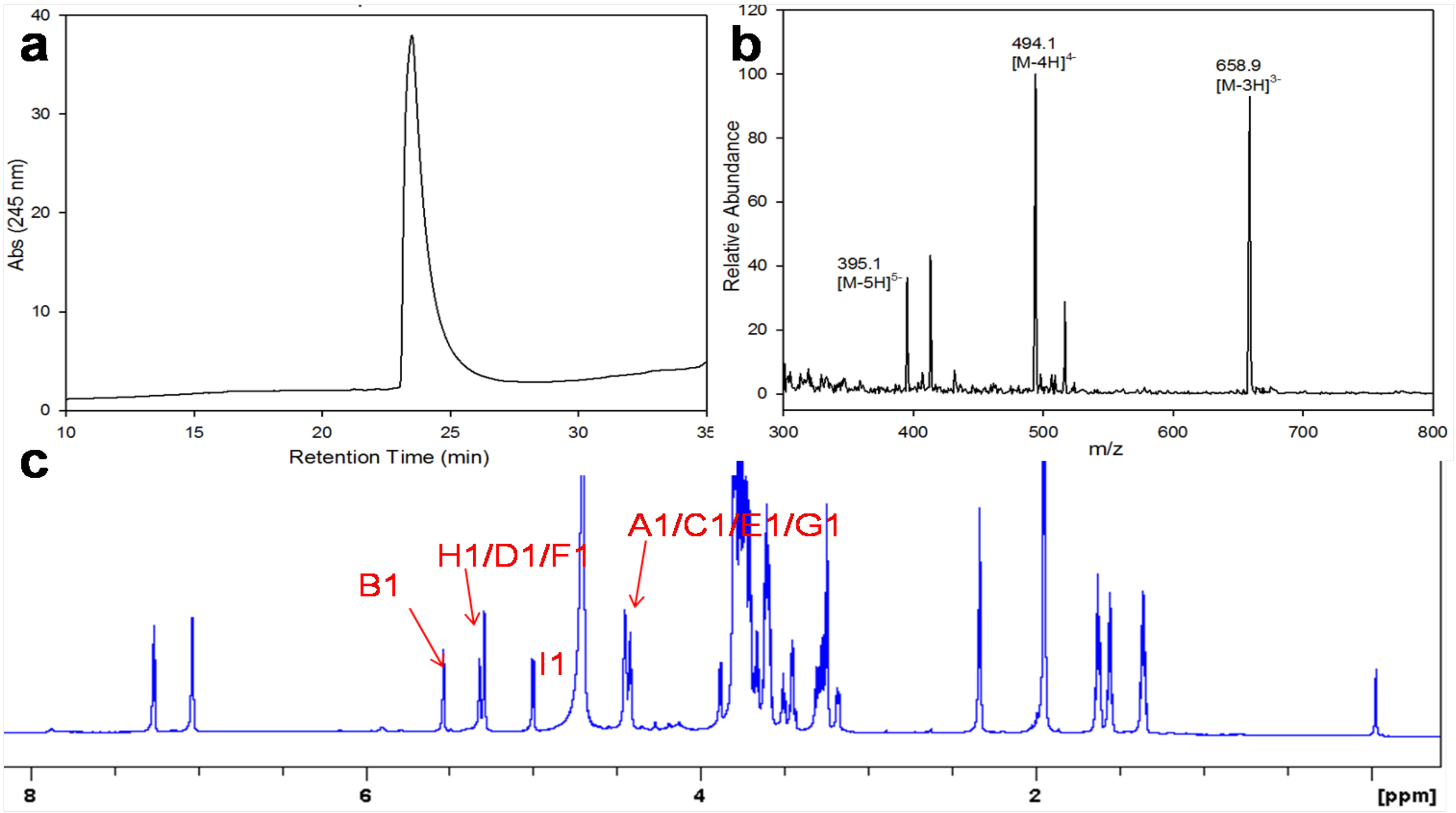

Supplementary Fig. S140. HPLC analysis (a), MS spectrum (b) and ^{1}H NMR spectrum (c) of compound **47**.

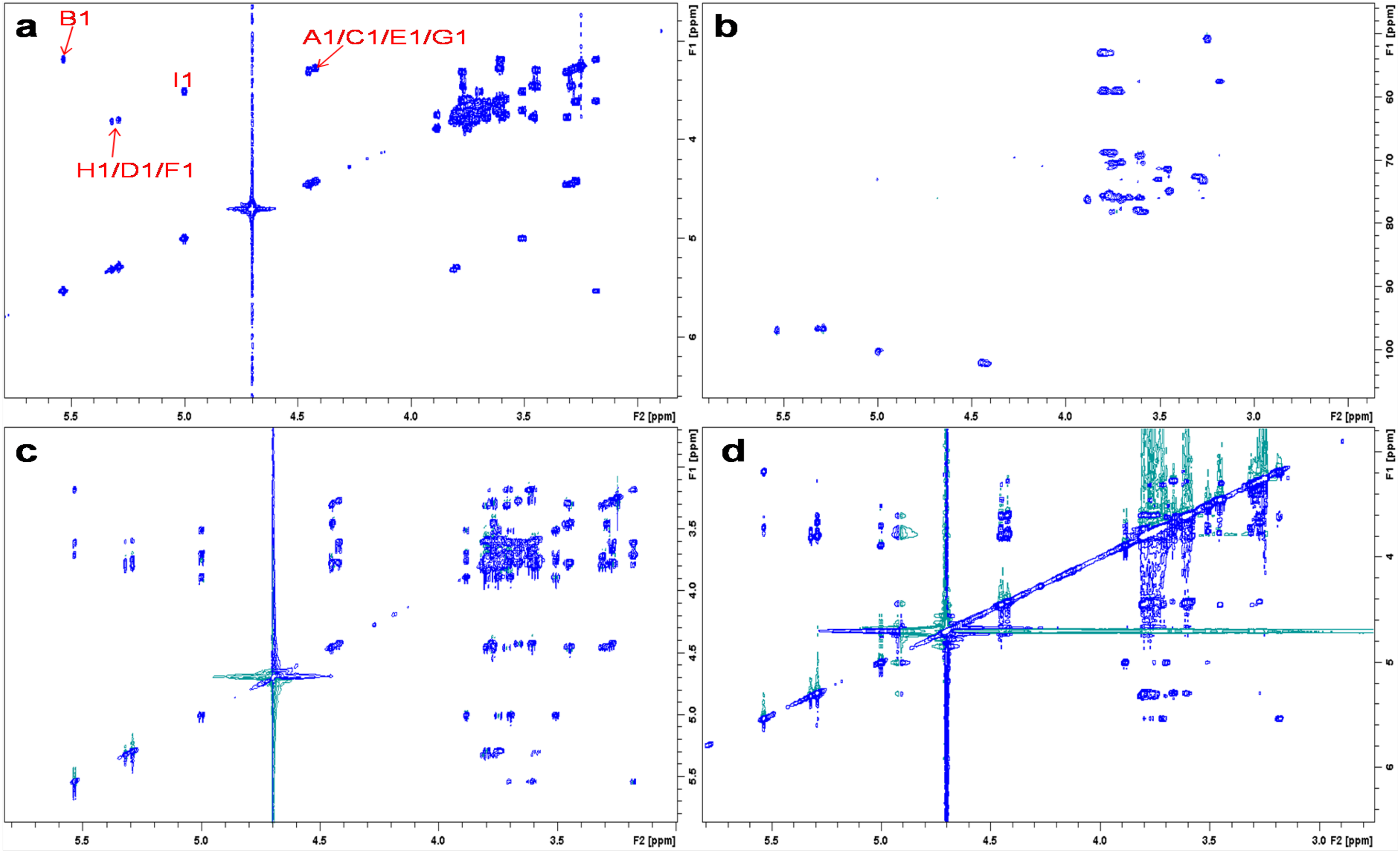

Supplementary Fig. S141. 2D NMR COSY (a), HSQC (b), TOCSY (c) and NOE (d) spectra of compound **47.**

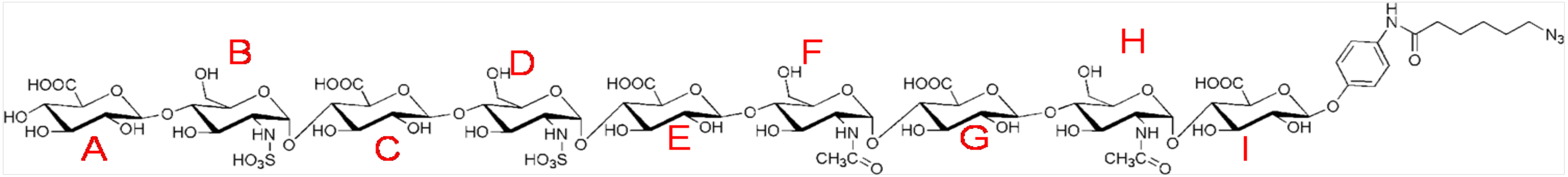

	1	2	3	4	5	6a	6b
A	4.42	3.29	3.40	3.50	3.75	----------	----------
B	5.47	3.12	3.54	3.67	3.74	3.71	3.75
C	4.40	3.26	3.62	3.75	3.75	----------	----------
D	5.47	3.12	3.54	3.67	3.74	3.71	3.75
E	4.40	3.26	3.62	3.75	3.75	----------	----------
F	5.27	3.78	3.72	3.72	3.72	3.56	3.78
G	4.40	3.26	3.62	3.75	3.75	----------	----------
H	5.30	3.79	3.75	3.75	3.75	3.60	3.79
I	4.96	3.46	3.66	3.70	3.86	----------	----------

Supplementary Fig. S142. ^{1}H NMR chemical shift assignments (in ppm) of compound **48**. The structure of compound **48** is shown. The protons of *p*NA-N_3 are not listed in the table.

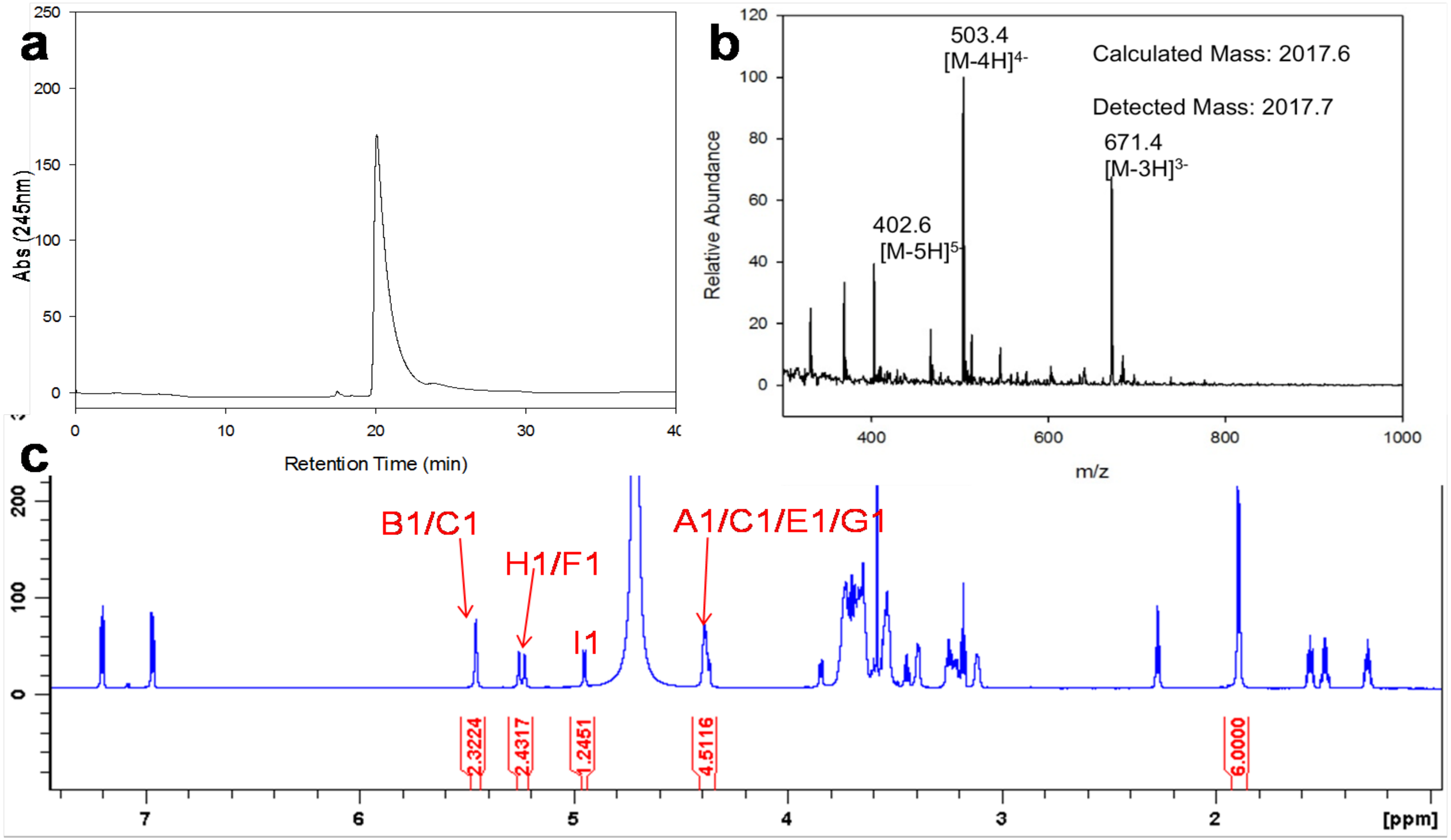

Supplementary Fig. S143. HPLC analysis (a), MS spectrum (b) and ^{1}H NMR spectrum (c) of compound **48**.

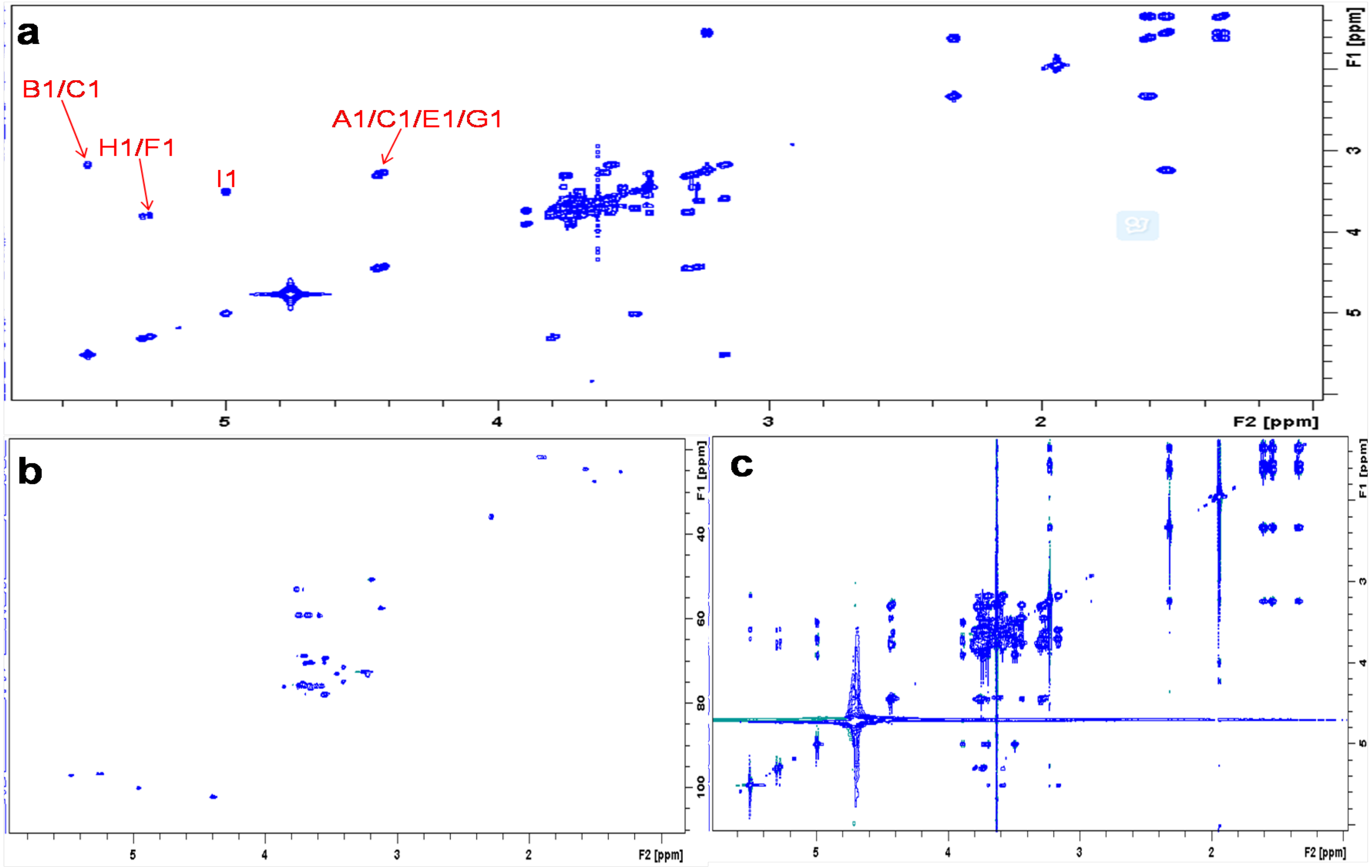

Supplementary Fig. S144. 2D NMR COSY (a), HSQC (b) and TOCSY (c) spectra of compound **48.**

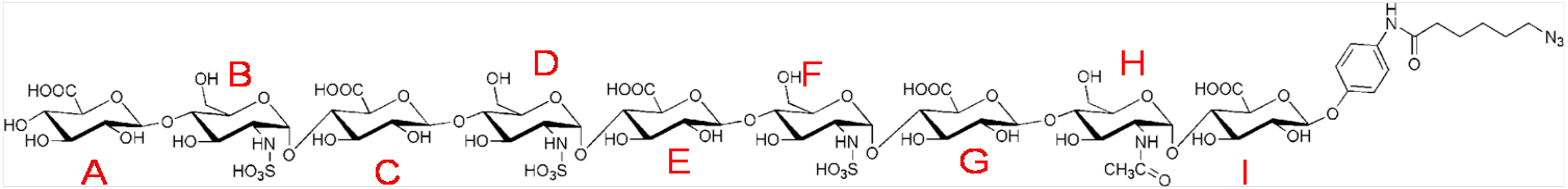

	1	2	3	4	5	6a	6b
A	4.46	3.32	3.45	3.45	3.83	----------	----------
B	5.52	3.21	3.60	3.70	3.72	3.75	3.78
C	4.46	3.32	3.76	3.78	3.83	----------	----------
D	5.52	3.21	3.60	3.70	3.72	3.75	3.78
E	4.46	3.32	3.76	3.78	3.83	----------	----------
F	5.52	3.21	3.60	3.70	3.72	3.75	3.78
G	4.46	3.32	3.76	3.78	3.83	----------	----------
H	5.31	3.81	3.75	3.79	3.79	3.62	3.83
I	5.01	3.50	3.71	3.79	3.90	----------	----------

Supplementary Fig. S145. ^{1}H NMR chemical shift assignments (in ppm) of compound **49**. The structure of compound **49** is shown. The protons of *p*NA-N_3 are not listed in the table.

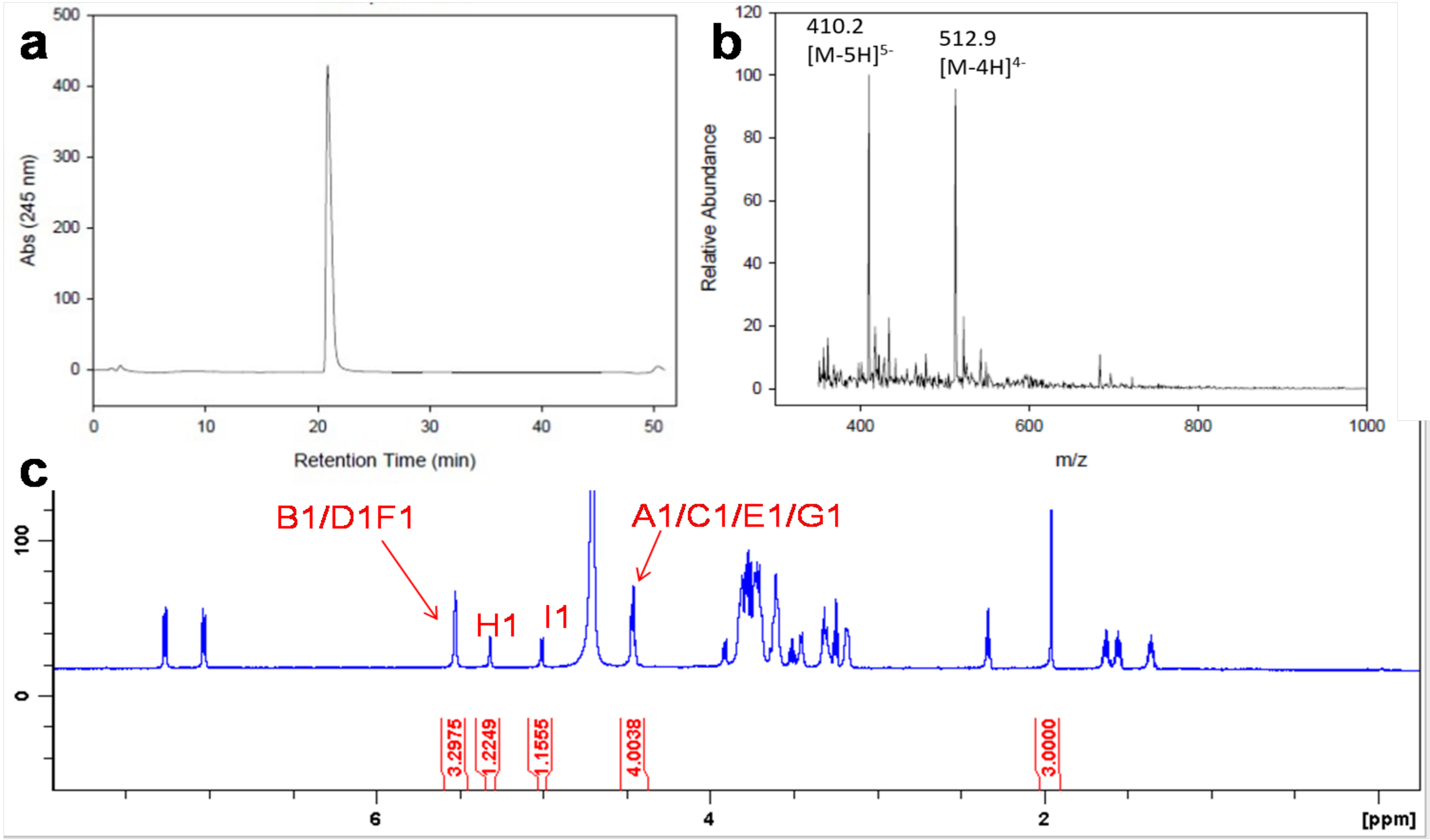

Supplementary Fig. S146. HPLC analysis (a), MS spectrum (b) and ^{1}H NMR spectrum (c) of compound **49**.

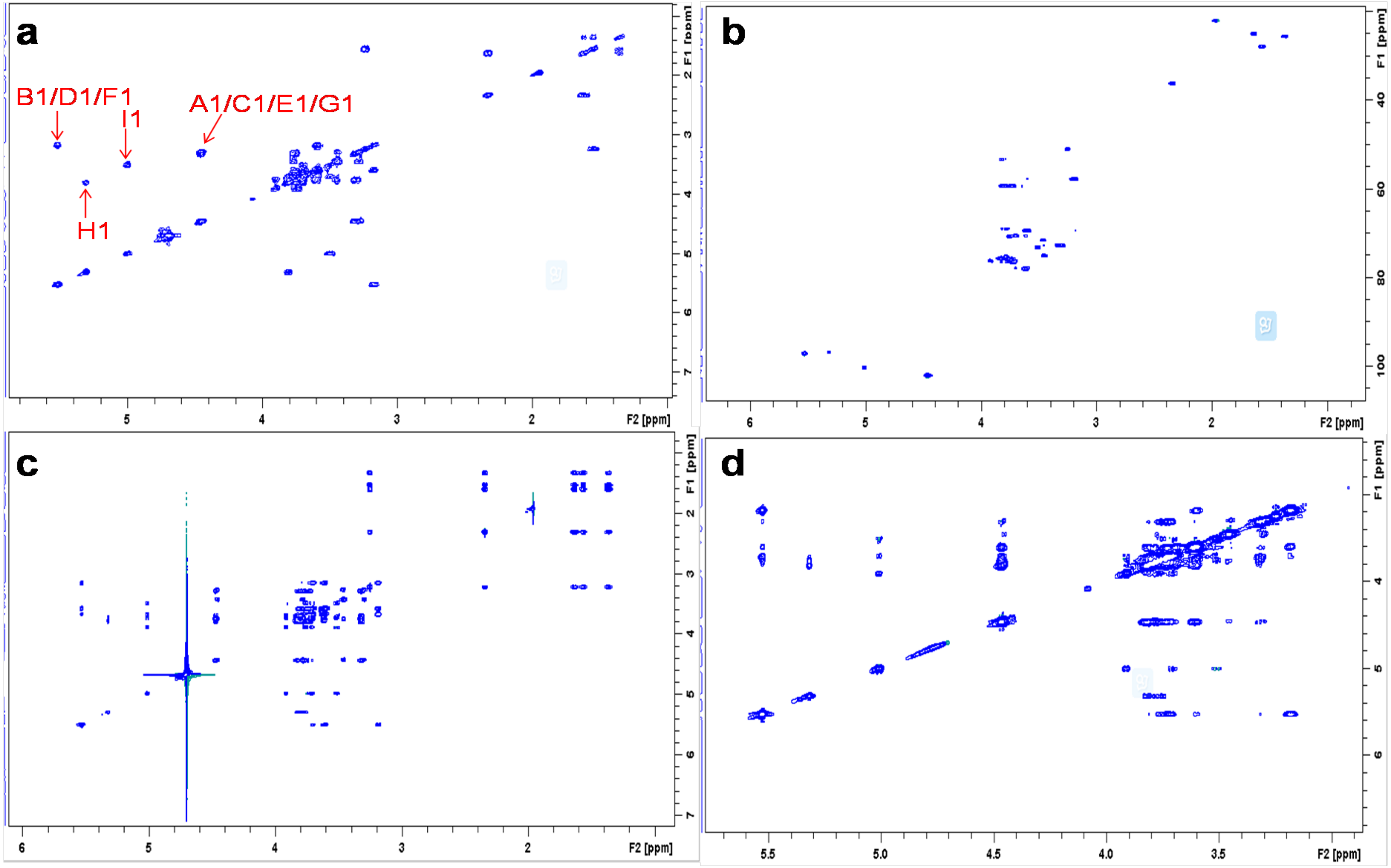

Supplementary Fig. S147. 2D NMR COSY (a), HSQC (b), TOCSY (c) and NOE (d) spectra of compound **49.**

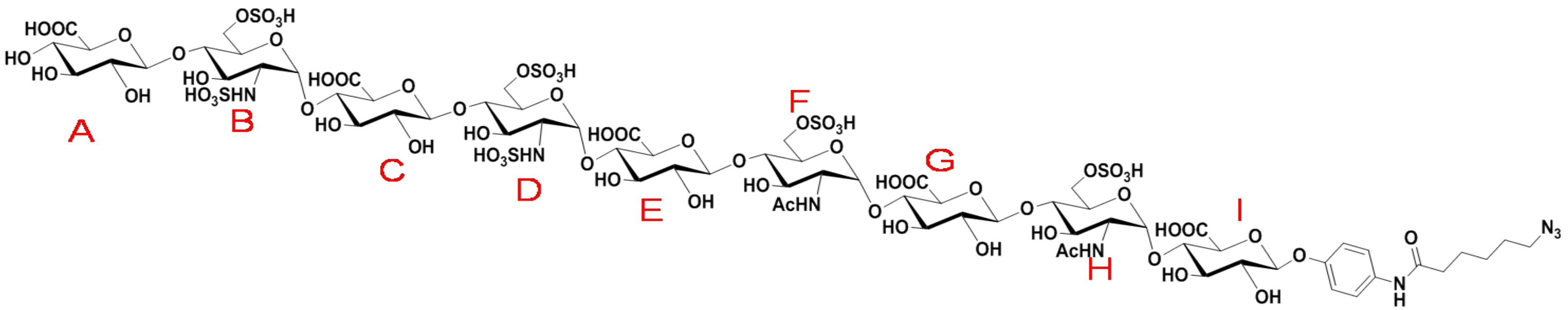

	1	2	3	4	5	6a	6b
A	4.43	3.21	3.39	3.61	3.68	----------	----------
B	5.49	3.15	3.55	3.61	3.88	4.05	4.30
C	4.45	3.23	3.70	3.70	3.70	----------	----------
D	5.49	3.15	3.55	3.61	3.88	4.05	4.30
E	4.45	3.23	3.70	3.70	3.70	----------	----------
F	5.28	3.79	3.71	3.59	3.90	4.05	4.30
G	4.45	3.23	3.70	3.70	3.70	----------	----------
H	5.28	3.79	3.71	3.59	3.90	4.05	4.30
I	4.95	3.46	3.65	3.70	3.81	----------	----------

Supplementary Fig. S148. ^{1}H NMR chemical shift assignments (in ppm) of compound **50**. The structure of compound **50** is shown. The protons of *p*NA-N_3 are not listed in the table.

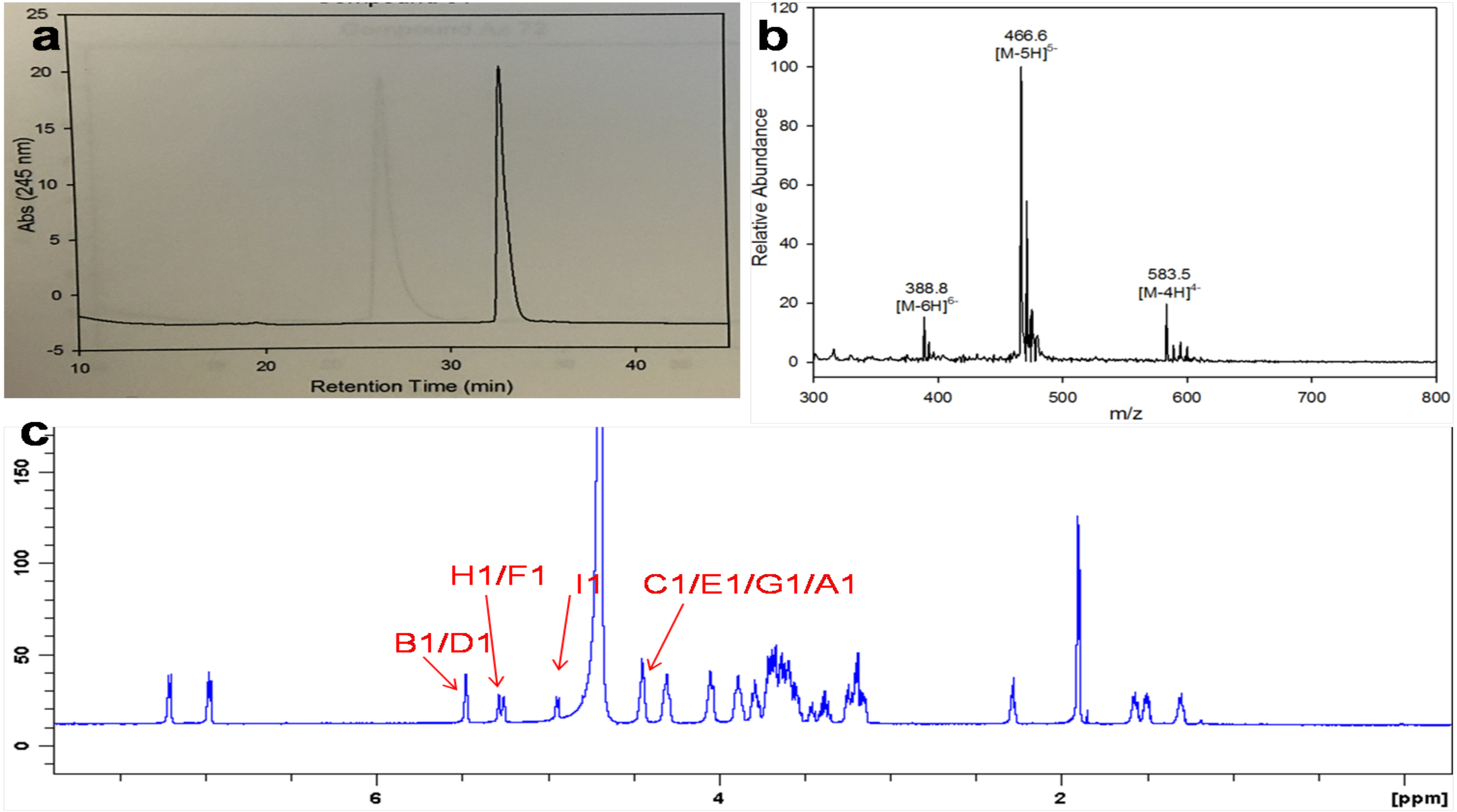

Supplementary Fig. S149. HPLC analysis (a), MS spectrum (b) and ^{1}H NMR spectrum (c) of compound **50**.

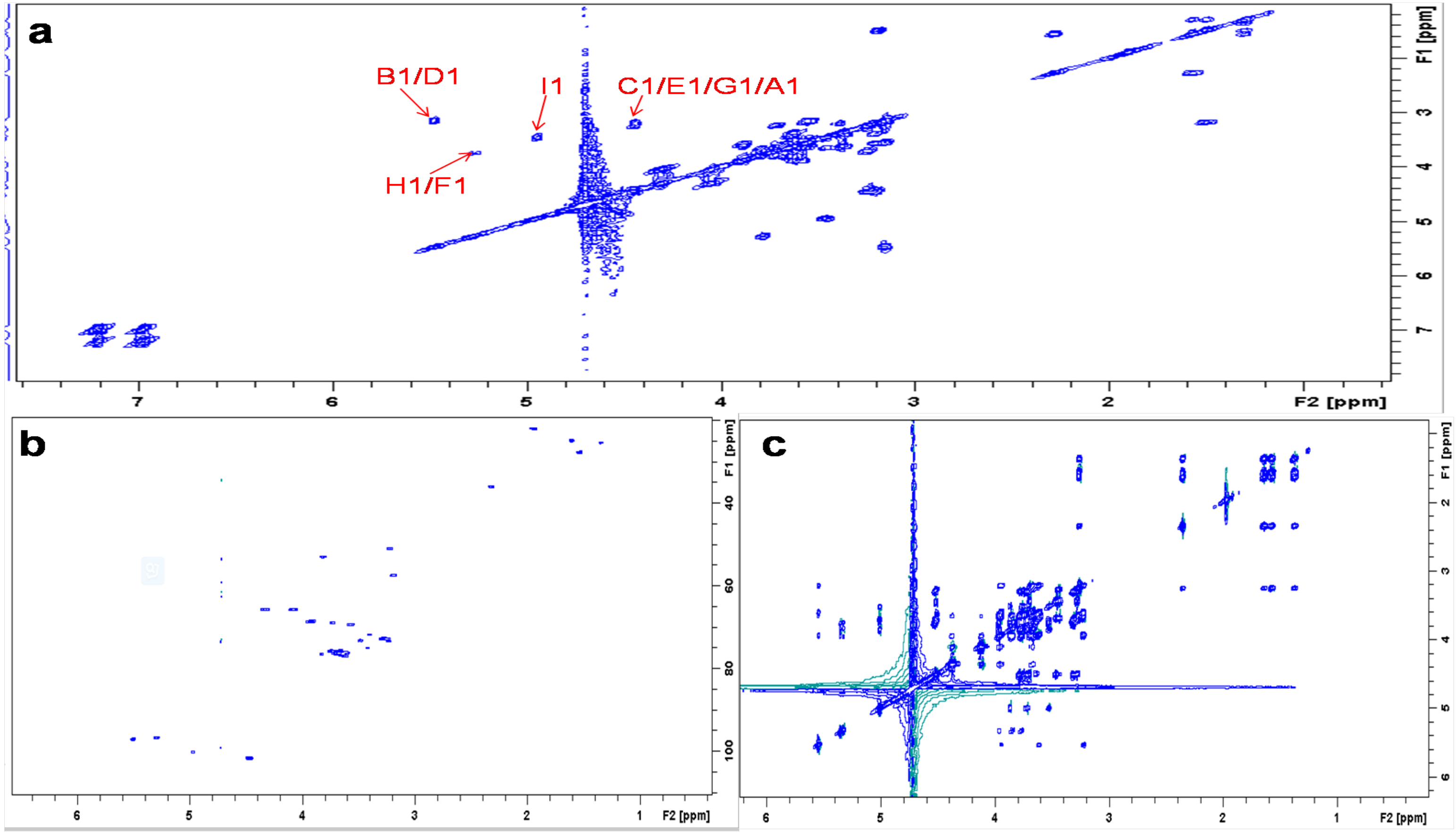

Supplementary Fig. S150. 2D NMR COSY (a), HSQC (b) and TOCSY (c) spectra of compound **50.**

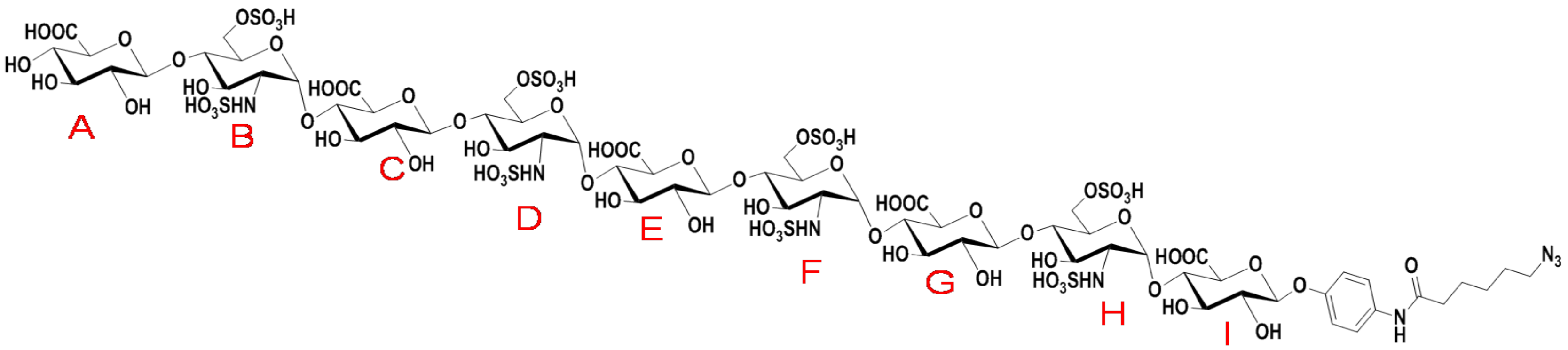

	1	2	3	4	5	6a	6b
A	4.52	3.26	3.46	3.46	3.70	----------	----------
B	5.55	3.23	3.61	3.81	3.96	4.10	4.39
C	4.52	3.31	3.78	3.71	3.76	----------	----------
D	5.55	3.23	3.61	3.81	3.96	4.10	4.39
E	4.52	3.31	3.78	3.71	3.76	----------	----------
F	5.55	3.23	3.61	3.81	3.96	4.10	4.39
G	4.52	3.31	3.78	3.71	3.76	----------	----------
H	5.59	3.26	3.62	3.72	3.94	4.13	4.38
I	5.05	3.56	3.86	3.79	3.87	----------	----------

Supplementary Fig. S151. ^{1}H NMR chemical shift assignments (in ppm) of compound **51**. The structure of compound **51** is shown. The protons of *p*NA-N_3 are not listed in the table.

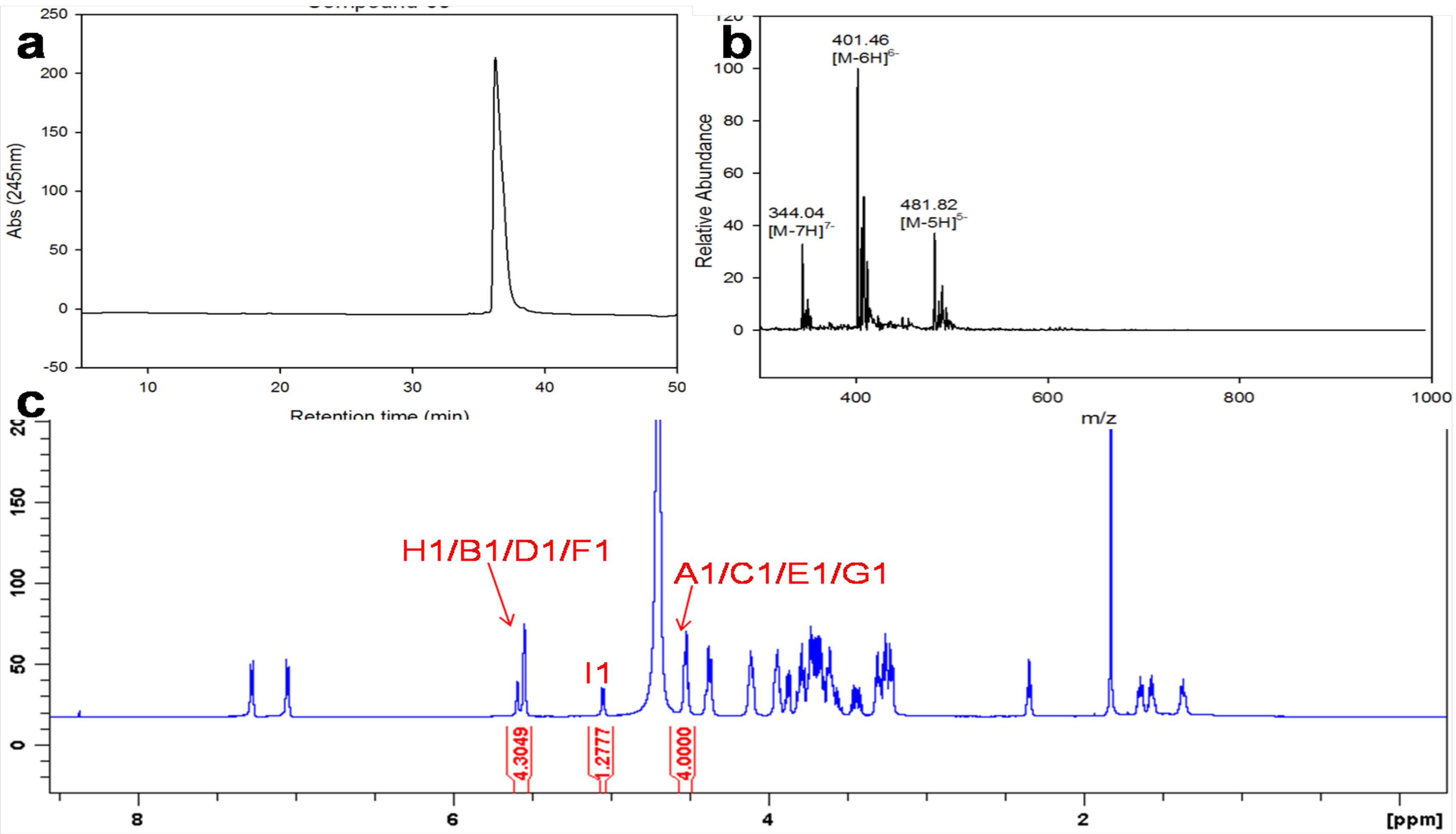

Supplementary Fig. S152. HPLC analysis (a), MS spectrum (b) and ^{1}H NMR spectrum (c) of compound **51**.

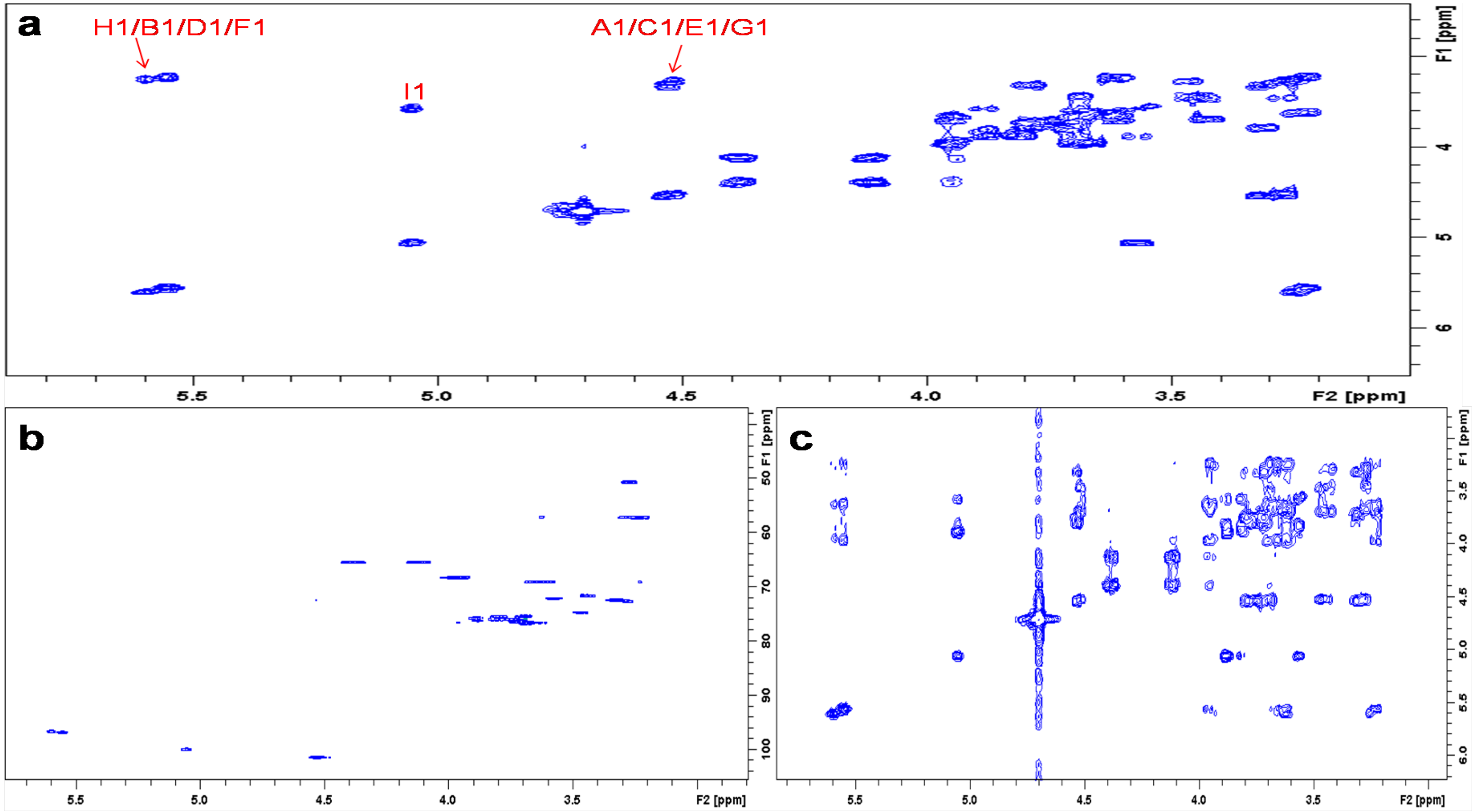

Supplementary Fig. S153. 2D NMR COSY (a), HSQC (b) and TOCSY (c) spectra of compound **51.**

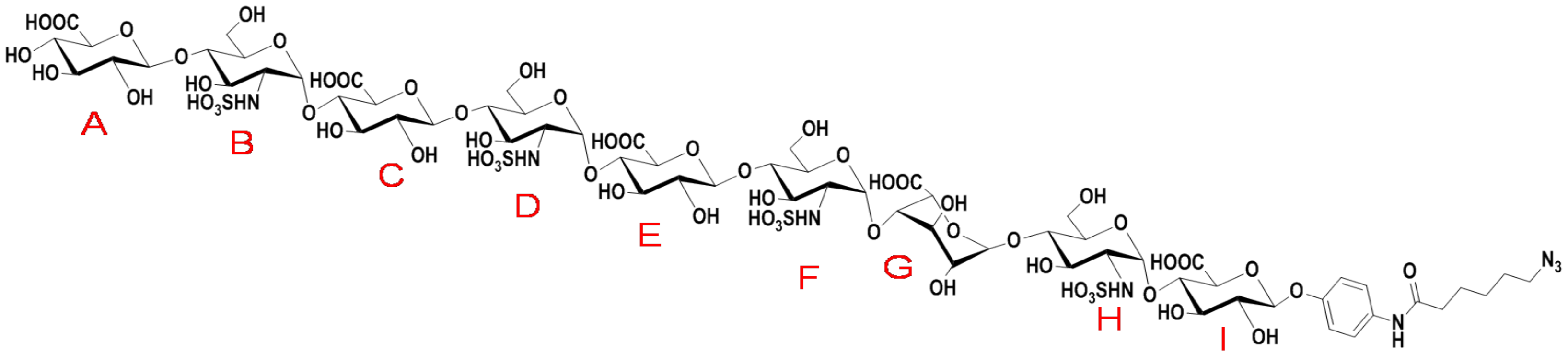

	1	2	3	4	5	6a	6b
A	4.43	3.30	3.44	3.67	3.71	----------	----------
B	5.53	3.19	3.57	3.60	3.75	3.75	3.80
C	4.45	3.31	3.76	3.72	3.76	----------	----------
D	5.53	3.19	3.57	3.60	3.75	3.75	3.80
E	4.45	3.31	3.76	3.72	3.76	----------	----------
F	5.28	3.16	3.61	3.79	3.77	3.75	3.84
G	4.87	3.65	4.02	3.97	4.69	----------	----------
H	5.53	3.19	3.57	3.60	3.75	3.75	3.80
I	5.04	3.55	3.86	3.80	3.82	----------	----------

Supplementary Fig. S154. ^{1}H NMR chemical shift assignments (in ppm) of compound **52**. The structure of compound **52** is shown. The protons of *p*NA-N_3 are not listed in the table.

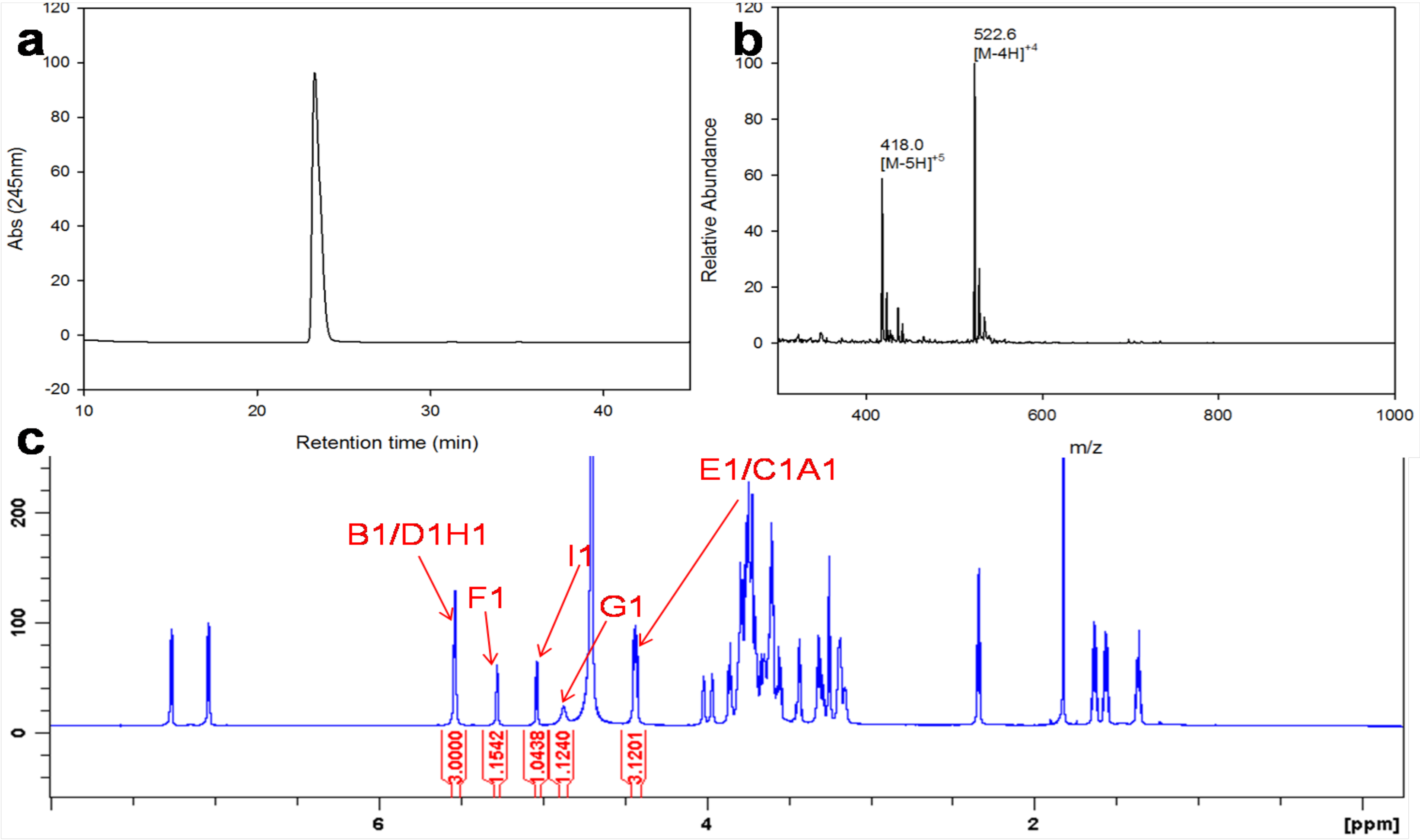

Supplementary Fig. S155. HPLC analysis (a), MS spectrum (b) and ^{1}H NMR spectrum (c) of compound **52**.

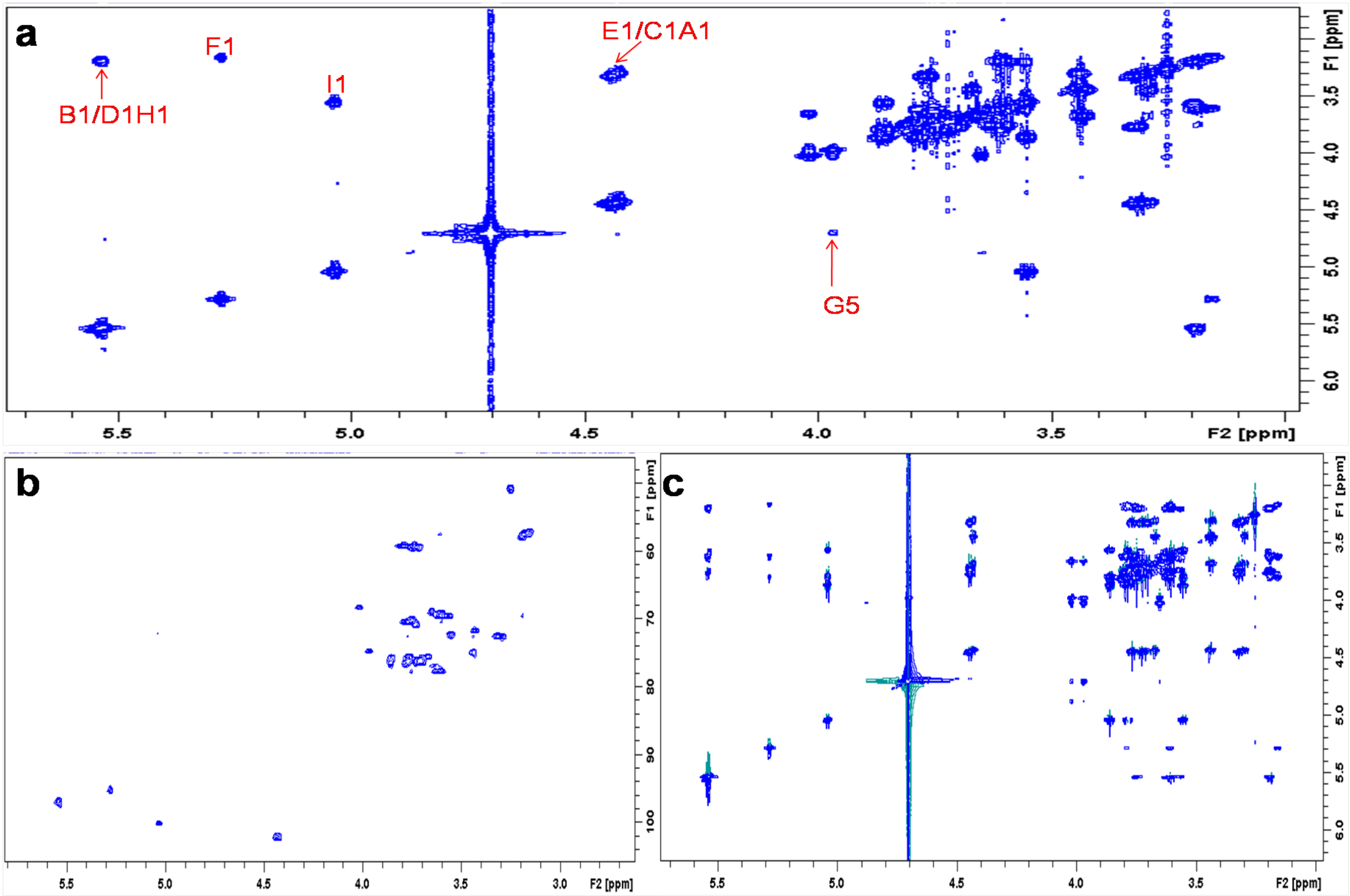

Supplementary Fig. S156. 2D NMR COSY (a), HSQC (b) and TOCSY (c) spectra of compound **52.**

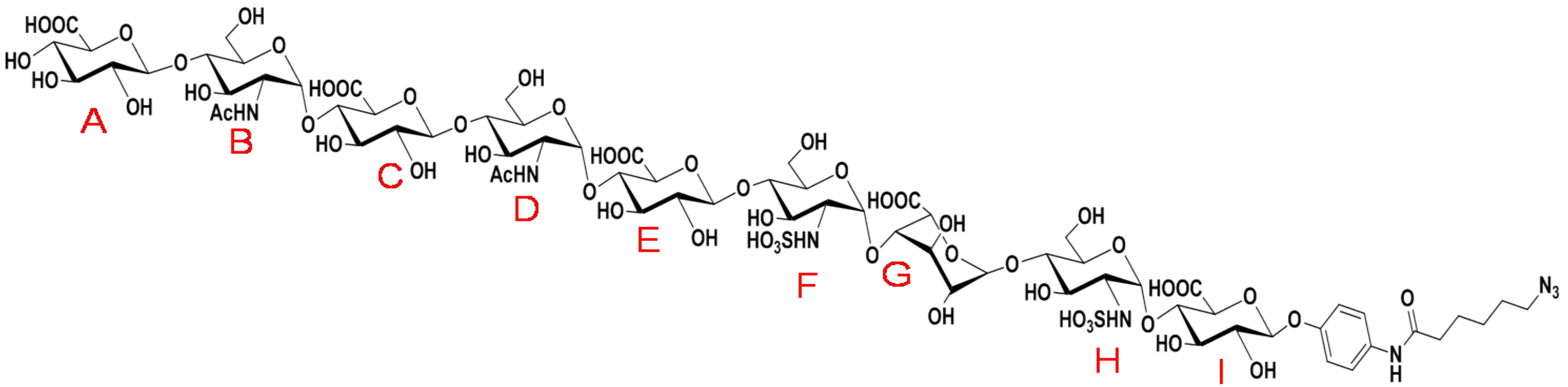

	1	2	3	4	5	6a	6b
A	4.54	3.38	3.56	3.78	3.84	----------	----------
B	5.42	3.92	3.87	3.84	3.84	3.72	3.84
C	4.54	3.39	3.72	3.72	3.83	----------	----------
D	5.42	3.92	3.87	3.84	3.84	3.72	3.84
E	4.54	3.39	3.72	3.72	3.83	----------	----------
F	5.41	3.27	3.72	3.76	3.86	3.75	3.86
G	4.98	3.76	4.13	4.08	4.79	----------	----------
H	5.65	3.31	3.69	3.73	3.79	3.82	3.89
I	5.15	3.67	3.97	3.90	3.98	----------	----------

Supplementary Fig. S157. ^{1}H NMR chemical shift assignments (in ppm) of compound **53**. The structure of compound **53** is shown. The protons of *p*NA-N_3 are not listed in the table.

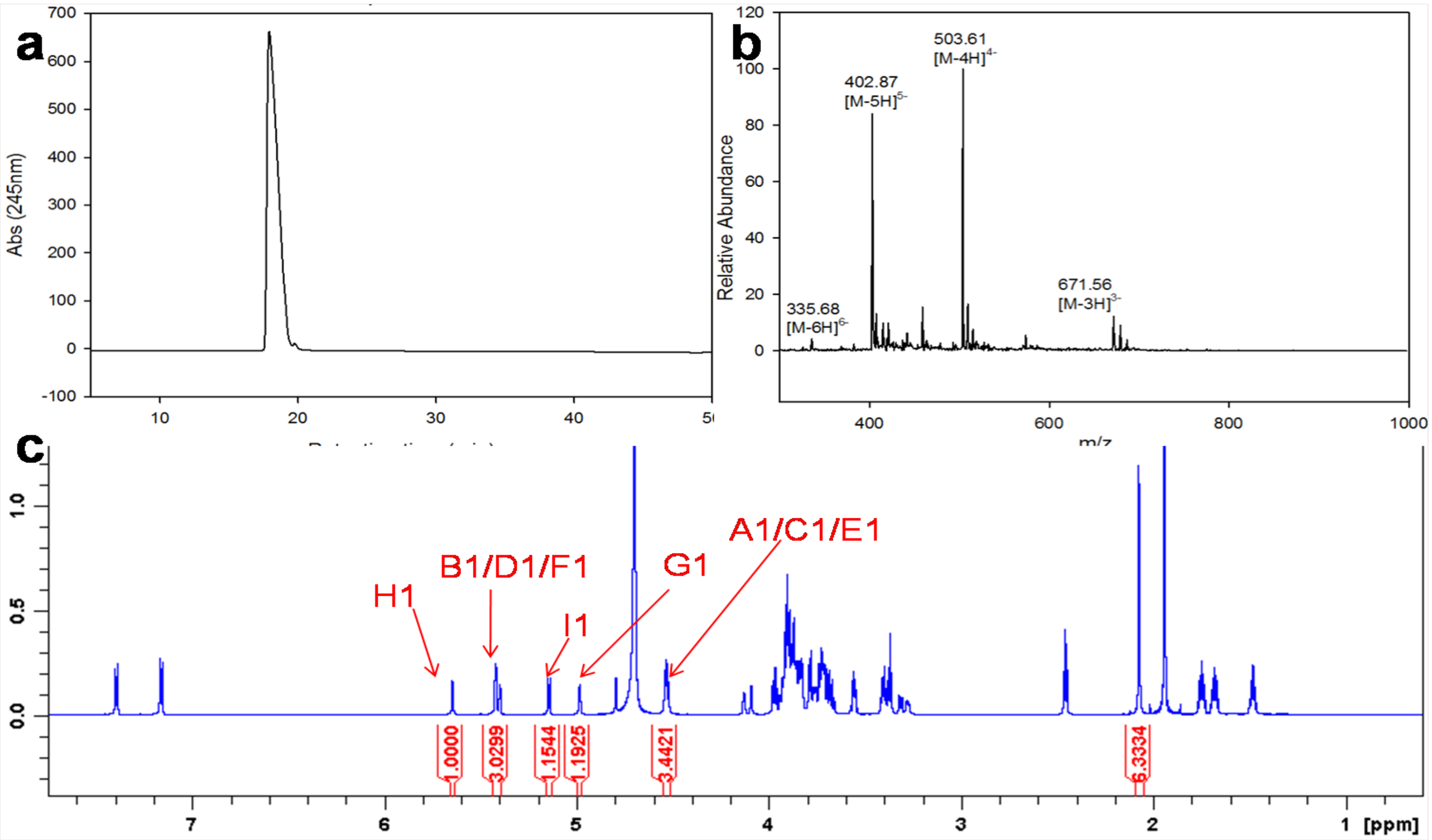

Supplementary Fig. S158. HPLC analysis (a), MS spectrum (b) and ^{1}H NMR spectrum (c) of compound **53**.

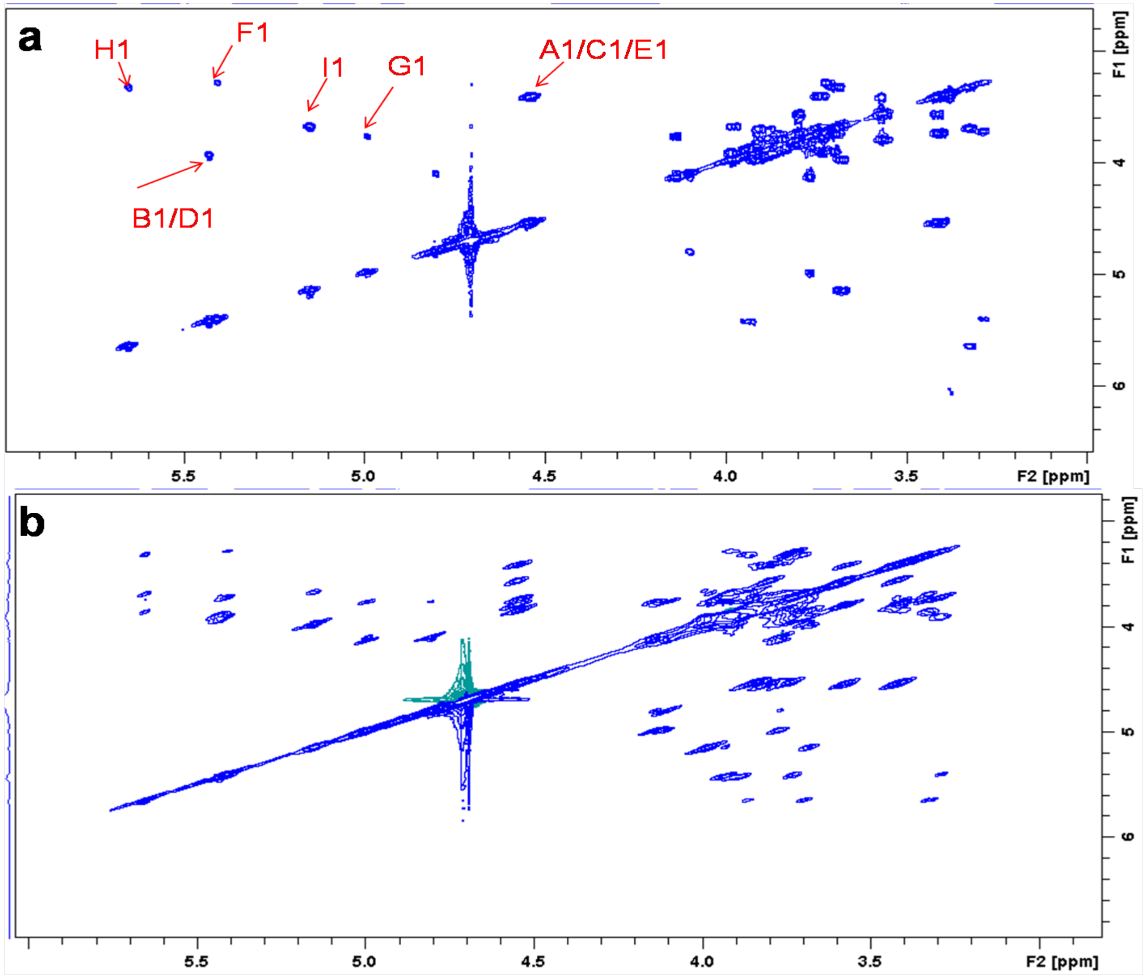

Supplementary Fig. S159. 2D NMR COSY (a) and TOCSY (b) spectra of compound **53.**

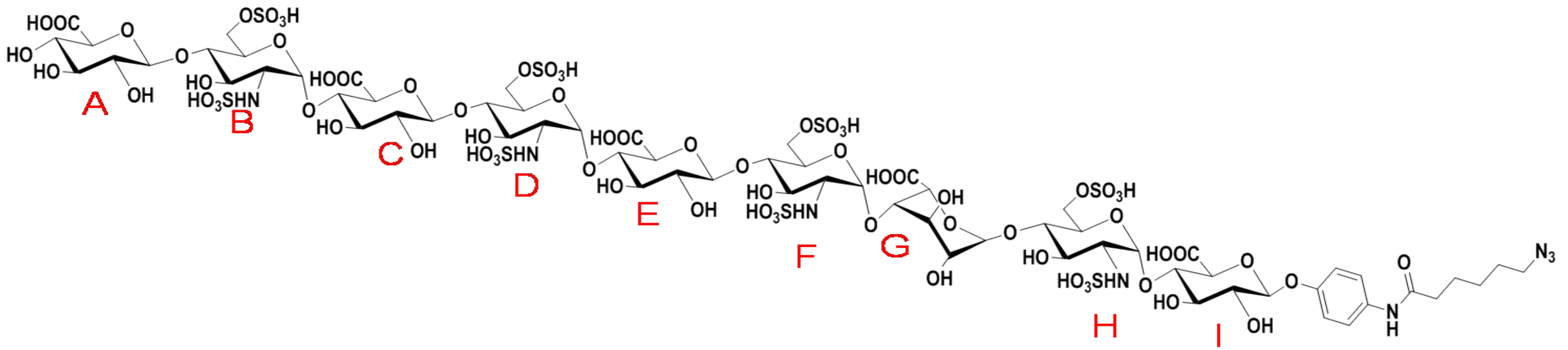

	1	2	3	4	5	6a	6b
A	4.52	3.26	3.46	3.44	3.69	----------	----------
B	5.56	3.23	3.63	3.71	3.96	4.12	4.37
C	4.54	3.31	3.79	3.72	3.75	----------	----------
D	5.56	3.23	3.63	3.71	3.96	4.12	4.37
E	4.54	3.31	3.79	3.72	3.75	----------	----------
F	5.26	3.19	3.63	3.70	3.96	4.10	4.25
G	4.96	3.73	4.05	3.98	4.75	----------	----------
H	5.56	3.23	3.63	3.71	3.96	4.12	4.37
I	5.05	3.57	3.86	3.79	3.88	----------	----------

Supplementary Fig. S160. ^{1}H NMR chemical shift assignments (in ppm) of compound **54**. The structure of compound **54** is shown. The protons of *p*NA-N_3 are not listed in the table.

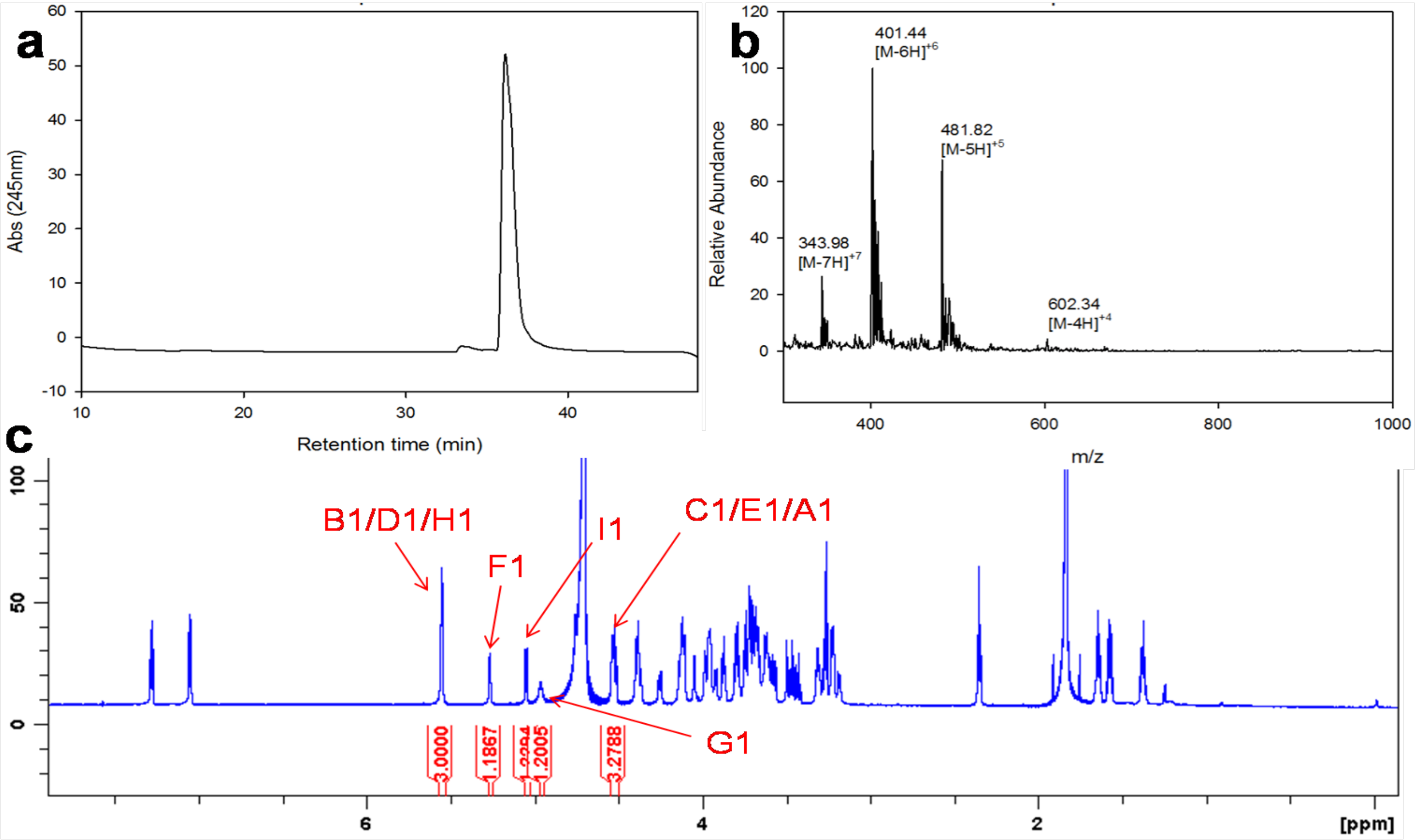

Supplementary Fig. S161. HPLC analysis (a), MS spectrum (b) and ^{1}H NMR spectrum (c) of compound **54**.

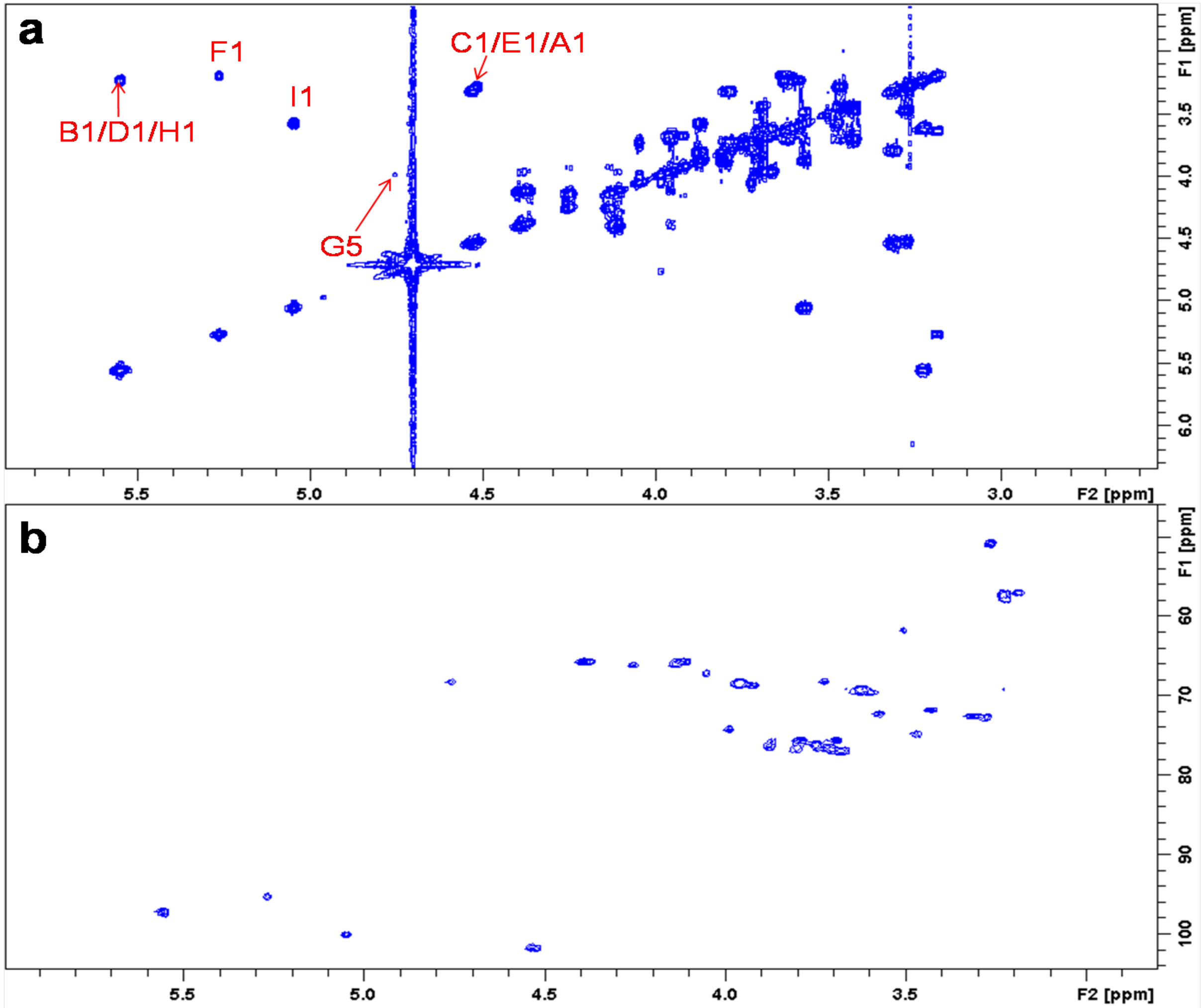

Supplementary Fig. S162. 2D NMR COSY (a) and HSQC (b) spectra of compound **54.**

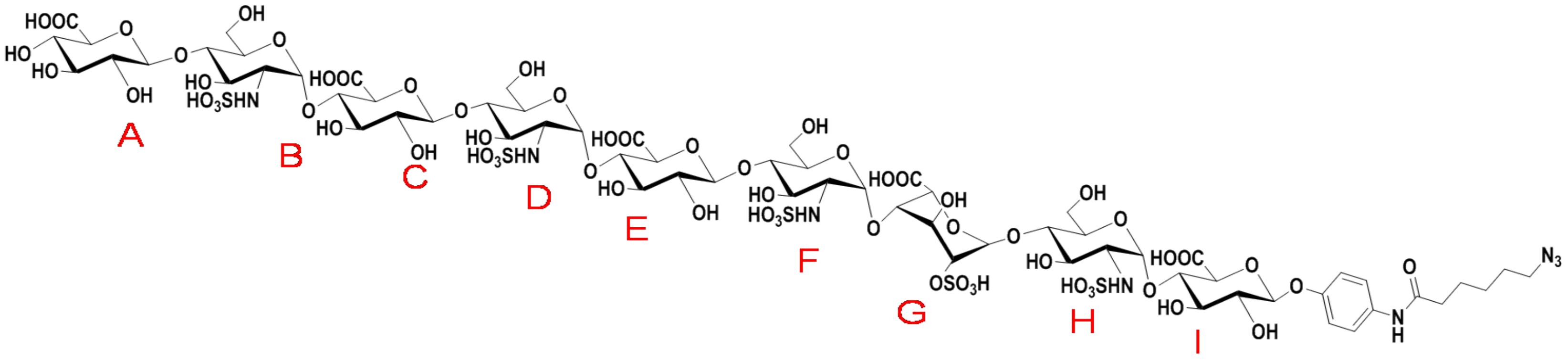

	1	2	3	4	5	6a	6b
A	4.37	3.25	3.39	3.39	3.62	----------	----------
B	5.48	3.14	3.56	3.60	3.60	3.60	3.70
C	4.38	3.25	3.66	3.72	3.72	----------	----------
D	5.48	3.14	3.56	3.60	3.60	3.60	3.70
E	4.38	3.25	3.66	3.72	3.72	----------	----------
F	5.17	3.10	3.58	3.68	3.68	3.65	3.77
G	5.12	4.20	4.11	3.90	4.72	----------	----------
H	5.45	3.14	3.54	3.58	3.58	3.60	3.68
I	4.99	3.50	3.77	3.72	3.80	----------	----------

Supplementary Fig. S163. ^{1}H NMR chemical shift assignments (in ppm) of compound **55**. The structure of compound **55** is shown. The protons of *p*NA-N_3 are not listed in the table.

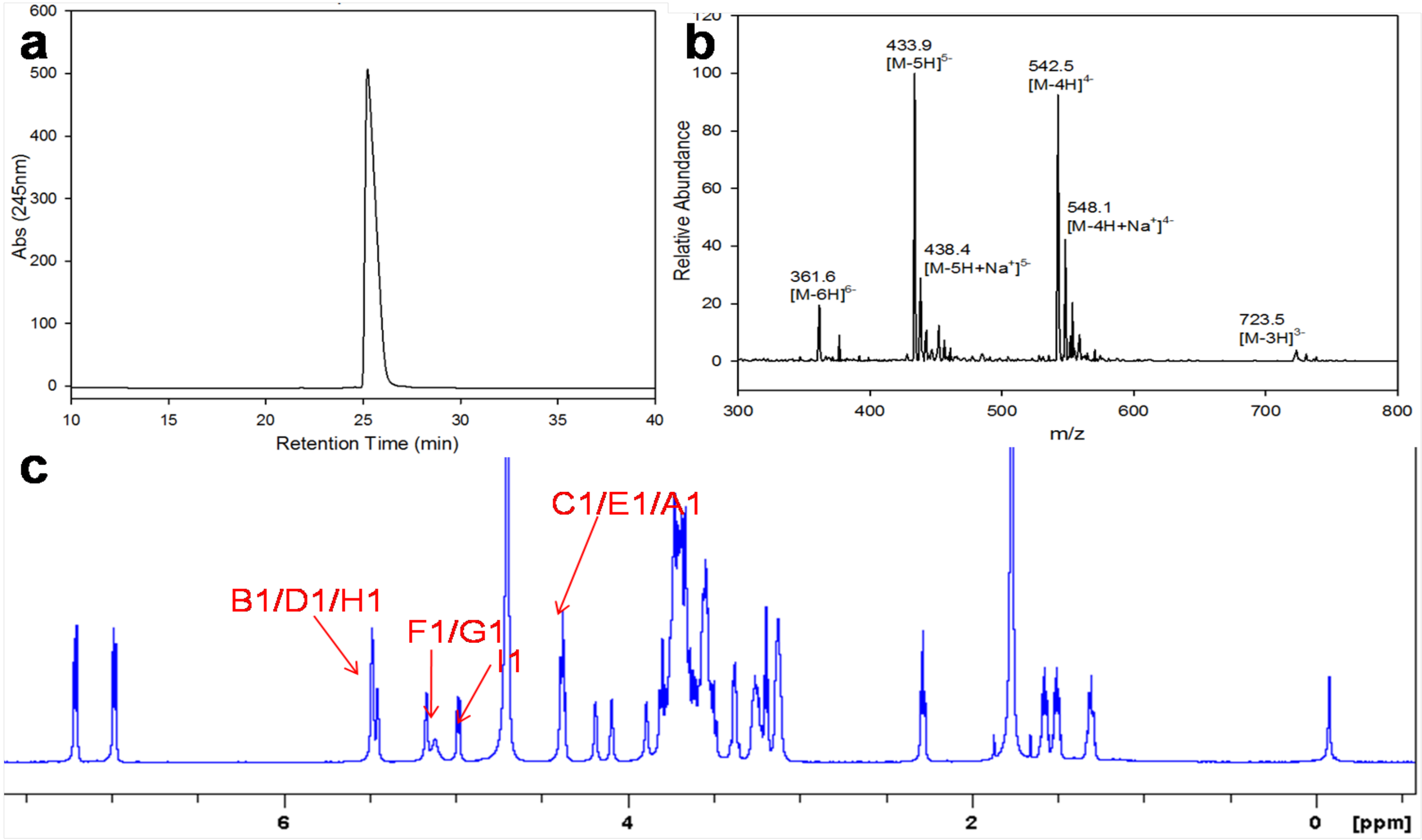

Supplementary Fig. S164. HPLC analysis (a), MS spectrum (b) and ^{1}H NMR spectrum (c) of compound **55**.

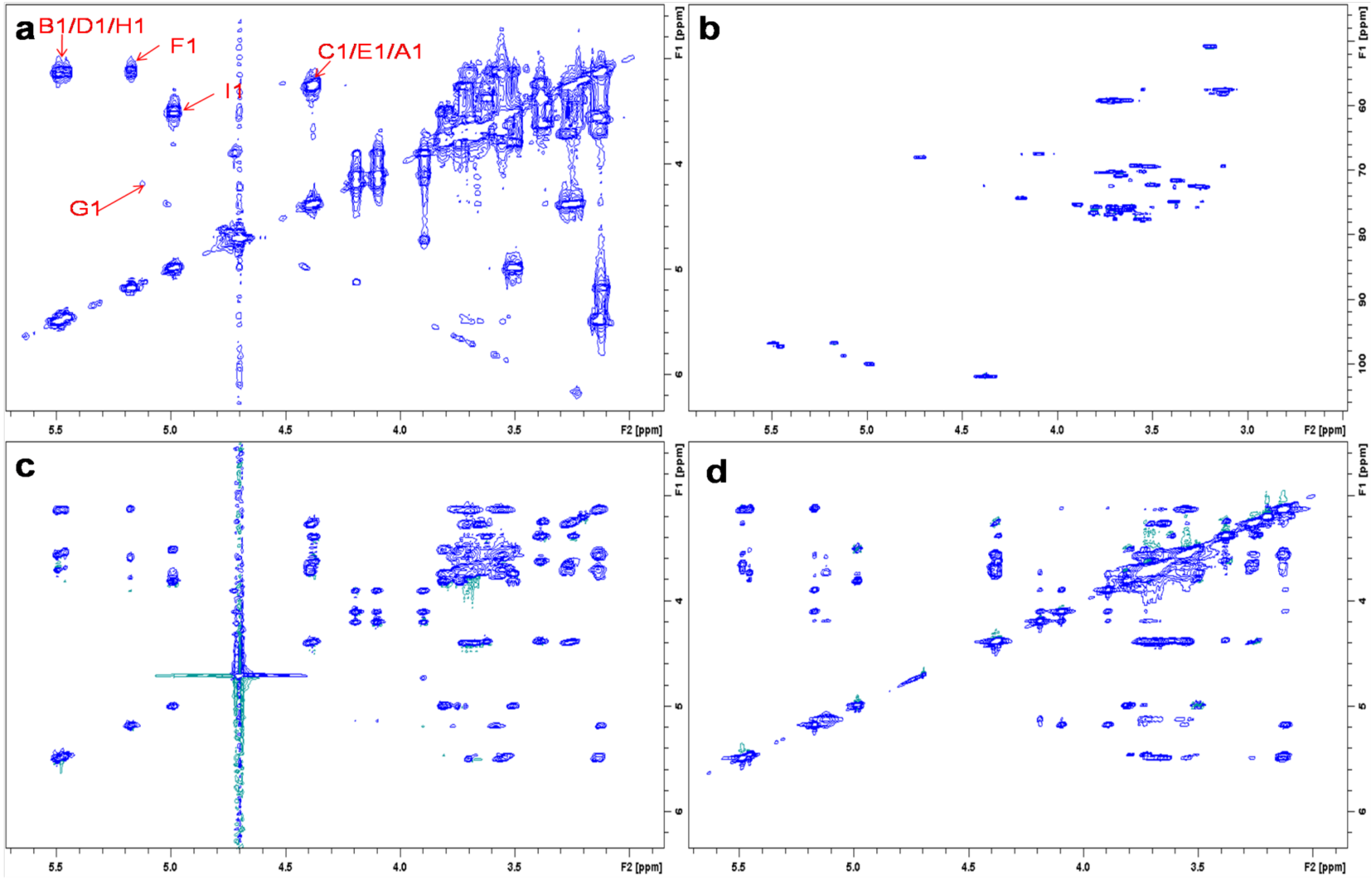

Supplementary Fig. S165. 2D NMR COSY (a), HSQC (b), TOCSY (c) and NOE (d) spectra of compound **55.**

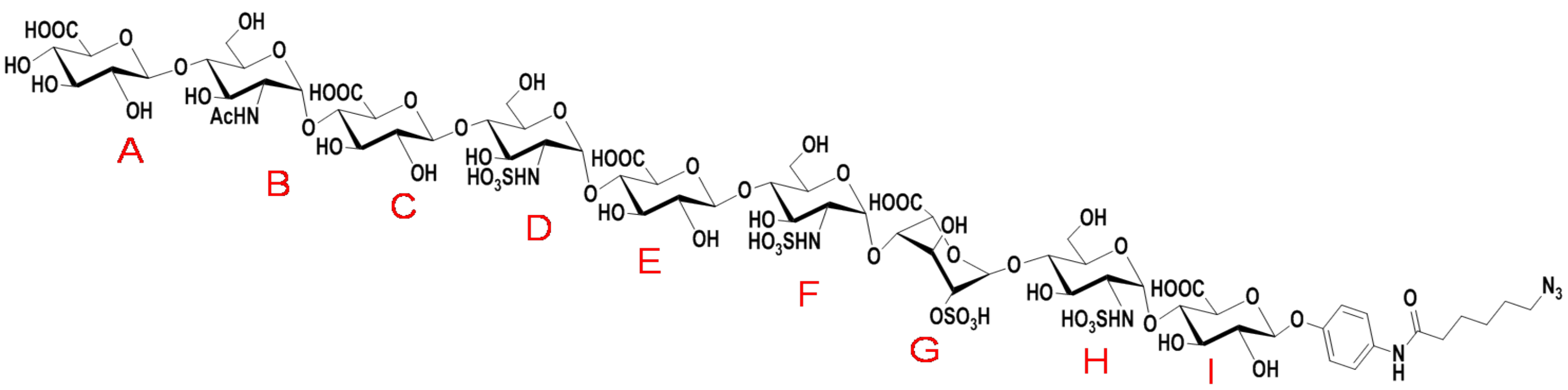

	1	2	3	4	5	6a	6b
A	4.42	3.28	3.44	3.44	3.67	----------	----------
B	5.31	3.81	3.61	3.75	3.75	3.75	3.80
C	4.41	3.31	3.61	3.71	3.71	----------	----------
D	5.53	3.18	3.59	3.64	3.75	3.64	3.75
E	4.44	3.31	3.76	3.72	3.72	----------	----------
F	5.23	3.17	3.63	3.71	3.75	3.75	3.82
G	5.17	4.25	4.15	3.95	4.77	----------	----------
H	5.50	3.18	3.58	3.64	3.64	3.64	3.72
I	5.03	3.55	3.84	3.80	3.86	----------	----------

Supplementary Fig. S166. ^{1}H NMR chemical shift assignments (in ppm) of compound **56**. The structure of compound **56** is shown. The protons of *p*NA-N_3 are not listed in the table.

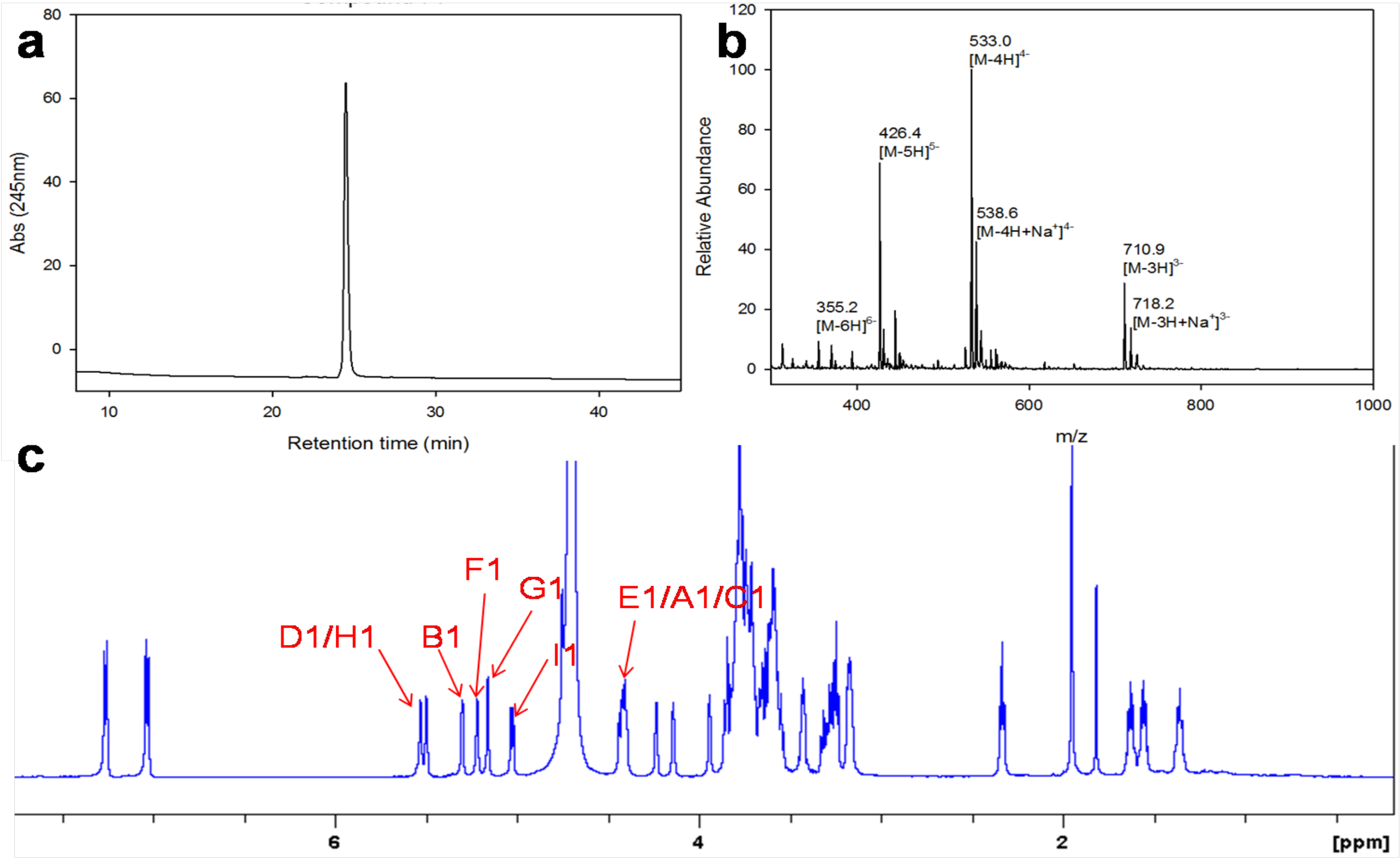

Supplementary Fig. S167. HPLC analysis (a), MS spectrum (b) and ^{1}H NMR spectrum (c) of compound **56**.

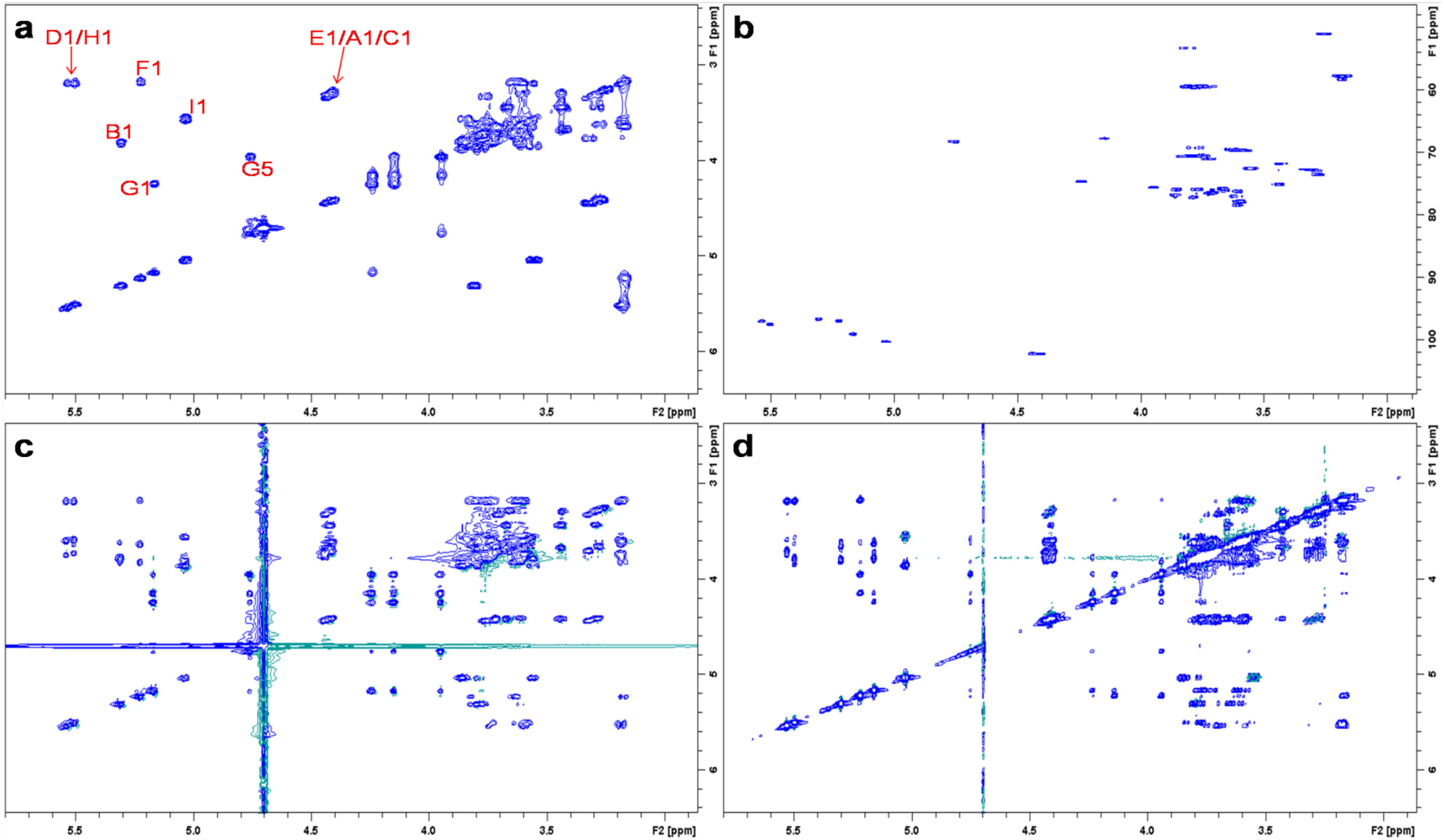

Supplementary Fig. S168. 2D NMR COSY (a), HSQC (b), TOCSY (c) and NOE (d) spectra of compound **56.**

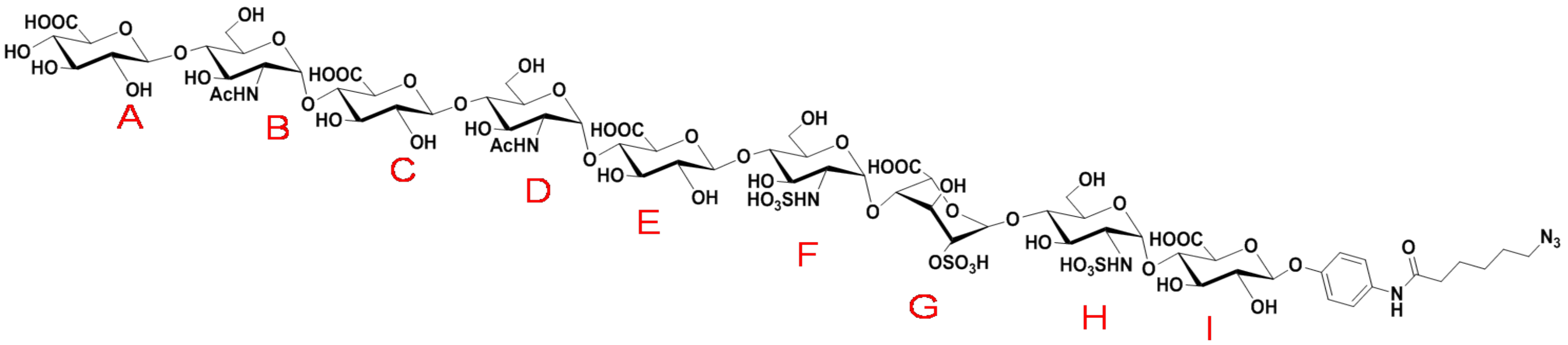

	1	2	3	4	5	6a	6b
A	4.42	3.28	3.44	3.61	3.68	----------	----------
B	5.31	3.81	3.60	3.75	3.75	3.75	3.77
C	4.41	3.29	3.61	3.67	3.72	----------	----------
D	5.31	3.81	3.60	3.75	3.75	3.75	3.77
E	4.41	3.29	3.61	3.67	3.72	----------	----------
F	5.24	3.17	3.63	3.75	3.75	3.63	3.82
G	5.23	4.25	4.16	3.97	4.78	----------	----------
H	5.51	3.18	3.59	3.66	3.74	3.66	3.74
I	5.04	3.55	3.81	3.78	3.87	----------	----------

Supplementary Fig. S169. ^{1}H NMR chemical shift assignments (in ppm) of compound **57**. The structure of compound **57** is shown. The protons of *p*NA-N_3 are not listed in the table.

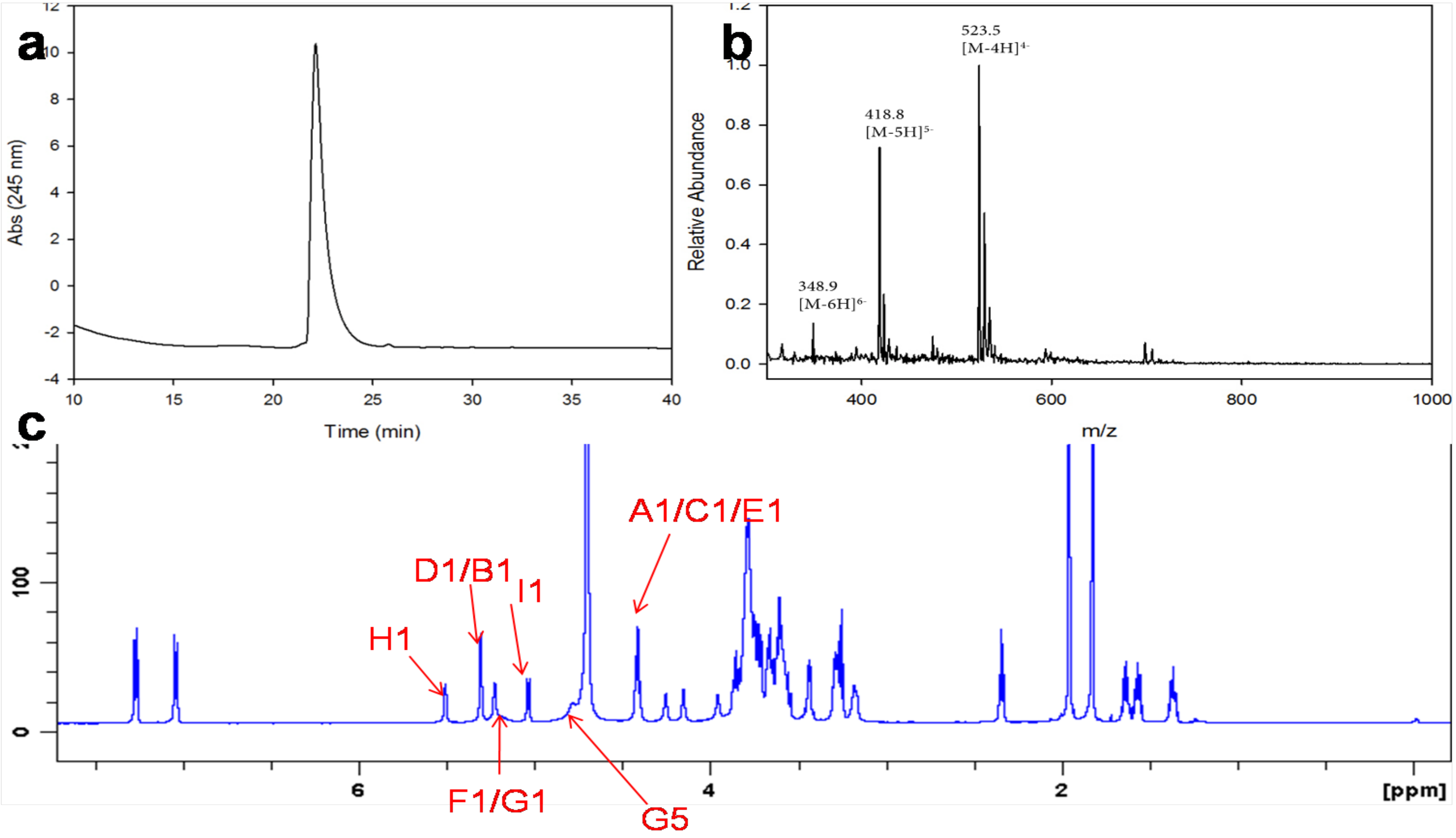

Supplementary Fig. S170. HPLC analysis (a), MS spectrum (b) and ^{1}H NMR spectrum (c) of compound **57**.

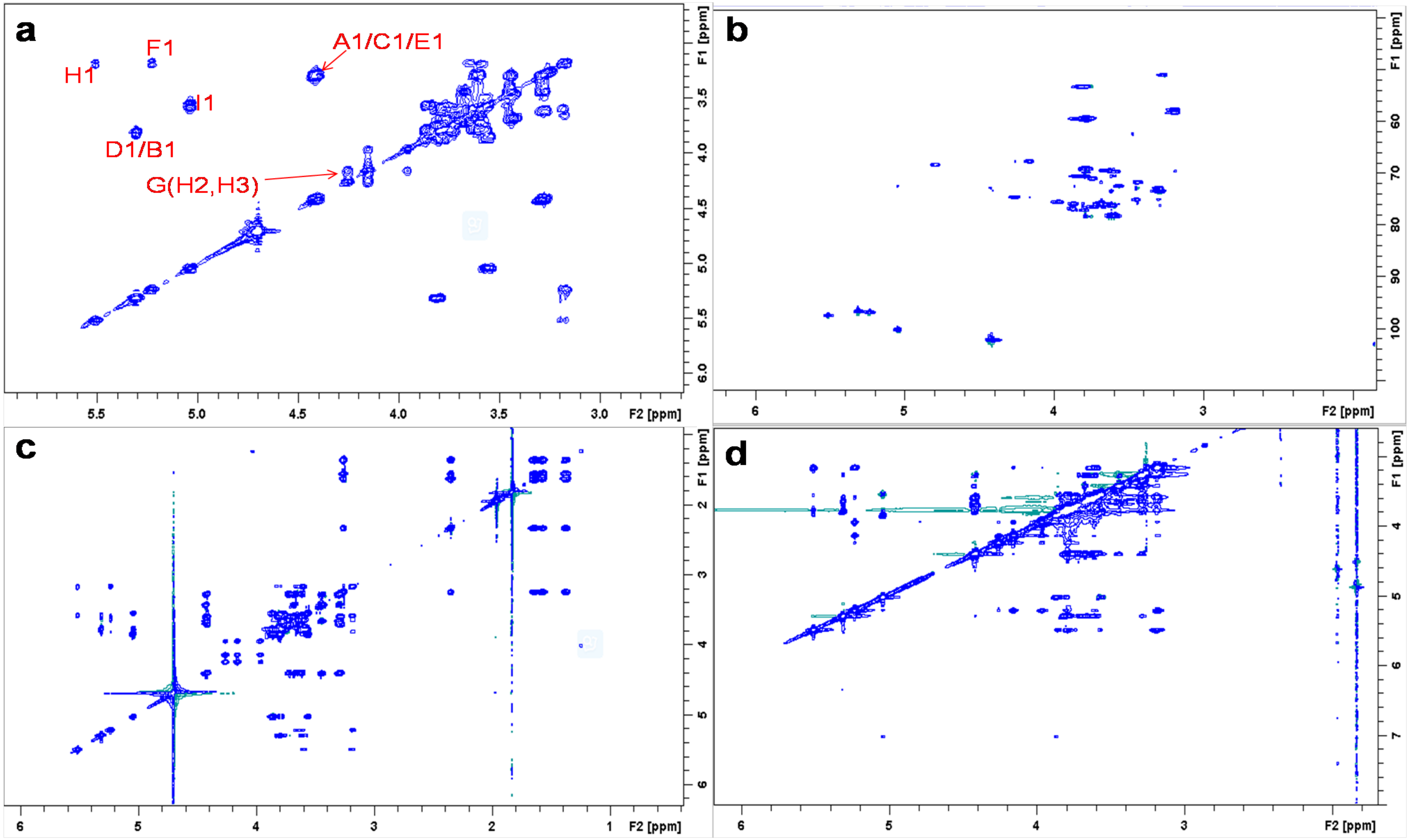

Supplementary Fig. S171. 2D NMR COSY (a), HSQC (b), TOCSY (c) and NOE (d) spectra of compound **57.**

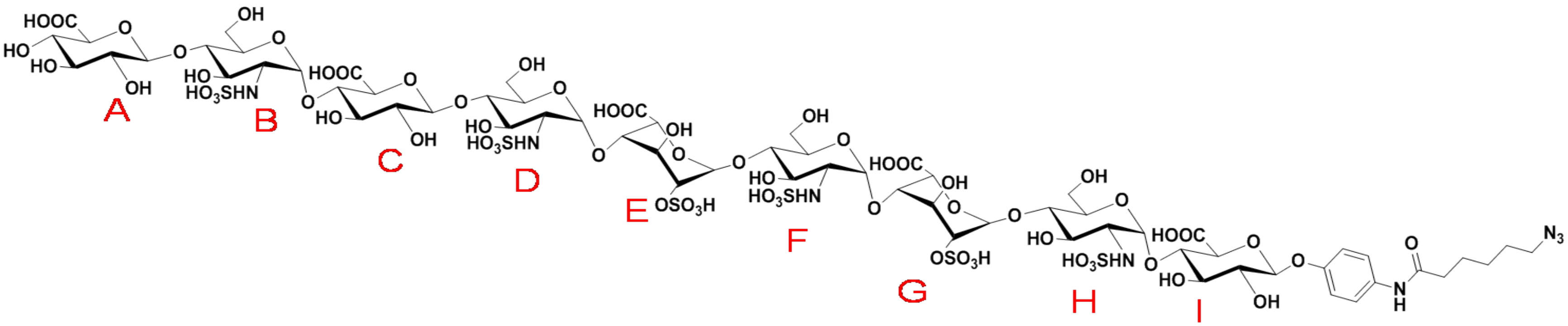

	1	2	3	4	5	6a	6b
A	4.43	3.31	3.44	3.45	3.68	----------	----------
B	5.55	3.19	3.61	3.74	3.74	3.74	3.80
C	4.45	3.32	3.73	3.77	3.77	----------	----------
D	5.24	3.18	3.62	3.78	3.78	3.66	3.82
E	5.19	4.26	4.16	3.96	4.80	----------	----------
F	5.24	3.18	3.62	3.78	3.78	3.66	3.82
G	5.19	4.26	4.16	3.96	4.80	----------	----------
H	5.50	3.19	3.60	3.65	3.70	3.70	3.74
I	5.04	3.56	3.82	3.77	3.87	----------	----------

Supplementary Fig. S172. ^{1}H NMR chemical shift assignments (in ppm) of compound **58**. The structure of compound **58** is shown. The protons of *p*NA-N_3 are not listed in the table.

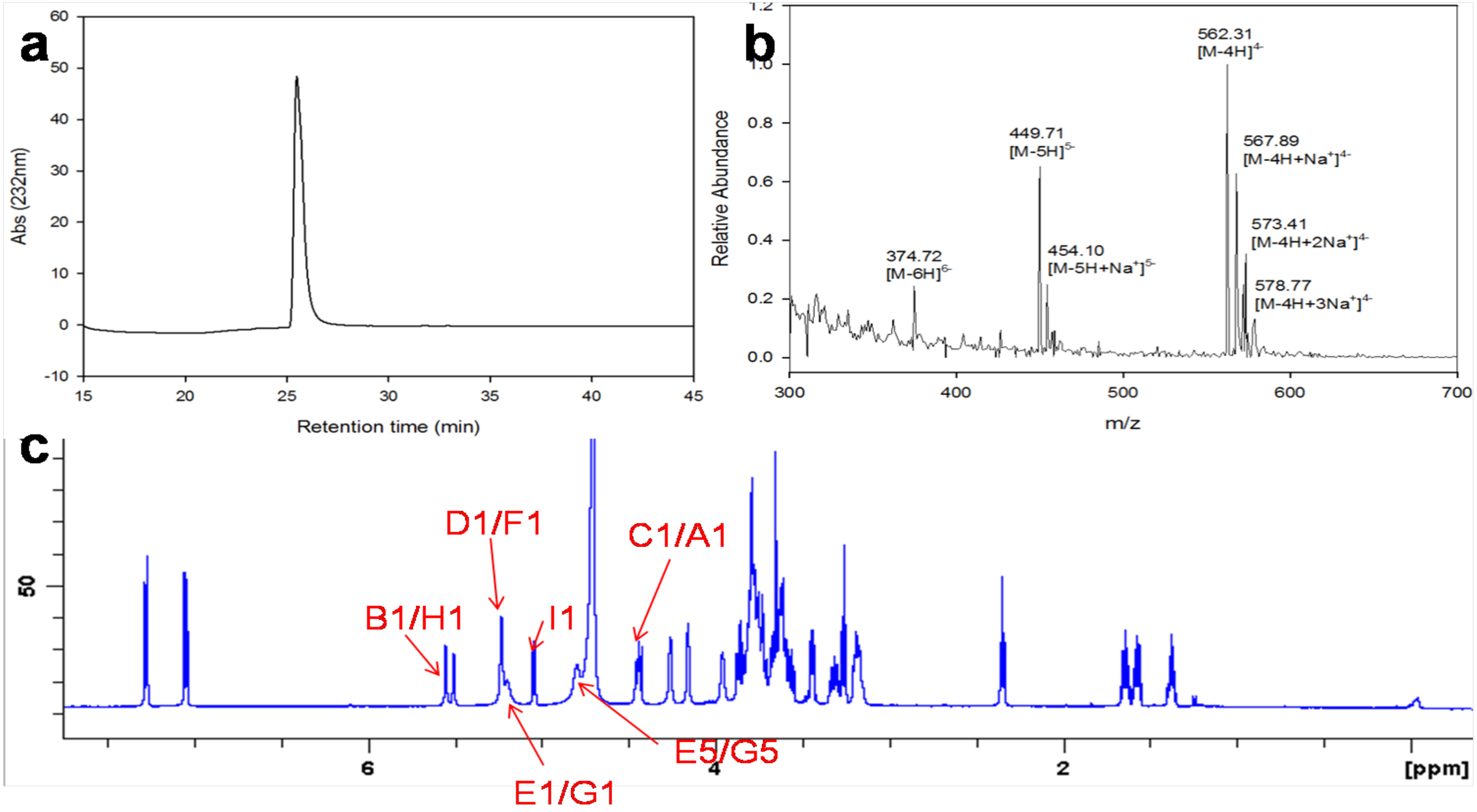

Supplementary Fig. S173. HPLC analysis (a), MS spectrum (b) and ^{1}H NMR spectrum (c) of compound **58**.

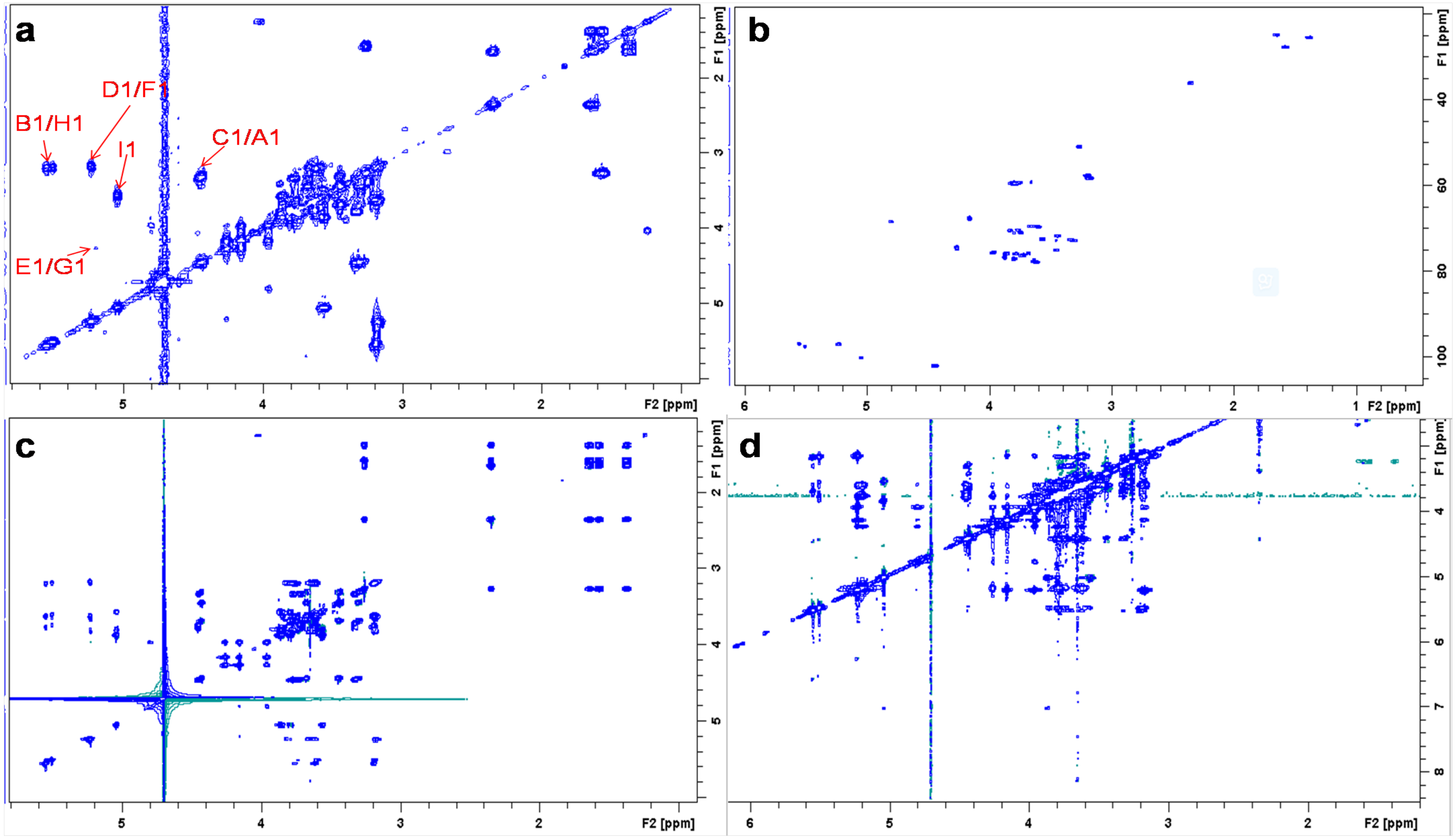

Supplementary Fig. S174. 2D NMR COSY (a), HSQC (b), TOCSY (c) and NOE (d) spectra of compound **58.**

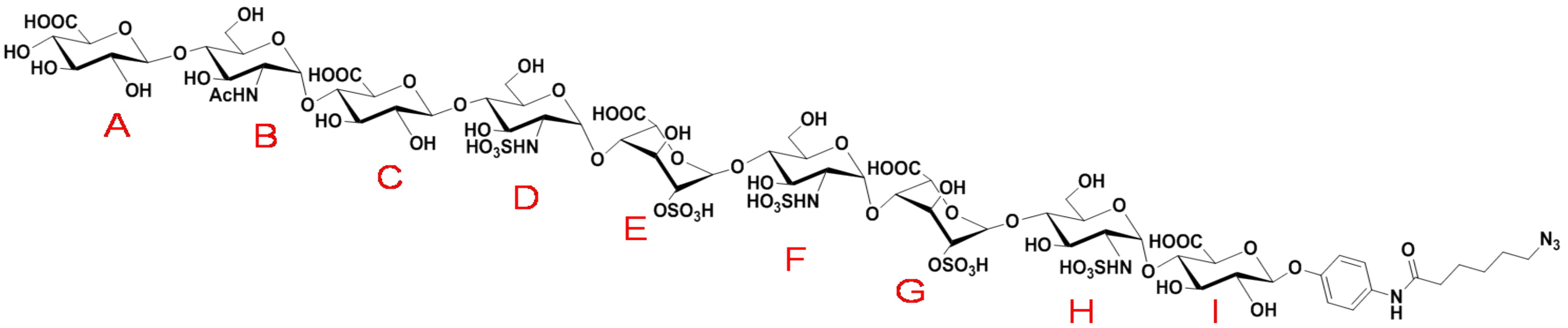

	1	2	3	4	5	6a	6b
A	4.43	3.30	3.45	3.46	3.68	----------	----------
B	5.32	3.82	3.65	3.77	3.72	3.72	3.80
C	4.42	3.30	3.62	3.67	3.72	----------	----------
D	5.23	3.17	3.62	3.65	3.79	3.72	3.83
E	5.18	4.26	4.16	3.96	4.79	----------	----------
F	5.23	3.17	3.62	3.65	3.79	3.72	3.83
G	5.18	4.26	4.16	3.96	4.79	----------	----------
H	5.52	3.19	3.61	3.63	3.80	3.63	3.74
I	5.05	3.58	3.86	3.79	3.89	----------	----------

Supplementary Fig. S175. ^{1}H NMR chemical shift assignments (in ppm) of compound **59**. The structure of compound **59** is shown. The protons of *p*NA-N_3 are not listed in the table.

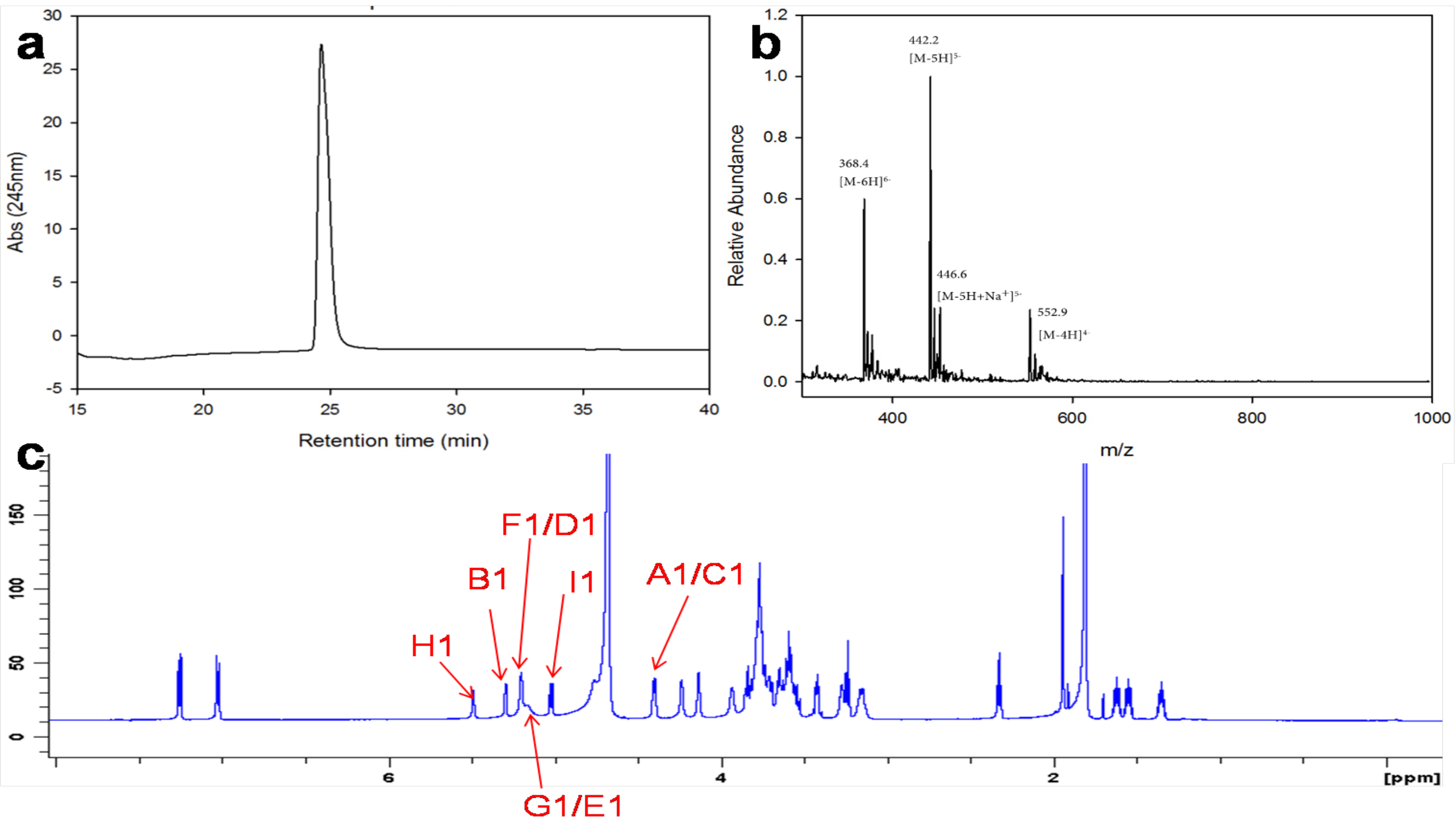

Supplementary Fig. S176. HPLC analysis (a), MS spectrum (b) and ^{1}H NMR spectrum (c) of compound **59**.

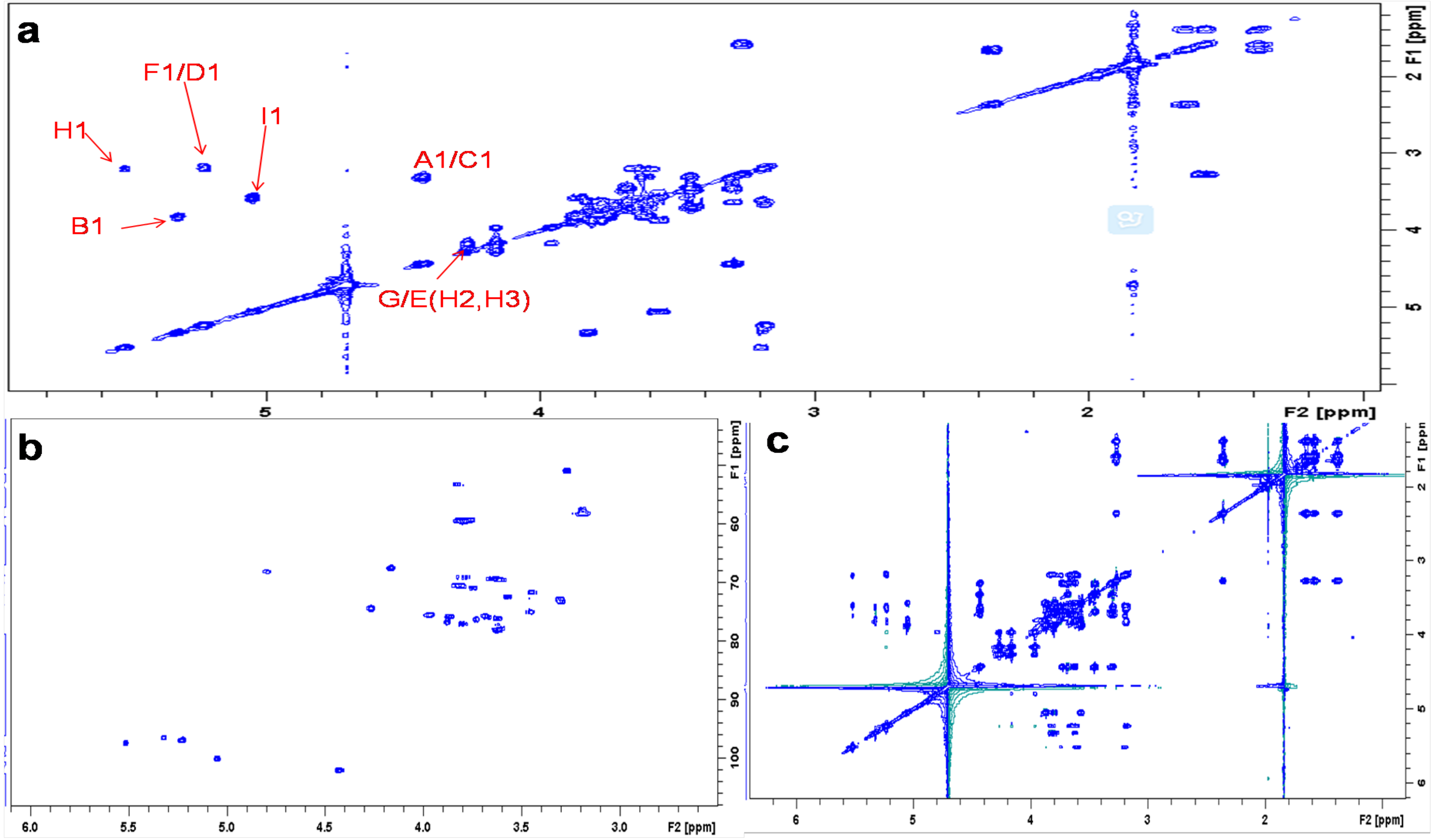

Supplementary Fig. S177. 2D NMR COSY (a), HSQC (b) and TOCSY (c) spectra of compound **59.**

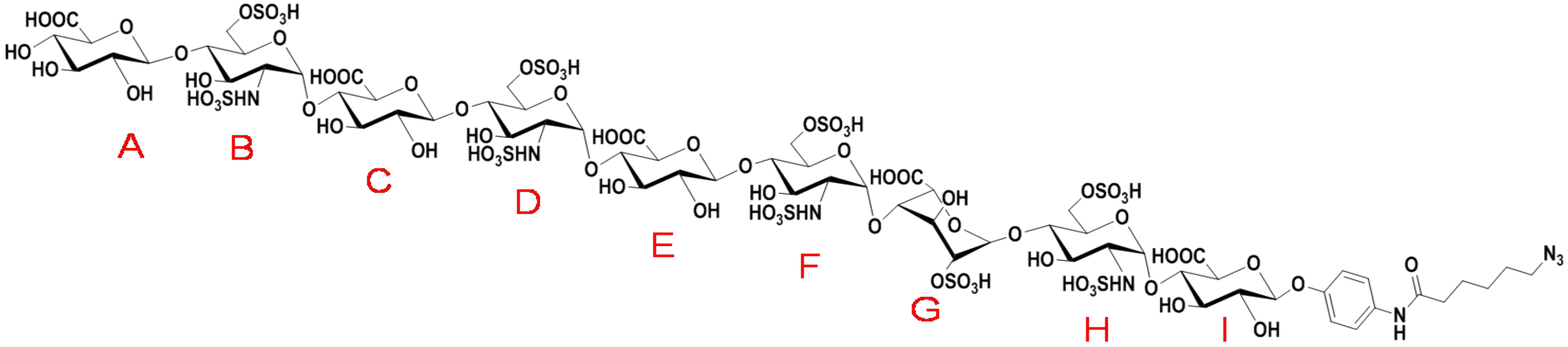

	1	2	3	4	5	6a	6b
A	4.51	3.27	3.46	3.41	3.68	----------	----------
B	5.54	3.22	3.61	3.68	3.95	4.10	4.39
C	4.53	3.31	3.78	3.70	3.74	----------	----------
D	5.54	3.22	3.61	3.68	3.95	4.10	4.39
E	4.53	3.31	3.78	3.70	3.74	----------	----------
F	5.34	3.20	3.62	3.66	4.01	4.16	4.37
G	5.16	4.25	4.12	4.02	4.73	----------	----------
H	5.52	3.22	3.59	3.71	3.88	4.15	4.33
I	5.04	3.57	3.86	3.80	3.87	----------	----------

Supplementary Fig. S178. ^{1}H NMR chemical shift assignments (in ppm) of compound **60**. The structure of compound **60** is shown. The protons of *p*NA-N_3 are not listed in the table.

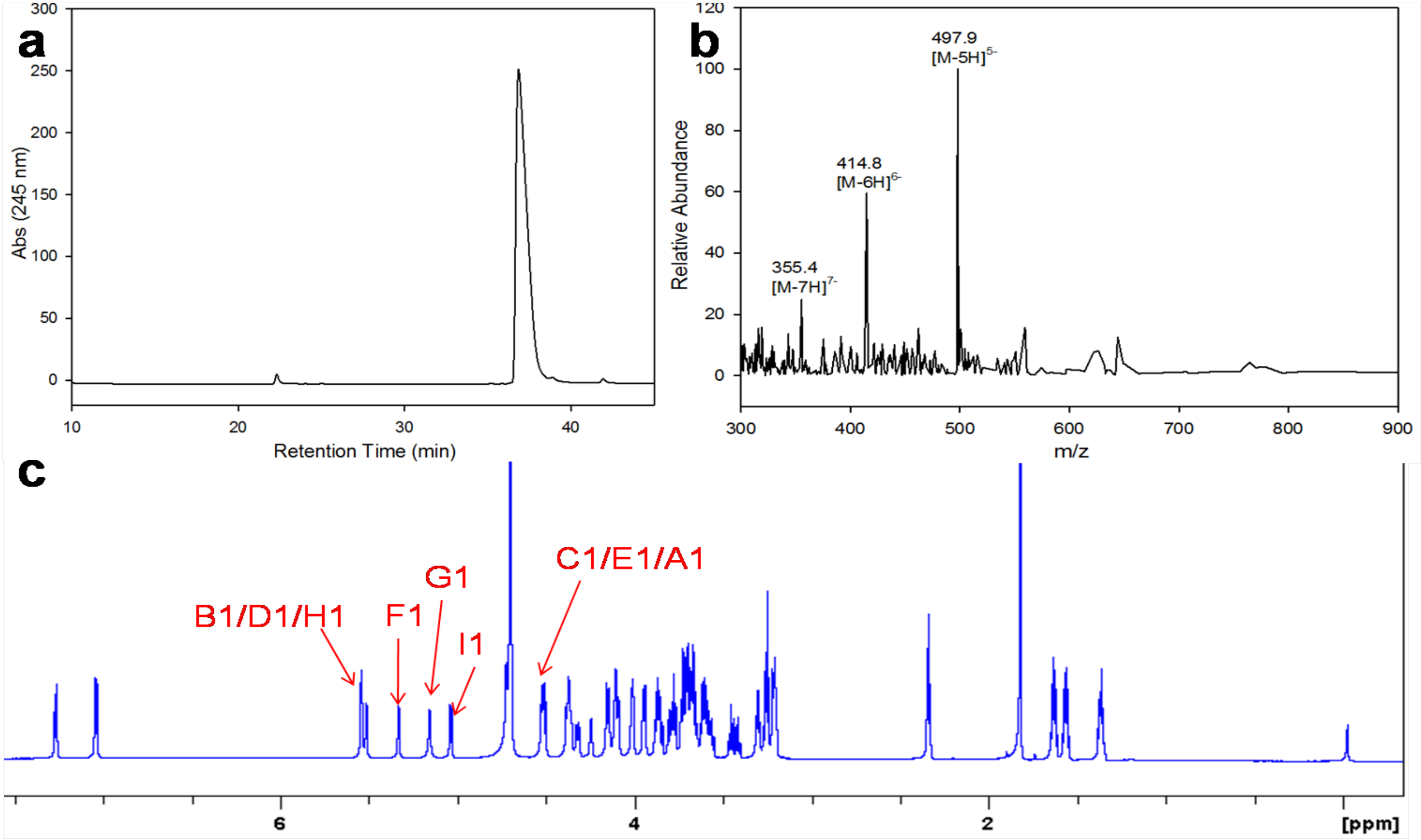

Supplementary Fig. S179. HPLC analysis (a), MS spectrum (b) and ^{1}H NMR spectrum (c) of compound **60**.

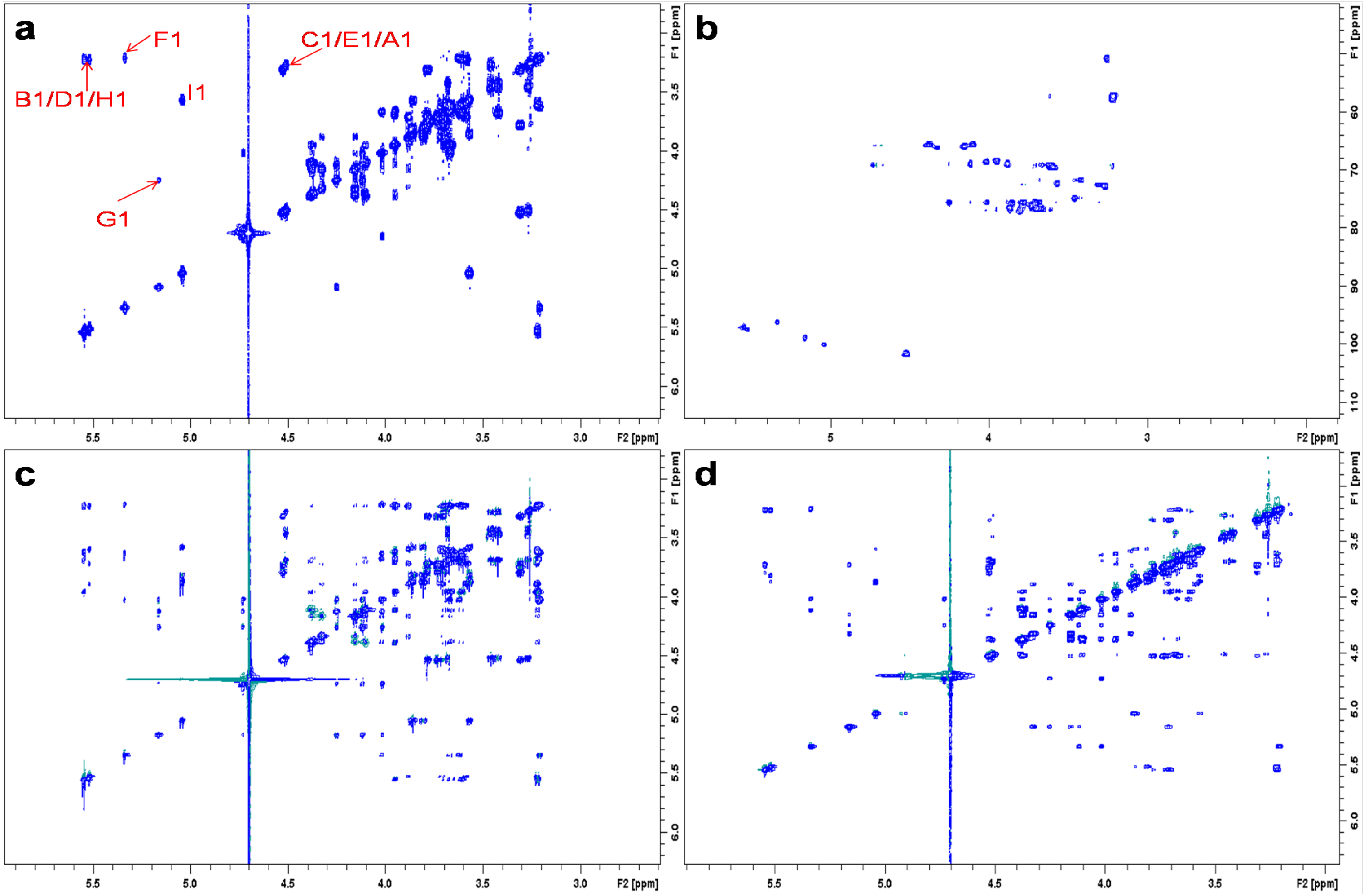

Supplementary Fig. S180. 2D NMR COSY (a), HSQC (b), TOCSY (c) and NOE (d) spectra of compound **60.**

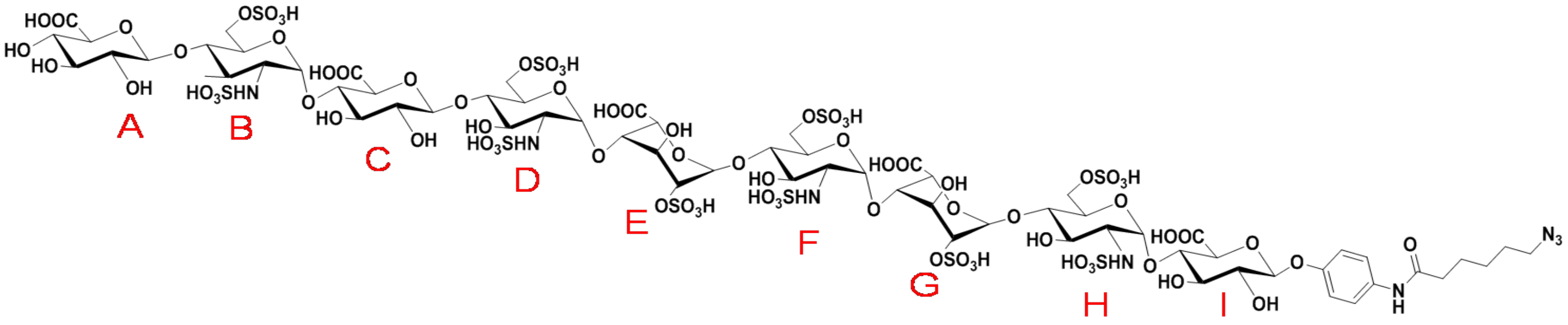

	1	2	3	4	5	6a	6b
A	4.53	3.27	3.46	3.47	3.77	----------	----------
B	5.52	3.21	3.61	3.68	3.89	4.10	4.36
C	4.55	3.32	3.78	3.78	3.85	----------	----------
D	5.32	3.22	3.61	3.67	3.92	4.15	4.36
E	5.21	4.26	4.18	4.06	4.97	----------	----------
F	5.32	3.22	3.61	3.67	3.92	4.15	4.36
G	5.21	4.26	4.18	4.06	4.97	----------	----------
H	5.53	3.21	3.61	3.68	3.91	4.15	4.29
I	5.08	3.57	3.88	3.83	4.01	----------	----------

Supplementary Fig. S181. ^{1}H NMR chemical shift assignments (in ppm) of compound **61**. The structure of compound **61** is shown. The protons of *p*NA-N_3 are not listed in the table.

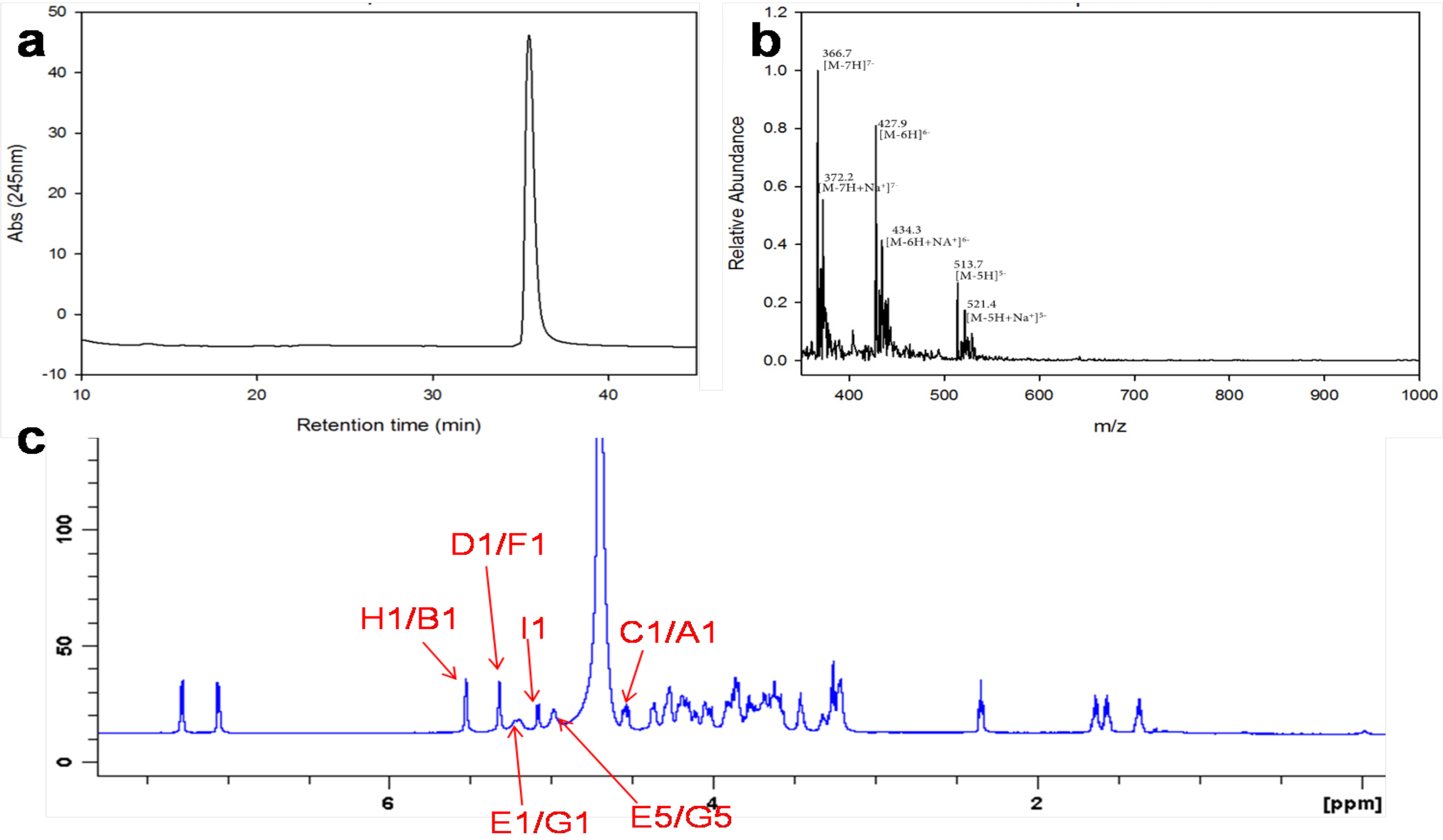

Supplementary Fig. S182. HPLC analysis (a), MS spectrum (b) and ^{1}H NMR spectrum (c) of compound **61**.

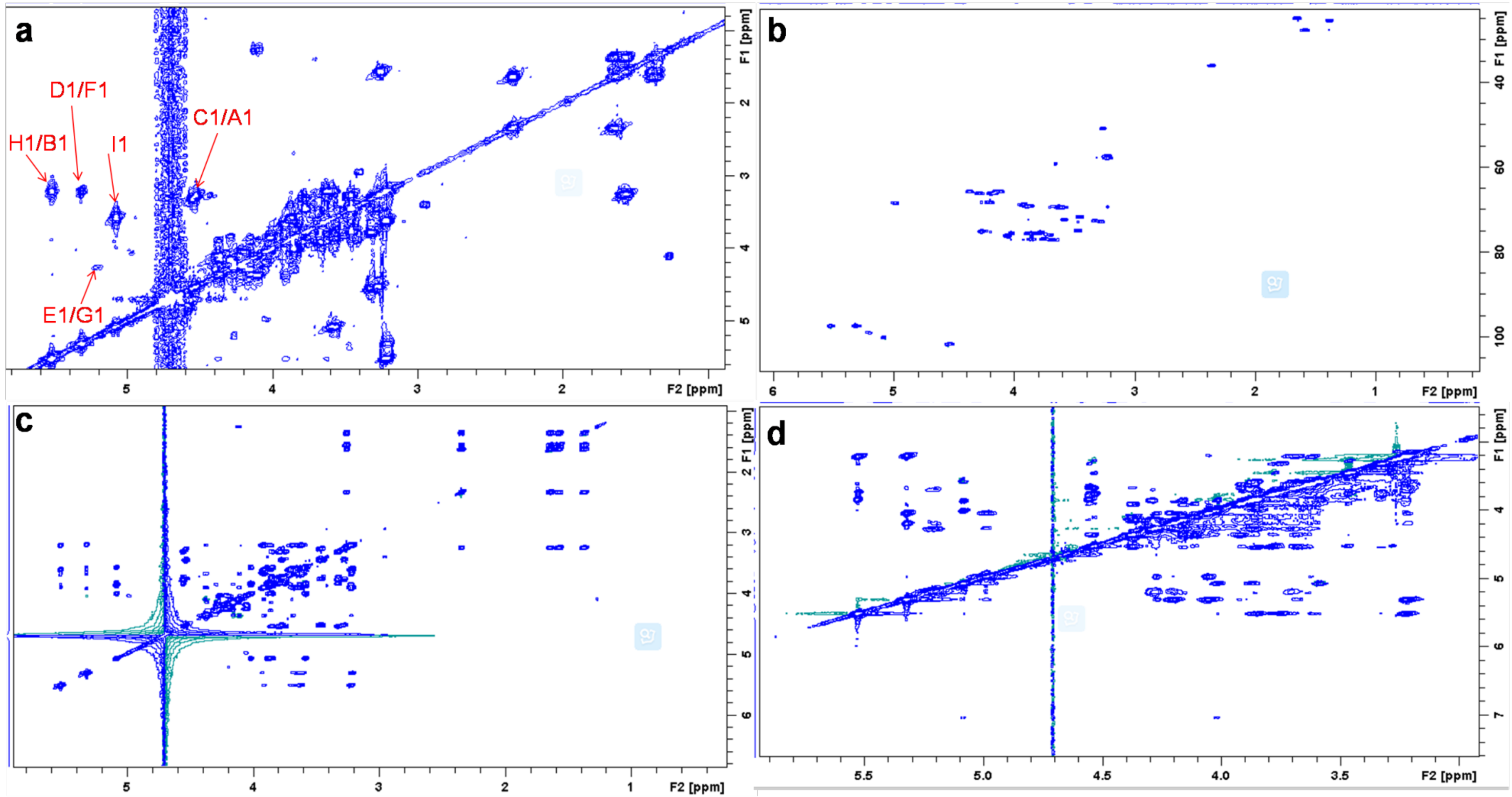

Supplementary Fig. S183. 2D NMR COSY (a), HSQC (b), TOCSY (c) and NOE (d) spectra of compound **61.**

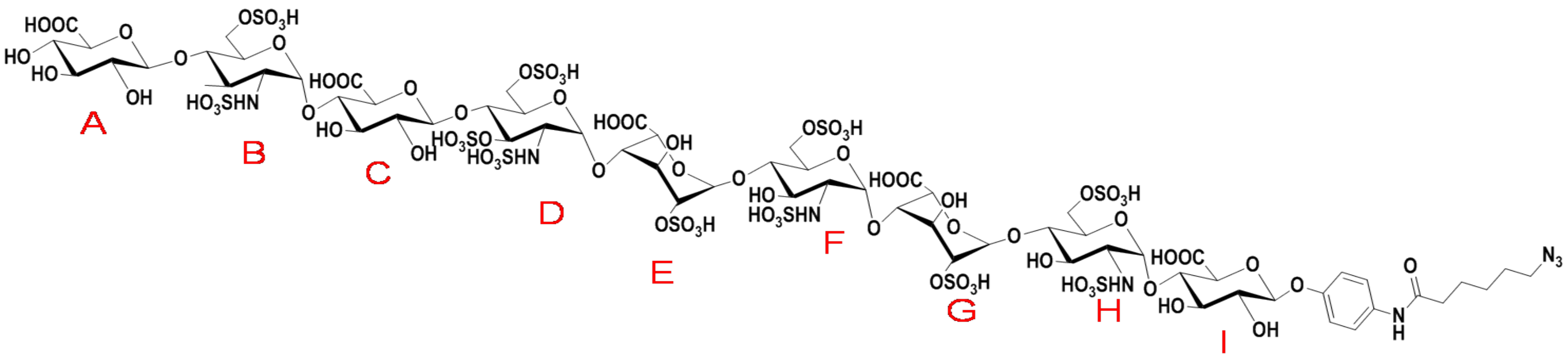

	1	2	3	4	5	6a	6b
A	4.47	3.22	3.40	3.39	3.63	----------	----------
B	5.49	3.17	3.55	3.63	3.89	4.04	4.34
C	4.49	3.28	3.71	3.63	3.72	----------	----------
D	5.37	3.32	4.25	3.82	4.02	4.12	4.35
E	5.09	4.19	4.07	4.01	4.68	----------	----------
F	5.26	3.14	3.54	3.67	3.90	4.10	4.32
G	5.15	4.21	4.07	3.96	4.71	----------	----------
H	5.47	3.17	3.55	3.68	3.84	4.11	4.26
I	5.00	3.50	3.79	3.73	3.82	----------	----------

Supplementary Fig. S184. ^{1}H NMR chemical shift assignments (in ppm) of compound **62**. The structure of compound **62** is shown. The protons of *p*NA-N_3 are not listed in the table.

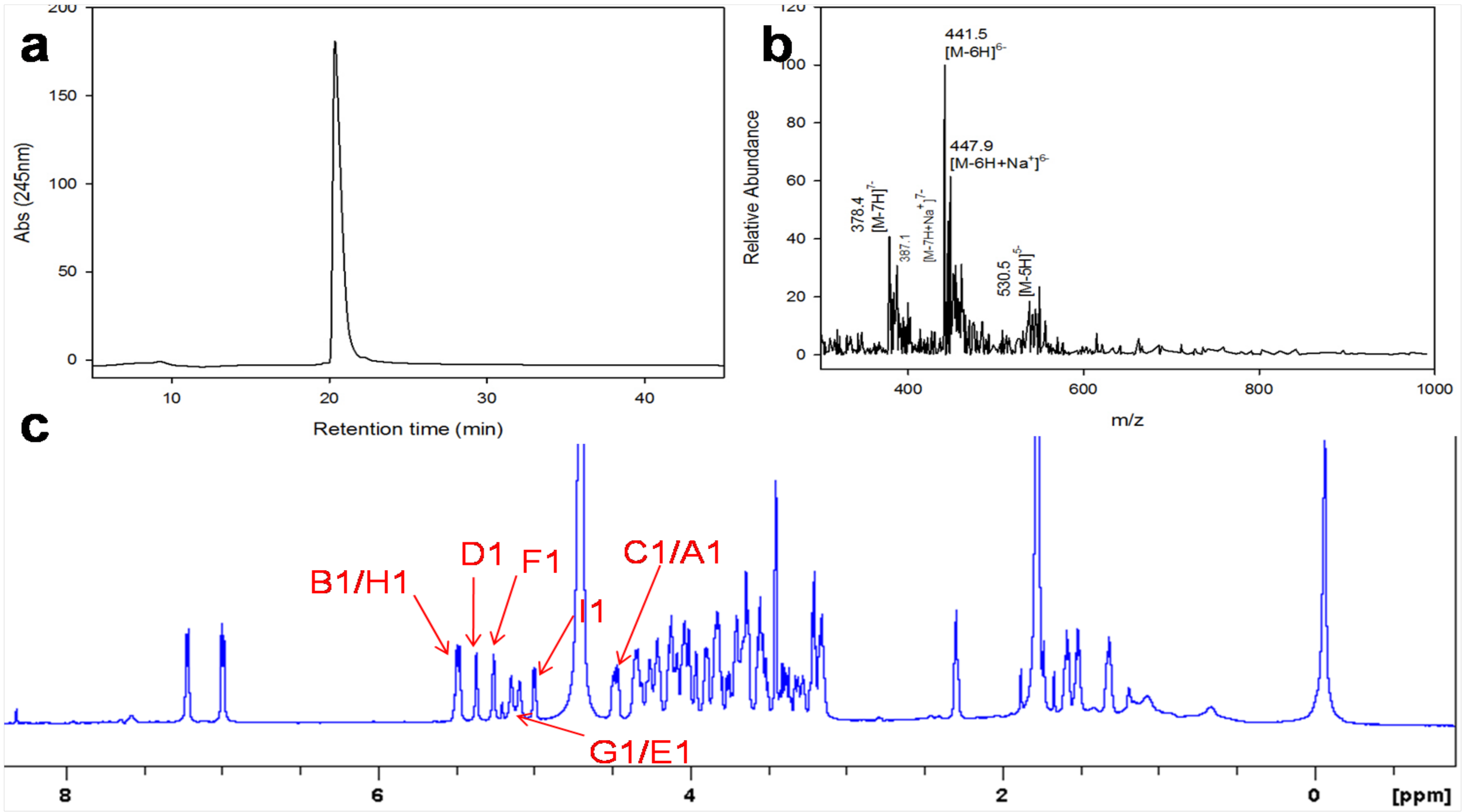

Supplementary Fig. S185. HPLC analysis (a), MS spectrum (b) and ^{1}H NMR spectrum (c) of compound **62**.

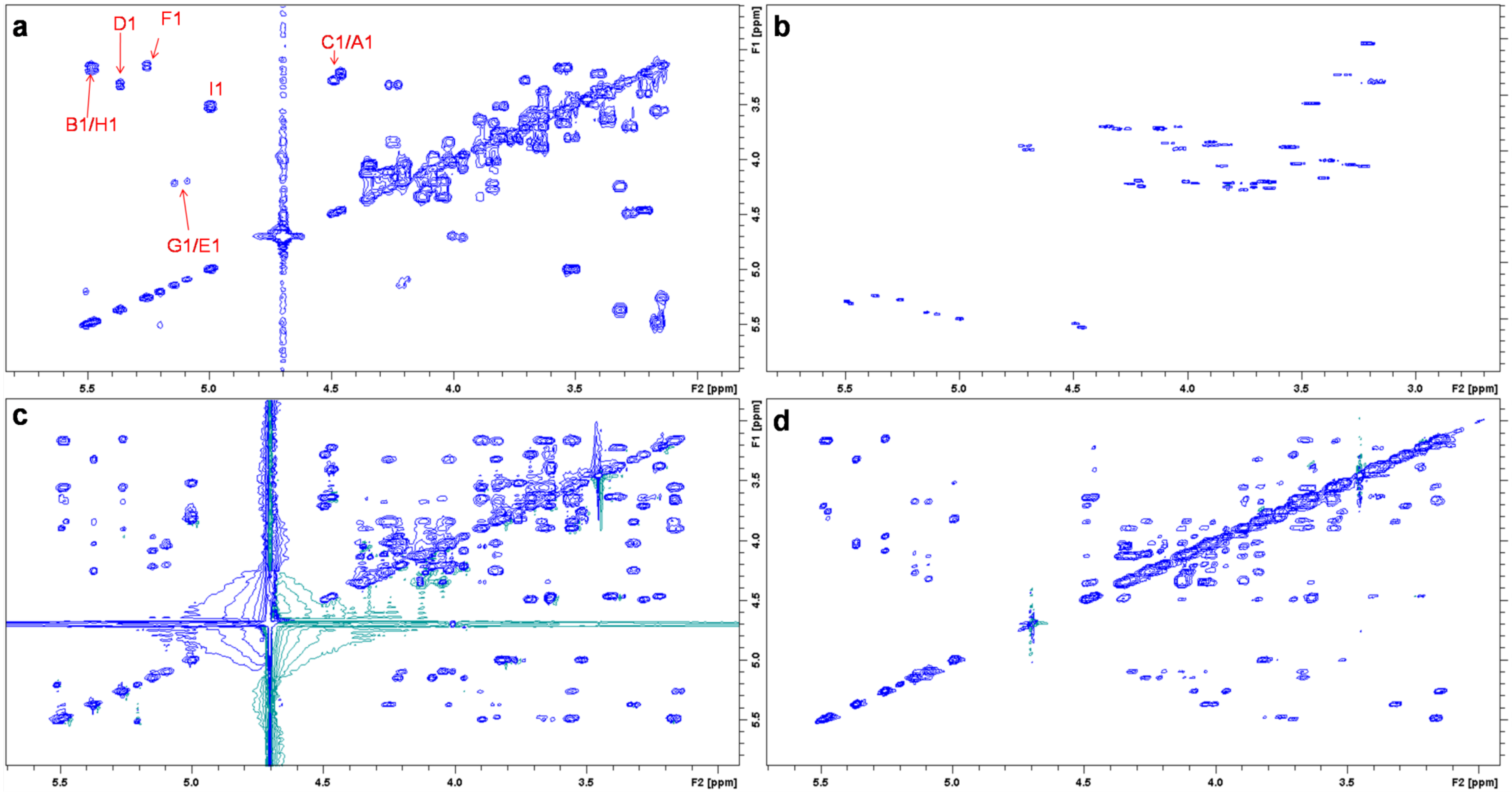

Supplementary Fig. S186. 2D NMR COSY (a), HSQC (b), TOCSY (c) and NOE (d) spectra of compound **62.**

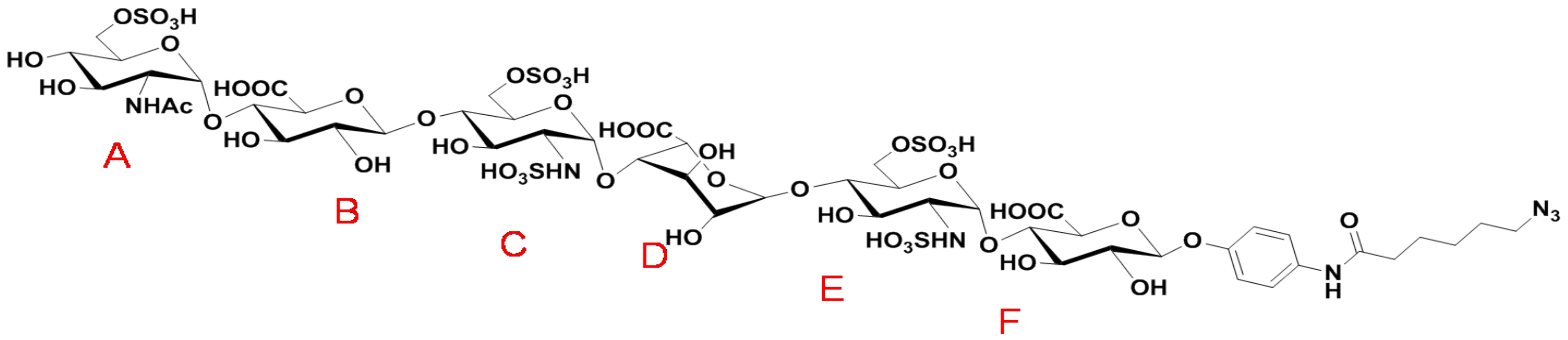

	1	2	3	4	5	6a	6b
A	5.35	3.83	3.49	3.66	3.83	4.08	4.28
B	4.51	3.26	3.63	3.68	3.73	----------	----------
C	5.25	3.16	3.60	3.66	3.95	4.10	4.40
D	4.94	3.86	4.03	3.98	4.75	----------	----------
E	5.54	3.21	3.58	3.66	3.91	4.12	4.25
F	5.04	3.57	3.85	3.79	3.87	----------	----------

Supplementary Fig. S187. ^{1}H NMR chemical shift assignments (in ppm) of compound **63**. The structure of compound **63** is shown. The protons of *p*NA-N_3 are not listed in the table.

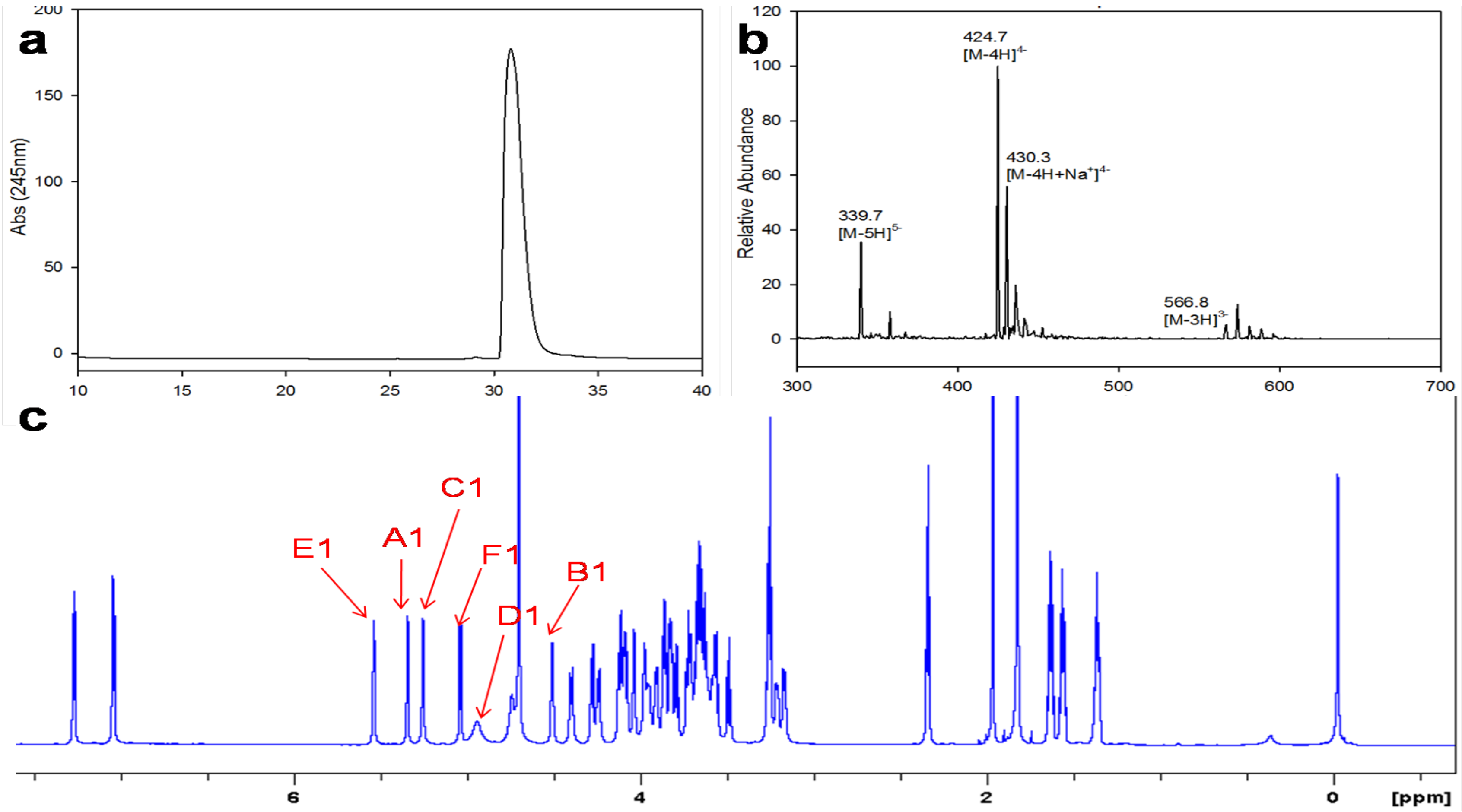

Supplementary Fig. S188. HPLC analysis (a), MS spectrum (b) and ^{1}H NMR spectrum (c) of compound **63**.

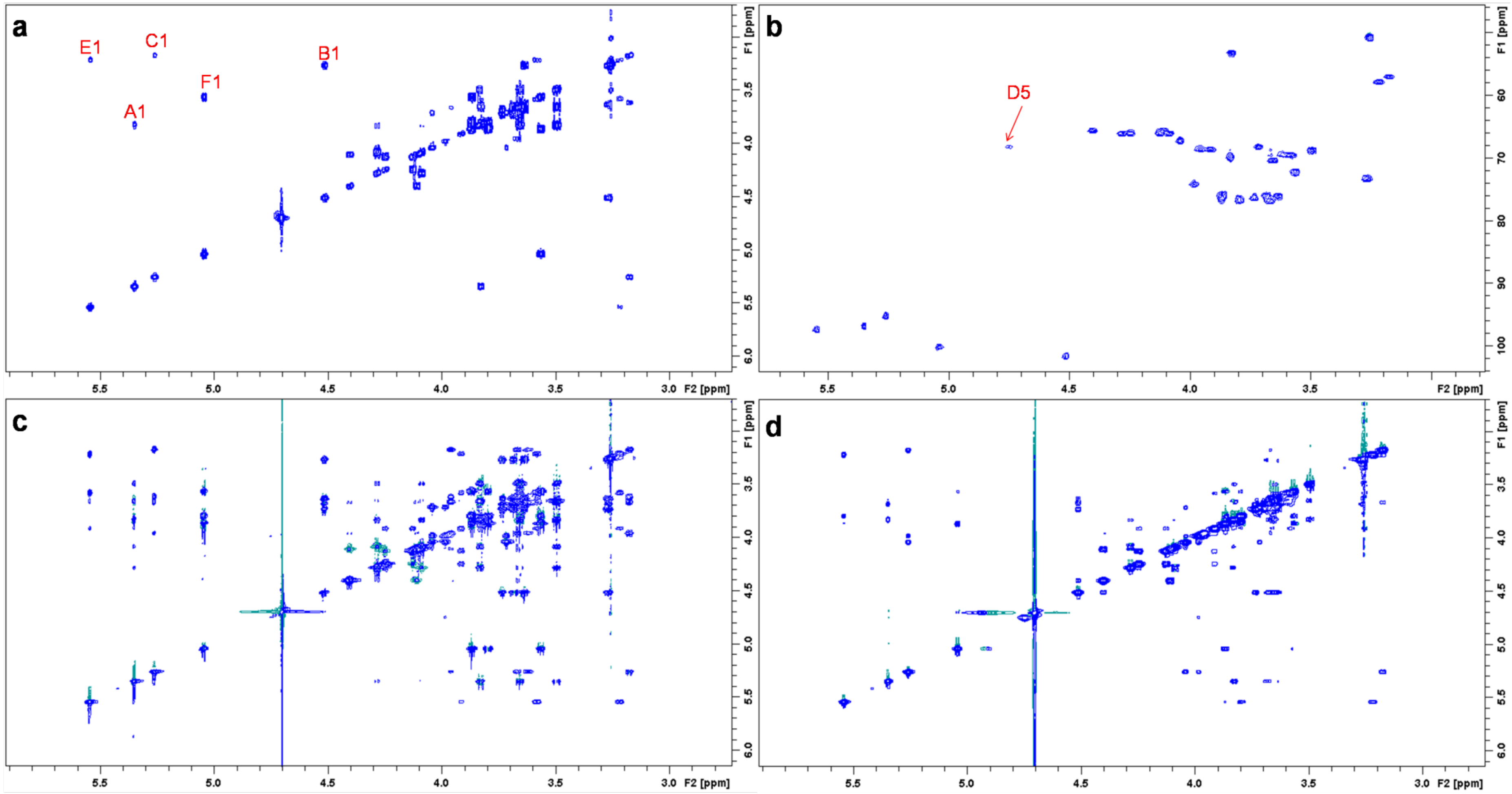

Supplementary Fig. S189. 2D NMR COSY (a), HSQC (b), TOCSY (c) and NOE (d) spectra of compound **63.**

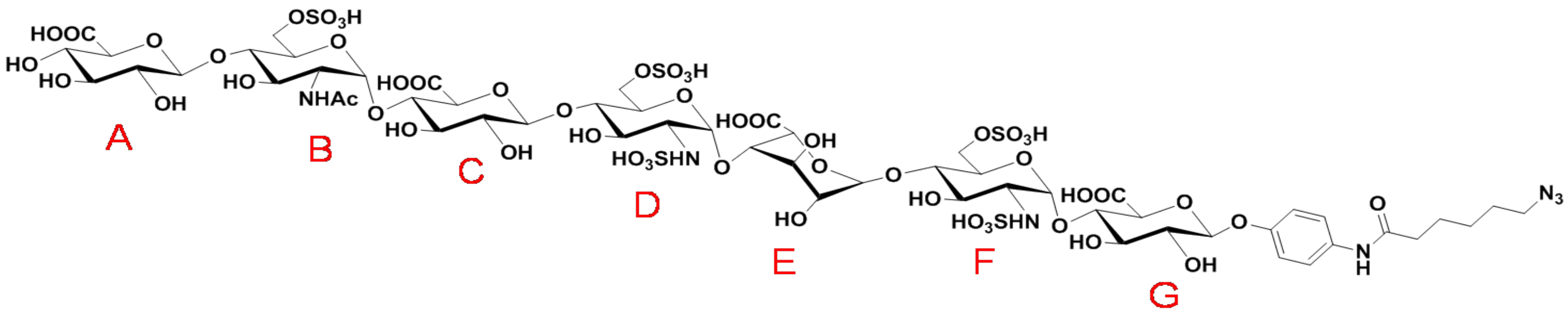

	1	2	3	4	5	6a	6b
A	4.50	3.27	3.46	3.42	3.69	----------	----------
B	5.34	3.85	3.76	3.69	3.98	4.11	4.38
C	4.52	3.26	3.64	3.68	3.74	----------	----------
D	5.26	3.18	3.61	3.66	3.96	4.11	4.40
E	4.95	3.71	4.04	3.98	4.74	----------	----------
F	5.54	3.22	3.58	3.66	3.91	4.13	4.25
G	5.04	3.58	3.86	3.80	3.88	----------	----------

Supplementary Fig. S190. ^{1}H NMR chemical shift assignments (in ppm) of compound **64**. The structure of compound **64** is shown. The protons of *p*NA-N_3 are not listed in the table.

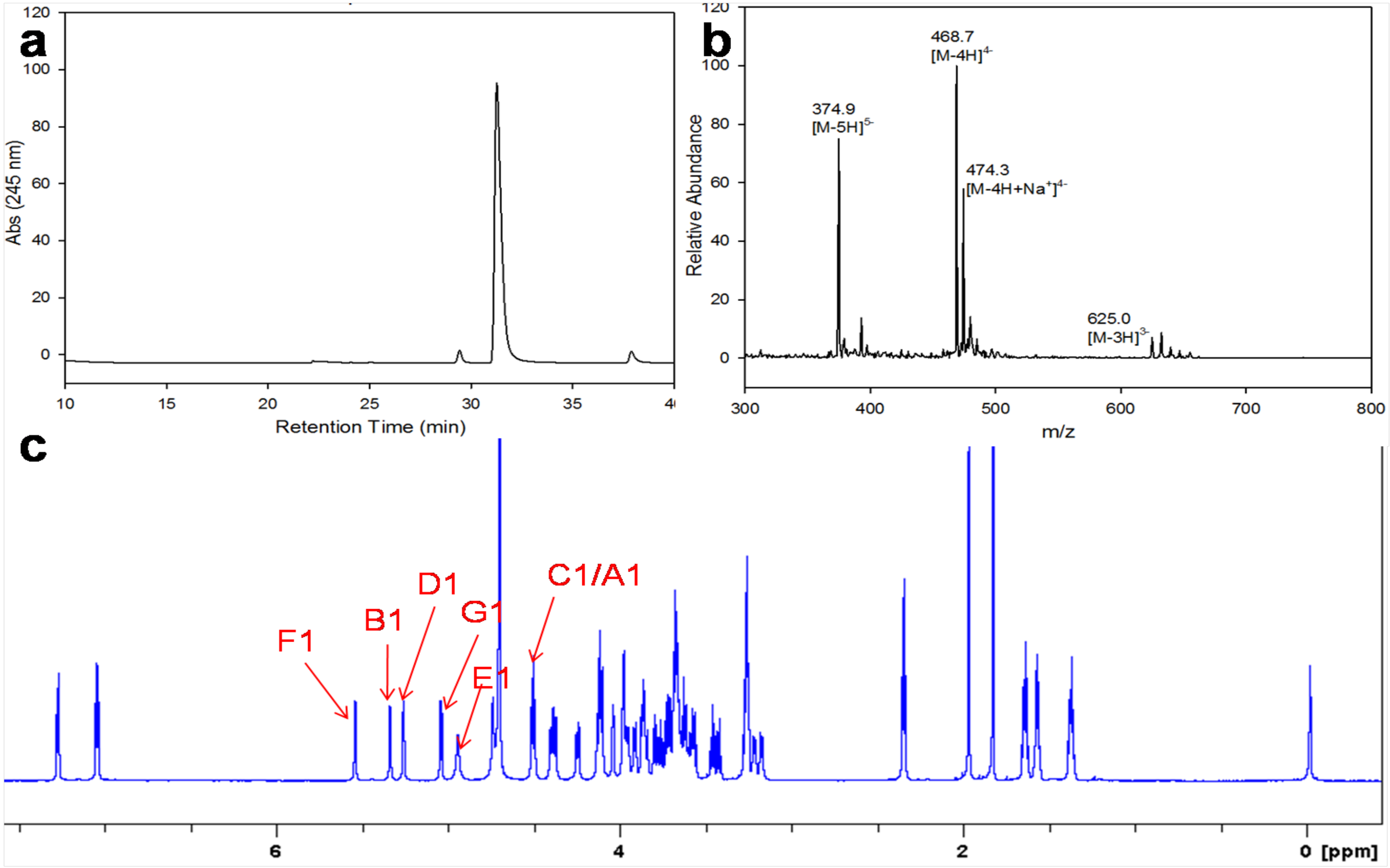

Supplementary Fig. S191. HPLC analysis (a), MS spectrum (b) and ^{1}H NMR spectrum (c) of compound **64**.

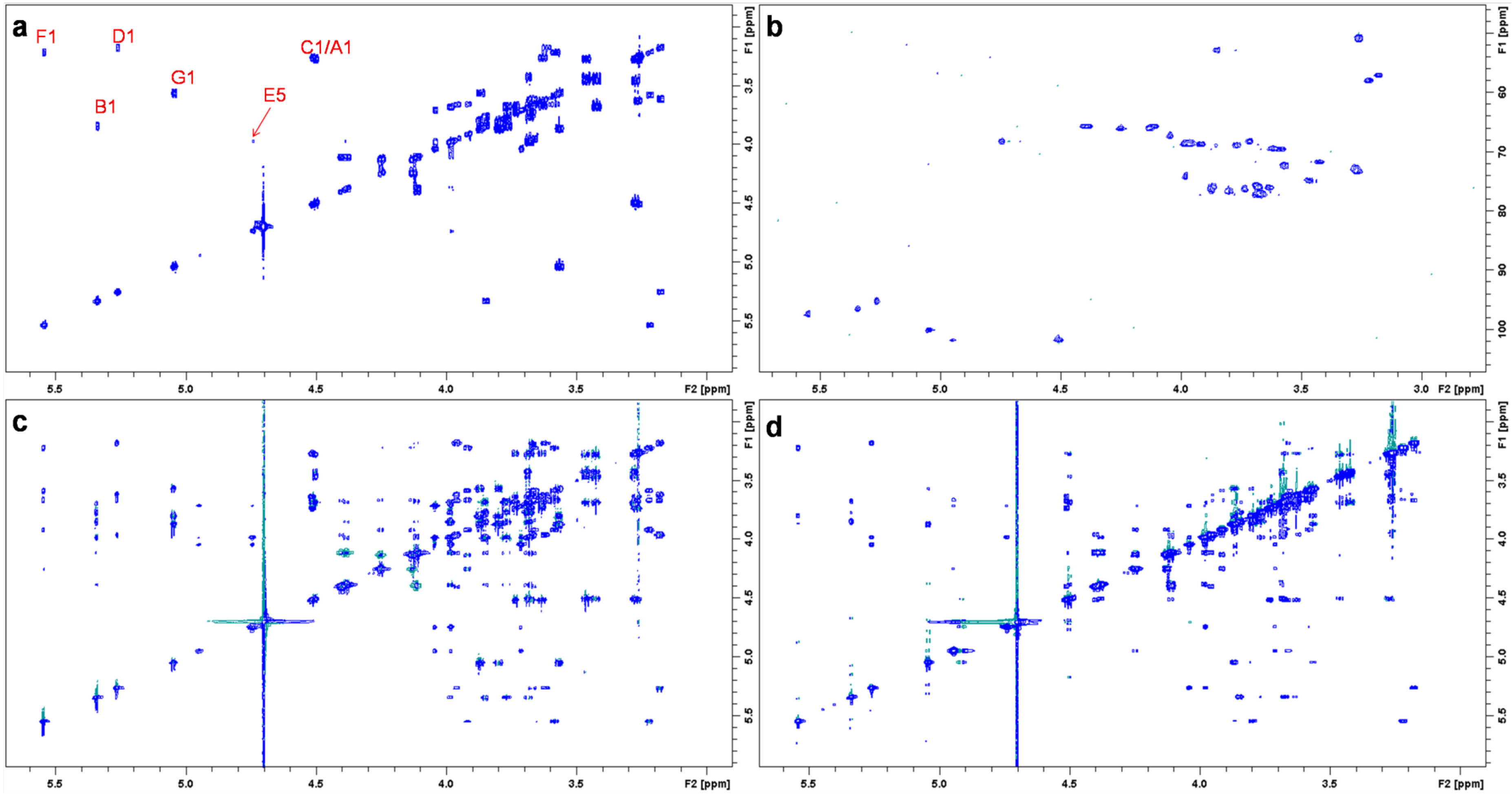

Supplementary Fig. S192. 2D NMR COSY (a), HSQC (b), TOCSY (c) and NOE (d) spectra of compound **64.**

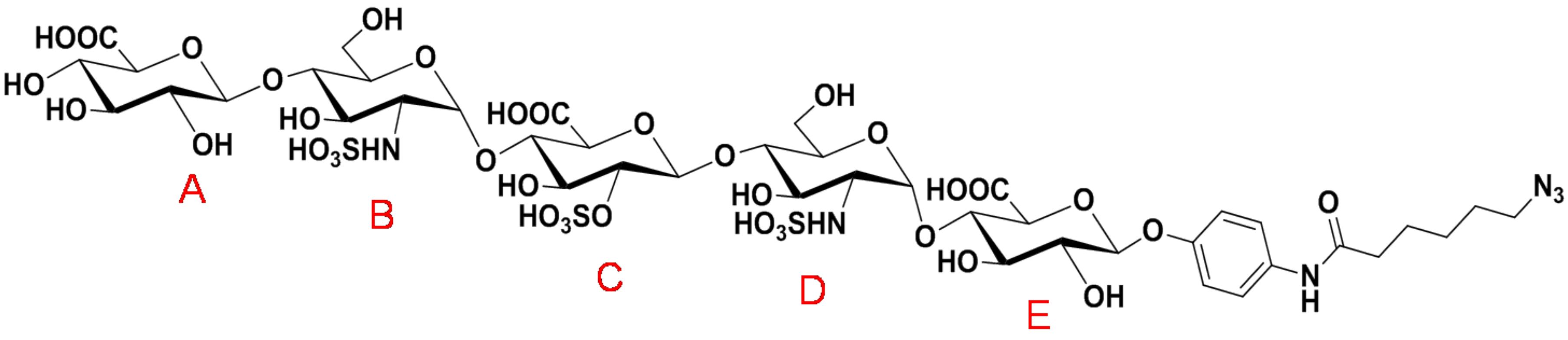

	1	2	3	4	5	6a	6b
A	4.45	3.29	3.43	3.45	3.75	----------	----------
B	5.56	3.16	3.61	3.65	3.70	3.74	3.80
C	4.65	4.05	3.95	3.79	3.85	----------	----------
D	5.48	3.19	3.65	3.60	3.68	3.74	3.91
E	5.08	3.57	3.86	3.83	4.02	----------	----------

Supplementary Fig. S193. ^{1}H NMR chemical shift assignments (in ppm) of compound **65**. The structure of compound **65** is shown. The protons of *p*NA-N_3 are not listed in the table.

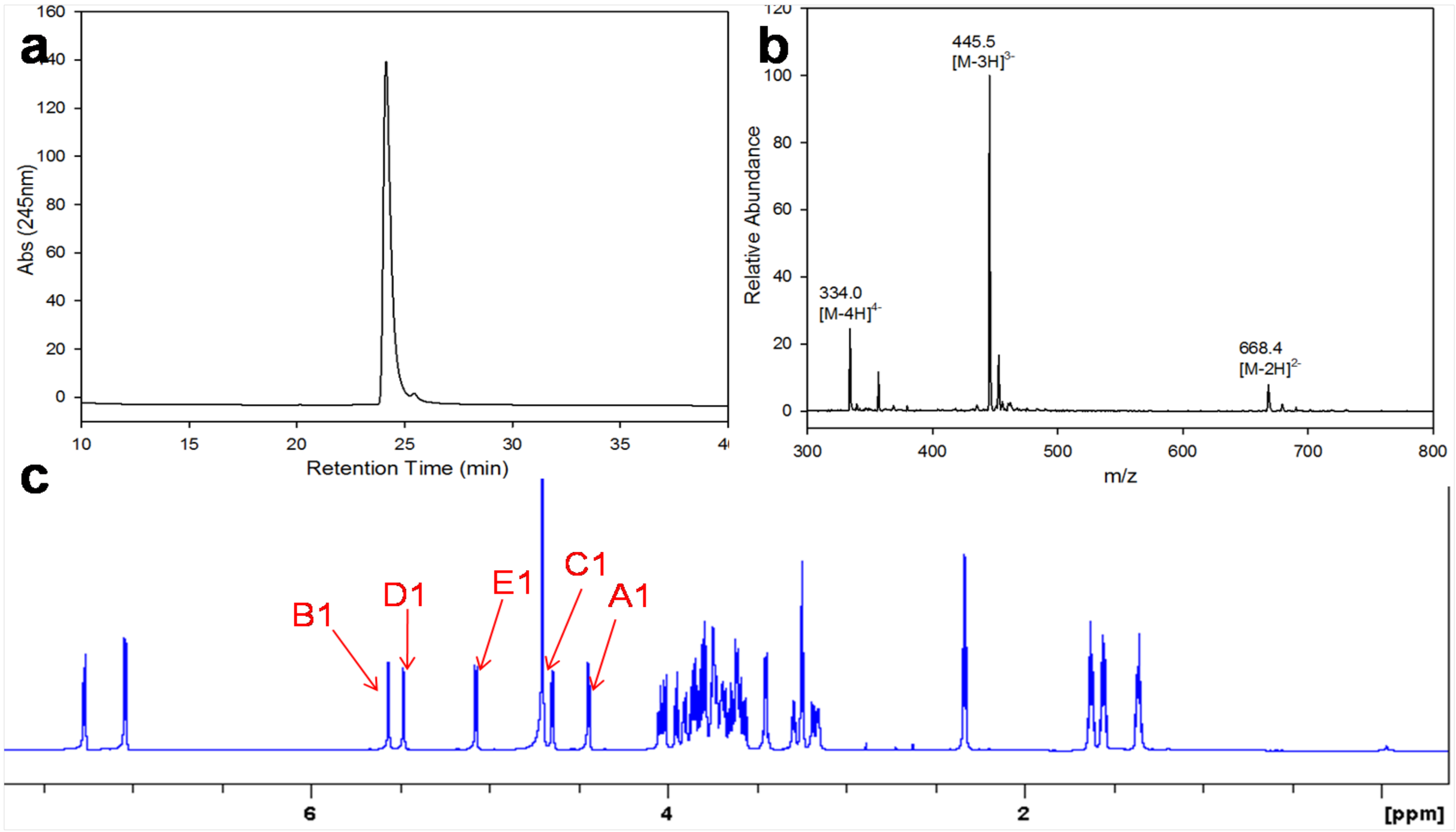

Supplementary Fig. S194. HPLC analysis (a), MS spectrum (b) and ^{1}H NMR spectrum (c) of compound **65**.

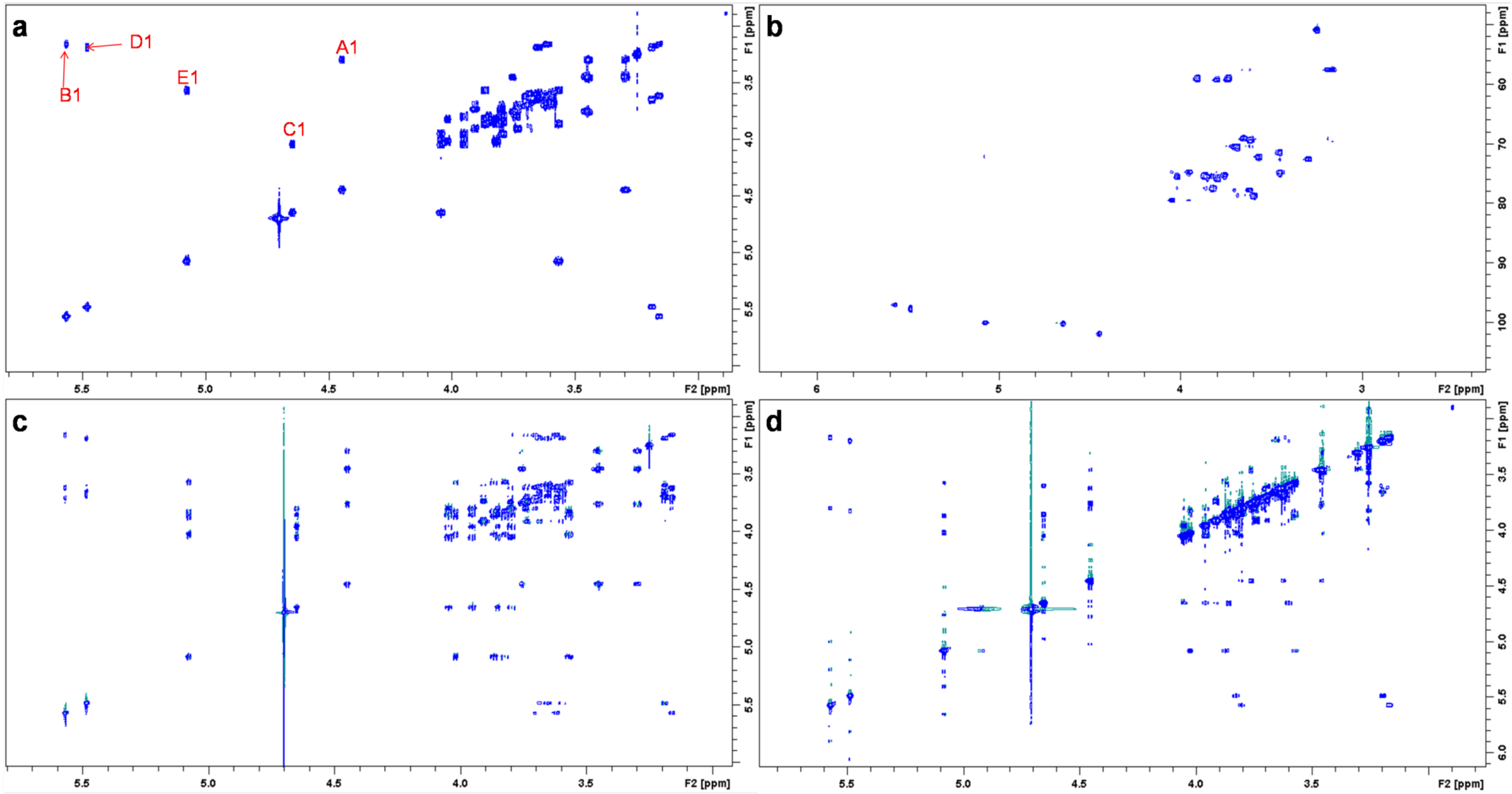

Supplementary Fig. S195. 2D NMR COSY (a), HSQC (b), TOCSY (c) and NOE (d) spectra of compound **65.**

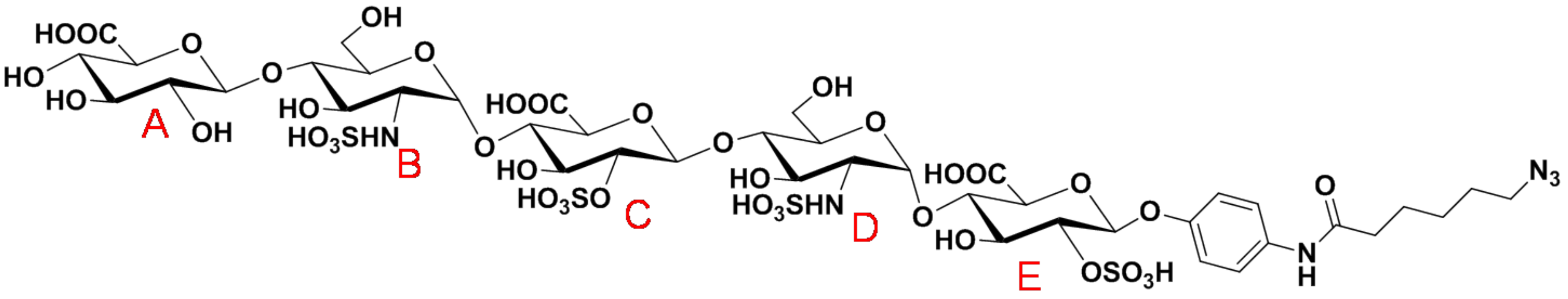

	1	2	3	4	5	6a	6b
A	4.45	3.29	3.43	3.45	3.77	----------	----------
B	5.57	3.15	3.60	3.64	3.68	3.73	3.79
C	4.65	4.04	3.94	3.80	3.86	----------	----------
D	5.51	3.16	3.65	3.60	3.68	3.72	3.90
E	5.23	4.30	4.02	3.91	4.07	----------	----------

Supplementary Fig. S196. ^{1}H NMR chemical shift assignments (in ppm) of compound **66**. The structure of compound **66** is shown. The protons of *p*NA-N_3 are not listed in the table.

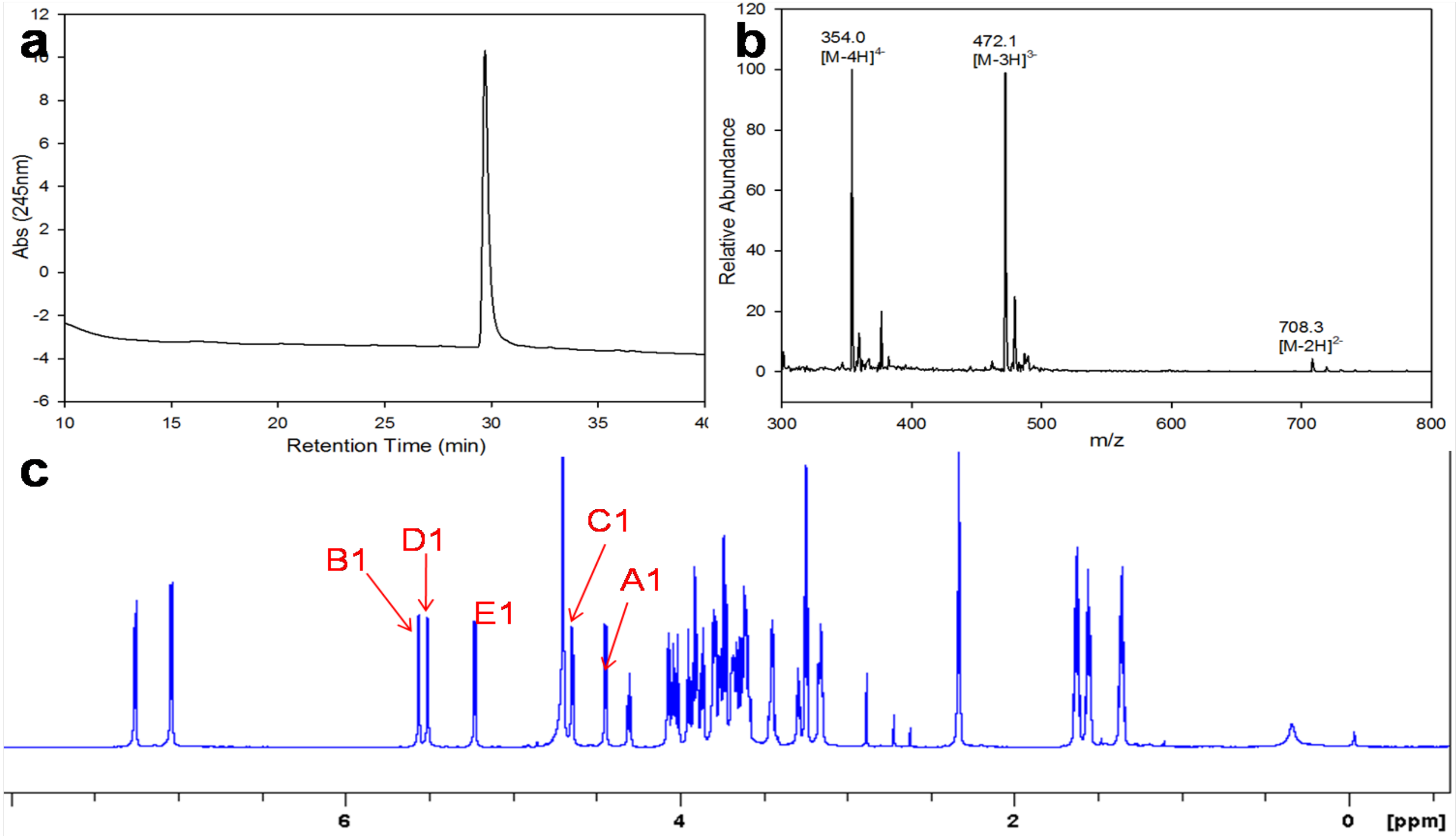

Supplementary Fig. S197. HPLC analysis (a), MS spectrum (b) and ^{1}H NMR spectrum (c) of compound **66**.

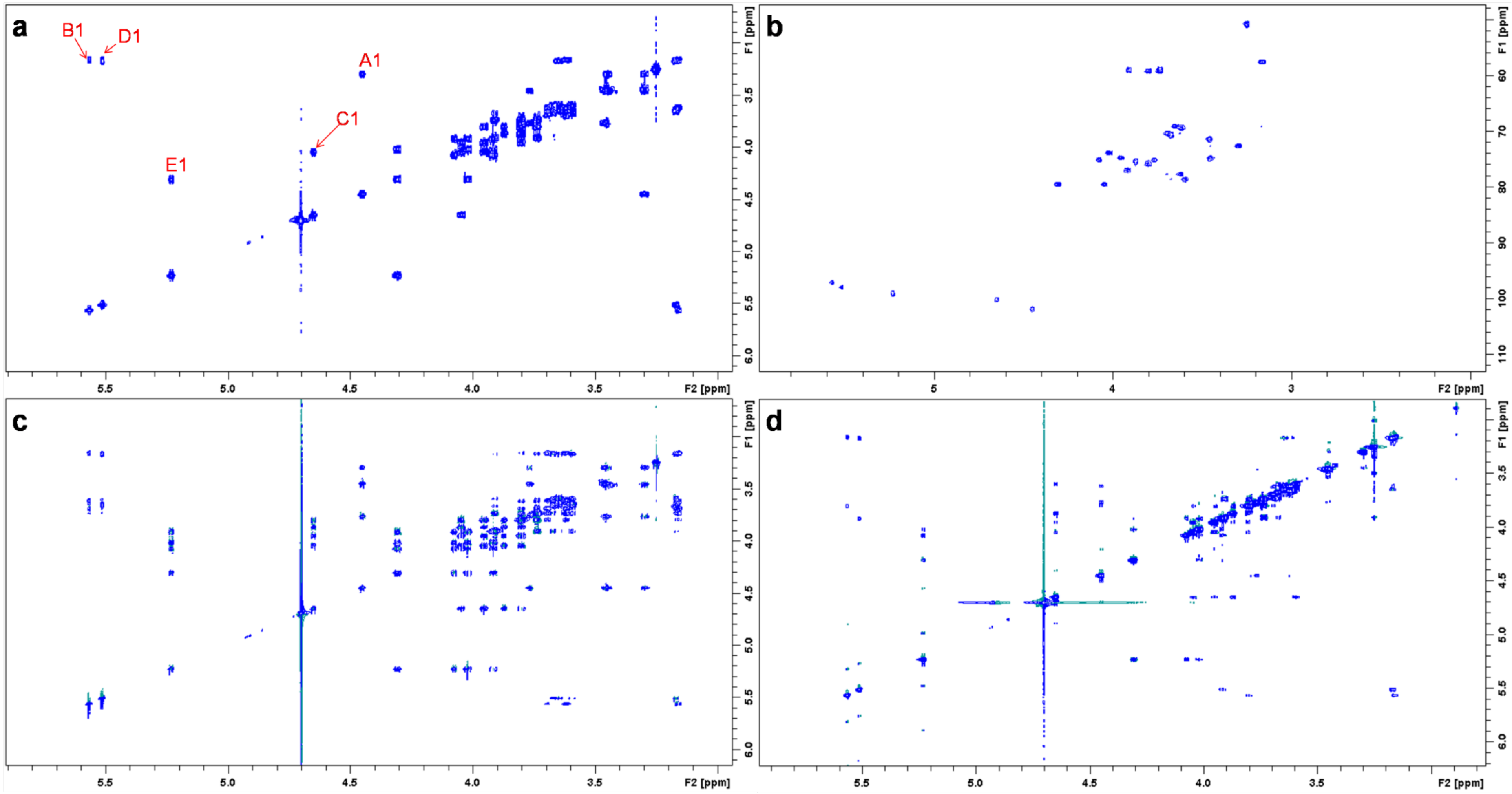

Supplementary Fig. S198. 2D NMR COSY (a), HSQC (b), TOCSY (c) and NOE (d) spectra of compound **66.**
